# Automatic Clustering Using Multi-objective Particle Swarm and Simulated Annealing

**DOI:** 10.1371/journal.pone.0130995

**Published:** 2015-07-01

**Authors:** Ahmad Abubaker, Adam Baharum, Mahmoud Alrefaei

**Affiliations:** 1 School of Mathematical Sciences, University Sains Malaysia, 11800 USM Penang, Malaysia; 2 Department of Mathematics & Statistics, Al-Imam Muhammad Ibn Saud Islamic University, P.O. Box 90950, 11623 Riyadh, Saudi Arabia; 3 Departments of Mathematics & Statistics, Jordan University of Science and Technology, Irbid 22110, Jordan; Southwest University, CHINA

## Abstract

This paper puts forward a new automatic clustering algorithm based on Multi-Objective Particle Swarm Optimization and Simulated Annealing, “MOPSOSA”. The proposed algorithm is capable of automatic clustering which is appropriate for partitioning datasets to a suitable number of clusters. MOPSOSA combines the features of the multi-objective based particle swarm optimization (PSO) and the Multi-Objective Simulated Annealing (MOSA). Three cluster validity indices were optimized simultaneously to establish the suitable number of clusters and the appropriate clustering for a dataset. The first cluster validity index is centred on Euclidean distance, the second on the point symmetry distance, and the last cluster validity index is based on short distance. A number of algorithms have been compared with the MOPSOSA algorithm in resolving clustering problems by determining the actual number of clusters and optimal clustering. Computational experiments were carried out to study fourteen artificial and five real life datasets.

## Introduction

Data clustering is an important task in the field of unsupervised datasets. The clustering technique distributes the dataset into clusters of similar features [1]. To solve a clustering problem, the number of clusters that fits a dataset must be determined, and the objects for these clusters must be assigned appropriately. The number of clusters may or may not be known, thereby making it difficult to find the best solution to the clustering problem. As such, the clustering problem can be viewed as an optimization problem. This challenge has led to the proposal of many automatic clustering algorithms in previous literature; these algorithms estimate the appropriate number of clusters and appropriately partition a dataset into these clusters without the need to know the actual number of clusters [2–8]. Most of these algorithms rely exclusively on one internal evaluation function (validity index). The validity index has an objective function to evaluate the various characteristics of clusters, which illustrates the clustering quality and accuracy of the clustering solutions [9]. Nevertheless, the single evaluation function is often ineligible to determine the appropriate clusters for a dataset, thus giving an inferior solution [10]. Accordingly, the clustering problem is structured as a multi-objective optimization problem wherein different validity indices can be applied and evaluated simultaneously.

Several automatic multi-objective clustering algorithms are proposed in literature to solve the clustering problem. Evolution appeared in this area after Handl and Knowles [3] proposed an evolutionary approach called multi-objective clustering with automatic K determination (MOCK). For some of the automatic multi-objective clustering algorithms related to MOCK, can refer to [11–13]. A multi-objective clustering technique inspired by MOCK named VAMOSA, which is based on simulated annealing as the underlying optimization strategy and the point symmetry-based distance, was proposed by Saha and Bandyopadhyay [5].

How to deal with various shapes of datasets (hyper spheres, linear, spiral, convex, and non-convex), overlapping datasets, datasets with a small or large number of clusters, and datasets that have objects with small or large dimensions without providing the proper clustering or knowing the cluster number is a challenge. Saha and Bandyopadhyay [8] developed two multi-objective clustering techniques (GenClustMOO and GenClustPESA2) by using a simulated annealing-based multi-objective optimization technique and the concept of multiple centers to each cluster that can deal with different types of cluster structures. GenClustMOO and GenClustPESA2 were compared with MOCK [3], VGAPS [4], K-means (KM) [14], and single-linkage clustering technique (SL) [15] using numerous artificial and real-life datasets of diverse complexities. However, these algorithms did not give the desired high accuracy in clustering datasets.

The current study proposes an automatic clustering algorithm, namely, hybrid multi-objective particle swarm optimization with simulated annealing (MOPSOSA), which deals with different sizes, shapes, and dimensions of datasets and an unknown number of clusters. The Numerical results of the proposed algorithm are shown to perform better than those of the GenClustMOO [8] and GenClustPESA2 [8] methods in terms of clustering accuracy (see the Results and Discussions Section). In order to deal with any dataset and qualification to determine appropriate clusters and obtain good solutions with high accuracy, combinatorial particle swarm optimization II [7]is developed to deal with three different cluster validity indices, simultaneously. The first cluster validity index is the Davies-Bouldin index (*DB*-index) [16], which is based on Euclidean distance; the second one is symmetry-based cluster validity index (*Sym*-index) [4], which is based on point symmetry distance; and the last one is a connectivity-based cluster validity index (*Conn*-index) [17], which is based on short distance. If no change exists in a particle position or when it is moved to a bad position, then the MOPSOSA algorithm uses MOSA [18] to improve the searching particle. The MOPSOSA algorithm also utilizes KM method [14] to improve the selection of the initial particle position because of its significance in the overall performance of the search process. It creates a large number of Pareto optimal solutions through a trade-off between the three different validity indices. Therefore, the idea of sharing fitness [19] is incorporated in the proposed algorithm to maintain diversity in the repository that contains Pareto optimal solutions. Pareto optimal solutions are important for decision makers to choose from. Furthermore, to comply with the decision-maker requirements, the proposed algorithm utilizes a semi-supervised method [20] to provide a single best solution from the Pareto set. The performance of MOPSOSA is compared with the performances of three automatic multi-objective clustering techniques, namely, GenClustMOO [8], GenClustPESA2 [8], and MOCK [3], and with those of three single-objective clustering techniques, namely, VGAPS [4], KM [14], and SL [15], using 14 artificial and 5 real-life datasets.

The reminder of this paper is structured as follows; Section 2 describes the multi-objective clustering problem; Section 3 illustrates the proposed MOPSOSA algorithm in details; Section 4 presents the datasets used in the numerical experiments, the evaluation of clustering quality, and the setting of the parameters for the MOPSOSA algorithm; Section 5 includes discussion of the results; Finally, concluding remarks are given in Section 6.

## Clustering Problem

The clustering problem is defined as follows: Consider the dataset *P* = {*p*_1_,*p*_2_,…,*p*_*n*_}, where *p*_*i*_ = (*p*_i1_,*p*_i2_,…,*p*_*id*_) is a feature vector of *d*-dimensions and also referred to as the object, *p*_*ij*_ is the feature value of object *i* at dimension *j*, and *n* is the number of objects in *P*. The clustering of *P* is the partitioning of *P* into *k* clusters {*C*_1_,*C*_2_,…,*C*_*k*_} with the following properties:

$$\cup_{i=1}^{k}C_{i}=P$$ (1)

$$C_{i}\cap C_{j}=\phi ,\;i\neq j,\;i=1,2,\ldots ,k,\;j=1,2,\ldots ,k$$ (2)

$$C_{i}\neq \phi ,\;i=1,2,\ldots ,k$$ (3)

The clustering optimization problem with one objective function for the clustering problem can be formed as follows: $\underset{C\in \Theta }{min/max}\;f(C)$ such that Eqs (1) to (3) are satisfied, where *f* is the validity index function, Θ is the feasible solutions set that contains all possible clustering for the dataset *P* of *n* objects into *k* clusters, *C* = {*C*_1_,*C*_2_,…,*C*_*k*_} and k = 2,3,…,*n*‒1.

The multi-objective clustering problem for *S* different validity indices is defined as follows:

$$\underset{C\in \Theta }{min}\;F(C)=[f_{1}(C),f_{2}(C),\ldots ,f_{S}(C)].$$ (4)

where *F*(*C*) is a vector of *S* validity indices. Note that there may be no solution that minimizes all the functions *f*_*i*_(*C*). Therefore, the aim is to identify the set of all non-dominant solutions.

**Definition**: Consider *C* and C* as two solutions in the feasible solutions set Θ, the solution *C* is said to be dominated by the solution C* if and only if *f*_*i*_(C*) ≤ *f*_*i*_(*C*), ∀ *i* = 1,…,*S* and *f*_*i*_(C*) < *f*_*i*_(*C*) for at least one *i*. Otherwise, *C* is said to be non-dominated by C*.

The Pareto optimal set is a set that includes all non-dominated solutions in the feasible solutions set Θ.

## The Proposed MOPSOSA Algorithm

Simulated annealing requires more calculation time than does particle swarm optimization [21]. The former requires low variations of temperature parameters to obtain a global solution [22]. Some of the particles may become stagnant and remain unchanged, especially when the objective functions of the best personal position and the best global position are similar [21]. As such, the particle cannot jump out, which in turn causes convergence toward the local solution and the loss of its capability to search for the optimal Pareto set. This phenomenon is a disadvantage in comparison with simulated annealing, which can jump away from a local solution. The proposed MOPSOSA algorithm, as previously mentioned, is a hybrid algorithm that merges the advantages of fast calculation and convergence in particle swarm optimization with the capability to evade local solutions in simulated annealing.

The clustering solution *X*_*i*_ is described using label-based integer encoding [23]. Each particle position is a clustering solution. The particle position $X_{i}^{t}$ and velocity $V_{i}^{t}$ are presented as vectors with *n* components $X_{i}^{t}=(X_{i1}^{t},X_{i2}^{t},\ldots ,X_{in}^{t})$ and $V_{i}^{t}=(V_{i1}^{t},V_{i2}^{t},\ldots ,V_{in}^{t})$ at time *t*, *i* = 1,…,*m*, where *n* is the number of data objects, and *m* is the number of particles (swarm size). The position component $X_{ij}^{t}\in \{1,\ldots ,K_{i}^{t}\}$ represents the cluster number of *j* ^th^ object in *i* ^th^ particle, and $V_{ij}^{t}\in \{0,\ldots ,K_{i}^{t}\}$ represents the motion of *j* ^th^ object in *i* ^th^ particle, where $K_{i}^{t}\in \{K_{min},\ldots ,K_{max}\}$ is the number of clusters related to particle *i* at time *t* (where *K*_min_ and *K*_max_ are the minimum and maximum number of clusters, respectively; the default value of *K*_min_ is 2; and *K*_max_ is $\sqrt{n}+1$ unless it is manually specified) [24]. The best previous position of *i* ^th^ particle at iteration *t* is represented as $XP_{i}^{t}=(XP_{i1}^{t},XP_{i2}^{t},\ldots ,XP_{in}^{t})$. The leader position chosen from the repository of Pareto sets for *i* ^th^ particle at iteration *t* is represented by $XG_{i}^{t}=(GP_{i1}^{t},GP_{i2}^{t},\ldots ,GP_{in}^{t})$.

**Fig 1:**
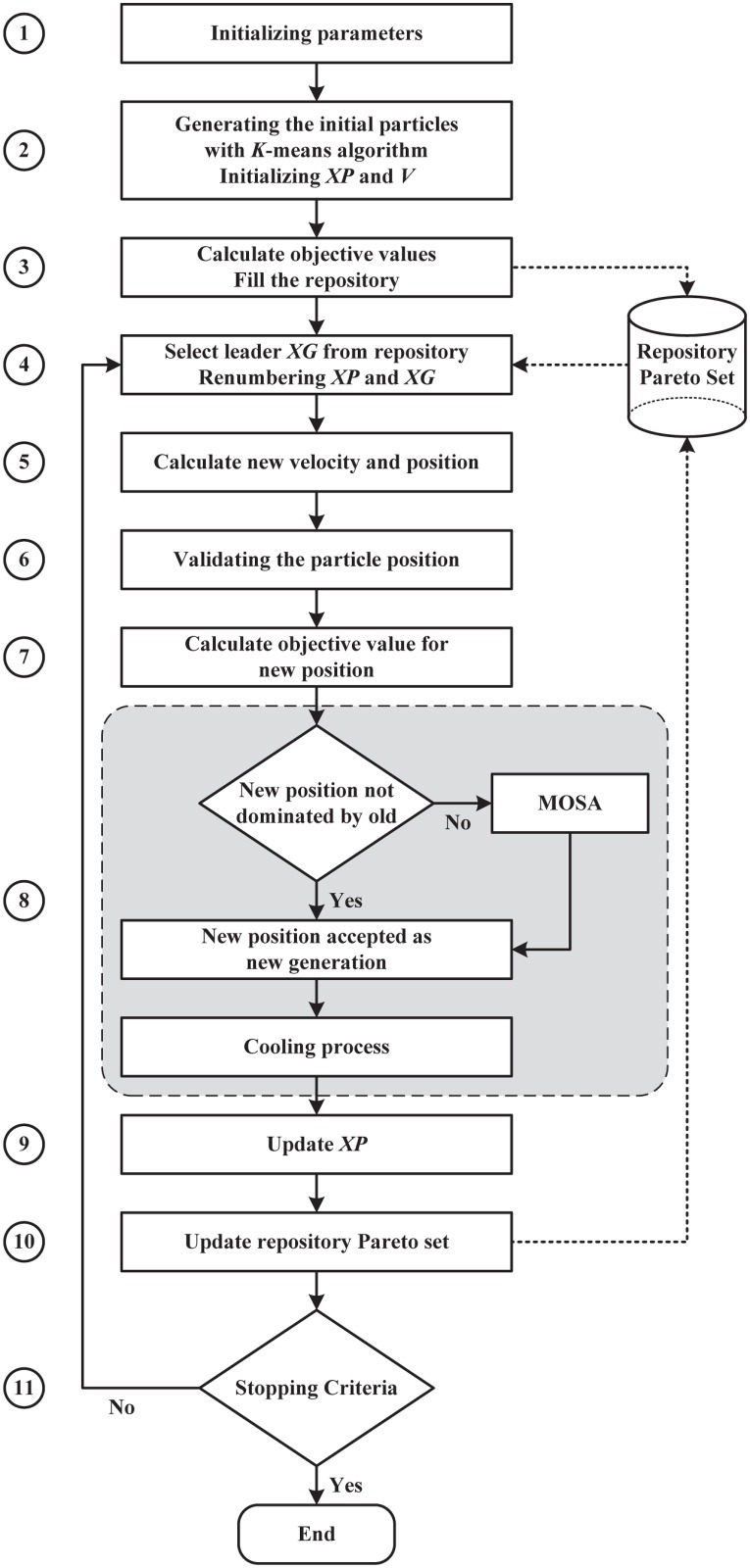
Flowchart for the proposed MOPSOSA algorithm.

The flowchart in Fig 1 illustrates the general process of the MOPSOSA algorithm. The process of the algorithm is described in the following 11 steps:

Step 1: The algorithm parameters, such as swarm size *m*, number of iterations *Iter*, maximum and minimum numbers of clusters, velocity parameters, initial cooling temperature *T*_0_, and *t* = 0, are initialized.
Step 2: The initial particle position $X_{i}^{t}$ using KM method [14], initial velocity $V_{i}^{t}=0$, and initial $XP_{i}^{t}=X_{i}^{t}$, *i* = 1,…,*m* are generated.
Step 3: The objective functions $f_{1}(X_{i}^{t}),\ldots ,f_{S}(X_{i}^{t})$, *i* = 1,…,*m*, where *S* is the number of objective functions, are computed. The repository of Pareto sets is filled with all non-dominated $XP_{i}^{t}$, *i* = 1,…,*m* based on a fitness-sharing basis.
Step 4: The leader $XG_{i}^{t}$ from the repository of Pareto sets nearest to current $X_{i}^{t}$ is selected. The clusters in $XP_{i}^{t}$ and $XG_{i}^{t}$ are renumbered on the basis of their similarity to the clusters in $X_{i}^{t}$, *i* = 1,…,*m*.
Step 5: The new *Vnew*_*i*_ and *Xnew*_*i*_, *i* = 1,…,*m*, are computed using $XG_{i}^{t}$, $XP_{i}^{t}$, $X_{i}^{t}$, and $V_{i}^{t}$.
Step 6: The validity of *Xnew*_*i*_, *i* = 1,…,*m* is checked, and the correction process is applied if it is not valid.
Step 7: The objective functions *f*_1_(*Xnew*_*i*_),…,*f*_*s*_(*Xnew*_*i*_) and $f_{1}(X_{i}^{t}),\ldots ,f_{s}(X_{i}^{t})$, *i* = 1,…,*m* are computed.
Step 8: A dominance check for *Xnew*_*i*_, *i* = 1,…,*m* is performed, that is, if *Xnew*_*i*_ is non-dominated by $X_{i}^{t}$, then $X_{i}^{t+1}=Xnew_{i}^{}$ and $V_{i}^{t+1}=Vnew_{i}^{}$; otherwise, the MOSA technique is applied and $X_{i}^{t+1}=X_{i}^{MOSA}$ and $V_{i}^{t+1}=V_{i}^{MOSA}$, *i* = 1,…,*m*, where $X_{i}^{MOSA}$ and $V_{i}^{MOSA}$ are the position and velocity particles respectively obtained by applying the MOSA technique. The MOSA is discussed in details in section MOSA Technique below. Upon completion of the generation of new positions for all particles, the cooling temperature *T*_t+1_ is updated.
Step 9: The new $XP_{i}^{t+1}$, *i* = 1,…,*m* is identified.
Step 10: The Pareto set repository is updated.
Step 11: *t* = *t* + 1 is set; if *t* ≥ *Iter*, then the algorithm is stopped and the Pareto set repository contains the Pareto solutions; otherwise, go to step 4.

The following sections will elucidate the steps of the MOPSOSA algorithm.

### Particles swarm initialization

**Fig 2:**
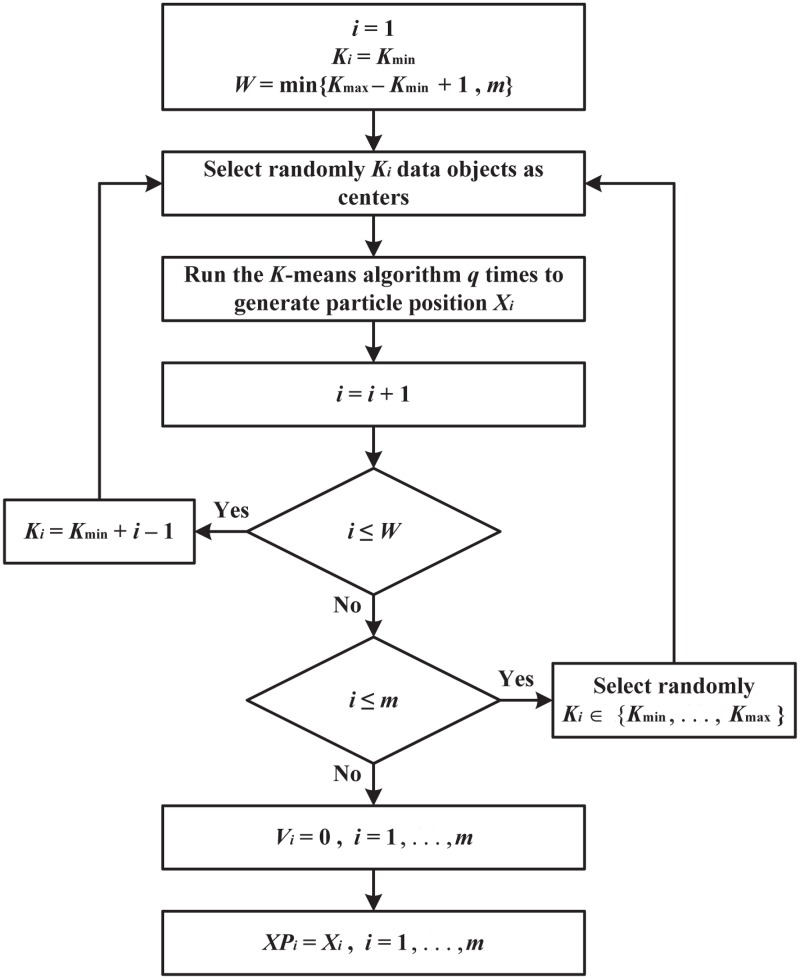
Flowchart for initializing particle swarm.

Initial particles are generally considered one of the success factors in particle swarm optimization that affect the quality of the solution and the speed of convergence. Hence, the MOPSOSA algorithm employs KM method as a means to improve the generation of the initial swarm of particles. Fig 2 depicts a flowchart for the generation of *m* particles. Starting with *i* = 1 and *W* = min{*K*_max_−*K*_min_+1,*m*}, if *W* = *m*, then *m* particles will be generated by KM method with the number of clusters *K*_*i*_ = *K*_min_+*i*−1, *i* = 1,…,*m*. If *W* = *K*_max_−*K*_min_+1, then the first *W* particles will be generated by KM with the number of clusters *K*_*i*_ = *K*_min_+*i*−1, *i* = 1,…,*W*, and the other particle will be generated by KM with the number of clusters *K*_*i*_, *i* = *W*+1,…*m* selected randomly between *K*_min_ and *K*_max_. For each particle, the initial velocities are selected to be zero *V*_*i*_ = 0, *i* = 1,…,*m*, and the initial *XP*_*i*_ is equal to the current position *X*_*i*_ for all *i* = 1,…,*m*.

### Objective functions

The proposed algorithm uses three types of cluster validity indices as objective functions to achieve optimization. These validity indices, *DB*-index, *Sym*-index, and *Conn*-index, apply three different distances, namely, Euclidean distance, point symmetric distance, and short distance, respectively. Each validity index indicates a different aspect of good solutions in clustering problems. These validity indices are described below.

#### DB-index

This index was developed by Davies—Bouldin [16] which is a function of the ratio of the sum of within-cluster objects (intra-cluster distance) and between cluster separation (inter-cluster distance). The within *i* ^th^ cluster *C*_*i*_, *S*_i,q_ is calculated using Eq (5). The distance between clusters *C*_*i*_ and *C*_*j*_ is denoted by *d*_*ij*,*t*_, which is computed using Eq (6).

$$S_{i,q}={\left(\frac{1}{n_{i}}\overset{}{\underset{p\in C_{i}}{\sum }}\|p-c_{i}{\|}_{2}^{q}\right)}^{\frac{1}{q}}$$ (5)

$$d_{ij,t}={\left\|c_{i}-c_{j}\right\|}_{t}$$ (6)

where *n*_*i*_ = |*C*_*i*_| is the number of objects in cluster *C*_*i*_, *c*_*i*_ is the cluster center of cluster *C*_*i*_ and is defined as $c_{i}=\frac{1}{n_{i}}\sum_{p\in C_{i}}p$, and *q* and *t* are positive integer numbers. *DB* is defined as:

$$DB=\frac{1}{k}\overset{k}{\underset{i=1}{\sum }}R_{i,qt}$$ (7)

where $R_{i,qt}=\underset{j,j\neq i}{\text{max}}\left\{\frac{S_{i,q}+S_{j,q}}{d_{ij,t}}\right\}$. A small value of *DB* means a good clustering result.

#### Sym-index

The recently developed point symmetry distance *d*_*ps*_(*p*,*c*) is employed in this cluster validity index *Sym*, which measures the overall average symmetry in connection with the cluster centers [4]. It is defined as follows. Let *p* be a point, and the reflected symmetrical point of *p* with respect to a specific center *c* is 2*c* − *p* and is denoted by p*. Let *knear* unique nearest neighbors to p* be at the Euclidean distances of *d*_*i*_, *i* = 1,…,*knear*. The point symmetric distance is defined as:

$$d_{ps}(p,c)=d_{sym}(p,c)\times d_{e}(p,c)=\frac{\sum_{i=1}^{knear}d_{i}}{knear}\times d_{e}(p,c)$$ (8)

where *d*_*e*_(*p*,*c*) is the Euclidean distance between the point *p* and the center *c* and *d*_*sym*_(*p*,*c*) is a symmetric measure of *p* with respect to *c*, which is defined as $\sum_{i=1}^{knear}d_{i}/knear$. In this study, *knear* = 2. The cluster validity function is defined as

$$Sym=(\frac{1}{k}\times \frac{1}{\varepsilon_{k}}\times D_{k})$$ (9)

where $\varepsilon_{k}=\sum_{i=1}^{k}E_{i}$, $E_{i}=\sum_{j=1}^{n_{i}}d_{ps}^{*}(p_{j}^{i},c_{i})$, $p_{j}^{i}$ is the *j* ^th^ object of cluster *i*, and $D_{k}={max}_{i,j=1}^{k}\|c_{i}-c_{j}\|$ is the maximum Euclidean distance between the two centers among all cluster pairs. Eq (8) is used with some constraint to compute $d_{ps}^{*}(p_{j}^{i},c_{i})$. The *knear* nearest neighbors of $p_{j}^{*}$ and $p_{j}^{i}$ should belong to the *i* ^th^ cluster, where $p_{j}^{*}$ is the reflected point of the point $p_{j}^{i}$ with respect to *c*_*i*_. A large value for *Sym*-index means that the actual number of clusters and proper partitioning are obtained.

#### *Conn*-index

The third cluster validity index used in this study is proposed by Saha and Bandyopadhyay [17], it depends on the notion of cluster connectedness. To compute *Conn*-index, the the relative neighborhood graph [25] structuring for the dataset has to be conducted first. Subsequently, the short distance between two points *x* and *y* is denoted by *d*_*short*_(*x*,*y*) and is defined as follows:

$$d_{short}(x,y)=\overset{npath}{\underset{i=1}{min}}\overset{ned_{i}}{\underset{j=1}{max}}w(ed_{j}^{i})$$ (10)

where *npath* is the number of all paths between *x* and *y* in the RNG structuring; *ned*_*i*_ is the number of edges along *i* ^th^ path, *i* = 1,…,*npath*; $ed_{j}^{i}$ is *j* ^th^ edge in *i* ^th^ path, *j* = 1,…,*ned*_*i*_ and *i* = 1,…,*npath*; and $w(ed_{j}^{i})$ is the edge weight of the edge $ed_{j}^{i}$. The edge weight $w(ed_{j}^{i})$ is equal to the Euclidean distance between *a* and *b*, *d*_*e*_(*a*,*b*), where *a* and *b* are the end points of the edge $ed_{j}^{i}$.

The cluster validity index *Conn* developed by Saha and Bandyopadhyay [17] is defined as follows:

$$Conn=\frac{\overset{k}{\underset{i=1}{\sum }}\overset{n_{i}}{\underset{j=1}{\sum }}d_{short}(p_{j}^{i},m_{i})}{n\left(\overset{k}{\underset{i,j=1,\;i\neq j}{\text{min}}}d_{short}(m_{j},m_{i})\right)}$$ (11)

where *m*_*i*_ is the medoid of the *i* ^th^ cluster that is equal to the point with the minimum average distance to all points in the *i* ^th^ cluster $m_{i}=p_{\text{minindex}}^{i}$, and $\text{minindex}=\text{arg}{\text{min}}_{t=1}^{n_{i}}\left(\overset{n_{i}}{\underset{j=1}{\sum }}d_{e}(p_{t}^{i},p_{j}^{i})/n_{i}\right)$. The minimum value of *Conn*-index means the clusters interconnected internally and separately from each other.

After the particles have been moved to a new position, the three objective functions are computed for each particle in the swarm. The objective functions for a particle position *X* are {*DB*(*X*),1/*Sym*(*X*),*Conn*(*X*)}. The three objectives are minimized simultaneously using MOPSOSA algorithm.

### XP updating

The previous best position of *i* ^th^ particle at iteration *t* is updated by non-dominant criteria. $XP_{i}^{t}$ is compared with the new position $X_{i}^{t+1}$. Three cases of this comparison are considered.

- If $XP_{i}^{t}$ is dominated by $X_{i}^{t+1}$, then $XP_{i}^{t+1}=X_{i}^{t+1}$.
- If $X_{i}^{t+1}$ is dominated by $XP_{i}^{t}$, then $XP_{i}^{t+1}=XP_{i}^{t}$.
- If $XP_{i}^{t}$ and $X_{i}^{t+1}$ are non-dominated, then one of them will be chosen randomly as $XP_{i}^{t+1}$.

This update occurs on each particle.

### Repository updating

The repository is utilized as a guide by MOPSOSA algorithm for the swarm toward the Pareto front. The non-dominated particle positions are stored in the repository. To preserve the diversity of non-dominated solutions in the repository, sharing fitness [19] is a good method to control the acceptance of new entries into the repository when it is full. Fitness sharing was used by Lechuga and Rowe [26] in multi-objective particle swarm optimization. In each iteration, the new non-dominated solutions are added into the external repository and elimination of the dominated solutions. In case the non-dominated solutions are increased than the size of the repository, the fitness sharing is calculated for all non-dominated solutions. The solutions that have largest values of fitness sharing are selected to fill the repository.

### Cluster re-numbering

The re-numbering process is designed to eliminate the redundant particles that represent the same solution. The proposed MOPSOSA algorithm employs the re-numbering procedure designed by Masoud et al. [7]. This procedure uses a similarity function to measure the degree of similarity between the clusters of two input solutions $X_{i}^{t}$ and $XP_{i}^{t}$ (or $XG_{i}^{t}$). The two clusters that are most similar are matched. Any cluster in $XP_{i}^{t}$ (or $XG_{i}^{t}$) not matched to any cluster $X_{i}^{t}$ will use the unused number in the clustering numbering. MOPSOSA algorithm uses the similarity function known as Jaccard coefficient [27], which is defined as follows:

$$Sim(C_{j},{\overset{⌢}{C}}_{k})=\frac{n_{11}}{n_{11}+n_{10}+n_{01}}$$ (12)

where *C*_*j*_ is *j* ^th^ cluster in $X_{i}^{t}$, ${\overset{⌢}{C}}_{k}$ is *k* ^th^ cluster in $XP_{i}^{t}$, *n*_11_ is the number of objects that exist in both *C*_*j*_ and ${\overset{⌢}{C}}_{k}$, *n*_10_ is the number of objects that exist in *C*_*j*_ but does not exist in ${\overset{⌢}{C}}_{k}$, and *n*_01_ is the number of objects that do not exist in *C*_*j*_ but exist in ${\overset{⌢}{C}}_{k}$.

### Velocity computation

MOPSOSA algorithm employs the expressions and operators modified by Masoud et al. [7]. The new velocity for particle *i* at iteration *t* is calculated as follows:

$$V_{i}^{t+1}=(W⊗V_{i}^{t})⊕((R_{1}⊗(XP_{i}^{t}⊖X_{i}^{t}))⊕(R_{2}⊗(XG_{i}^{t}⊖X_{i}^{t})))$$ (13)

where *W*, *R*_1_, and *R*_2_ are the vectors of *n* components with values 0 or 1 that are generated randomly with a probability of *w*, *r*_1_, and *r*_2_, respectively. The operations ⊗, ⊕, and ⊖ are the multiplication, merging, and difference, respectively.

- **Difference operator⊖**

The difference operation calculates the difference between $X_{i}^{t}$ and $XP_{i}^{t}$ (or *XG* ^*t*^). Let $\lambda P_{i}^{t}=(\lambda p_{i1}^{t},\ldots ,\lambda p_{in}^{t})=XP_{i}^{t}⊖X_{i}^{t}$, and $\lambda G_{i}^{t}=(\lambda g_{i1}^{t},\ldots ,\lambda g_{in}^{t})=XG_{}^{t}⊖X_{i}^{t}$ be defined as follows:

$$\lambda p_{ij}^{t}=\{\begin{array}{ll} XP_{ij}^{t} & \text{if }X_{ij}^{t}\neq XP_{ij}^{t}\\ 0 & \text{otherwise} \end{array}$$ (14)

$$\lambda g_{ij}^{t}=\{\begin{array}{ll} XG_{j}^{t} & \text{if }X_{ij}^{t}\neq XG_{j}^{t}\\ 0 & \text{otherwise} \end{array}$$ (15)

- **Multiplication operator ⊗**

The multiplication operator is defined as follows: let *A* = (*a*_*1*_,…,*a*_*n*_) and *B* = (*b*_*1*_,…,*b*_*n*_) are two vectors of *n* components, then *A*⊗*B* = (*a*_1_ *b*_1_,…,*a*_*n*_ *b*_*n*_).

- **Merging operator ⊕**

The merging operator is defined as follows: let *A* = (*a*_*1*_,…,*a*_*n*_) and *B* = (*b*_*1*_,…,*b*_*n*_) be two vectors of *n* components, then *C* = *A*⊕*B* = (*c*_1_,*c*_2_,…,*c*_*n*_), where

$$c_{i}=\{\begin{array}{ll} a_{i} & \text{if }a_{i}\neq 0\text{ and }b_{i}=0\\ b_{i} & \text{if }a_{i}=0\text{ and }b_{i}\neq 0\\ a_{i}\text{ or }b_{i}\text{ randomly} & \text{if }a_{i}\neq 0\text{ and }b_{i}\neq 0\\ 0 & \text{otherwise} \end{array}$$ (16)

### Position computation

MOPSOSA algorithm employs the definition to generate new positions, as proposed by Masoud et al. [7]. The new position is generated from the velocity as follows:

$$X_{ij}^{t+1}=\{\begin{array}{ll} V_{ij}^{t} & \text{if }V_{ij}^{t+1}\neq 0\\ r & \text{otherwise} \end{array}$$ (17)

where *r* is an integer random number in $\left[1,K_{i}^{t}+1\right]$ and $K_{i}^{t}+1<K_{max}$. This property enables the particle to add new clusters. The previous operators and the differences in cluster number of $X_{i}^{t}$, $XP_{i}^{t}$, and *XG* ^*t*^ lead to the addition or removal of some of the clusters in the output of the new position $X_{i}^{t+1}$. Sometimes an empty cluster may exist, which leads to invalid particle position. Such an instance can be avoided by exposing the particle to reset the numbering clusters. The re-numbering process works by encoding the largest cluster number to the smallest unused one.

### MOSA technique

**Fig 3:**
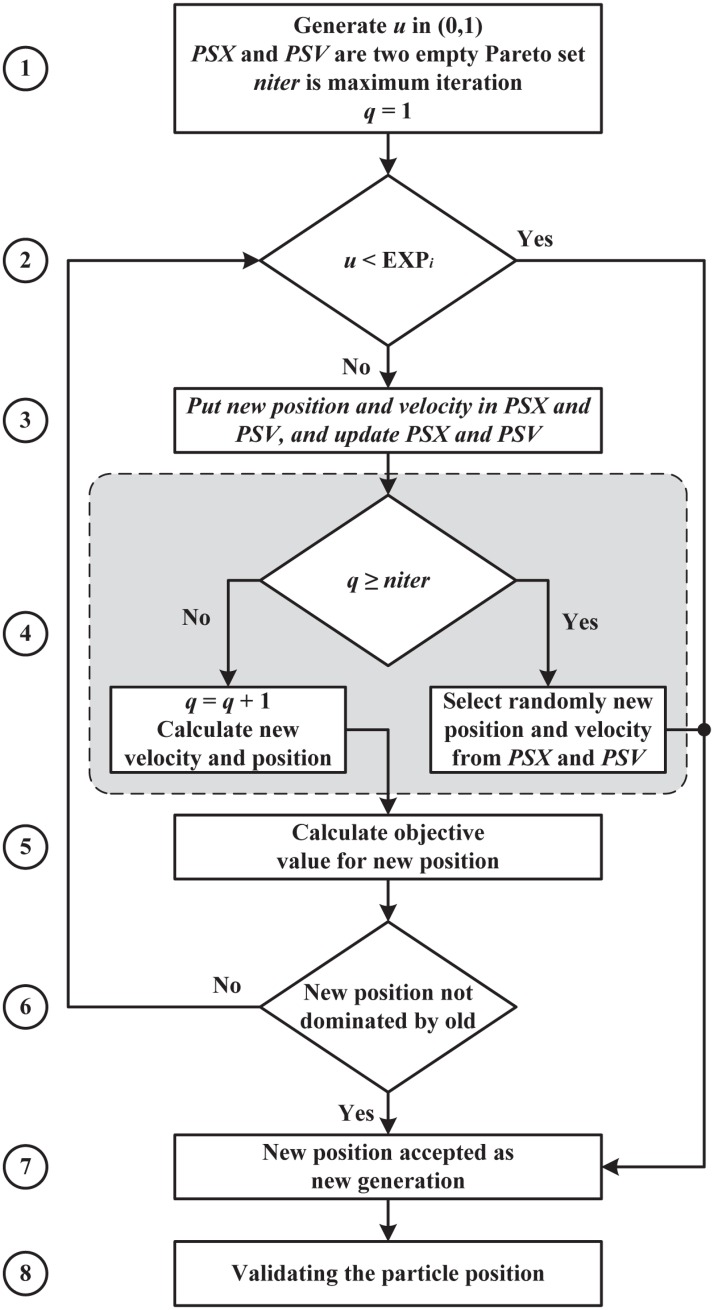
Flowchart for the MOSA technique applied in MOPSOSA.

MOSA method [18] is applied in the MOPSOSA algorithm at iteration *t* for particle *i* in case $X_{i}^{t}$ dominates the new position *Xnew*_*i*_. Fig 3 presents the flowchart for the MOSA technique applied in MOPSOSA. The procedure for the MOSA technique is explained in eight steps below.

1. Step 1: Let *PSX* and *PSV* be two empty sets, *niter* is a maximum number of iteration, and *q* = 0.
2. Step 2: Evaluate $EXP_{q}=\prod_{j=1}^{s}exp(-{\left[f_{j}(Xnew_{i}^{})-f_{j}(X_{i}^{t})\right]}^{+}/T_{t})$, where the cooling temperature *T*_*t*_ is updated in step 8 of MOPSOSA algorithm. Generate uniform random number *u*∈(0,1), if *u*<*EXP*_*q*_, go to step 7. Otherwise, proceed to the next step.
3. Step 3: Add *Xnew*_*i*_ to *PSX* and *Vnew*_*i*_ to *PSV*, then *PSX* and *PSV* are updated to include only non-dominant solutions.
4. Step 4: If *q*≥*niter*, then choose a solution randomly from *PSX* as the new particle position *Xnew*_*i*_ and the corresponding velocity *Vnew*_*i*_ from *PSV*, and proceed to step 7. Otherwise, *q* = *q*+1, and generate the new velocity *Vnew*_*i*_ and position *Xnew*_*i*_ from the old position $X_{i}^{t}$.
5. Step 5: Calculate the objective function *f*_1_(*Xnew*_*i*_),…,*f*_*s*_(*Xnew*_*i*_), and $f_{1}(X_{i}^{t}),\ldots ,f_{S}(X_{i}^{t})$.
6. Step 6: Perform a dominance check for *Xnew*_*i*_, if *Xnew*_*i*_ is non-dominated by $X_{i}^{t}$, then proceed to step 7. Otherwise go to step 2.
7. Step 7: The new position and velocity *Xnew*_*i*_ and *Vnew*_*i*_ are accepted as the new generation of $X_{i}^{MOSA}$ and $V_{i}^{MOSA}$, respectively, $X_{i}^{MOSA}=Xnew_{i}^{}$ and $V_{i}^{MOSA}=Vnew_{i}.$
8. Step 8: Check the validity for $X_{i}^{MOSA}$, and apply the re-numbering process if it is invalid. Return $X_{i}^{MOSA}$ and $V_{i}^{MOSA}$.

### Selection of the best solution

In general, a Pareto set containing several non-dominated solutions is provided on the final run of multi-objective problems [28]. Each non-dominated solution introduces a pattern of clustering for the given dataset. The semi-supervised method proposed by Saha and Bandyopadhyay [20] is utilized in the MOPSOSA algorithm to select the best solution from the Pareto optimal set. This semi-supervised approach can only be applied when the cluster labels of some points in the dataset are known. The misclassification value is computed by using the Minkowski score *MS* [29]. Let *T* be the actual solution and *C* be the selected solution; hence, *MS* is defined as follows:

$$MS(T,C)=\sqrt{\frac{n_{01}+n_{10}}{n_{11}+n_{10}}}$$ (18)

The low values of *MS* are “better” with the optimal value for *MS* set as 0.

## Experimental Study

This section presents the datasets used for the experiment, the measurement of the accuracy solution, and parameters settings of the proposed algorithm.

### Experimental datasets

The MOPSOSA algorithm is examined on 14 artificial and 5 real-life datasets (S1 File). Table 1 displays the types of datasets, the number of points (objects), the dimensions (features), and the number of clusters. Further details on these datasets are provided below.

**Table 1:** Description of the artificial and real-life datasets.

Dataset	# Points	Dimension	# Clusters
Sph_5_2	250	2	5
Sph_4_3	400	3	4
Sph_6_2	300	2	6
Sph_10_2	500	2	10
Sph_9_2	900	2	9
Pat1	557	2	3
Pat2	417	2	2
Long1	1000	2	2
Sizes5	1000	2	4
Spiral	1000	2	2
Square1	1000	2	4
Square4	1000	2	4
Twenty	1000	2	20
Fourty	1000	2	40
Iris	150	4	3
Cancer	683	9	2
Newthyroid	215	5	3
LiverDisorder	345	6	2
Glass	214	9	6

**Fig 4:**
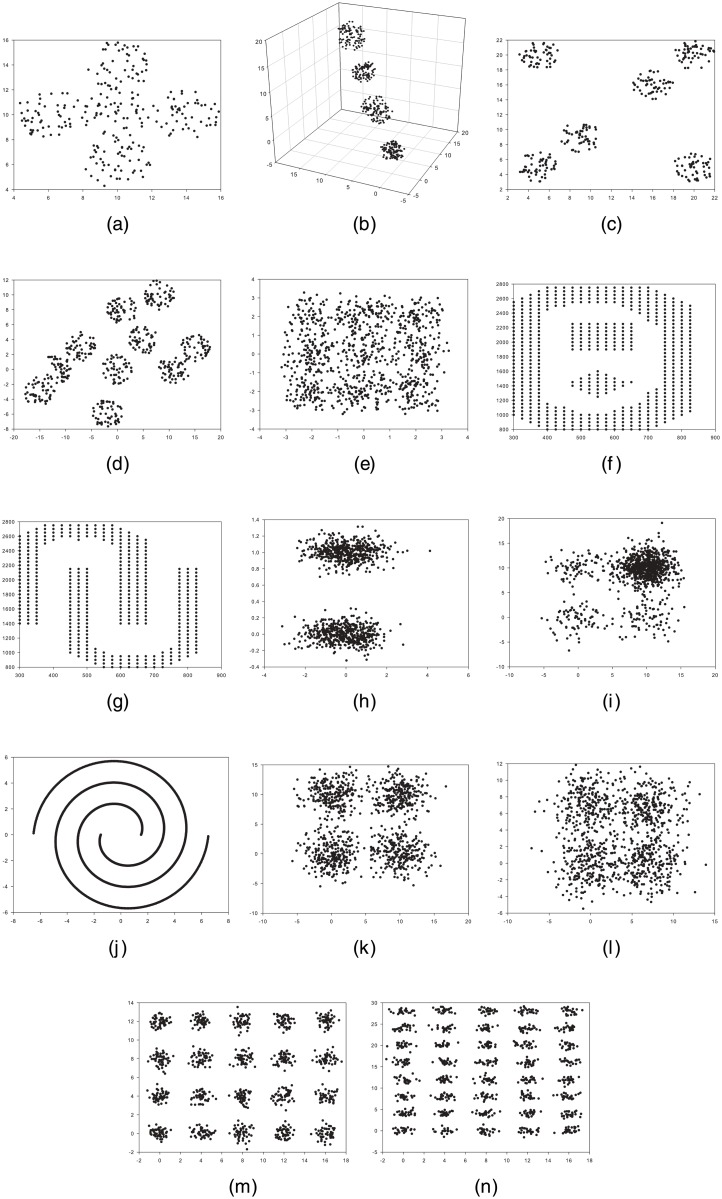
Graphs of the artificial datasets. (a) Sph_5_2. (b) Sph_4_3. (c) Sph_6_2. (d) Sph_10_2. (e) Sph_9_2. (f) Pat1. (g) Pat2. (h) Long1. (i) Sizes5. (j) Spiral. (k) Square1. (l) Square4. (m) Twenty. (n) Fourty.

- **Artificial datasets**
  1. Sph_5_2 [2] dataset (Appendix A in S1 File): This dataset consists of 250-point 2D distributed over five overlapping spherically shaped clusters. Each cluster contains 50 points. Fig 4a illustrates this dataset.
  2. Sph_4_3 [2] dataset (Appendix B in S1 File): This dataset demonstrated in Fig 4b comprises 400-point 3D distributed over four disjointed hyper spherically shaped clusters. Each cluster contains 100 points.
  3. Sph_6_2 [2] dataset (Appendix C in S1 File): This dataset involves 300-point 2D distributed over six different clusters. Each cluster embodies 50 points. This dataset is depicted in Fig 4c.
  4. Sph_10_2 [30] dataset (Appendix D in S1 File): This dataset accommodates 500-point 2D distributed over 10 different clusters, of which some are overlapping. Each cluster holds 50 points. This dataset is shown in Fig 4d.
  5. Sph_9_2 [30] dataset (Appendix E in S1 File): Specified in Fig 4e, this dataset embodies 900-point 2D distributed over nine highly overlapping clusters, in which each cluster incorporates 100 points.
  6. Pat1 [31] dataset (Appendix F in S1 File): This dataset involves 557-point 2D distributed over three different clusters; one of these clusters is non-convex. This dataset is signified in Fig 4f.
  7. Pat2 [31] dataset (Appendix G in S1 File): This dataset contains 417-point 2D distributed over two nonlinear, non-symmetric, and non-overlapping clusters. Fig 4g shows this dataset.
  8. Long1 [3] dataset (Appendix H in S1 File): This dataset shown in Fig 4h encloses 1000-point 2D distributed over two long-shaped clusters.
  9. Sizes5 [3] dataset (Appendix I in S1 File): This dataset comprises 1000-point 2D distributed over four square-shaped clusters, one of which contains more points than the others. Fig 4i displays this dataset.
  10. Spiral [3] dataset (Appendix J in S1 File): This dataset exhibited in Fig 4j consists of 1000-point 2D distributed over two spiral-shaped clusters.
  11. Square1 [3] dataset (Appendix K in S1 File): This dataset includes 1000-point 2D distributed over four semi-overlapping square-shaped clusters. Each cluster contains 250 points. This dataset is shown in Fig 4k.
  12. Square4 [3] dataset (Appendix L in S1 File): Specified in Fig 4l, this dataset comprises 1000-point 2D distributed over four overlapping square-shaped clusters, each containing 250 points.
  13. Twenty [3] dataset (Appendix M in S1 File): This dataset incorporates 1000-point 2D distributed over 20 small clusters. Each cluster contains 50 points. This dataset is shown in Fig 4m.
  14. Fourty [3] dataset (Appendix N in S1 File): This dataset exhibited in Fig 4n consists of 1000-point 2D distributed over 40 small clusters. Each cluster contains 25 points.
- **Real-life datasets**
  1. Iris [32] dataset (Appendix O in S1 File): This dataset comprises 150 four-feature samples distributed over three clusters each containing 50 observations. These samples are obtained from different categories of the iris flower (i.e., Setosa, Versicolor, and Virginica). Each sample has four feature values: sepal length, sepal width, petal length, and petal width. Two clusters of the iris flower (Versicolor and Virginica) are highly overlapping.
  2. Cancer [32] dataset (Appendix P in S1 File): This dataset consists of 683 samples with nine laboratory tests distributed over two clusters. Procured from Wisconsin Breast Cancer, these samples consist of two categories, malignant and benign, which are known to be linearly separable.
  3. Newthyroid [32] dataset (Appendix Q in S1 File): This dataset incorporates 215 instances with five laboratory tests distributed over three clusters. These samples are labeled as “Thyroid gland data,” which embody three categories (i.e., normal, hypo, and hyper).
  4. LiverDisorder [32] dataset (Appendix R in S1 File): This dataset represents 345 instances with six laboratory tests distributed over two clusters. The task is to determine whether a person suffers from alcoholism.
  5. Glass [32] dataset (Appendix S in S1 File): This dataset involves 214 samples with nine features distributed over six clusters. The field of criminological investigations has motivated the study on classifying the types of glass. At the scene of the crime, a glass left can provide evidence if it is correctly identified. In this dataset, the 10th feature (ID number) has been removed.

### Evaluating the clustering quality

An external criterion of the clustering quality for evaluating the results is presented in this section. The F-measure [33] is selected to compute the final solution obtained from the MOPSOSA, GenClustMOO, GenClustPESA2, MOCK, VGAPS, KM, and SL clustering algorithms. Let *T* and *C* be the two clustering solutions, $T=\{T_{1},\ldots ,T_{k_{T}}\}$ be the truth solution, and $C=\{C_{1},\ldots ,C_{k_{C}}\}$ be the solution to be measured, where *k*_*T*_ and *K*_*C*_ are number of clusters for the solutions *T* and *C* respectively. The F-measure of classes *T*_*i*_ and cluster *C*_*j*_ are defined as follows:

$$F(T_{i},C_{j})=\frac{2*P(T_{i},C_{j})*R(T_{i},C_{j})}{P(T_{i},C_{j})+R(T_{i},C_{j})}$$ (19)

where *P*(*T*_*i*_,*C*_*j*_) = *n*_*ij*_/|*C*_*j*_| and *R*(*T*_*i*_,*C*_*j*_) = *n*_*ij*_/|*T*_*i*_| Meanwhile, the F-measure of solutions *T* and *C* are construed below:

$$F(T,C)=\sum_{i=1}^{k_{T}}\frac{\left|T_{i}\right|}{n}\underset{c_{j}\in C}{max}\{F(T_{i},C_{j})\}$$ (20)

where *n* is the number of the dataset. Higher values of *F*(*T*,*C*) are better values, and the optimal value of *F*(*T*,*C*) is 1.

### Parameter settings

Table 2 presents the parameter settings employed in the proposed MOPSOSA algorithm. The performance of this algorithm is compared with three multi-objective automatic and three single-objective clustering algorithms (i.e., GenClustMOO, GenClustPESA2, MOCK, VGAPS, KM, and SL). These algorithms and the proposed algorithm are executed on all the above mentioned datasets. Employing semi-supervised method [20], the GenClustMOO and GenClustPESA2 algorithms select the best solutions from the final Pareto set. Additional details on the standard parameters employed in these algorithms can be acquired in Saha and Bandyopadhyay [8]. In the MOCK algorithm, GAP statistics [34] is used to select the best solution. The source code of the standard parameters used in MOCK is available in [3]. VGAPS, KM, and SL clustering algorithms provide a single solution. In VGAPS, population size is equal to 100, the number of generation is equivalent to 60, and mutation and crossover probabilities are computed adaptively. The total computations implemented in the proposed algorithm, GenClustMOO, GenClustPESA2, MOCK, and VGAPS, as well as the number of iterations in KM and SL, are all equal. Each algorithm is implemented 30 times.

**Table 2:** Parameter settings used in MOPSOSA algorithm.

Description	Parameters	Value
Swarm size	*k*	50
Number of iteration	*Iter*	100
Probability value to generate *W*	*w*	0.95
Probability value to generate *R*_1_	*r*_1_	0.90
Probability value to generate *R*_2_	*r*_2_	0.90
Minimum number of clusters	*K*_min_	2
Maximum number of clusters	*K*_max_	$\sqrt{n}+1$
Initial temperature	*T*_0_	100

## Results and Discussions

For each algorithm, the average value of F-measure is calculated for the final best solution to compare and exhibit the performance of the proposed algorithm with that of other algorithms. More information about the results of the cluster number and F-measure values of GenClustMOO, GenClustPESA2, MOCK, VGAPS, KM, and SL on the specified datasets can be acquired from Saha and Bandyopadhyay [8]. Table 3 displays the best value of F-measure and the number of clusters for the datasets automatically obtained with MOPSOSA, GenClustMOO, GenClustPESA2, MOCK, and VGAPS automatic clustering techniques. KM and SL are implemented with the actual number of clusters on all datasets.

**Table 3:** F-measure value and the number of clusters for different datasets obtained by MOPSOSA compared with those acquired by GenClustMOO, GenClustPESA2, MOCK, and VGAPS algorithms.

		MOPSOSA		GenClustMOO [8]		GenClustPESA2 [8]		MOCK [3]		VGAPS [4]	
Dataset	# Clusters	k	FM	k	FM	k	FM	k	FM	k	FM
Sph_5_2	5	5	0.98	5	0.97	5	0.94	6	0.91	5	0.55
Sph_4_3	4	4	1.00	4	1.00	4	1.00	4	1.00	4	1.00
Sph_6_2	6	6	1.00	6	1.00	6	1.00	6	1.00	6	1.00
Sph_10_2	10	10	0.99	10	0.99	12	0.94	6	0.72	7	0.76
Sph_9_2	9	9	0.92	9	0.69	8	0.66	9	0.73	9	0.49
Pat1	3	3	1.00	3	0.95	3	0.95	10	0.55	4	0.42
Pat2	2	2	1.00	2	1.00	2	1.00	11	0.55	4	0.59
Long1	2	2	1.00	2	1.00	2	1.00	2	1.00	3	0.50
Sizes5	4	4	0.98	4	0.97	3	0.88	2	0.80	5	0.82
Spiral	2	2	1.00	2	1.00	2	1.00	3	0.95	6	0.38
Square1	4	4	0.99	4	0.99	4	0.99	4	0.99	4	0.99
Square4	4	4	0.94	4	0.92	4	0.88	4	0.90	2	0.93
Twenty	20	20	1.00	20	1.00	24	0.95	20	1.00	20	0.48
Fourty	40	40	1.00	40	1.00	40	0.98	40	1.00	2	0.10
Iris	3	3	0.92	3	0.79	3	0.93	2	0.78	3	0.76
Cancer	2	2	0.98	2	0.97	2	0.98	2	0.82	2	0.95
Newthyroid	3	3	0.89	3	0.86	9	0.69	2	0.74	5	0.66
Liver Disorder	2	2	0.69	2	0.67	5	0.60	2	0.67	2	0.70
Glass	6	6	0.57	6	0.49	5	0.53	5	0.53	5	0.53

### Discussion of the artificial datasets results

**Fig 5:**
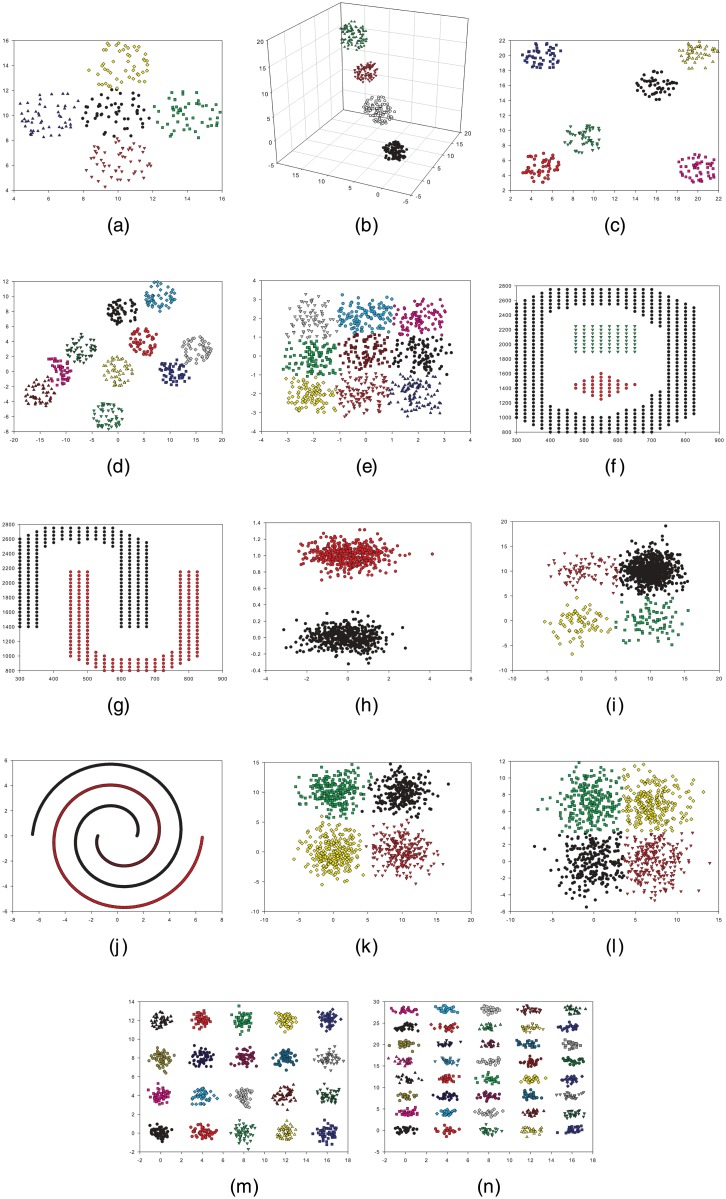
Graphs of the artificial datasets after applying the MOPSOSA algorithm. (a) Sph_5_2. (b) Sph_4_3. (c) Sph_6_2. (d) Sph_10_2. (e) Sph_9_2. (f) Pat1. (g) Pat2. (h) Long1. (i) Sizes5. (j) Spiral. (k) Square1. (l) Square4. (m) Twenty. (n) Fourty.

1. Sph_5_2: Table 4 displays that the maximum F-measure value for this dataset was obtained with the MOPSOSA algorithm even though existence five overlapping spherical clusters. However, MOPSOSA, GenClustMOO, GenClustPESA2, and VGAPS established the actual number of clusters as illustrated in Table 3. Fig 5a shows the clustering of this dataset after the MOPSOSA algorithm was applied.
2. Sph_4_3: The actual number for this dataset was detected with the MOPSOSA, GenClustMOO, GenClustPESA2, MOCK, and VGAPS clustering algorithms. All seven algorithms also achieved an F-measure value of 1, providing 100% accuracy for the clustering of this dataset (refer to Tables 3 and 4). Fig 5b exhibits the graph of clusters Sph_4_3 after the MOPSOSA algorithm was employed.
3. Sph_6_2: The F-measure value for this dataset was determined to be 1 for the seven algorithms (Table 4), signifying the accurate performance of all algorithms. Moreover, all algorithms attained the real number of clusters as demonstrated in Table 3. Fig 5c depicts the graph of the clusters for this dataset with the application of the MOPSOSA algorithm.
4. Sph_10_2: Table 3 reveals that only the MOPSOSA and GenClustMOO clustering algorithms achieved the desired number of clusters for this dataset. However, a maximum F-measure value was obtained with MOPSOSA (refer to Table 4) despite some overlap in these datasets. Fig 5d shows the graph for the clustering of Sph_10_2 with the post-application of the MOPSOSA algorithm.
5. Sph_9_2: For this dataset, Table 3 shows that MOPSOSA, GenClustMOO, MOCK, and VGAPS, except GenClustPESA2, were identified to be highly efficient in detecting the actual number of clusters. Despite the existence overlaps in all clusters for this dataset, MOPSOSA obtained a maximum F-measure value, demonstrating the accuracy of its performance (refer to Table 4). Fig 5e illustrates the dataset clustering with the MOPSOSA algorithm.
6. Pat1: Table 4 demonstrates that only MOPSOSA achieved the maximum F-measure value for this dataset, indicating a high accuracy clustering for well-separated clusters and for clusters of various shapes. Nevertheless, MOPSOSA, GenClustMOO, and GenClustPESA2 clustering algorithms attained the real number of clusters (Table 3), whereas MOCK was observed inappropriate for this dataset. The three clusters are clearly depicted in Fig 5f after the algorithm was applied on this dataset.
7. Pat2: Tables 3 and 4 show that the MOPSOSA, GenClustMOO, and GenClustPESA2 clustering algorithms obtained the real number of clusters for this dataset with the F-measure value as 1, signifying the high clustering accuracy of these algorithms in clustering these nonlinear and non-spherically dataset. Fig 5g reveals the graph of the two clusters in Pat2 with the application of the MOPSOSA algorithm.
8. Long1: For this dataset, MOPSOSA, GenClustMOO, GenClustPESA2, MOCK, and SL acquired the F-measure value of 1. Meanwhile, MOPSOSA, GenClustMOO, GenClustPESA2, and MOCK automatically resolved the proper cluster numbers for this dataset (refer to Tables 3 and 4). Fig 5h presents the clustering of this dataset into two correct clusters with the application of the MOPSOSA algorithm.
9. Sizes5: Table 4 reveals the maximum F-measure value obtained with the MOPSOSA algorithm for this dataset, which indicates that the proposed algorithm is qualified to clustering a dataset with different sizes of clusters. Regardless, Table 3 specifies that both MOPSOSA and GenClustMOO identified the actual number of clusters. Fig 5i shows the result of clustering on this dataset with the application of the MOPSOSA algorithm.
10. Spiral: Table 4 indicates that an F-measure value of 1 was acquired by MOPSOSA, GenClustMOO, and GenClustPESA2 for this dataset, indicating 100% accurate clustering on the spiral shapes. MOPSOSA, GenClustMOO, and GenClustPESA2 clustering algorithms also determined the real number of clusters as shown in Table 3. Fig 5j is a clear graphic illustration of the two spirals for this dataset with the application of the MOPSOSA algorithm.
11. Square1: For this dataset, all five automatic clustering algorithms (MOPSOSA, GenClustMOO, GenClustPESA2, MOCK, and VGAPS) detected the appropriate number of clusters (refer to Tables 3 and 4) and obtained the maximum F-measure value, thereby indicating their high accuracy in clustering this dataset. Fig 5k illustrates the result of clustering Square1 into four clusters by applying the MOPSOSA algorithm.
12. Square4: Table 3 exhibits that, for this dataset, MOPSOSA, GenClustMOO, GenClustPESA2, and MOCK, except VGAPS, established the actual number of clusters, with the maximum F-measure value obtained via MOPSOSA (see Table 4). The proposed algorithm was capable to clustering this data with high accuracy even though there are four overlapping clusters. The graph for the clustering of this dataset using the MOPSOSA algorithm is depicted in Fig 5l.
13. Twenty: For this dataset, MOPSOSA, GenClustMOO, MOCK, and VGAPS determined the real number of clusters (see Table 3), except GenClustPESA2. However, MOPSOSA, GenClustMOO, and MOCK obtained an F-measure value of 1, demonstrating an extremely high clustering accuracy even for several clusters (refer to Table 4). The clusters for this dataset after the application of MOPSOSA algorithm is graphically shown in Fig 5m.
14. Fourty: Table 3 reveals that for this dataset, only three automatic clustering algorithms (MOPSOSA, GenClustMOO, and MOCK) identified the desired cluster number. All these algorithms also obtained the F-measure value of 1, demonstrating an exceedingly high clustering accuracy despite the large number of clusters (refer to Table 4). Fig 5n depicts the graph for clustering this dataset after the application of the MOPSOSA algorithm.

**Table 4:** Averages and standard deviations for the F-measure values on the different datasets obtained from MOPSOSA, GenClustMOO, GenClustPESA2, MOCK, VGAPS, KM, and SL algorithms.

	F-measure that obtained from						
Dataset	MOPSOSA	GenClustMOO [8]	GenClustPESA2 [8]	MOCK [3]	VGAPS [4]	KM [14]	SL [15]
Sph_5_2	**0.982 ± 0.006**	0.957 ± 0.021	0.936 ± 0.012	0.902 ± 0.011	0.541 ± 0.011	0.938 ± 0.015	0.661 ± 0.012
Sph_4_3	**1.000 ± 0.000**	**1.000 ± 0.000**	**1.000 ± 0.000**	**1.000 ± 0.000**	**1.000 ± 0.000**	**1.000 ± 0.000**	**1.000 ± 0.000**
Sph_6_2	**1.000 ± 0.000**	**1.000 ± 0.000**	**1.000 ± 0.000**	**1.000 ± 0.000**	**1.000 ± 0.000**	**1.000 ± 0.000**	**1.000 ± 0.000**
Sph_10_2	**0.991 ± 0.002**	0.981 ± 0.011	0.931 ± 0.021	0.717 ± 0.013	0.752 ± 0.011	0.891 ± 0.014	0.841 ± 0.011
Sph_9_2	**0.921 ± 0.001**	0.681 ± 0.012	0.652 ± 0.018	0.717 ± 0.009	0.481 ± 0.012	0.683 ± 0.013	0.250 ± 0.014
Pat1	**0.989 ± 0.012**	0.946 ± 0.013	0.946 ± 0.009	0.547 ± 0.011	0.418 ± 0.014	0.618 ± 0.008	0.882 ± 0.011
Pat2	**1.000 ± 0.000**	**1.000 ± 0.000**	**1.000 ± 0.000**	0.545 ± 0.013	0.582 ± 0.021	0.754 ± 0.013	**1.000 ± 0.000**
Long1	**1.000 ± 0.000**	**1.000 ± 0.000**	**1.000 ± 0.000**	**1.000 ± 0.000**	0.487 ± 0.021	0.500 ± 0.011	**1.000 ± 0.000**
Sizes5	**0.977 ± 0.001**	0.968 ± 0.001	0.883 ± 0.011	0.791 ± 0.012	0.816 ± 0.013	0.226 ± 0.021	0.181 ± 0.011
Spiral	**1.000 ± 0.000**	**1.000 ± 0.000**	**1.000 ± 0.000**	0.948 ± 0.011	0.373 ± 0.016	0.509 ± 0.011	0.504 ± 0.015
Square1	**0.999 ± 0.011**	**0.999 ± 0.013**	**0.999 ± 0.014**	**0.999 ± 0.012**	**0.999 ± 0.014**	0.732 ± 0.021	0.368 ± 0.006
Square4	**0.935 ± 0.001**	0.918 ± 0.014	0.878 ± 0.011	0.895 ± 0.011	0.925 ± 0.013	0.715 ± 0.015	0.368 ± 0.016
Twenty	**1.000 ± 0.000**	**1.000 ± 0.000**	0.948 ± 0.015	**1.000 ± 0.000**	0.479 ± 0.022	0.809 ± 0.003	0.947 ± 0.009
Fourty	**1.000 ± 0.000**	**1.000 ± 0.000**	0.979 ± 0.015	**1.000 ± 0.000**	0.950 ± 0.006	0.798 ± 0.018	0.909 ± 0.023
Iris	**0.937 ± 0.011**	0.788 ± 0.011	0.926 ± 0.015	0.775 ± 0.022	0.754 ± 0.013	0.887 ± 0.001	0.764 ± 0.009
Cancer	**0.981 ± 0.003**	0.969 ± 0.009	0.979 ± 0.014	0.918 ± 0.014	0.953 ± 0.012	0.961 ± 0.013	0.688 ± 0.008
Newthyroid	**0.885 ± 0.010**	0.863 ± 0.016	0.687 ± 0.015	0.739 ± 0.014	0.659 ± 0.011	0.677 ± 0.013	0.648 ± 0.009
Liver Disorder	**0.770 ± 0.010**	0.673 ± 0.002	0.603 ± 0.015	0.671 ± 0.012	0.705 ± 0.009	0.655 ± 0.013	0.672 ± 0.006
Glass	**0.568 ± 0.002**	0.494 ± 0.012	0.534 ± 0.012	0.534 ± 0.006	0.534 ± 0.008	0.492 ± 0.014	0.422 ± 0.007

The best F-measure for each dataset is marked in bold. Each algorithm is implemented on 30 independent runs.

### Discussion of the real-life datasets results

1. Iris: Table 4 shows that for this dataset, the maximum F-measure value was obtained with the proposed algorithm MOPSOSA. However, with the exception of MOCK, all four automatic clustering algorithms (MOPSOSA, GenClustMOO, GenClustPESA2, and VGAPS) resolved the proper number of clusters, as evidenced in Table 3.
2. Cancer: The maximum F-measure value for this dataset was obtained with the proposed MOPSOSA algorithm (see Table 4). Nevertheless, all five automatic clustering algorithms (MOPSOSA, GenClustMOO, GenClustPESA2, MOCK, and VGAPS) identified the correct number of clusters, as illustrated in Table 3.
3. Newthyroid: Table 4 reveals that the maximum F-measure value for this dataset was attained with the MOPSOSA algorithm. However, Table 3 specifies that only two automatic clustering algorithms (MOPSOSA and GenClustMOO) determined the actual number of clusters.
4. Liver Disorder: For this dataset, MOPSOSA, GenClustMOO, MOCK, and VGAPS, except GenClustPESA2, identified the actual number of clusters (refer to Table 3). Meanwhile, the maximum F-measure was achieved with the proposed algorithm MOPSOSA (refer to Table 4).
5. Glass: Table 4 demonstrates that the maximum F-measure value for this dataset was obtained with the MOPSOSA algorithm. Only MOPSOSA and GenClustMOO automatic clustering algorithms were determined to be capable of achieving the desired number of clusters (see Table 3).

### Summary of results

The above results signify that the proposed MOPSOSA algorithm achieves accurate results in all datasets. Moreover, the proposed algorithm can automatically establish the correct cluster numbers for all datasets used in the experiment. The algorithm is also proven capable of dealing with various shapes of datasets (hyper spheres, linear, and spiral), overlapping datasets, datasets that have well-separated clusters with any convex and non-convex shapes, and datasets that contain several clusters. With most datasets having dimensions from 2 to 9, objects from 150 to 1000, and number of clusters from 2 to 40, the MOPSOSA algorithm displays superiority over the three multi-objective automatic and three single-objective clustering algorithms. The results also show that the GenClustMOO algorithm can automatically identify the actual cluster numbers, but with a lower quality of clustering accuracy than the proposed algorithm. In general, MOCK can detect the number of clusters for hyper spheres and linear, but it is unsuccessful for non-convex well-separated and overlapping clusters. The results also prove that the VGAPS algorithm is not suitable for non-convex well-separated clusters or for datasets with numerous clusters.

The main factors that led to the accuracy of the proposed algorithm in solving the clustering problem are attributed to the power and speed of the search characterized by the particle swarm, with the guarantee of not becoming stagnant into local solutions via the MOSA algorithm. The development of particle swarm to address more than one validity index can cluster any dataset. The generation of the initial swarm of particles can be improved with KM method. Meanwhile, the repository for preserving the diversity of clustering solutions can be updated by adopting the sharing fitness, and the redundant particles can be eliminated with the re-numbering process.

## Conclusion

This research proposed a new automatic multi-objective clustering algorithm MOPSOSA based on a hybrid multi-objective particle swarm algorithm and multi-objective simulated annealing. A multi-objective particle swarm optimization was also developed from a combinatorial particle swarm optimization. The proposed algorithm was proven capable of automatically clustering the dataset into the appropriate number of clusters. With the simultaneous optimization of three objective functions, the Pareto optimal set was obtained from the proposed algorithm. The first objective function considered the compactness of the clustering based on Euclidean distance. The second function regarded the total symmetry of the clusters, and the third considered the connectedness of the clusters. The proposed algorithm was performed on 19 real-life and artificial datasets, and its performance was compared with that of three multi-objective automatic and three single-objective clustering techniques. MOPSOSA obtained better accuracy in its results compared to that of other algorithms. The results also demonstrated that the proposed algorithm can be used for datasets of various shapes and for overlapping and non-convex datasets.

## Supporting Information

S1 File Experimental datasets. 250 points of the artificial datasets Sph_5_2 (Appendix A). 400 points of the artificial datasets Sph_4_3 (Appendix B). 300 points of the artificial datasets Sph_6_2 (Appendix C). 500 points of the artificial datasets Sph_10_2 (Appendix D). 900 points of the artificial datasets Sph_9_2 (Appendix E). 557 points of the artificial datasets Pat1 (Appendix F). 417 points of the artificial datasets Pat2 (Appendix G). 1000 points of the artificial datasets Long1 (Appendix H). 1000 points of the artificial datasets Sizes5 (Appendix I). 1000 points of the artificial datasets Spiral (Appendix J). 1000 points of the artificial datasets Square1 (Appendix K). 1000 points of the artificial datasets Square4 (Appendix L). 1000 points of the artificial datasets Twenty (Appendix M). 1000 points of the artificial datasets Fourty (Appendix N). 150 samples of the real-life datasets Iris (Appendix O). 683 samples of the real-life datasets Cancer (Appendix P). 215 instances of the real-life datasets Newthyroid (Appendix Q). 345 instances of the real-life datasets LiverDisorder (Appendix R). 214 samples of the real-life datasets Glass (Appendix S).
(PDF)
