# Supplementary material for: Automatic Clustering Using Multi-objective Particle Swarm and Simulated Annealing
Source: PLoS One. 2015 Jul 1;10(7):e0130995. doi: 10.1371/journal.pone.0130995 (PMC4488466; doi:10.1371/journal.pone.0130995)
Supplement: S1 File — 250 points of the artificial datasets Sph_5_2 (Appendix A). 400 points of the artificial datasets Sph_4_3 (Appendix B). 300 points of the artificial datasets Sph_6_2 (Appendix C). 500 points of the artificial datasets Sph_10_2 (Appendix D). 900 points of the artificial datasets Sph_9_2 (Appendix E). 557 points of the artificial datasets Pat1 (Appendix F). 417 points of the artificial datasets Pat2 (Appendix G). 1000 points of the artificial datasets Long1 (Appendix H). 1000 points of the artificial datasets Sizes5 (Appendix I). 1000 points of the artificial datasets Spiral (Appendix J). 1000 points of the artificial datasets Square1 (Appendix K). 1000 points of the artificial datasets Square4 (Appendix L). 1000 points of the artificial datasets Twenty (Appendix M). 1000 points of the artificial datasets Fourty (Appendix N). 150 samples of the real-life datasets Iris (Appendix O). 683 samples of the real-life datasets Cancer (Appendix P). 215 instances of the real-life datasets Newthyroid (Appendix Q). 345 instances of the real-life datasets LiverDisorder (Appendix R). 214 samples of the real-life datasets Glass (Appendix S). (PDF) [file pone.0130995.s001.pdf]

Appendix A

Sph_5_2
Number of dimensions: 2
Number of clusters: 5
Number of points: 250

Cluster #	x-coordinates	y-coordinates
1	11.11	10.14
1	8.68	9.76
1	10.04	10.63
1	8.79	10.13
1	10.53	8.95
1	10.58	9.72
1	11.19	11.6
1	8.14	10.21
1	9.62	10.83
1	11.31	9.7
1	9.95	8.16
1	9.84	10.93
1	10.05	10.35
1	9.41	11.39
1	10.91	11.33
1	10.13	11.76
1	8.51	11.3
1	10.2	10.89
1	9.94	10.1
1	8.25	9.49
1	8.93	10.87
1	8.16	10.69
1	10.74	8.36
1	11.79	9.77
1	9.33	10.64
1	10.86	11.3
1	9.04	11.66
1	8.19	9.17
1	9.06	10.1
1	11.49	10.43
1	9.03	10.04
1	10.84	10.82
1	11.09	10.28
1	11.11	10.39
1	9.77	8.33
1	8.24	10.66
1	9.88	9.48
1	8.87	10.72
1	11.42	9.34

Cluster #	x-coordinates	y-coordinates
1	9.2	8.49
1	9.46	9.88
1	10.86	11.78
1	9.88	10.23
1	10.37	9.72
1	9.26	8.63
1	11.42	9.06
1	8.96	10.78
1	10.38	8.92
1	8.78	9.96
1	11.19	10.71
2	11.81	6.15
2	10.4	6.66
2	9.15	5.55
2	11.43	7.07
2	8.61	6.62
2	10.6	7.27
2	9.49	5.97
2	10.99	5.51
2	8.45	6.57
2	10.69	7.42
2	10.14	4.86
2	11.52	7.39
2	10.04	6.87
2	11.7	5.99
2	10.26	6.71
2	10.06	5.87
2	10.62	4.81
2	11.25	7.51
2	8.17	5.62
2	8.83	7.48
2	10.15	6.99
2	8.76	7
2	10.27	7.4
2	10.91	5.35
2	9.87	5.55
2	11.48	5
2	10.05	7.96
2	10.73	6.3
2	11.89	5.97
2	9.03	6.44
2	10.27	7.35
2	8.86	4.74
2	9.23	5.94
2	11.24	6.37

Cluster #	x-coordinates	y-coordinates
2	10.22	5.51
2	8.68	7.15
2	8.48	5.08
2	9.97	4.86
2	8.79	6.09
2	10.8	7.26
2	9.87	5.59
2	9.62	5.06
2	8.63	7.52
2	9.24	7.04
2	8.88	7.35
2	10.91	7.76
2	9.87	7.84
2	9.16	6.18
2	9.25	4.29
2	11.37	5.5
3	13.74	11.7
3	14.63	11.17
3	15.44	10.46
3	13.28	11.27
3	13.15	10.56
3	13.16	10.65
3	13.23	8.76
3	14.46	8.7
3	13.21	10.44
3	13.45	10.2
3	12.34	10.33
3	14.32	9.79
3	15.39	9.18
3	13.44	9.48
3	15.09	10.79
3	12.85	9.3
3	14.13	8.91
3	13.19	9.97
3	13.8	8.84
3	14.08	10.93
3	14.53	11.83
3	13.61	10.37
3	15.69	9.18
3	13.95	9.73
3	13.99	9.22
3	15.71	9.4
3	13.77	10.07
3	14.85	9.43
3	12.09	10.71

Cluster #	x-coordinates	y-coordinates
3	15.88	10.25
3	13.28	11.25
3	13.1	11.83
3	15.65	10.86
3	12.94	11.38
3	13.59	10.51
3	14.52	10.94
3	13.88	10.65
3	14.88	10.75
3	15.73	9.8
3	12.27	9.45
3	13.37	10.67
3	13.51	10.42
3	13.74	11.9
3	14.68	10.91
3	12.56	9.02
3	13.09	8.48
3	15.18	9.13
3	15.31	9.71
3	13.01	8.33
3	14.64	8.21
4	11.12	13.31
4	11.43	13.45
4	11.2	15.49
4	10.93	13.91
4	11.19	13.24
4	10.67	14.77
4	9.94	12.17
4	9.46	12.35
4	9.31	15.25
4	10.31	15.26
4	10.58	15.4
4	11.53	12.71
4	10.88	13.61
4	9.08	12.46
4	9.64	12.05
4	10.42	15.57
4	11.09	15.2
4	9.56	13.41
4	10.94	15.5
4	9.9	15.62
4	10.09	15.15
4	9.39	15.76
4	10.2	12.66
4	10.44	13.58

Cluster #	x-coordinates	y-coordinates
4	9.08	13.97
4	9.56	12.32
4	11.58	14.9
4	9.17	15.25
4	11.69	14.05
4	9.24	13.7
4	11.67	14.36
4	10.58	14.32
4	10.56	14.87
4	9.47	15.62
4	8.32	13.62
4	10.03	15.79
4	9.41	13.6
4	11.67	13.58
4	9.29	12.55
4	9.93	12.91
4	10.88	12.93
4	9.73	14.31
4	9.92	12.07
4	8.84	13.29
4	11.48	14.49
4	10.65	13.06
4	11.53	14.4
4	11.23	12.72
4	8.89	12.49
4	9.16	14.12
5	7.21	9.66
5	4.79	10.99
5	5.38	8.74
5	7.96	9.76
5	7.1	11.72
5	5.49	11.63
5	6.82	11.73
5	7.26	9.27
5	5.69	8.95
5	4.47	9.53
5	5.06	8.59
5	5.52	11.29
5	4.69	9.5
5	6.02	9.05
5	4.36	10.93
5	6.96	9.41
5	6.61	9.98
5	7.27	9.04
5	6.17	11.69

Cluster #	x-coordinates	y-coordinates
5	6.14	8.38
5	6.61	9.06
5	4.98	8.77
5	5.03	9.42
5	4.65	9.12
5	5.08	9.74
5	4.95	8.49
5	7.48	10.64
5	6.54	10.76
5	5.6	9.53
5	6.78	8.98
5	5.11	8.75
5	4.6	9.7
5	4.82	9.54
5	4.8	10.34
5	5.54	9.4
5	5.26	10.29
5	5.48	10.88
5	6.92	10.55
5	6.55	10.25
5	5.41	10.15
5	4.45	10.14
5	5	8.43
5	6.62	8.4
5	7.7	11.15
5	5.45	9.99
5	7.01	9.11
5	6.95	10.75
5	6.7	9.87
5	5.71	8.26
5	5.79	10.67

Appendix B

Sph_4_3
Number of dimensions: 3
Number of clusters: 4
Number of points: 400

Cluster #	x-coordinates	y-coordinates	z-coordinates
1	-0.13	1.39	0.33
1	1.21	-0.18	0.65
1	1.14	-0.17	0.13
1	-0.51	1.29	1.15
1	1.08	0.33	1.35
1	0.95	-0.14	1.4
1	0.87	0.12	-1.05
1	0.73	0.49	-0.08
1	-0.12	-0.64	-1.11
1	0.4	-1.66	-1.02
1	-0.52	1.85	0.55
1	0.26	-1.26	1.16
1	-0.25	0.5	1.4
1	0.79	0.72	-0.09
1	0.08	1.81	-0.26
1	0.8	0.99	-0.84
1	0.23	-0.19	-0.22
1	0.38	0.82	-0.26
1	-1.43	0.4	-1.04
1	0.45	0.28	1.55
1	0.36	1.33	0.01
1	-1.59	0.75	0.64
1	0.98	-0.25	-0.58
1	0.22	-1.37	0.25
1	-0.84	0.67	-1.17
1	-0.17	0.76	-0.54
1	0.83	-0.95	-1.27
1	-0.28	-1.69	-0.87
1	-0.44	-0.12	0.92
1	1.02	0.12	0.58
1	1.44	-1.08	-0.64
1	0.71	1.32	-1
1	0.37	-0.47	1.09
1	-1.57	-0.31	0.91
1	-1.5	0.89	0.48
1	-0.35	-1.04	-0.54
1	0.93	0.05	0.23
1	1.18	-0.29	-1.48
1	-0.38	0.22	-1.52

Cluster #	x-coordinates	y-coordinates	z-coordinates
1	-0.82	-1.19	-1.16
1	-0.83	-1.36	-0.47
1	0.24	-0.53	-1.37
1	0.81	1.26	0.89
1	-0.74	0.6	1.75
1	-1.27	-0.39	0.42
1	1.55	0.91	0.81
1	0.59	1.83	-0.43
1	-1.05	-0.43	-0.89
1	-1.04	1.18	-1.14
1	1.37	-0.13	0.1
1	-0.23	-0.83	1.77
1	0.37	-0.98	0.05
1	-0.05	-0.16	1.44
1	0.62	-1.2	-0.13
1	-0.25	-0.61	-1.39
1	0.24	-0.72	-1.56
1	-1.58	-0.37	0.14
1	-0.2	1.72	0.43
1	0.7	-0.28	1.3
1	-0.93	0.11	1.27
1	1.1	1.08	-0.97
1	0.71	0.3	-0.22
1	1.21	0.62	0.46
1	0.47	-0.09	-1.69
1	-1.21	-0.45	-0.38
1	-1.07	0.76	0.61
1	1.29	-0.07	-1.11
1	0.87	-1.74	0.31
1	0.92	-1.3	-1.21
1	0.51	-0.75	-0.91
1	-0.19	-0.48	1.4
1	0.51	-0.21	1.01
1	0.84	-0.84	0.5
1	1.8	-0.4	0.16
1	-0.7	0.82	-1.57
1	-1.26	-0.92	-0.34
1	0.88	1.64	-0.67
1	-0.32	-0.41	1.72
1	-0.73	1.26	0.46
1	-1.24	-0.38	-0.18
1	1.92	0.55	-0.01
1	-1.65	-0.47	-0.24
1	1.15	0.63	0.71
1	0.51	0.04	-1.34

Cluster #	x-coordinates	y-coordinates	z-coordinates
1	0.76	0.48	-1.29
1	-1.75	-0.43	0.57
1	1.1	0.14	-0.83
1	0.4	0.19	1.62
1	-1.11	-0.12	-0.01
1	-0.02	0.48	-0.48
1	-0.28	-0.27	0.42
1	0.83	-0.44	-0.31
1	-1.22	1	-0.55
1	0.46	-0.12	-0.47
1	-0.21	0.32	0.52
1	0.5	-0.99	0.55
1	0.31	-0.71	-0.24
1	-0.15	0.35	0.01
1	0.05	-0.87	0.22
1	0.05	1.11	0.85
2	3.29	4.27	5.91
2	5.89	7.57	4.71
2	3.91	4.44	6.44
2	4.86	3.36	5.63
2	4.57	4.36	3.07
2	4.38	2.58	4.43
2	3.39	4.85	6.15
2	4.48	6.99	4.37
2	5.06	4.91	5.7
2	4.51	7.12	5.2
2	6.12	3.46	3.18
2	6.81	5.93	4.7
2	5.72	4.57	4.1
2	6.29	6.23	5.48
2	4.37	5.36	5.59
2	4.74	3.3	6.09
2	5.31	7.68	4.82
2	3.09	5.41	4.7
2	4.89	3.78	2.55
2	5.14	6.72	6.89
2	4.76	5.76	4.04
2	3.95	5.25	3.5
2	6.71	4.3	5.68
2	4.27	7.3	5.06
2	4.92	3.38	4.94
2	4.32	6.23	5.39
2	4.31	5.35	4.79
2	3.8	6.98	5.64
2	4.88	6.05	2.67

Cluster #	x-coordinates	y-coordinates	z-coordinates
2	3.96	5.09	5.73
2	5.44	6.63	5.57
2	5.69	3.04	3.44
2	4.16	3.51	5.23
2	3.46	3.72	4.98
2	4.94	5.23	2.45
2	4.7	6.02	3.87
2	3.96	5.25	7.33
2	5.1	4.13	2.85
2	6.25	2.8	4.54
2	4.83	6.25	5.78
2	4.43	7.02	6.94
2	5.48	3.29	5.6
2	5	6.78	4.57
2	3.44	4.36	4
2	6.08	4.82	7.23
2	4.68	4.72	4.11
2	4.98	7.74	5.66
2	3.42	5.58	6.31
2	5.86	6.14	3.74
2	4.06	5.32	6.87
2	5.96	4.99	3.85
2	3.89	4.89	5.34
2	4.61	5.34	6.27
2	3.18	5.46	5.99
2	4.83	4.62	2.22
2	5.65	3.62	3.79
2	4.13	7.11	5.22
2	3.84	3.91	3
2	3.19	4.41	5.19
2	3.82	5.32	2.97
2	5.2	7.57	6.09
2	6.58	4.8	6.33
2	3.87	6.71	6.46
2	5.45	7.31	4.67
2	4.56	6.25	7.62
2	5.27	6.9	4.47
2	3.28	5.06	4.7
2	5.87	5.49	2.78
2	5.36	4.91	5.1
2	4.24	4.14	5.16
2	5.31	3.57	3.52
2	5.4	6.93	4.74
2	5.18	3.93	4.82
2	5.17	7.16	4.45

Cluster #	x-coordinates	y-coordinates	z-coordinates
2	5.19	5.71	4.84
2	4.56	7.88	5.24
2	3.52	6.13	5.33
2	5.36	3.7	5.86
2	5.3	7.08	4.86
2	5.04	7.82	4.04
2	4.17	5.73	4
2	4.85	4.62	7.9
2	3.96	2.71	4.01
2	6.83	4.94	5.39
2	6	5.7	6.79
2	5.15	3.63	5.58
2	6.26	5.81	7.03
2	3.56	3.57	5.48
2	5.47	5.25	6.7
2	3.43	4.49	5.37
2	4.52	6.79	5.09
2	4.53	4.59	6.06
2	5.97	6.11	4.52
2	5.52	4.77	6.08
2	5.45	5.84	2.33
2	5.75	5.28	4.92
2	3.93	2.62	5.79
2	5.3	4.08	2.66
2	4.23	6.43	3.88
2	3.55	4.33	3.45
3	12.61	10.32	9.76
3	9.9	9.95	11.06
3	7.99	10.72	10.59
3	8.51	8.39	10.35
3	10.46	9.45	11.13
3	11.77	11.55	9.75
3	12.76	9.78	10.52
3	9.49	9.36	11.7
3	10.09	9.65	9.84
3	10.04	8.87	8.52
3	8.61	10.48	10.39
3	7.28	9.28	9.88
3	9.17	9.19	8.87
3	9.85	11.55	10.88
3	10.85	9.73	9.06
3	10.89	9.61	10.83
3	9.3	10.73	8.44
3	8.2	9.12	10.1
3	12.74	9.89	10.4

Cluster #	x-coordinates	y-coordinates	z-coordinates
3	9.98	10.34	10.41
3	8.66	11.03	8.91
3	8.14	11.22	10.35
3	10.8	11.86	10.35
3	11	10.75	10.35
3	8.73	8.35	9.86
3	11.14	9.61	10.71
3	9.98	11.47	10.78
3	10.07	9.42	11.03
3	8.64	8.93	10.01
3	8.54	9.61	11.66
3	7.56	8.97	10.28
3	9.48	9.05	8.36
3	9.26	11.25	11.16
3	8.74	10.33	9.28
3	11.11	10.05	10.62
3	9.31	9.86	9.55
3	9.61	10.54	9.38
3	10.85	10.57	9.68
3	8.19	8.57	10.1
3	11.39	9.72	9.12
3	10.2	9.41	8.84
3	9.97	8.19	10.21
3	10.24	8.15	9.43
3	10.69	9.05	11.01
3	11.66	9.8	10.52
3	8.71	11.59	10.58
3	8.74	9.97	8.95
3	8.67	10.71	8.95
3	11.69	9.09	11.26
3	10.66	9.29	10.98
3	8.04	10.19	10.62
3	10.71	11.17	10.77
3	11.7	9.98	10.79
3	8.83	10.94	9.08
3	8.42	10.77	9.9
3	9.35	10.56	10.58
3	7.48	10.23	10.61
3	11.55	10.74	8.84
3	10.83	8.92	8.6
3	12.58	10.41	10.26
3	11.61	9.5	10.74
3	8.68	9.74	11.54
3	8.94	11.06	9.26
3	9.42	8.94	11.28

Cluster #	x-coordinates	y-coordinates	z-coordinates
3	10.76	9.45	8.43
3	11.04	9.49	8.53
3	9.28	8.98	9.83
3	8.01	9.1	9.58
3	8.12	9.78	10.14
3	9.99	11.25	9.87
3	10.4	11.03	8.74
3	8.44	11.37	10.75
3	10.33	11.73	10.3
3	10.7	9.39	9.02
3	8.55	8.34	10.1
3	11.61	8.56	10.21
3	9.7	11.23	9.31
3	8.87	9.9	11.21
3	12.46	10.83	10.73
3	12.2	11.34	9.98
3	9.53	9.01	10.89
3	12.41	9.2	9.65
3	8.38	10.6	11.08
3	9.69	9.6	11.24
3	10.37	9.57	11.43
3	11.19	10.47	10.22
3	9.15	9.87	11
3	9.85	9.57	11.43
3	8.93	11.6	9.1
3	10.24	11.08	11.26
3	10.18	11.42	10.33
3	8.95	10.45	8.64
3	10.82	8.72	10.09
3	11.19	8.95	9.19
3	9.53	8.92	11.13
3	11.09	9.58	11.67
3	7.76	10.28	10.26
3	8.5	9.88	8.44
3	12.23	10.9	10.41
3	7.86	9.8	11.34
4	15.46	12.77	14.46
4	14.61	13.9	13.72
4	17.06	16.28	15.79
4	16.22	17.3	15.65
4	13.03	14.02	17
4	13.92	16.3	14.34
4	13.77	15.04	14.21
4	15.18	13.49	16.89
4	12.48	15.36	16.21

Cluster #	x-coordinates	y-coordinates	z-coordinates
4	15.02	13.03	17.24
4	16.2	16.42	14.12
4	13.99	13.98	13.44
4	14.44	16.42	16.68
4	17.66	15.76	16.08
4	16.51	15.52	15.08
4	16.37	14.15	15.08
4	14.12	15.65	13.72
4	14.53	12.85	15.74
4	14.52	15.21	17.28
4	14.97	15.38	16.69
4	16.07	12.74	14.39
4	16.35	13.5	13.79
4	16.49	17.02	16.06
4	13.82	16.45	15.96
4	12.94	16.26	14.02
4	13.62	14.53	14.67
4	16.92	14.55	15.52
4	15.93	13.58	14.82
4	12.45	15.9	15.1
4	12.82	14.51	16.85
4	14.17	13.34	16.42
4	15.59	14.55	16.71
4	14.56	13.12	14.39
4	14.6	12.62	16.5
4	14.93	14.95	15.37
4	14.44	16.94	16.4
4	16.92	14.34	12.97
4	14.77	13.14	13
4	12.42	14.61	14.91
4	14.58	16.44	17.08
4	15.14	13.08	17.28
4	15.59	15.91	13.89
4	17.38	16.4	15.66
4	16.21	15.88	15.78
4	14.14	13.9	15.23
4	14.49	15.75	12.98
4	14.93	15.73	14.77
4	14.85	12.82	16.56
4	15.36	17.73	16.07
4	15.4	12.52	14.12
4	15.92	14.37	16.06
4	15.56	17.33	16.02
4	14.84	17.29	13.94
4	13.97	16.04	14.47

Cluster #	x-coordinates	y-coordinates	z-coordinates
4	13.73	16.08	12.95
4	15.81	14.1	13.81
4	17.5	14.51	13.72
4	15.4	12.94	13.1
4	16.6	17.1	15.01
4	15.64	13.1	16.09
4	15.32	14.08	12.67
4	14.54	15.84	12.27
4	15.48	15.4	15.34
4	15.5	16.9	16.22
4	15.6	14.93	17.87
4	13.81	14.71	16.42
4	14.44	14.97	13.29
4	15.53	13.16	14.35
4	16.09	17.6	15.27
4	16.87	15.61	13.97
4	15.38	14.37	13.51
4	15.98	13.99	14.38
4	12.93	14.26	14.37
4	13.65	14.71	14.74
4	17.26	13.45	15.58
4	14.47	15.48	17.7
4	16.55	13.7	15.06
4	14.42	13.18	13.38
4	15.19	16.3	16.33
4	15.78	16.29	12.72
4	13.02	14.96	16.85
4	14.32	13.49	14.46
4	13.33	15.05	16.67
4	13.9	16.42	13.81
4	17.29	16.1	13.8
4	14.66	16.28	17.25
4	14.23	13.4	12.89
4	14.2	13.88	12.46
4	16.92	14.76	15.7
4	15.33	13.76	15.91
4	15.37	14.22	13.94
4	13.78	13.2	16.6
4	13.41	14.41	15.96
4	16.12	16.74	16.55
4	15.88	13.42	13.42
4	13.7	15.21	14.37
4	16.2	15.87	17.59
4	15.41	13.19	16.65
4	16.47	17.32	15.91

Cluster #	x-coordinates	y-coordinates	z-coordinates
4	16.56	16.75	15.16

Appendix C

Sph_6_2
Number of dimensions: 2
Number of clusters: 6
Number of points: 300

Cluster #	x-coordinates	y-coordinates
1	14.86	16.60
1	14.13	16.28
1	17.10	16.64
1	14.82	17.40
1	17.16	15.54
1	16.72	16.53
1	15.37	15.95
1	17.26	14.75
1	14.27	15.99
1	17.51	16.42
1	16.29	14.13
1	15.72	17.85
1	15.92	16.71
1	15.86	16.04
1	14.19	15.32
1	14.54	15.71
1	16.18	15.57
1	15.58	16.98
1	16.63	15.60
1	17.30	16.24
1	14.94	14.79
1	14.61	16.84
1	17.94	16.28
1	15.79	17.88
1	17.69	16.37
1	15.34	16.58
1	15.08	16.54
1	15.33	14.52
1	16.13	15.58
1	15.43	16.50
1	16.40	15.09
1	16.50	15.46
1	15.80	14.87
1	17.03	16.17
1	15.68	16.60
1	16.82	15.14
1	16.30	15.70
1	16.15	16.29
1	15.62	14.81

Cluster #	x-coordinates	y-coordinates
1	16.50	17.80
1	14.77	15.24
1	15.76	16.64
1	17.64	15.59
1	14.24	16.06
1	15.46	16.09
1	17.54	16.34
1	16.10	17.69
1	16.02	15.05
1	15.99	14.11
1	15.94	15.01
2	5.11	3.08
2	4.06	3.69
2	3.18	4.34
2	4.65	4.45
2	5.08	4.53
2	5.92	6.27
2	5.12	5.16
2	4.27	4.20
2	5.18	5.99
2	4.78	4.30
2	3.74	4.56
2	5.61	3.53
2	4.77	4.66
2	5.40	5.22
2	5.77	4.88
2	4.22	3.18
2	4.41	6.60
2	5.30	3.64
2	6.59	5.74
2	5.16	6.93
2	5.69	5.29
2	3.54	4.39
2	5.64	4.50
2	5.41	6.07
2	3.40	4.35
2	4.72	3.98
2	5.08	5.70
2	3.95	4.31
2	6.62	5.40
2	4.05	4.54
2	3.67	5.04
2	3.89	6.56
2	3.69	6.51
2	3.74	5.00

Cluster #	x-coordinates	y-coordinates
2	5.07	3.07
2	3.56	4.63
2	4.00	6.48
2	6.51	4.73
2	6.22	4.69
2	5.63	5.47
2	5.69	5.71
2	4.29	5.34
2	4.28	6.15
2	5.58	4.00
2	6.45	4.83
2	6.08	6.28
2	6.06	6.68
2	5.31	5.86
2	5.73	5.94
2	6.46	5.16
3	8.68	9.72
3	9.62	9.97
3	7.65	9.81
3	8.54	7.08
3	10.46	7.64
3	8.30	7.28
3	9.10	8.25
3	9.52	9.08
3	7.93	9.48
3	9.28	10.19
3	7.50	8.12
3	10.30	8.27
3	7.20	8.42
3	8.92	9.31
3	8.60	9.60
3	7.76	10.52
3	8.95	9.47
3	7.70	9.57
3	9.33	8.39
3	10.50	8.77
3	8.93	8.15
3	8.88	8.92
3	9.60	10.29
3	9.89	10.42
3	9.85	8.07
3	10.51	10.03
3	9.87	7.37
3	7.43	9.90
3	8.84	7.04

Cluster #	x-coordinates	y-coordinates
3	9.02	9.83
3	9.90	10.25
3	7.73	8.33
3	10.32	8.07
3	9.57	10.63
3	10.45	10.33
3	8.97	9.27
3	8.62	9.68
3	8.19	9.37
3	8.69	9.64
3	9.35	10.65
3	9.76	8.43
3	9.88	8.43
3	9.20	9.79
3	9.72	8.73
3	7.82	8.54
3	9.62	8.72
3	9.19	9.05
3	9.33	10.44
3	8.21	7.26
3	9.49	8.65
4	21.92	20.47
4	21.86	20.56
4	19.70	19.02
4	20.83	21.22
4	19.13	18.25
4	20.71	18.29
4	19.07	21.58
4	20.28	20.24
4	19.13	20.18
4	19.13	19.84
4	21.52	20.51
4	19.37	21.16
4	18.95	19.42
4	18.81	19.04
4	21.62	20.88
4	21.43	19.14
4	19.85	19.56
4	19.42	18.27
4	20.63	20.14
4	20.13	21.84
4	19.49	19.60
4	19.50	20.42
4	19.14	21.42
4	19.89	20.29

Cluster #	x-coordinates	y-coordinates
4	21.09	19.99
4	20.08	21.11
4	20.19	21.32
4	18.58	19.24
4	21.38	20.75
4	18.40	19.91
4	19.46	20.61
4	19.40	20.34
4	20.06	19.86
4	20.67	20.82
4	20.26	21.09
4	19.87	21.11
4	19.14	21.21
4	21.42	20.92
4	19.07	20.73
4	19.17	19.71
4	18.96	20.28
4	18.55	20.63
4	19.67	20.86
4	18.22	20.66
4	20.03	21.15
4	20.81	20.07
4	19.40	20.77
4	19.01	19.57
4	19.43	20.84
4	20.41	19.63
5	4.09	19.84
5	4.41	21.57
5	3.73	20.48
5	5.80	19.19
5	6.49	19.07
5	4.52	20.99
5	4.52	18.42
5	6.09	19.51
5	4.08	18.46
5	6.80	20.61
5	5.98	20.28
5	5.82	21.11
5	4.72	20.51
5	3.59	19.83
5	4.99	18.80
5	3.55	19.61
5	4.22	18.64
5	4.83	18.88
5	4.24	20.60

Cluster #	x-coordinates	y-coordinates
5	5.14	19.35
5	3.81	19.94
5	5.43	21.45
5	4.74	18.81
5	5.68	20.68
5	4.67	20.40
5	6.23	19.94
5	5.76	18.38
5	4.56	20.60
5	6.02	20.95
5	3.78	20.51
5	5.82	20.08
5	4.16	19.70
5	4.27	19.07
5	3.57	19.09
5	6.11	20.26
5	6.10	18.40
5	6.07	18.84
5	6.39	21.38
5	4.60	20.51
5	5.49	21.59
5	6.58	19.26
5	3.37	19.30
5	5.47	20.34
5	6.13	20.93
5	4.85	18.35
5	4.38	19.65
5	4.48	21.38
5	5.73	20.64
5	4.87	19.66
5	3.13	20.06
6	19.57	5.37
6	20.29	4.18
6	18.37	5.16
6	19.71	5.05
6	18.16	5.79
6	19.54	6.28
6	20.08	3.35
6	21.37	5.64
6	20.97	4.38
6	19.87	3.94
6	21.51	3.77
6	21.45	4.90
6	21.53	3.74
6	20.37	3.20

Cluster #	x-coordinates	y-coordinates
6	18.50	6.12
6	18.85	5.15
6	19.64	5.07
6	21.24	5.30
6	18.49	4.19
6	19.55	3.10
6	19.66	4.98
6	19.92	5.45
6	19.23	3.65
6	20.93	5.05
6	20.37	5.81
6	18.51	3.99
6	20.20	4.40
6	21.54	6.08
6	18.84	5.18
6	19.44	3.28
6	21.49	4.07
6	20.73	6.44
6	19.81	5.47
6	19.77	5.32
6	18.36	4.43
6	20.13	6.70
6	19.11	6.31
6	20.24	3.23
6	20.04	6.39
6	18.84	3.80
6	19.70	6.78
6	20.20	5.63
6	20.28	3.46
6	21.19	3.60
6	21.03	3.92
6	19.99	4.95
6	20.88	5.53
6	19.12	4.56
6	19.90	5.67
6	21.15	5.52

Appendix D

Sph_10_2
Number of dimensions: 2
Number of clusters: 10
Number of points: 500

Cluster #	x-coordinates	y-coordinates
1	3.51	8.23
1	1.15	7.98
1	0.36	9.5
1	0.75	8.45
1	0.73	8.27
1	-0.1	7.94
1	-0.18	6.3
1	3.26	6.88
1	-0.88	7.83
1	-1.9	7.67
1	2.69	8.71
1	-0.27	8.91
1	2.44	9.5
1	-1.01	8.5
1	-0.89	9.21
1	3.21	7.17
1	1.35	7.64
1	-0.03	8.47
1	3.28	7.86
1	-0.01	7.68
1	3.19	8.95
1	-0.54	8.18
1	0.39	7.13
1	2.75	9.17
1	0.08	7.1
1	-0.42	7.61
1	-1.24	7.36
1	-1.87	8.33
1	-1.38	8.78
1	-0.83	8.1
1	0.64	6.72
1	-0.15	6.55
1	0.36	8.75
1	-0.63	6.76
1	1.37	7.87
1	-1.7	8.83
1	2.58	8.09
1	0.18	7.66
1	-1.26	7.62

Cluster #	x-coordinates	y-coordinates
1	1.37	9.06
1	-0.09	6.72
1	2.72	8.73
1	-0.03	9.43
1	2.38	6.71
1	-0.5	9.21
1	-1.18	6.92
1	-1.68	7.51
1	0.17	9.49
1	1.28	7.82
1	0.31	6.54
2	6.23	3.1
2	6.77	4.38
2	7.09	3.27
2	2.45	4.39
2	6.59	4.56
2	4.22	2.94
2	3.66	5.42
2	2.96	5
2	5.64	4.64
2	3.12	5.01
2	5.39	5.66
2	3.84	5.67
2	3.47	2.65
2	6.53	3.05
2	4.3	3.71
2	3.38	4.3
2	5.03	2.61
2	5.23	5.38
2	7.11	3.54
2	2.55	3.35
2	6.39	2.91
2	5.97	4.79
2	3.57	3.75
2	2.96	3.88
2	5.74	5.3
2	3.13	5.29
2	6.22	3.43
2	4.95	3.88
2	5.42	2.29
2	3.42	3.83
2	4.3	4.15
2	2.97	3.89
2	2.73	4.98
2	4.12	3.66

Cluster #	x-coordinates	y-coordinates
2	6.13	3.26
2	4.91	2.3
2	2.9	5.06
2	2.35	3.55
2	7.36	4.95
2	5.71	2.66
2	6.56	4.28
2	3.65	5.28
2	6.44	2.52
2	3.1	2.9
2	3.72	3.89
2	3.77	3.29
2	6.1	2.2
2	3.9	3.49
2	4.12	4.97
2	3.8	3.71
3	0.74	-5.26
3	-0.22	-6.19
3	-1.44	-6.59
3	-1.81	-6.49
3	0.54	-5.04
3	-3.06	-7.39
3	0.14	-6.69
3	-1.81	-7.64
3	-3.01	-5.02
3	-3.8	-4.65
3	-0.96	-5.37
3	-1.51	-4.37
3	-1.28	-7.34
3	-4.64	-5.19
3	-2.34	-7.03
3	-4.69	-6.23
3	0.09	-6.91
3	-1.14	-6.33
3	-1.91	-4.32
3	-0.82	-4.39
3	-4.51	-5.64
3	-3.06	-4.23
3	0.8	-6.32
3	-1.87	-5.2
3	-1.5	-6.57
3	-0.79	-4.58
3	-0.38	-5.96
3	-4.15	-6.54
3	-3.4	-7.2

Cluster #	x-coordinates	y-coordinates
3	-0.32	-6.29
3	-2.73	-4.26
3	-3.34	-5.03
3	0.1	-4.95
3	-1.17	-4.98
3	-2.93	-5.28
3	-3.71	-6.66
3	-0.27	-7.33
3	-3.05	-7.13
3	-3.03	-7.27
3	-1.8	-7.12
3	-2.04	-4.33
3	-2.72	-4.49
3	-3.25	-5.96
3	-3.86	-7.34
3	-0.34	-6.89
3	0.28	-7.09
3	-3.83	-7.1
3	-2.24	-5.96
3	-0.44	-7.1
3	-3.06	-4.57
4	0.57	1.59
4	-1.1	0.14
4	-0.46	-1.79
4	2.72	-0.34
4	0.97	-0.3
4	0.45	-1.17
4	-1.24	-0.78
4	1.82	0.34
4	0.86	1.69
4	-0.76	-1.92
4	0.26	-0.42
4	1.93	-1.25
4	0.45	0.1
4	-2	0.1
4	2.24	0.82
4	-1.12	1.84
4	1.67	0.82
4	-0.64	-1.36
4	-1.17	1.43
4	0.24	-1.9
4	1.26	-1.43
4	-0.68	0.03
4	-2.25	-1.11
4	-0.6	-1.72

Cluster #	x-coordinates	y-coordinates
4	1.25	0.68
4	-0.71	-1.38
4	0.61	-1.83
4	-1.27	0.98
4	-1.78	-0.14
4	0.26	-1.05
4	1.49	1.3
4	-2.67	-0.53
4	1.22	-1.52
4	-0.76	1.62
4	-0.26	-0.74
4	-0.64	0.39
4	2.66	0.22
4	-0.62	-0.75
4	1.75	0.4
4	2.69	-0.21
4	0.6	-1.84
4	-0.19	1.94
4	-2.72	0.76
4	-2.34	0.73
4	-0.08	-0.06
4	-0.71	0.75
4	1.62	0.3
4	0.03	1.58
4	-1.34	0.95
4	-1.93	1.32
5	8.4	0.95
5	12.67	-0.43
5	11.25	-1.81
5	11.74	-0.54
5	11.83	0.55
5	10.2	-0.81
5	8.66	-1.11
5	9.32	-1.42
5	10.37	-0.59
5	9.23	0.43
5	11.26	-1.32
5	12.18	-0.46
5	9.15	-1.48
5	9.94	0.34
5	11.77	-0.88
5	11.81	0.31
5	13.06	0.02
5	12.45	-0.7
5	10.91	-0.86

Cluster #	x-coordinates	y-coordinates
5	8.85	-0.51
5	10.34	0.15
5	9.99	-0.31
5	11.09	1.02
5	13.07	1.08
5	10.23	-1.75
5	9.04	1.05
5	12.25	-0.38
5	9.91	0.62
5	9.13	0.24
5	11.69	0.28
5	12.58	0.69
5	9.23	-0.66
5	10.49	-0.05
5	12.34	-0.87
5	8.89	0.78
5	8.07	0.3
5	12	0.8
5	12.82	-1.32
5	10.06	-0.04
5	10.62	0.68
5	9.72	0.32
5	10.6	-1.34
5	8.38	0.14
5	12.29	0.89
5	10.05	-1.03
5	11.3	1.54
5	10.07	0.98
5	10.07	-1.09
5	13.66	-0.87
5	8.46	1.07
6	-11.64	1.37
6	-10.71	-0.95
6	-12.22	0.62
6	-10.64	0.57
6	-10.93	0.41
6	-11.43	0.62
6	-13.04	1.65
6	-9.74	-0.11
6	-10.69	-1.03
6	-10.95	-0.37
6	-11.04	1.41
6	-11.99	0.33
6	-9.75	1.04
6	-9.69	1.27

Cluster #	x-coordinates	y-coordinates
6	-10.24	0.13
6	-9.84	-0.18
6	-11.56	-1.1
6	-13.34	1.65
6	-10.35	-1.05
6	-11.92	1.42
6	-13.35	-1.19
6	-10.4	-0.58
6	-11.87	-0.02
6	-10.62	-1.66
6	-10.73	-0.57
6	-12.86	-0.97
6	-10.38	-0.43
6	-9.63	-1.21
6	-12.67	1.68
6	-10.83	0
6	-11.1	0.29
6	-9.78	-1.14
6	-9.8	1.11
6	-10.97	0.73
6	-9.59	-0.87
6	-12.25	1.35
6	-11.72	-1.82
6	-9.69	0.46
6	-10.09	-1.03
6	-12.67	-1.27
6	-9.43	-0.86
6	-12.62	0.78
6	-10.74	0.53
6	-10.74	-1.52
6	-9.07	0.08
6	-13.53	-0.73
6	-10.37	-1.61
6	-12.09	1.71
6	-11.46	0.73
6	-9.31	0.03
7	7.18	9.04
7	5.82	8.79
7	6.97	11.86
7	5.75	9.03
7	6.76	9.48
7	9.28	10.41
7	8.22	11.69
7	6.59	8.8
7	8.38	8.71

Cluster #	x-coordinates	y-coordinates
7	5.71	9.23
7	9.12	10.29
7	10.22	10.54
7	6.78	10.07
7	6.6	10.11
7	8.04	8.51
7	6.1	9.34
7	10.67	9.58
7	6.43	9.5
7	5.38	9.55
7	6.54	10.07
7	5.82	10.07
7	10.18	9.18
7	5.36	10.12
7	7.81	8.68
7	5.62	9.1
7	8.48	8.95
7	7.79	10.12
7	9.36	9.48
7	8.27	9.74
7	10	10.04
7	5.5	10.49
7	9.76	8.47
7	7.58	11.9
7	10.5	10.88
7	10.23	9.9
7	7.6	11.41
7	6.87	9.26
7	8.05	8.02
7	6.38	11.31
7	6.59	8.6
7	5.89	9.83
7	10.9	9.58
7	10.84	10.42
7	10.08	9.68
7	8.17	10.86
7	10.35	9.36
7	6.01	9.17
7	9.6	11.25
7	6.51	9.79
7	9.25	8.77
8	12.4	3.31
8	15.47	2.23
8	15.06	1.62
8	12.71	3.93

Cluster #	x-coordinates	y-coordinates
8	17.59	2.61
8	13.96	3.05
8	17.51	2.37
8	13.47	3.63
8	16.56	3.13
8	13.99	1.71
8	17.04	1.81
8	14.35	4.39
8	16.72	3.98
8	16.15	1.92
8	17.87	2.99
8	13.09	2.1
8	17.26	2.16
8	15.78	4.6
8	13	2.13
8	13.77	1.46
8	14.89	2.92
8	17.43	3.72
8	17.77	2.69
8	16.98	2.6
8	17.39	3.18
8	14.65	4.43
8	12.7	3.55
8	15.68	3.58
8	13.03	2.59
8	16.16	4.09
8	13.04	2.97
8	15.04	1.1
8	13.68	3.41
8	16.19	4.36
8	16.9	3.12
8	16.3	3.44
8	14.04	4.03
8	16.58	2.88
8	16.22	4.05
8	13.37	1.83
8	13.34	1.34
8	17	2.2
8	16.35	3.61
8	16.19	1.98
8	13.54	4.16
8	16.45	1.48
8	16.57	2.9
8	17.15	1.95
8	16.5	3.1

Cluster #	x-coordinates	y-coordinates
8	13.45	4.22
9	-17.15	-1.94
9	-15.84	-4.15
9	-15.42	-1.77
9	-15.88	-4.2
9	-16.26	-3.35
9	-13.71	-3.24
9	-15.19	-1.71
9	-17.64	-3.9
9	-15.82	-3.47
9	-13.85	-4.61
9	-13.01	-3.64
9	-15.98	-3.49
9	-17.27	-4.29
9	-15.09	-3.65
9	-16.18	-2.44
9	-16.13	-1.47
9	-12.34	-2.87
9	-14.55	-4.11
9	-16.81	-3.41
9	-15.8	-3.63
9	-14.44	-1.84
9	-17.78	-3.31
9	-14.9	-4.27
9	-17.2	-4.32
9	-13.01	-3.99
9	-13.74	-2.83
9	-12.18	-2.92
9	-14.23	-2.26
9	-16.51	-3.37
9	-13.42	-2.78
9	-14.31	-2.18
9	-13.63	-4.24
9	-16.18	-3.5
9	-14.46	-2.32
9	-15.08	-4
9	-16.1	-3.55
9	-15.69	-1.1
9	-17.39	-3.1
9	-15.85	-1.94
9	-15.74	-3.54
9	-14.06	-1.3
9	-15.92	-4.38
9	-12.78	-2.33
9	-13.12	-3.82

Cluster #	x-coordinates	y-coordinates
9	-12.94	-3.46
9	-17.66	-2.42
9	-13.37	-1.88
9	-14.38	-2.81
9	-16.39	-2.04
9	-13.94	-4.29
10	-5.1	2.07
10	-9.66	2.24
10	-6.91	2.66
10	-7.19	3.22
10	-5.2	2.76
10	-7.76	1.88
10	-4.65	4.2
10	-4.36	2.86
10	-7.5	4.16
10	-9.62	2.82
10	-7.37	2.25
10	-4.58	3.41
10	-7.8	1.62
10	-9.26	2.93
10	-9.11	2.78
10	-5.27	3.19
10	-4.36	3.25
10	-4.55	2.85
10	-9.05	2.72
10	-7.46	1.85
10	-7.71	2.2
10	-4.48	2.9
10	-8.91	3.16
10	-8.7	4.34
10	-9.77	3.49
10	-8.36	3.63
10	-9.3	2.03
10	-6.03	3.55
10	-5.5	2.07
10	-8.12	2.91
10	-6.83	2.15
10	-5.19	2.13
10	-8.37	1.31
10	-7.15	4.51
10	-7.81	3.17
10	-7.97	3.8
10	-7.31	1.38
10	-5.07	3.48
10	-5.3	3.19

Cluster #	x-coordinates	y-coordinates
10	-9.83	2.83
10	-7.1	5
10	-4.93	3
10	-5.28	4.25
10	-6.12	1.61
10	-6.61	1.17
10	-6.19	2.09
10	-8.57	3.76
10	-8.44	2.62
10	-7.33	1.8
10	-6.85	4.63

Appendix E

Sph_9_2
Number of dimensions: 2
Number of clusters: 9
Number of points: 900

Cluster #	x-coordinates	y-coordinates
1	-3.032441	1.158165
1	-2.414818	1.72212
1	-2.213644	2.678879
1	-2.472206	2.896035
1	-2.76633	1.271111
1	-2.412839	1.896345
1	-2.796668	1.606939
1	-2.407333	1.926278
1	-0.968905	2.500413
1	-1.94932	2.086108
1	-2.083773	2.867428
1	-2.18951	1.882033
1	-1.958493	2.689939
1	-1.434849	2.906251
1	-1.949031	1.698587
1	-1.868516	2.144176
1	-1.662504	1.801115
1	-0.920653	2.522056
1	-1.036697	2.379066
1	-0.925738	1.723582
1	-2.077808	1.3093
1	-2.640381	1.921389
1	-1.809468	3.162982
1	-2.027487	2.441138
1	-1.606598	1.332476
1	-2.453172	1.037615
1	-2.101243	2.067889
1	-1.249761	1.941452
1	-2.71709	2.087638
1	-2.30162	3.285637
1	-1.016156	2.105078
1	-1.087677	1.834475
1	-1.474888	1.254246
1	-2.255914	2.111397
1	-2.097162	2.333326
1	-2.383669	1.244008
1	-2.515849	2.101071
1	-2.394256	2.87114
1	-2.492071	2.375098

Cluster #	x-coordinates	y-coordinates
1	-2.367047	1.620038
1	-2.844092	1.69237
1	-2.459592	1.947833
1	-1.575574	1.118759
1	-1.919276	1.941867
1	-1.615261	0.950021
1	-1.51549	1.637475
1	-2.320183	1.761284
1	-1.670614	1.535113
1	-2.12002	2.27079
1	-2.777068	1.594687
1	-1.551383	1.520723
1	-1.658207	2.676311
1	-1.877004	1.059083
1	-1.567288	2.796054
1	-1.491027	1.660519
1	-2.159901	1.617062
1	-1.55507	1.054893
1	-2.754005	1.235793
1	-1.537826	2.631603
1	-2.053987	2.989606
1	-2.091644	2.953341
1	-0.898505	1.844794
1	-2.395681	1.303772
1	-1.676072	2.275712
1	-1.665179	2.177637
1	-1.615373	1.90545
1	-2.000655	1.9251
1	-2.517429	2.385077
1	-2.569233	2.620279
1	-0.955933	2.361975
1	-2.523317	2.545208
1	-1.701527	2.381814
1	-1.85435	1.614697
1	-2.14717	1.022456
1	-1.99226	1.857206
1	-2.39705	1.71924
1	-1.808119	1.8296
1	-2.096134	2.908089
1	-2.911128	2.51224
1	-2.194817	1.363051
1	-2.271291	2.853696
1	-1.948123	2.29262
1	-2.968241	1.018432
1	-1.362506	1.945724

Cluster #	x-coordinates	y-coordinates
1	-2.343894	2.306488
1	-1.756022	1.773653
1	-1.116165	1.311918
2	-0.011177	1.705894
2	-0.65397	2.226539
2	-0.197106	2.102319
2	0.662488	1.460443
2	0.555874	1.461527
2	-0.731151	1.971621
2	0.547534	2.003953
2	-0.60349	2.662569
2	0.015024	2.209096
2	-0.293286	3.079113
2	-0.339427	1.835793
2	-0.356383	0.803076
2	0.659985	1.954799
2	0.47414	2.181734
2	0.171313	1.035007
2	0.151562	2.761806
2	0.096262	1.018594
2	0.824706	0.979255
2	-0.16067	1.464974
2	-0.328164	1.094403
2	-0.177223	1.319707
2	-0.276317	1.703893
2	0.828738	2.003357
2	0.853811	2.583847
2	-0.326361	1.908826
2	0.031099	2.371091
2	-0.906513	3.237391
2	0.801094	3.10972
2	0.454941	1.475753
2	-0.030409	2.298267
2	0.088702	2.082756
2	-0.41377	1.249949
2	-0.542547	2.844658
2	0.866832	1.276236
2	-0.352782	2.118927
2	-0.78552	1.586172
2	-0.146052	2.514995
2	0.202967	1.976761
2	-0.297959	2.581547
2	0.129411	2.714617
2	-0.456712	1.501777
2	0.50559	2.736068

Cluster #	x-coordinates	y-coordinates
2	0.551727	1.970282
2	0.906775	2.267937
2	-0.391867	2.028704
2	-0.254087	1.824659
2	-0.462481	1.880898
2	0.956329	2.167299
2	0.345406	2.158048
2	-0.414818	1.714065
2	0.133118	1.585881
2	0.652295	2.184921
2	-0.319604	1.59509
2	0.676311	2.667687
2	0.340502	2.273299
2	0.58464	2.813128
2	0.533477	1.589715
2	-0.337979	1.150102
2	-0.336854	1.394508
2	0.183257	2.558096
2	-0.969331	1.924932
2	-0.405025	1.087011
2	0.527512	2.021906
2	0.235562	1.441278
2	0.472206	2.126331
2	-0.839589	1.843757
2	0.573409	2.164116
2	-0.706306	2.135684
2	-0.576396	1.682865
2	0.930999	1.855735
2	-0.888446	1.895871
2	-0.055395	2.421721
2	-0.994125	1.61402
2	0.192952	2.495589
2	-0.571707	2.800371
2	-0.875491	2.780532
2	0.730697	2.583631
2	0.247745	2.944381
2	0.888196	1.902752
2	-0.535701	2.335623
2	0.240954	1.24675
2	0.304361	2.262355
2	0.506174	2.548151
2	-0.613334	2.037701
2	0.322291	2.34492
2	0.086278	2.059795
2	-0.365611	2.347461

Cluster #	x-coordinates	y-coordinates
2	0.741767	1.756657
2	0.331726	1.130421
2	-0.151652	1.511877
2	0.540575	2.540847
2	0.686735	2.305553
2	-0.535026	2.099738
2	0.040401	0.882815
2	0.987783	3.011837
2	0.196265	2.369372
2	-0.502868	1.463016
2	1.226761	2.449223
2	-0.290472	2.777167
2	-0.549937	1.441765
2	0.29175	2.575755
2	-0.924625	1.741366
2	0.070949	2.074774
2	0.679461	2.545071
2	-0.144444	2.516968
2	0.198884	2.021502
2	0.788738	2.444266
2	0.234787	2.50345
2	0.247696	2.36214
2	-0.148378	2.202732
2	0.510997	1.098825
2	-0.841385	2.229041
2	-0.144935	1.316704
2	-0.233481	2.547397
2	0.525245	1.588032
2	-0.438141	1.732262
3	2.040196	1.818911
3	2.281874	1.472221
3	2.015626	1.972188
3	2.553107	1.682078
3	1.91771	2.139633
3	2.030653	1.62334
3	2.24188	2.492965
3	2.119233	1.736949
3	1.862631	2.90364
3	1.856359	2.206405
3	2.268337	2.119233
3	1.765358	2.210373
3	2.377331	2.654129
3	2.081909	1.901207
3	2.740383	2.31373
3	1.476616	2.504746

Cluster #	x-coordinates	y-coordinates
3	2.388919	1.389215
3	1.389739	1.151589
3	1.526983	2.031262
3	1.464231	2.55629
3	1.732462	1.697088
3	2.12994	1.232413
3	1.979707	1.928631
3	1.374923	1.568061
3	1.907545	1.591973
3	1.431473	2.034885
3	2.042327	1.483493
3	1.599745	1.795339
3	1.090196	2.647772
3	1.06549	1.696312
3	1.315113	1.133644
3	2.621342	2.429625
3	1.811512	0.880258
3	1.580332	1.496938
3	1.705125	1.993856
3	1.608189	1.342829
3	1.913552	1.941784
3	1.961195	2.673341
3	2.570928	1.705792
3	0.96348	2.556984
3	2.094593	2.739554
3	2.154125	2.895653
3	1.737197	1.997558
3	1.721918	1.728759
3	2.15453	1.405852
3	2.555528	0.987087
3	1.414061	1.543777
3	2.809855	1.819464
3	1.169287	2.195891
3	2.016068	2.084834
3	2.086915	1.874328
3	2.908748	1.809747
3	2.591959	2.184967
3	2.517298	2.743896
3	1.402556	1.607849
3	1.778015	2.084409
3	1.408113	1.398064
3	1.918895	2.317292
3	1.953236	1.824934
3	3.028423	2.333539
3	1.925142	1.746307

Cluster #	x-coordinates	y-coordinates
3	1.962462	2.726995
3	2.614913	2.311275
3	2.089384	1.885736
3	2.819204	2.183627
3	2.69367	2.341147
3	2.85358	1.53795
3	1.399613	1.989223
3	2.504552	2.272244
3	1.344432	1.167526
3	2.920389	2.649039
3	1.937124	1.747243
3	1.334267	1.786451
3	3.008633	2.390449
3	2.113873	2.349847
3	2.640772	1.426875
3	2.106676	1.318541
3	1.509651	2.190904
3	1.999305	2.711717
3	1.076883	1.998631
3	2.071621	2.102233
3	1.847134	2.297444
3	2.1539	1.793973
3	2.408201	2.033581
3	1.814385	2.293952
3	2.870899	2.085301
3	1.952207	2.711016
3	2.664027	1.952907
3	2.842736	2.989274
3	2.816316	1.691434
3	1.971986	1.932696
3	1.514858	1.379341
3	2.194211	3.011837
3	2.899755	1.11268
3	2.402838	2.328058
4	-1.534681	1.003197
4	-1.882905	0.783619
4	-2.228993	-0.323981
4	-1.953524	-0.540983
4	-1.422819	0.366605
4	-2.179661	-0.160263
4	-1.772403	-0.037906
4	-1.807281	0.192393
4	-2.153675	0.328536
4	-2.19095	-0.544252
4	-3.090128	-0.018239

Cluster #	x-coordinates	y-coordinates
4	-1.793501	0.020898
4	-1.912617	-0.221363
4	-2.093694	0.758939
4	-2.173554	0.457875
4	-3.031095	0.34887
4	-2.504163	0.024936
4	-2.097248	-0.123434
4	-1.571382	-0.576967
4	-1.465715	0.177729
4	-2.241588	-1.078181
4	-1.869884	0.388862
4	-1.972878	0.491752
4	-1.773172	0.118622
4	-1.360713	0.350716
4	-2.087255	-0.274706
4	-2.298216	-0.061501
4	-1.632842	0.47514
4	-2.370925	0.655168
4	-0.950227	-0.971994
4	-1.394953	-0.171999
4	-1.748523	-0.828082
4	-1.637255	-0.988942
4	-2.000615	0.073091
4	-2.299504	0.701461
4	-1.03593	0.625688
4	-1.64591	0.494757
4	-1.727204	0.036435
4	-2.92216	-0.414177
4	-1.217379	0.577538
4	-2.027041	-0.301052
4	-1.338886	0.228511
4	-2.071411	-0.69265
4	-1.970485	0.644695
4	-2.525978	0.805246
4	-1.706407	0.107109
4	-1.71696	-0.493477
4	-2.669239	-0.379233
4	-3.034181	-0.584712
4	-1.352623	-0.24564
4	-2.21845	-0.853927
4	-2.930301	0.699482
4	-2.889073	-0.043558
4	-2.007021	0.115265
4	-2.782123	-0.760562
4	-1.378049	1.0926

Cluster #	x-coordinates	y-coordinates
4	-1.242109	0.441078
4	-1.661753	-0.255074
4	-2.463282	-0.425251
4	-2.089767	0.205603
4	-1.580157	0.567963
4	-2.854042	0.390109
4	-1.233477	0.473453
4	-2.169259	0.068769
4	-1.953401	0.534824
4	-1.866838	0.427967
4	-1.368165	-0.382038
4	-2.936633	-0.642495
4	-2.070404	0.654609
4	-2.00738	0.160127
4	-2.73937	0.032035
4	-1.694965	0.182611
4	-1.59532	-0.543024
4	-2.569092	-0.652375
4	-2.156874	0.07032
4	-2.042819	-0.115962
4	-2.435392	-0.363901
4	-2.200056	-0.504617
4	-1.524798	-0.530724
4	-2.2452	0.446076
4	-1.263383	-0.75495
4	-1.483098	0.640928
4	-2.391073	-0.087766
4	-1.39954	-0.924488
4	-1.766084	-0.926001
4	-2.070865	-0.017033
4	-1.915633	-0.402838
4	-2.133648	-0.464022
4	-1.453288	0.313207
4	-1.476151	-0.186541
4	-1.713149	-0.926415
4	-1.895785	0.318553
4	-2.295337	0.229185
4	-1.517259	-0.070404
4	-0.801055	0.56888
4	-3.162607	-0.705698
4	-2.217592	-0.488313
4	-2.239446	0.973254
4	-2.701116	-0.426549
4	-2.014422	-0.502674
4	-2.229282	0.212695

Cluster #	x-coordinates	y-coordinates
4	-1.86405	-0.558026
5	-0.067806	0.761232
5	0.699396	0.854389
5	0.422896	0.735702
5	0.232224	-0.158228
5	-0.468848	-0.317975
5	0.007859	-0.948759
5	-0.077935	0.172182
5	0.565853	0.135109
5	-0.071831	0.865405
5	-0.564519	0.221028
5	0.477018	-0.193278
5	0.817384	-0.317922
5	-1.171933	0.420136
5	-0.402263	0.199634
5	0.055395	-0.251084
5	-0.807755	0.131748
5	-0.339588	-0.045201
5	0.01358	0.222272
5	0.710928	0.669566
5	-0.391073	0.479591
5	0.449587	0.550763
5	-0.03905	-0.555736
5	-0.454392	1.016529
5	-0.096648	-0.105424
5	0.704311	-0.11679
5	0.630447	-0.369206
5	-0.991439	-0.720551
5	-0.870299	-0.372925
5	-0.277325	-0.119583
5	0.637883	0.242514
5	0.327156	0.117138
5	0.34757	0.469531
5	-0.029069	0.772062
5	0.120457	-0.118404
5	0.19109	1.068398
5	0.0757	0.454575
5	0.056141	0.067638
5	-0.540304	0.468911
5	-0.237891	-0.372147
5	-0.207868	-0.454941
5	0.372925	-0.918357
5	-0.65381	0.891213
5	-0.640928	-0.8691
5	-0.635628	-0.28071

Cluster #	x-coordinates	y-coordinates
5	0.453904	-0.23658
5	-0.252511	-1.145636
5	0.175204	-0.532939
5	1.203115	0.822649
5	0.574403	0.546028
5	0.497708	-0.426254
5	-0.014623	0.511128
5	-0.470153	-0.322502
5	0.00738	-0.225147
5	0.104733	-0.170263
5	-0.402034	-1.269531
5	-0.385585	-0.618612
5	-0.448859	1.20803
5	0.030612	-0.339052
5	0.186402	-0.299659
5	-0.412026	0.411097
5	-0.250494	-0.716913
5	-0.055892	-0.356274
5	-0.065756	0.192393
5	-0.346379	0.002641
5	-0.563538	0.433962
5	0.607202	-1.00337
5	0.309191	0.387957
5	-0.663946	-0.12453
5	0.349738	0.939483
5	0.529451	0.272094
5	-0.983029	0.010376
5	-0.084622	0.82319
5	1.242546	-0.555874
5	-0.760084	0.716736
5	0.060586	0.032767
5	-0.112135	0.133206
5	-0.010937	-0.057884
5	0.316663	0.449284
5	0.292313	-0.041302
5	-0.672272	-0.577752
5	-0.570363	0.55629
5	-0.175204	-0.662407
5	-0.469966	-0.343085
5	-0.002482	-0.444206
5	0.726365	0.354363
5	0.352238	0.618309
5	0.380523	0.422014
5	-0.759416	0.206971
5	-0.681209	0.078442

Cluster #	x-coordinates	y-coordinates
5	-0.433069	0.432177
6	0.984379	0.236823
6	2.052167	0.863156
6	3.089637	-0.982379
6	2.874399	0.887695
6	1.544266	-0.04973
6	1.235696	-0.337979
6	1.721161	0.915932
6	2.330289	-0.321658
6	1.649773	0.605566
6	2.232127	0.508126
6	1.885692	0.180628
6	2.035783	-0.065882
6	2.654129	0.371813
6	2.303274	-0.742137
6	1.84583	-0.887945
6	1.856538	0.199353
6	1.31528	-0.977864
6	2.589051	-0.287768
6	2.433723	-0.333326
6	1.416369	-1.094098
6	2.434438	-0.57277
6	2.327475	-0.075153
6	0.906152	-0.552693
6	1.053291	-0.410865
6	1.25406	0.009057
6	0.710156	0.314619
6	2.897442	0.460636
6	1.882905	-0.284918
6	2.860332	0.27889
6	2.244467	-0.420547
6	1.877924	-0.258238
6	2.115309	-0.282432
6	1.810955	0.740383
6	1.326577	-0.2393
6	2.175158	-0.162664
6	1.749408	0.325144
6	2.563538	-1.098401
6	3.264818	0.175158
6	2.760084	0.08212
6	1.740474	-0.81717
6	0.760938	0.322027
6	1.878318	0.418906
6	1.110424	-0.287971
6	1.702247	0.747525

Cluster #	x-coordinates	y-coordinates
6	1.329779	0.135596
6	2.481543	-0.759989
6	2.703965	0.808384
6	2.249561	-0.674659
6	1.828001	-0.616345
6	2.018601	-0.353872
6	1.836383	-0.342654
6	2.436706	-0.288583
6	1.174425	0.34147
6	1.102301	-0.120107
6	2.348979	0.885325
6	1.685747	0.748459
6	2.368319	0.594879
6	1.386816	0.668422
6	2.374652	-0.10659
6	2.534083	-0.011657
6	2.057427	0.022633
6	2.478586	0.097248
6	2.259526	0.529718
6	1.283441	1.178973
6	2.365556	-0.017234
6	2.203343	-0.20452
6	2.356055	0.464516
6	2.295491	-0.413421
6	1.591163	1.113277
6	2.197761	0.618612
6	2.283496	0.198182
6	3.198436	0.595977
6	3.067066	0.152461
6	2.167026	0.176305
6	1.592551	0.353818
6	2.337818	-1.05393
6	2.304051	-0.157822
6	2.1736	0.346217
6	1.201072	0.609514
6	2.489267	-0.699826
6	2.169487	0.694948
6	1.19004	0.57348
6	2.820278	-0.672272
6	1.829418	1.005114
6	2.514535	-0.008618
6	2.637494	0.307682
6	2.074058	-0.363295
6	2.187282	0.784119
6	1.70963	0.433723

Cluster #	x-coordinates	y-coordinates
6	1.854127	-0.00272
6	2.337068	-0.571424
6	1.890684	0.573977
6	2.020494	-0.44892
6	2.238425	-0.582193
6	2.400312	-0.910333
6	2.885325	-0.551727
6	2.013099	0.601641
6	1.767341	0.460943
6	2.293388	0.016148
7	-1.357818	-2.071201
7	-2.225339	-1.938249
7	-2.714617	-2.528982
7	-1.690757	-2.763342
7	-2.432296	-2.455307
7	-1.26017	-0.87911
7	-2.310441	-1.740028
7	-2.450011	-2.749208
7	-1.830787	-0.956537
7	-2.54774	-2.758177
7	-2.147214	-1.876566
7	-2.068476	-2.073554
7	-2.578824	-2.184458
7	-0.798469	-1.324846
7	-2.08047	-0.936886
7	-2.148916	-1.755827
7	-1.905536	-1.678553
7	-1.616724	-2.507085
7	-1.820247	-2.322608
7	-2.659099	-2.788335
7	-2.355891	-2.167071
7	-1.637695	-2.720729
7	-1.921685	-1.136607
7	-1.119252	-1.61695
7	-2.385077	-1.322945
7	-1.508695	-2.506759
7	-2.564659	-2.897058
7	-2.555944	-2.878295
7	-2.019689	-1.947792
7	-1.590874	-2.040155
7	-2.549593	-1.665874
7	-2.470588	-1.709324
7	-2.833026	-2.472767
7	-1.958698	-2.571707
7	-2.594294	-1.997677

Cluster #	x-coordinates	y-coordinates
7	-1.778541	-1.795151
7	-1.987582	-1.792888
7	-1.181439	-0.916881
7	-1.720403	-2.378282
7	-2.461497	-1.732712
7	-1.75524	-1.846954
7	-0.927104	-1.889991
7	-1.578514	-2.036354
7	-2.582984	-1.339047
7	-2.575043	-1.907502
7	-1.413411	-1.808864
7	-2.198884	-2.31682
7	-2.256952	-2.052456
7	-2.076627	-2.462851
7	-2.300793	-2.463899
7	-2.921069	-1.941119
7	-2.372592	-1.520031
7	-1.172464	-2.887695
7	-2.763726	-1.18315
7	-3.056035	-1.199319
7	-1.920629	-1.667208
7	-1.602207	-2.981244
7	-2.65677	-0.984923
7	-1.292215	-1.917541
7	-1.915633	-1.725042
7	-0.85767	-2.669075
7	-1.627686	-1.412904
7	-1.847359	-1.479202
7	-2.626453	-2.411968
7	-1.876215	-2.401919
7	-1.254153	-2.081697
7	-2.276166	-1.661056
7	-0.932934	-2.487678
7	-2.304776	-1.539794
7	-1.878274	-2.335409
7	-0.948573	-1.896216
7	-2.044133	-2.331993
7	-1.835021	-2.390279
7	-1.916608	-2.071201
7	-1.832564	-2.831372
7	-2.2344	-2.197433
7	-2.392151	-1.252568
7	-2.318027	-2.374373
7	-0.894038	-1.226176
7	-2.832585	-1.406582

Cluster #	x-coordinates	y-coordinates
7	-2.967155	-2.462112
7	-2.81017	-2.156649
7	-1.88164	-2.473017
7	-2.121769	-0.8206
7	-2.047669	-2.221985
7	-2.043886	-0.814457
7	-2.246668	-1.972675
7	-2.956029	-2.244076
7	-2.287411	-2.718684
7	-1.223326	-2.768945
7	-2.057884	-2.013981
7	-1.781741	-2.295799
7	-2.807545	-1.629464
7	-1.786118	-1.885214
7	-2.03232	-1.571322
7	-1.627297	-2.060835
7	-2.096862	-2.148692
7	-2.246619	-1.402336
7	-2.412781	-2.303792
7	-1.787447	-1.583782
7	-1.752549	-2.055146
7	-1.906989	-1.90009
7	-1.84565	-2.967155
7	-0.955328	-2.038274
7	-1.88921	-1.952248
8	0.031953	-1.131856
8	0.196311	-1.452191
8	0.537458	-2.937201
8	-0.18178	-1.132572
8	-0.362855	-1.772884
8	-0.583847	-2.093609
8	-0.257792	-2.281115
8	0.532402	-2.420488
8	-0.353545	-2.428322
8	-0.449587	-2.149274
8	-0.618234	-1.84028
8	0.409474	-1.680553
8	-0.078906	-2.283446
8	-0.119059	-2.007181
8	0.469842	-2.029069
8	-0.529852	-2.11157
8	0.536241	-2.584352
8	0.16935	-2.341093
8	-0.178832	-1.961072
8	0.90338	-1.815541

Cluster #	x-coordinates	y-coordinates
8	0.161259	-2.169076
8	0.055934	-2.189092
8	0.939483	-2.465937
8	0.683129	-1.675544
8	0.250101	-1.522606
8	0.509886	-2.698967
8	-0.30255	-1.986981
8	0.037865	-2.2195
8	0.317292	-1.546462
8	-0.695289	-1.441487
8	0.837688	-1.705587
8	-0.131528	-2.019044
8	0.30069	-1.234154
8	0.87683	-2.545618
8	0.70735	-1.366155
8	0.465319	-2.382432
8	-1.004241	-2.5322
8	0.692565	-2.132411
8	-0.683798	-2.423778
8	-0.443965	-2.623016
8	0.651181	-1.935872
8	0.49386	-2.954682
8	-0.043517	-2.394199
8	0.400255	-1.107777
8	-0.116572	-2.112309
8	-0.382038	-2.277527
8	-0.29088	-1.799756
8	-0.305657	-1.800225
8	0.834575	-2.135108
8	-0.366439	-1.193083
8	0.260667	-1.712385
8	0.556637	-1.615542
8	0.490986	-2.585939
8	-1.096365	-1.944605
8	0.036231	-1.954388
8	0.84251	-2.071621
8	-0.725826	-1.637475
8	-0.565361	-2.016068
8	-0.90338	-2.113525
8	-0.748553	-3.107296
8	-0.804517	-2.217926
8	0.00778	-2.835129
8	-0.306799	-2.036435
8	-0.17525	-1.470348
8	-0.265541	-1.28837

Cluster #	x-coordinates	y-coordinates
8	-0.467856	-2.631681
8	-0.515652	-1.729861
8	0.04516	-2.87102
8	-0.642652	-1.69807
8	0.11631	-1.676389
8	0.263648	-1.852875
8	0.007341	-1.848797
8	0.620507	-1.985618
8	-0.238473	-2.657893
8	0.778451	-1.323689
8	-0.66063	-1.622334
8	-0.106071	-1.850143
8	-0.297907	-2.312319
8	-0.982379	-2.274756
8	-0.647298	-2.950525
8	0.658456	-1.381161
8	0.668912	-1.268396
8	1.038485	-2.00754
8	0.613484	-1.984052
8	-0.265143	-2.502997
8	-0.075953	-3.025401
8	-0.039664	-1.416513
8	0.501768	-1.955498
8	0.69087	-1.132334
8	-0.879152	-1.860589
8	-0.812916	-3.202055
8	-0.178602	-2.150575
8	1.020745	-1.398137
8	-0.553936	-2.388976
8	-0.774805	-2.02769
8	-0.072293	-2.027487
8	-0.597297	-1.123901
8	0.997346	-1.769658
8	-0.227645	-2.50767
8	0.095063	-2.216925
8	-0.000178	-1.280428
8	0.107238	-2.247059
8	0.741029	-1.114551
8	-0.492837	-2.521593
8	-0.461435	-1.811372
8	-1.162983	-1.769272
8	0.099996	-1.708864
8	-1.009875	-1.808305
8	0.472268	-2.857292
8	-0.446258	-2.759893

Cluster #	x-coordinates	y-coordinates
8	0.945397	-1.988903
9	1.600776	-1.207922
9	1.608189	-1.687368
9	2.431226	-1.647599
9	1.58809	-1.78702
9	1.99258	-1.712844
9	1.531772	-1.84281
9	2.826119	-3.031671
9	1.58524	-2.920118
9	2.048164	-2.755992
9	2.614687	-2.057552
9	1.788158	-1.530593
9	1.641647	-1.748375
9	2.277224	-2.259873
9	1.842042	-2.172273
9	1.019886	-1.409278
9	2.020575	-1.625014
9	0.793085	-1.782979
9	1.790762	-1.278023
9	2.653251	-1.111804
9	2.235611	-2.666302
9	2.508061	-2.141502
9	1.831699	-2.848411
9	1.694913	-2.597297
9	2.367489	-2.582624
9	1.250136	-1.6087
9	2.390562	-1.565085
9	1.845244	-1.454929
9	2.394825	-2.599944
9	2.613709	-2.714177
9	1.686165	-2.091857
9	1.801584	-2.29221
9	2.799443	-2.476454
9	2.306747	-1.756998
9	2.734972	-1.837699
9	1.721868	-1.601806
9	2.755518	-2.744082
9	2.164525	-2.638273
9	1.923626	-2.857526
9	1.494929	-2.95082
9	1.735356	-2.481795
9	1.832792	-2.04664
9	2.010696	-1.567942
9	2.306436	-1.437302
9	1.447376	-1.647762

Cluster #	x-coordinates	y-coordinates
9	1.659713	-2.256457
9	2.447588	-2.501639
9	2.093994	-1.324928
9	2.506305	-2.790355
9	1.679711	-2.382038
9	1.411238	-1.727154
9	1.619645	-1.49991
9	2.371258	-2.038682
9	2.033459	-2.468228
9	1.917583	-2.863984
9	2.851735	-2.510736
9	1.943154	-2.48129
9	1.224507	-2.459653
9	1.59232	-3.199971
9	1.991302	-1.246279
9	2.037048	-1.557239
9	1.732612	-2.916604
9	1.788253	-1.635382
9	1.491418	-1.546949
9	1.878274	-2.216306
9	1.70533	-1.573687
9	1.379265	-2.825575
9	1.705022	-1.083396
9	2.063627	-2.188767
9	1.663039	-2.129631
9	1.370477	-1.867501
9	1.983972	-1.640387
9	2.182195	-1.633892
9	1.693253	-2.265292
9	1.130421	-1.530468
9	1.491353	-1.218574
9	1.674009	-1.430767
9	2.469345	-3.018537
9	2.079793	-1.505051
9	2.308566	-1.362895
9	1.711468	-2.532267
9	1.494929	-1.906733
9	1.954635	-1.66987
9	2.635395	-2.66055
9	1.704509	-2.312528
9	1.506076	-2.274857
9	1.92371	-1.869884
9	1.951465	-1.442182
9	2.820923	-1.451299
9	2.72448	-1.848213

Cluster #	x-coordinates	y-coordinates
9	2.047546	-1.610175
9	1.915378	-1.444056
9	1.399982	-1.848303
9	2.436228	-1.556577
9	1.860811	-1.986059
9	1.953483	-2.668749

Appendix F

Pat1

Number of dimensions: 2

Number of clusters: 3

Number of points: 557

Cluster #	x-coordinates	y-coordinates
1	350	1200
1	350	1250
1	350	1300
1	350	1350
1	350	1400
1	350	1450
1	350	1500
1	350	1550
1	350	1600
1	350	1650
1	350	1700
1	350	1750
1	350	1800
1	350	1850
1	350	1900
1	350	1950
1	350	2000
1	350	2050
1	350	2100
1	350	2150
1	350	2200
1	350	2250
1	350	2300
1	350	2350
1	350	2400
1	375	1150
1	375	1200
1	375	1250
1	375	1300
1	375	1350
1	375	1400
1	375	1450
1	375	1500
1	375	1550
1	375	1600
1	375	1650
1	375	1700
1	375	1750
1	375	1800
1	375	1850
1	375	1900
1	375	1950
1	375	2000

Cluster #	x-coordinates	y-coordinates
1	375	2050
1	375	2100
1	375	2150
1	375	2200
1	375	2250
1	375	2300
1	375	2350
1	375	2400
1	375	2450
1	400	1100
1	400	1150
1	400	2450
1	400	2500
1	425	1050
1	425	1100
1	425	2500
1	425	2550
1	450	1050
1	450	1100
1	450	2500
1	450	2550
1	475	1000
1	475	1050
1	475	2550
1	475	2600
1	500	950
1	500	1000
1	500	2600
1	500	2650
1	525	950
1	525	1000
1	525	2600
1	525	2650
1	550	950
1	550	1000
1	550	2600
1	550	2650
1	575	950
1	575	1000
1	575	2600
1	575	2650
1	600	1000
1	600	1050
1	600	2550
1	600	2600
1	625	1050
1	625	1100
1	625	2500
1	625	2550

Cluster #	x-coordinates	y-coordinates
1	650	1050
1	650	1100
1	650	2500
1	650	2550
1	675	1100
1	675	1150
1	675	2450
1	675	2500
1	700	1150
1	700	1200
1	700	1250
1	700	1300
1	700	2300
1	700	2350
1	700	2400
1	700	2450
1	725	1200
1	725	1250
1	725	1300
1	725	1350
1	725	2250
1	725	2300
1	725	2350
1	725	2400
1	750	1300
1	750	1350
1	750	1400
1	750	1450
1	750	1500
1	750	1550
1	750	1600
1	750	1650
1	750	1700
1	750	1750
1	750	1800
1	750	1850
1	750	1900
1	750	1950
1	750	2000
1	750	2050
1	750	2100
1	750	2150
1	750	2200
1	750	2250
1	750	2300
1	775	1350
1	775	1400
1	775	1450
1	775	1500

Cluster #	x-coordinates	y-coordinates
1	775	1550
1	775	1600
1	775	1650
1	775	1700
1	775	1850
1	775	1900
1	775	1950
1	775	2000
1	775	2050
1	775	2100
1	775	2150
1	775	2200
1	775	2250
1	775	2300
1	775	2350
1	300	1200
1	300	1250
1	300	1300
1	300	1350
1	300	1400
1	300	1450
1	300	1500
1	300	1550
1	300	1600
1	300	1650
1	300	1900
1	300	1950
1	300	2000
1	300	2050
1	300	2100
1	300	2150
1	300	2200
1	300	2250
1	300	2300
1	300	2350
1	300	2400
1	325	1150
1	325	1200
1	325	1250
1	325	1300
1	325	1350
1	325	1400
1	325	1450
1	325	1500
1	325	1550
1	325	1600
1	325	1650
1	325	1700
1	325	1850

Cluster #	x-coordinates	y-coordinates
1	325	1900
1	325	1950
1	325	2000
1	325	2050
1	325	2100
1	325	2150
1	325	2200
1	325	2250
1	325	2300
1	325	2350
1	325	2400
1	325	2450
1	350	1100
1	350	1150
1	350	2450
1	350	2500
1	375	1050
1	375	1100
1	375	2500
1	375	2550
1	400	1000
1	400	1050
1	400	2550
1	400	2600
1	425	950
1	425	1000
1	425	2600
1	425	2650
1	450	900
1	450	950
1	450	1000
1	450	2600
1	450	2650
1	450	2700
1	475	850
1	475	900
1	475	950
1	475	2650
1	475	2700
1	500	850
1	500	900
1	500	2550
1	500	2700
1	525	850
1	525	900
1	525	2550
1	525	2700
1	550	850
1	550	900

Cluster #	x-coordinates	y-coordinates
1	550	2550
1	550	2700
1	575	850
1	575	900
1	575	2550
1	575	2700
1	600	900
1	600	950
1	600	2650
1	600	2700
1	625	950
1	625	1000
1	625	2600
1	625	2650
1	650	950
1	650	1000
1	650	2450
1	650	2600
1	675	1000
1	675	1050
1	675	2400
1	675	2550
1	675	2600
1	700	1050
1	700	1100
1	700	2500
1	700	2550
1	725	1100
1	725	1150
1	725	2450
1	725	2500
1	750	1150
1	750	1200
1	750	1250
1	750	2350
1	750	2400
1	750	2450
1	775	1200
1	775	1250
1	775	1300
1	775	2400
1	800	1250
1	800	1300
1	800	1350
1	800	1400
1	800	1450
1	800	1500
1	800	1550
1	800	1600

Cluster #	x-coordinates	y-coordinates
1	800	1650
1	800	1900
1	800	1950
1	800	2000
1	800	2050
1	800	2100
1	800	2150
1	800	2200
1	800	2250
1	800	2300
1	800	2350
1	825	1300
1	825	1350
1	825	1400
1	825	1450
1	825	1500
1	825	1550
1	825	1600
1	825	1950
1	825	2000
1	825	2050
1	825	2100
1	825	2150
1	825	2200
1	825	2250
1	825	2300
1	300	1000
1	300	1050
1	300	1100
1	300	1150
1	300	2450
1	300	2500
1	300	2550
1	325	950
1	325	1000
1	325	1050
1	325	1100
1	325	2500
1	325	2550
1	325	2600
1	350	900
1	350	950
1	350	1000
1	350	1050
1	350	2550
1	350	2600
1	350	2650
1	375	850
1	375	900

Cluster #	x-coordinates	y-coordinates
1	375	950
1	375	1000
1	375	2600
1	375	2650
1	375	2700
1	400	800
1	400	850
1	400	900
1	400	950
1	400	2650
1	400	2700
1	400	2750
1	425	800
1	425	850
1	425	900
1	425	2700
1	425	2750
1	450	800
1	450	850
1	450	2750
1	475	800
1	475	2750
1	500	800
1	500	2750
1	525	800
1	525	2750
1	550	800
1	550	2750
1	575	800
1	575	2750
1	600	800
1	600	850
1	600	2750
1	625	800
1	625	850
1	625	900
1	625	2700
1	625	2750
1	650	800
1	650	850
1	650	900
1	650	2650
1	650	2700
1	650	2750
1	675	800
1	675	850
1	675	900
1	675	950
1	675	2650

Cluster #	x-coordinates	y-coordinates
1	675	2700
1	675	2750
1	700	800
1	700	850
1	700	900
1	700	950
1	700	1000
1	700	2600
1	700	2650
1	700	2700
1	700	2750
1	725	850
1	725	900
1	725	950
1	725	1000
1	725	1050
1	725	2550
1	725	2600
1	725	2650
1	725	2700
1	750	900
1	750	950
1	750	1000
1	750	1050
1	750	1100
1	750	2500
1	750	2550
1	750	2600
1	750	2650
1	775	950
1	775	1000
1	775	1050
1	775	1100
1	775	1150
1	775	2450
1	775	2500
1	775	2550
1	775	2600
1	800	1000
1	800	1050
1	800	1100
1	800	1150
1	800	1200
1	800	2400
1	800	2450
1	800	2500
1	800	2550
1	825	1050
1	825	1100

Cluster #	x-coordinates	y-coordinates
1	825	1150
1	825	1200
1	825	1250
1	825	2350
1	825	2400
1	825	2450
1	825	2500
1	825	1700
1	800	1700
1	825	1750
1	800	1750
1	775	1750
1	825	1650
1	825	1900
1	800	1800
1	775	1800
1	825	1800
1	825	1850
1	800	1850
1	300	1850
1	325	1800
1	300	1800
1	325	1750
1	300	1750
1	300	1700
2	475	1400
2	475	1450
2	500	1350
2	500	1400
2	500	1450
2	500	1500
2	525	1350
2	525	1400
2	525	1450
2	525	1500
2	525	1550
2	525	1300
2	550	1250
2	550	1300
2	550	1350
2	550	1400
2	550	1450
2	550	1500
2	575	1300
2	575	1350
2	575	1400
2	575	1450
2	575	1500
2	550	1600

Cluster #	x-coordinates	y-coordinates
2	550	1550
2	575	1550
2	600	1350
2	600	1400
2	600	1450
2	600	1500
2	625	1400
2	625	1450
2	650	1450
3	475	1900
3	475	1950
3	475	2000
3	475	2050
3	475	2100
3	475	2150
3	475	2200
3	475	2250
3	500	1900
3	500	1950
3	500	2000
3	500	2050
3	500	2100
3	500	2150
3	500	2200
3	500	2250
3	525	1900
3	525	1950
3	525	2000
3	525	2050
3	525	2100
3	525	2150
3	525	2200
3	525	2250
3	550	1900
3	550	1950
3	550	2000
3	550	2050
3	550	2100
3	550	2150
3	550	2200
3	550	2250
3	575	1900
3	575	1950
3	575	2000
3	575	2050
3	575	2100
3	575	2150
3	575	2200
3	575	2250

Cluster #	x-coordinates	y-coordinates
3	600	1900
3	600	1950
3	600	2000
3	600	2050
3	600	2100
3	600	2150
3	600	2200
3	600	2250
3	625	1900
3	625	1950
3	625	2000
3	625	2050
3	625	2100
3	625	2150
3	625	2200
3	625	2250
3	650	1900
3	650	1950
3	650	2000
3	650	2050
3	650	2100
3	650	2150
3	650	2200
3	650	2250

Appendix G

Pat2
Number of dimensions: 2
Number of clusters: 2
Number of points: 417

Cluster #	x-coordinates	y-coordinates
1	300	1400
1	300	1450
1	300	1500
1	300	1550
1	300	1600
1	300	1650
1	300	1700
1	300	1750
1	300	1800
1	300	1850
1	300	1900
1	300	1950
1	300	2000
1	300	2050
1	300	2100
1	300	2150
1	300	2200
1	300	2250
1	300	2300
1	300	2350
1	300	2400
1	300	2450
1	300	2500
1	300	2550
1	300	2600
1	325	1400
1	325	1450
1	325	1500
1	325	1550
1	325	1600
1	325	1650
1	325	1700
1	325	1750
1	325	1800
1	325	1850
1	325	1900
1	325	1950
1	325	2000
1	325	2050
1	325	2100
1	325	2150
1	325	2200
1	325	2250

Cluster #	x-coordinates	y-coordinates
1	325	2300
1	325	2350
1	325	2400
1	325	2450
1	325	2500
1	325	2550
1	325	2600
1	325	2650
1	350	1400
1	350	1450
1	350	1500
1	350	1550
1	350	1600
1	350	1650
1	350	1700
1	350	1750
1	350	1800
1	350	1850
1	350	1900
1	350	1950
1	350	2000
1	350	2050
1	350	2100
1	350	2150
1	350	2200
1	350	2250
1	350	2300
1	350	2350
1	350	2400
1	350	2450
1	350	2500
1	350	2550
1	350	2600
1	350	2650
1	350	2700
1	375	2500
1	375	2550
1	375	2600
1	375	2650
1	375	2700
1	375	2750
1	400	2550
1	400	2600
1	400	2650
1	400	2700
1	400	2750
1	425	2600
1	425	2650
1	425	2700

Cluster #	x-coordinates	y-coordinates
1	425	2750
1	450	2600
1	450	2650
1	450	2700
1	450	2750
1	475	2550
1	475	2600
1	475	2650
1	475	2700
1	475	2750
1	500	2600
1	500	2650
1	500	2700
1	500	2750
1	525	2600
1	525	2650
1	525	2700
1	525	2750
1	550	2550
1	550	2600
1	550	2650
1	550	2700
1	550	2750
1	575	2500
1	575	2550
1	575	2600
1	575	2650
1	575	2700
1	575	2750
1	600	1400
1	600	1450
1	600	1500
1	600	1550
1	600	1600
1	600	1650
1	600	1700
1	600	1750
1	600	1800
1	600	1850
1	600	1900
1	600	1950
1	600	2000
1	600	2050
1	600	2100
1	600	2150
1	600	2200
1	600	2250
1	600	2300
1	600	2350

Cluster #	x-coordinates	y-coordinates
1	600	2400
1	600	2450
1	600	2500
1	600	2550
1	600	2600
1	600	2650
1	600	2700
1	625	1400
1	625	1450
1	625	1500
1	625	1550
1	625	1600
1	625	1650
1	625	1700
1	625	1750
1	625	1800
1	625	1850
1	625	1900
1	625	1950
1	625	2000
1	625	2050
1	625	2100
1	625	2150
1	625	2200
1	625	2250
1	625	2300
1	625	2350
1	625	2400
1	625	2450
1	625	2500
1	625	2550
1	625	2600
1	625	2650
1	650	1400
1	650	1450
1	650	1500
1	650	1550
1	650	1600
1	650	1650
1	650	1700
1	650	1750
1	650	1800
1	650	1850
1	650	1900
1	650	1950
1	650	2000
1	650	2050
1	650	2100
1	650	2150

Cluster #	x-coordinates	y-coordinates
1	650	2200
1	650	2250
1	650	2300
1	650	2350
1	650	2400
1	650	2450
1	650	2500
1	650	2550
1	650	2600
1	675	1400
1	675	1450
1	675	1500
1	675	1550
1	675	1600
1	675	1650
1	675	1700
1	675	1750
1	675	1800
1	675	1850
1	675	1900
1	675	1950
1	675	2000
1	675	2050
1	675	2100
1	675	2150
1	675	2200
1	675	2250
1	675	2300
1	675	2350
1	675	2400
1	675	2450
1	675	2500
1	675	2550
2	450	1000
2	450	1050
2	450	1100
2	450	1150
2	450	1200
2	450	1250
2	450	1300
2	450	1350
2	450	1400
2	450	1450
2	450	1500
2	450	1550
2	450	1600
2	450	1650
2	450	1700
2	450	1750

Cluster #	x-coordinates	y-coordinates
2	450	1800
2	450	1850
2	450	1900
2	450	1950
2	450	2000
2	450	2050
2	450	2100
2	450	2150
2	475	950
2	475	1000
2	475	1050
2	475	1100
2	475	1150
2	475	1200
2	475	1250
2	475	1300
2	475	1350
2	475	1400
2	475	1450
2	475	1500
2	475	1550
2	475	1600
2	475	1650
2	475	1700
2	475	1750
2	475	1800
2	475	1850
2	475	1900
2	475	1950
2	475	2000
2	475	2050
2	475	2100
2	475	2150
2	500	900
2	500	950
2	500	1000
2	500	1050
2	500	1100
2	500	1150
2	500	1200
2	500	1250
2	500	1300
2	500	1350
2	500	1400
2	500	1450
2	500	1500
2	500	1550
2	500	1600
2	500	1650

Cluster #	x-coordinates	y-coordinates
2	500	1700
2	500	1750
2	500	1800
2	500	1850
2	500	1900
2	500	1950
2	500	2000
2	500	2050
2	500	2100
2	500	2150
2	525	850
2	525	900
2	525	950
2	525	1000
2	525	1050
2	550	800
2	550	850
2	550	900
2	550	950
2	550	1000
2	575	800
2	575	850
2	575	900
2	575	950
2	600	800
2	600	850
2	600	900
2	600	950
2	625	800
2	625	850
2	625	900
2	625	950
2	650	800
2	650	850
2	650	900
2	650	950
2	675	800
2	675	850
2	675	900
2	675	950
2	700	800
2	700	850
2	700	900
2	700	950
2	725	800
2	725	850
2	725	900
2	725	950
2	725	1000

Cluster #	x-coordinates	y-coordinates
2	750	850
2	750	900
2	750	950
2	750	1000
2	750	1050
2	775	900
2	775	950
2	775	1000
2	775	1050
2	775	1100
2	775	1150
2	775	1200
2	775	1250
2	775	1300
2	775	1350
2	775	1400
2	775	1450
2	775	1500
2	775	1550
2	775	1600
2	775	1650
2	775	1700
2	775	1750
2	775	1800
2	775	1850
2	775	1900
2	775	1950
2	775	2000
2	775	2050
2	775	2100
2	775	2150
2	800	950
2	800	1000
2	800	1050
2	800	1100
2	800	1150
2	800	1200
2	800	1250
2	800	1300
2	800	1350
2	800	1400
2	800	1450
2	800	1500
2	800	1550
2	800	1600
2	800	1650
2	800	1700
2	800	1750
2	800	1800

Cluster #	x-coordinates	y-coordinates
2	800	1850
2	800	1900
2	800	1950
2	800	2000
2	800	2050
2	800	2100
2	800	2150
2	825	1000
2	825	1050
2	825	1100
2	825	1150
2	825	1200
2	825	1250
2	825	1300
2	825	1350
2	825	1400
2	825	1450
2	825	1500
2	825	1550
2	825	1600
2	825	1650
2	825	1700
2	825	1750
2	825	1800
2	825	1850
2	825	1900
2	825	1950
2	825	2000
2	825	2050
2	825	2100
2	825	2150

Appendix H

Long1
Number of dimensions: 2
Number of clusters: 2
Number of points: 1000

Cluster #	x-coordinates	y-coordinates
1	0.253393	-0.00455816
1	0.83422	-0.0170388
1	0.915511	0.0706192
1	-1.05354	-0.00893493
1	-2.50606	0.2123
1	-0.157464	-0.0904913
1	-0.418497	-0.0761906
1	-0.136168	-0.0201592
1	-0.575993	0.0578409
1	-1.1775	-0.0551915
1	-0.224303	-0.0583399
1	0.879351	-0.0101605
1	-1.02454	0.293514
1	-1.09982	-0.0803789
1	0.242494	0.0304474
1	0.537456	-0.102094
1	1.67739	0.0600952
1	0.369955	0.225182
1	-0.121237	-0.0645134
1	-0.0847689	-0.0242522
1	-0.278211	-0.172555
1	-1.12848	0.0348156
1	-1.78313	-0.0476398
1	0.655194	-0.0558817
1	0.102952	-0.0935296
1	-1.86185	0.103667
1	1.40307	0.0332311
1	0.367027	0.0304474
1	0.348978	0.0477442
1	2.17934	0.0414917
1	0.927468	-0.0504754
1	-0.49395	-0.0682821
1	-0.255293	0.00225204
1	-1.43054	-0.164422
1	0.140132	0.122472
1	-0.875483	-0.0673006
1	-0.960479	-0.124474
1	-0.197906	-0.0326616
1	-0.676833	-0.117611

Cluster #	x-coordinates	y-coordinates
1	-0.820069	-0.110358
1	-0.353938	-0.128607
1	-0.714948	0.124348
1	-1.60032	0.0318294
1	0.712087	0.0460459
1	-0.732889	-0.0978885
1	0.0110781	-0.0154536
1	0.623428	-0.0964768
1	-0.299723	-0.0170185
1	-1.09003	-0.0599307
1	0.0962752	0.0357661
1	-0.54971	-0.0733061
1	-2.07216	0.171066
1	0.776518	-0.155619
1	1.31809	0.000322443
1	-0.879192	0.0255043
1	-0.944506	0.0640793
1	1.14447	0.0897981
1	-0.254995	-0.0312697
1	1.12886	-0.0401826
1	1.18115	0.040071
1	1.08215	-0.250612
1	0.339078	-0.136568
1	-0.997227	0.0330465
1	1.21921	0.0679839
1	0.583223	0.043173
1	0.340609	-0.0232696
1	0.0890815	0.0817915
1	0.874991	-0.031882
1	1.74955	-0.170421
1	-1.54689	0.0504144
1	0.426429	0.114779
1	0.505981	-0.185967
1	-0.0093805	0.146773
1	-0.201329	0.0417604
1	-0.82331	0.101594
1	1.00688	-0.311822
1	-0.722829	-0.126479
1	0.5747	-0.123048
1	-0.993967	4.77E-06
1	-0.00613301	-0.0211489
1	1.08883	0.00352096
1	1.07069	0.0379328
1	0.331842	-0.100362
1	-0.675418	0.0520742

Cluster #	x-coordinates	y-coordinates
1	-0.0937344	-0.0581621
1	-0.356825	0.0314091
1	-1.59416	-0.109356
1	-0.495751	0.0911588
1	-0.352381	-0.0896015
1	-1.75406	-0.137437
1	-1.53517	-0.0431254
1	-3.12948	0.0956477
1	-2.08666	0.0830681
1	-1.07235	-0.0968007
1	-1.0827	-0.00533056
1	-0.306367	0.0380407
1	0.825309	0.0749938
1	-0.827341	0.179749
1	-1.8258	-0.151838
1	-2.16512	-0.172181
1	2.66683	0.122222
1	0.151253	0.0638212
1	1.86715	0.000160533
1	0.0897607	0.134399
1	0.568813	-0.108111
1	-0.272864	0.0119195
1	0.329131	-0.0987918
1	-0.264881	0.00984813
1	-0.57571	0.0741154
1	-1.11533	0.130916
1	1.21768	-0.0797715
1	1.22179	0.0708551
1	-0.102856	0.132825
1	-1.39574	0.00434462
1	0.952595	0.0137998
1	-0.301796	0.0270658
1	1.05373	-0.0243458
1	-1.00729	-0.0666343
1	0.275425	-0.0743785
1	0.389594	-0.165659
1	0.0577835	-0.0521531
1	-0.84928	-0.0529002
1	0.562663	0.153429
1	0.325923	-0.0636706
1	0.160118	0.0370412
1	0.212422	0.107921
1	-1.54003	0.101316
1	1.47052	-0.05743
1	1.20684	-0.177299

Cluster #	x-coordinates	y-coordinates
1	-0.685078	0.0941776
1	0.117483	-0.169263
1	-2.4859	-0.178404
1	-1.15635	0.174864
1	1.20139	0.1378
1	-0.186969	0.0106851
1	0.621751	-0.202081
1	-1.38056	0.0733333
1	0.184596	-0.162484
1	-1.04	0.0463194
1	0.217772	0.0924579
1	-1.879	-0.152589
1	1.55626	0.13825
1	1.2442	0.0875042
1	-0.9399	0.122179
1	-0.580477	-0.0254866
1	-0.469324	0.0168792
1	-0.0277151	-0.135809
1	-0.59108	-0.232661
1	-0.27386	0.0422657
1	-1.85156	0.0765116
1	0.438771	-0.0931332
1	-0.953326	-0.0320126
1	1.11771	0.0431919
1	1.03534	-0.12861
1	0.102172	0.0620459
1	1.39031	-0.00690002
1	-0.358635	-0.0876824
1	0.767109	-0.0682202
1	-1.28805	-0.130789
1	0.8594	0.0991523
1	-0.525072	-0.13193
1	-0.204826	-0.0415838
1	0.73635	-0.0556326
1	0.914653	-0.263309
1	0.237551	0.0037076
1	1.04343	-0.0362584
1	0.341256	0.0492251
1	1.07892	-0.137839
1	0.0941037	0.0190803
1	-1.17668	0.0532943
1	-0.746271	0.00118665
1	-0.351828	0.0688129
1	-0.357883	-0.0964897
1	-0.654513	-0.142691

Cluster #	x-coordinates	y-coordinates
1	0.359708	0.107496
1	-0.713128	0.182556
1	1.30875	-0.00892225
1	0.520836	0.0839293
1	0.974391	0.0166899
1	0.774532	-0.187296
1	-0.787541	-0.0013662
1	0.900451	-0.160824
1	-0.299352	-0.0530108
1	-1.99578	-0.0782936
1	0.300498	-0.000396791
1	0.135372	-0.119167
1	-0.459734	-0.0760756
1	0.730423	0.0567894
1	-0.336895	-0.123597
1	0.27565	0.0475124
1	1.30017	0.0965536
1	0.773866	-0.0526884
1	0.195621	-0.103851
1	1.46322	-0.128414
1	-0.278447	0.0305865
1	0.645301	0.204664
1	-0.242643	-0.0718841
1	-1.30302	-0.0921394
1	0.180161	0.106317
1	-0.268031	-0.135043
1	0.0206855	-0.320913
1	0.408971	-0.0829172
1	-0.725392	0.0911762
1	0.988901	-0.158084
1	-0.422991	0.00688121
1	-0.283412	0.112527
1	0.0200999	0.10516
1	-1.66162	-0.0273628
1	1.8675	-0.0797977
1	-0.758317	0.103431
1	-0.453906	0.0673762
1	-0.12443	-0.0650107
1	-1.24093	-0.0127123
1	-0.918873	0.0671979
1	-1.3265	0.0124903
1	1.1757	-0.0210545
1	-0.069587	0.119859
1	0.850813	-0.0237574
1	-1.75606	0.0336788

Cluster #	x-coordinates	y-coordinates
1	0.968171	0.154858
1	0.292139	0.064167
1	1.06734	-0.248946
1	-0.117305	-0.0846088
1	2.79565	-0.12848
1	-0.552581	0.047418
1	0.0981731	-0.0560555
1	0.990402	-0.0713737
1	1.6486	-0.136905
1	-1.86339	-0.0310404
1	0.738282	-0.0292273
1	0.546452	0.192226
1	-0.0296005	0.161716
1	0.909412	-0.0637077
1	0.724999	0.123055
1	0.369682	0.123806
1	-0.232445	0.0681278
1	-0.133887	-0.00783047
1	0.464595	0.0561168
1	-1.56575	-0.0059767
1	1.09111	-0.0257051
1	-0.449422	0.0507276
1	0.187693	-0.0998503
1	-0.506888	-0.0143663
1	-0.359103	0.165263
1	0.984147	0.262128
1	0.00185955	0.0443587
1	-0.202741	-0.0586593
1	1.49313	-0.0152605
1	0.72016	-0.00490566
1	0.0691956	0.0748195
1	-0.167286	-0.0440802
1	-0.361046	0.156682
1	-1.32383	-0.0432046
1	-0.838184	0.0334978
1	1.02077	0.138825
1	-0.0688815	0.00703031
1	-0.945595	-0.0412386
1	0.755842	-0.0444767
1	1.11491	0.0581969
1	0.198919	0.0507321
1	0.699227	0.138317
1	0.810437	-0.0688753
1	-2.17603	-0.0276394
1	-0.869672	0.0682986

Cluster #	x-coordinates	y-coordinates
1	1.59185	-0.00829585
1	-0.23192	0.126741
1	-0.049774	0.076993
1	0.587074	-0.0212265
1	-0.572329	-0.169171
1	-1.39988	0.0341072
1	0.699529	0.0860492
1	0.694106	-0.0523796
1	-0.75516	-0.0237618
1	-0.277008	-0.0582489
1	0.712068	-0.118136
1	-0.528612	-0.0633473
1	-0.838062	-0.0817071
1	0.591955	-0.114341
1	-0.258245	0.0254778
1	-0.676164	0.0357927
1	0.155266	-0.0399413
1	-0.64096	-0.0125477
1	0.828332	0.0230323
1	-1.29338	-0.0250834
1	1.53288	0.00465289
1	-1.0588	0.0223981
1	0.765537	-0.0260314
1	1.26496	-0.0325155
1	-0.0262231	-0.0311237
1	0.0140755	0.180096
1	-0.99463	-0.0443944
1	-1.0945	0.0642885
1	-2.32988	-0.159325
1	0.691015	-0.161351
1	-1.79349	0.0480905
1	-1.79347	0.0121053
1	-0.611991	-0.0975015
1	0.42266	-0.0125173
1	-0.0590084	-0.130244
1	0.85369	-0.0310634
1	-0.134584	-0.0836553
1	1.36748	-0.166478
1	0.138626	-0.0815791
1	-0.856811	0.0366416
1	-0.229612	0.00508039
1	-0.357802	-0.0919612
1	-1.72534	-0.00249044
1	0.536381	0.0118484
1	-0.397013	0.120251

Cluster #	x-coordinates	y-coordinates
1	0.306403	0.122557
1	-0.77352	-0.155714
1	-3.01335	-0.123307
1	-0.0778479	0.208337
1	-0.80387	0.0139228
1	0.634893	-0.102391
1	-1.42741	0.0425692
1	-1.9772	0.106443
1	-1.60405	-0.11274
1	-2.28709	0.0138101
1	-0.459056	-0.0149183
1	0.847456	-0.138576
1	-0.519938	0.127489
1	0.966368	-0.0910562
1	0.338336	-0.0943031
1	0.558323	-0.017247
1	-1.53465	0.0670213
1	-2.2683	-0.0897967
1	-1.01049	0.176127
1	-0.988123	0.042354
1	1.26109	-0.00452123
1	1.51279	0.116683
1	-1.40209	0.115918
1	0.567598	0.00196185
1	1.30455	-0.075966
1	0.213999	-0.0824407
1	-0.344547	-0.013963
1	-0.409211	0.131968
1	0.987903	-0.0973073
1	0.486531	0.0764712
1	0.215283	0.0949274
1	-1.09524	0.0485825
1	0.203506	-0.096427
1	-1.12884	0.0207871
1	-1.70888	-0.0153499
1	-0.708086	-0.0294642
1	0.0140835	-0.0349754
1	1.02813	0.081929
1	0.0508634	0.023718
1	-0.00752674	0.103911
1	-0.634701	-0.12478
1	1.30529	0.0449888
1	0.201518	-0.0582813
1	-1.45402	0.153769
1	0.424004	-0.111945

Cluster #	x-coordinates	y-coordinates
1	-0.603592	0.139028
1	0.868067	-0.0454448
1	-0.625304	0.117827
1	-0.460233	0.0112467
1	1.32969	-0.108464
1	-0.439339	-0.0328808
1	-0.785188	-0.0763487
1	-0.147583	0.0276326
1	0.620501	-0.119919
1	-1.75183	-0.0451318
1	-2.00476	-0.000851254
1	0.425036	0.0631683
1	0.902	-0.0454373
1	-0.73582	-0.0446116
1	-1.32409	-0.0137508
1	-1.1655	-0.156684
1	1.17721	-0.081374
1	0.331858	0.0601991
1	-0.262941	-0.0171583
1	-0.262621	0.114578
1	-0.45878	-0.0794879
1	0.000281957	0.00492265
1	-0.589094	-0.0729572
1	0.1849	-0.0815576
1	-0.59227	0.16349
1	0.38639	-0.112301
1	-1.96601	0.0181512
1	-0.306279	0.113441
1	0.384221	-0.0952002
1	0.881756	-0.0922673
1	-1.00457	-0.0650769
1	1.8618	-0.0334596
1	-0.631212	0.0916184
1	1.35506	0.124252
1	1.40532	0.0657252
1	-0.122576	-0.0539021
1	-0.963082	0.0249623
1	-0.447111	0.0859273
1	0.0220329	-0.035294
1	-1.54171	-0.0429167
1	-2.78248	-0.113485
1	-2.53579	-0.0568664
1	-0.738674	0.0764642
1	1.18048	-0.016686
1	-0.199546	0.0193324

Cluster #	x-coordinates	y-coordinates
1	0.148807	-0.167221
1	0.17771	0.0978593
1	0.215096	0.113637
1	0.686159	-0.026765
1	-1.13569	0.112318
1	0.967341	-0.0879007
1	0.481453	0.306953
1	-2.32793	-0.00501965
1	-0.565795	-0.0109406
1	0.0737335	-0.0968202
1	1.16113	-0.000288949
1	1.14108	-0.0961267
1	0.305677	-0.0220936
1	0.288798	-0.102813
1	1.1221	0.0434623
1	0.516391	0.0910805
1	0.369052	-0.041685
1	-0.113167	0.0228592
1	-0.728676	0.0655107
1	0.581779	0.0398188
1	-1.41234	-0.0271456
1	-0.164629	0.0884568
1	0.0623831	0.0882274
1	0.347602	-0.0541812
1	-0.423101	0.027566
1	1.24389	0.196143
1	-1.23479	0.155197
1	-0.174855	0.209935
1	0.817342	0.0547912
1	0.567539	-0.0536422
1	-1.43932	-0.049941
1	-0.768826	0.0691513
1	0.881405	-0.0186069
1	-1.40302	-0.117873
1	-0.449823	-0.0070121
1	0.640689	-0.0718497
1	-1.38798	0.0733836
1	-0.951926	-0.00773655
1	-0.19147	0.194404
1	0.455673	0.0890925
1	0.548244	-0.12319
1	0.120971	0.056637
1	-0.751335	-0.127966
1	0.998591	-0.12753
1	0.620489	-0.0644643

Cluster #	x-coordinates	y-coordinates
1	-0.126826	-0.0447342
1	-0.772287	0.0342627
1	-0.307475	-0.0362906
1	1.05263	-0.00481072
1	-1.11586	0.108007
1	0.212303	0.0663939
1	-0.126013	0.00556105
1	-0.967442	-0.145505
1	0.847412	0.125246
1	-0.346808	-0.0104266
1	0.460423	-0.0401593
1	-1.44474	0.101415
1	0.246355	0.0531447
1	0.549585	-0.0776353
1	0.0323727	0.049237
1	0.151058	-0.0396377
1	-1.18356	0.179834
1	0.939979	-0.0454258
1	-1.46681	0.100495
1	0.995585	0.0106852
1	0.331556	-0.0976029
1	-0.464794	0.075982
1	-0.319692	-0.0512594
1	0.271096	0.22319
1	0.864347	0.00894678
1	-1.09879	0.177687
1	0.329608	-0.0363142
1	0.23368	0.314206
1	0.190379	0.0624598
1	-1.21975	-0.0762002
1	-0.92923	0.0392282
1	-0.548827	-0.218254
1	-1.69403	0.0498714
1	0.355805	-0.0296963
1	-0.723436	-0.00333439
1	-0.637213	0.0652832
1	-1.33217	0.0186277
1	-0.959765	-0.127227
1	0.550095	-0.0562966
1	0.692108	0.118421
1	-0.428851	0.0699166
1	-0.678973	0.0385305
1	0.344638	-0.101012
1	-0.0966308	-0.0626256
1	0.0634229	0.164655

Cluster #	x-coordinates	y-coordinates
1	-1.28008	0.034525
1	0.566191	-0.0382371
1	0.510395	0.109033
1	0.77937	0.0208194
1	-1.14343	0.0357556
1	0.0098053	-0.0937676
1	-0.207299	0.0320399
1	-1.12723	0.0786312
1	1.29173	-0.0651792
1	1.46509	0.130104
1	0.964691	-0.195421
2	-0.225065	0.952591
2	-0.525512	0.994807
2	1.31474	1.03657
2	-1.19313	1.27061
2	1.17326	1.04394
2	1.89282	1.08011
2	-0.0584518	0.918237
2	-0.206354	1.12443
2	2.7839	1.00077
2	-0.471401	0.942948
2	0.810235	1.31397
2	-0.518959	0.890253
2	0.819598	1.0417
2	-0.428873	1.12442
2	-1.14183	0.990503
2	-0.93828	0.99698
2	-0.201319	1.12736
2	-0.818975	0.973572
2	1.27065	0.846908
2	-0.155381	0.744029
2	-0.384263	0.889444
2	-0.386252	1.02348
2	-2.17007	0.902337
2	-0.684899	1.05715
2	0.57951	1.31339
2	-0.645793	1.23397
2	-0.905925	1.05187
2	-1.24901	1.03932
2	-0.326698	1.04779
2	-0.327617	0.98353
2	-0.58669	1.06067
2	1.42038	1.07271
2	0.882753	0.914946
2	-1.07902	0.841175

Cluster #	x-coordinates	y-coordinates
2	0.245255	1.15651
2	0.644159	1.06103
2	-0.702138	1.10755
2	-0.150024	1.06389
2	-0.6071	1.11036
2	-0.952138	1.02638
2	-0.682304	0.933249
2	0.562172	0.790533
2	-0.185441	0.821707
2	-0.656368	1.08779
2	0.661945	1.03102
2	-0.209827	1.18868
2	1.16975	0.964249
2	0.182033	0.849631
2	-0.0929888	1.12689
2	2.53173	1.00378
2	1.10284	1.19939
2	-0.838247	1.2186
2	2.00603	0.935767
2	0.448058	0.963035
2	-0.340341	1.04682
2	-0.960609	1.00861
2	0.172788	0.988685
2	0.0562029	1.0815
2	1.14715	0.917909
2	0.169496	0.970796
2	-1.18551	1.07968
2	0.437965	0.956517
2	0.928713	0.877822
2	-0.370856	0.980794
2	3	1.01981
2	0.840556	0.873698
2	0.679313	1.02241
2	-2.06855	0.97721
2	-0.243372	0.970767
2	-2.2947	1.14037
2	0.636475	1.07279
2	1.28511	1.11928
2	-0.39154	1.02821
2	1.36352	0.850488
2	0.195416	0.910011
2	0.601425	0.910279
2	0.306591	1.04504
2	1.85537	1.05563
2	-0.23102	0.980282

Cluster #	x-coordinates	y-coordinates
2	-0.36312	0.848385
2	-2.28454	0.890744
2	0.959982	0.9618
2	-0.442694	1.06529
2	1.03791	0.933194
2	0.843385	0.964811
2	-2.00473	0.964415
2	1.02767	0.805296
2	1.16609	0.761276
2	1.10594	0.892854
2	-0.143281	1.06282
2	-1.3508	1.0113
2	-1.54628	0.966339
2	1.80291	1.00527
2	0.0863198	0.833785
2	-0.428175	1.07292
2	0.00484389	0.898121
2	0.24753	0.857675
2	-0.0983201	1.0578
2	-0.94448	0.947899
2	0.16033	0.928858
2	-0.310212	0.998177
2	-0.0360567	1.05212
2	-1.49791	0.995386
2	-0.965493	1.13015
2	0.268153	0.959895
2	-0.769875	1.06411
2	0.597909	1.14813
2	0.407347	1.17268
2	-2.18495	0.956116
2	1.70381	1.08984
2	0.0823503	1.01707
2	-0.489914	1.05616
2	0.505071	0.983115
2	0.164695	0.965002
2	-0.863467	0.737702
2	0.464145	1.04084
2	-0.48489	1.06497
2	-0.541103	0.998512
2	1.19413	1.04853
2	1.03702	1.04445
2	-1.90131	1.14714
2	-0.649432	0.979814
2	-1.4849	1.10715
2	1.09761	1.02714

Cluster #	x-coordinates	y-coordinates
2	1.57242	0.811878
2	-0.0375146	1.11209
2	-0.773557	0.98219
2	0.189688	0.866304
2	0.434248	0.868667
2	1.22573	1.02805
2	-0.382632	1.02384
2	-0.237543	0.914989
2	-0.40782	1.15482
2	-0.0716617	1.13205
2	-0.952155	0.976694
2	1.31	0.957192
2	-0.430796	1.0686
2	1.61032	1.20354
2	-1.94552	1.11499
2	0.560726	1.02994
2	-1.17763	0.926072
2	-0.851006	0.939135
2	0.082557	1.08566
2	-1.43922	0.932747
2	1.13043	1.03877
2	0.698677	0.961357
2	0.815791	0.943406
2	1.52631	1.05623
2	0.0267234	1.06971
2	0.268485	1.00002
2	-0.34227	1.23294
2	2.31716	0.808588
2	-0.095591	1.10632
2	0.754661	0.911943
2	-1.10283	1.08566
2	2.30458	0.917452
2	0.237516	1.00449
2	1.47763	1.02854
2	0.220982	0.952279
2	-0.33646	0.933159
2	-0.511867	0.940731
2	0.0463534	0.985809
2	1.79215	1.14234
2	-0.453992	1.02021
2	1.80626	0.949023
2	-1.40107	0.964577
2	0.583139	1.05779
2	-1.15134	1.08036
2	0.412703	1.02723

Cluster #	x-coordinates	y-coordinates
2	-1.09276	0.940511
2	2.05023	1.05517
2	0.734214	0.854285
2	-1.31912	1.22092
2	-1.01558	1.00591
2	-1.58143	1.16748
2	0.703077	0.982845
2	0.707452	1.00866
2	-1.05038	1.03226
2	-0.327033	0.941949
2	-0.174112	0.893715
2	0.733221	0.958469
2	-0.830409	0.806626
2	2.41161	1.1183
2	0.948567	1.06135
2	-1.30291	0.802583
2	-2.21429	1.12426
2	0.461304	0.939394
2	0.155715	0.919014
2	0.553046	1.1504
2	1.82353	1.09421
2	-1.7749	0.936412
2	-0.421206	0.995861
2	-2.12898	1.01501
2	-0.994713	0.946629
2	0.808128	1.0257
2	-0.965132	0.787146
2	-0.73767	0.987242
2	-0.163227	0.795051
2	-0.558453	1.0408
2	0.322577	1.07975
2	1.22073	0.91903
2	-1.72872	0.943839
2	1.19047	1.16518
2	-0.439273	1.06688
2	0.563583	1.02678
2	-1.13509	1.08532
2	1.78918	1.00707
2	0.20267	0.967043
2	1.95683	0.994624
2	0.70396	1.08403
2	0.837075	1.03973
2	-0.291008	1.04201
2	1.66171	1.0378
2	-1.21834	0.914911

Cluster #	x-coordinates	y-coordinates
2	0.395834	0.97553
2	-0.599598	1.04926
2	0.652113	0.836563
2	1.26047	1.05068
2	1.85236	0.787976
2	1.21951	1.0473
2	0.527306	1.10527
2	0.937461	0.932491
2	0.507143	1.19355
2	-1.64337	1.06727
2	1.02878	0.968442
2	0.889301	1.08671
2	-0.875443	0.977285
2	-0.546873	0.839869
2	0.0523095	1.09339
2	0.565427	0.887904
2	-1.08839	1.02561
2	-1.5882	1.15392
2	0.930282	0.983624
2	-0.147351	1.08001
2	0.60933	0.88462
2	-1.02411	1.05439
2	-1.27772	0.982593
2	-0.483289	0.768504
2	1.55483	1.02722
2	0.545234	0.908423
2	0.474655	0.879724
2	-0.641432	0.968444
2	-0.796135	1.17283
2	0.282348	0.903134
2	0.639293	1.07402
2	1.74192	0.947771
2	-1.47098	1.08528
2	0.899178	0.933621
2	-0.594107	0.95105
2	-0.942273	1.14574
2	0.00473975	1.03656
2	1.02938	1.06551
2	-0.297471	0.891859
2	-0.746554	0.955226
2	1.04721	1.01801
2	-0.813541	1.02757
2	0.588605	1.07987
2	0.0964003	0.934021
2	0.0818951	0.964307

Cluster #	x-coordinates	y-coordinates
2	-0.660181	1.17373
2	1.62261	1.04569
2	-1.17175	1.00384
2	0.401122	1.19025
2	0.36875	0.955579
2	-0.0800132	1.15182
2	0.525828	0.924383
2	0.610587	0.91387
2	-0.249707	1.08071
2	-0.438972	0.953989
2	1.13425	1.08815
2	-0.0295738	0.964179
2	0.154947	1.05611
2	1.02719	1.04398
2	-0.847016	0.926344
2	0.0603502	0.977303
2	-0.325171	0.860964
2	0.581122	1.04687
2	-1.05464	1.22907
2	0.595779	0.971762
2	-1.54189	0.969891
2	0.269456	0.989061
2	-1.61436	1.06551
2	0.583418	0.80545
2	-0.955755	1.1068
2	0.706887	0.963498
2	0.143828	1.08714
2	-1.09074	1.074
2	1.14036	1.01413
2	2.00817	0.890302
2	-0.842738	0.888962
2	0.635763	0.95178
2	-0.922439	0.947899
2	2.13068	1.03734
2	-0.660021	1.13964
2	-0.994381	1.11126
2	0.662001	0.965423
2	-1.08794	1.01273
2	0.127849	0.908673
2	0.331306	1.01121
2	-0.740964	0.892639
2	-0.448739	1.04351
2	0.522822	0.884111
2	0.0387483	1.02578
2	0.0378355	0.822281

Cluster #	x-coordinates	y-coordinates
2	-1.74852	1.15752
2	0.026432	1.04763
2	0.0118357	0.892745
2	1.06674	1.10235
2	-0.415042	0.931725
2	-0.025456	1.00812
2	-0.599195	1.21484
2	-0.864038	1.08239
2	-0.295525	1.07476
2	-1.64925	0.944245
2	0.212015	0.961698
2	2.19212	1.07291
2	-0.756138	1.11615
2	-0.346507	1.05727
2	-0.411164	0.969974
2	0.807283	1.07984
2	-0.162405	1.18019
2	-0.320732	1.07257
2	-0.104639	0.894693
2	-0.481649	0.949199
2	0.884706	0.909968
2	0.962554	1.01271
2	-0.327928	1.06251
2	-0.868475	1.03324
2	-1.30938	1.04743
2	-0.430104	1.00119
2	-0.470567	1.07228
2	1.44223	1.26386
2	-1.46199	1.06384
2	0.128853	0.933671
2	0.097941	1.05293
2	0.163321	0.867575
2	-1.69258	1.04083
2	-0.155458	1.19292
2	-0.10142	1.04053
2	1.14637	0.930936
2	-0.847625	1.01272
2	1.20242	1.05403
2	-0.58379	1.04356
2	0.0695596	1.006
2	0.788608	0.922877
2	-0.493526	1.14754
2	0.370722	1.14764
2	1.17147	1.04819
2	-0.546828	1.024

Cluster #	x-coordinates	y-coordinates
2	0.29012	0.890958
2	-0.0136732	1.06185
2	-0.628859	1.03492
2	-1.22907	0.980334
2	0.47893	0.913365
2	-0.664373	0.938229
2	-0.995932	0.896801
2	0.659887	1.04717
2	1.25296	0.912369
2	0.817209	1.06965
2	-0.248002	1.00422
2	-0.752433	1.20477
2	-1.4079	1.04009
2	0.57472	0.900501
2	-0.602213	1.04986
2	-1.068	1.05825
2	-0.580554	0.944341
2	-0.0121175	1.13525
2	-0.201624	1.04805
2	0.829311	1.10857
2	-0.18549	1.0057
2	0.104816	1.08352
2	1.42531	1.05762
2	-0.811483	0.804322
2	-0.404832	1.09702
2	-0.144699	1.08705
2	0.668914	1.1127
2	1.51454	0.925557
2	-0.342683	1.02389
2	-0.372533	0.946919
2	-1.51585	1.03854
2	0.979759	0.763815
2	-1.24907	1.01015
2	0.661249	0.950899
2	-0.260214	1.06708
2	0.0643493	0.977164
2	0.0885806	1.13516
2	4.11187	1.01804
2	-0.745451	0.890229
2	0.396765	0.915922
2	0.0811907	0.909115
2	0.872057	1.02895
2	1.52586	1.02334
2	-0.0270912	1.05466
2	0.436307	0.922862

Cluster #	x-coordinates	y-coordinates
2	0.0455139	0.888064
2	0.726057	1.00885
2	1.38665	1.01854
2	-0.86399	1.08028
2	0.219234	1.03363
2	1.39332	1.02581
2	-0.481671	0.753056
2	-0.246028	0.952999
2	1.07463	0.938035
2	0.0824648	1.05713
2	-1.85445	1.03703
2	0.582313	0.909837
2	-1.0246	1.03643
2	0.491487	0.980475
2	-1.67327	0.968878
2	1.01174	1.06912
2	1.1363	1.04385
2	1.19324	1.00072
2	0.48978	1.07051
2	-0.388014	1.00923
2	1.36391	1.00373
2	0.475369	1.0419
2	-0.729257	1.0155
2	-0.38098	0.971334
2	-0.145488	0.849341
2	-0.421914	0.889686
2	-0.450638	0.992018
2	-0.710486	0.986675
2	0.780835	0.972715
2	0.241853	1.03867
2	-1.33978	0.963312
2	1.54798	0.935159
2	0.456219	0.897134
2	0.796422	1.16052
2	-1.21631	1.01596
2	-0.414238	0.999853
2	1.32583	0.883017
2	-0.141331	0.978879
2	0.48962	1.07361
2	-1.27876	0.701623
2	-0.232624	0.934124
2	0.498465	0.96941
2	-0.0801813	1.08802
2	0.893336	1.17852
2	-0.911264	1.0523

Cluster #	x-coordinates	y-coordinates
2	-0.502718	0.825122
2	0.19044	0.874579
2	0.705179	1.13396
2	-0.730076	0.926078
2	-0.660691	1.06783
2	-0.870188	0.998367
2	0.00569778	0.879347
2	0.801124	0.909207
2	-1.30837	0.95193
2	-1.09476	1.01524
2	-0.504489	0.957962
2	0.157636	0.727758
2	0.418801	1.07859
2	-1.13583	0.979322
2	0.539733	0.894501
2	0.0794719	0.888648
2	0.73615	1.04685
2	1.06396	1.03989
2	0.635945	0.959941
2	0.242583	0.880743
2	-1.77716	1.0672
2	-0.301302	0.87917
2	0.134548	0.835839
2	-0.0523225	1.02427
2	0.0342966	0.779578
2	0.580396	1.12087
2	1.36011	1.10632
2	-0.223135	0.959355
2	-0.210795	1.08053
2	0.346453	1.02521
2	0.697386	1.07078
2	0.292267	0.86639
2	1.53767	0.933195
2	-1.23541	1.00092
2	-1.29207	0.970125
2	0.110572	0.85296
2	-0.506952	1.04924
2	1.11085	1.16563
2	-0.980684	1.01326
2	-2.38914	1.05244
2	-0.159829	1.02212
2	0.256945	0.939834
2	1.05556	1.0859
2	0.925462	1.13952
2	-0.207237	1.0528

Cluster #	x-coordinates	y-coordinates
2	0.107325	1.00507
2	1.49155	0.987733
2	-0.719025	0.923848
2	0.579421	1.03337
2	1.5123	0.958644
2	-0.365782	0.914832
2	0.860474	0.977807
2	0.145721	0.962036
2	-1.74662	0.940761
2	0.965422	1.12073
2	-0.359882	1.05117
2	-1.19374	0.945698
2	-2.06286	1.02228
2	1.54997	1.19172
2	-0.0859812	0.958217
2	-0.123228	1.09792

Appendix I

Sizes5
Number of dimensions: 2
Number of clusters: 4
Number of points: 1000

Cluster #	x-coordinates	y-coordinates
1	10.8869	8.90031
1	10.2351	14.3846
1	8.42297	10.9991
1	7.62384	10.6236
1	8.15414	12.8138
1	11.8427	8.35072
1	9.88845	10.2225
1	7.44341	10.7994
1	6.93062	10.4146
1	8.37611	7.76093
1	8.77073	9.42551
1	10.4262	9.86048
1	12.4098	9.17625
1	11.7372	8.3753
1	12.4	10.969
1	14.2438	8.77079
1	8.66348	10.79
1	12.7459	10.3988
1	8.75253	12.331
1	13.8072	7.53461
1	10.6088	9.63339
1	10.2128	8.40602
1	11.9443	10.1502
1	7.1348	8.78567
1	11.5463	9.02141
1	7.39641	8.81803
1	12.1066	9.80128
1	8.43751	10.0806
1	8.90289	12.152
1	6.98745	13.2447
1	8.30263	10.3166
1	9.85813	8.12388
1	8.33359	7.83237
1	6.72891	9.521
1	8.22207	8.13031
1	12.7156	8.69083
1	9.76829	6.29709
1	4.96144	10.6544
1	11.0993	8.04041

Cluster #	x-coordinates	y-coordinates
1	8.65045	8.87529
1	9.88847	14.8919
1	10.4655	9.52826
1	8.68752	5.40063
1	9.98631	7.94411
1	11.5411	7.36012
1	10.2612	7.73086
1	9.58338	5.20515
1	10.1701	11.1436
1	10.0452	9.21341
1	12.874	9.06637
1	14.0355	9.9843
1	10.962	10.7913
1	12.4406	15.3743
1	11.1329	12.1674
1	12.7785	9.63481
1	5.1106	6.96972
1	9.5689	11.7748
1	4.62803	5.59998
1	7.14721	9.80502
1	7.87537	15.6053
1	7.94672	9.24352
1	10.3796	10.4133
1	9.73973	10.97
1	10.6888	7.77007
1	8.75719	9.27635
1	12.0341	11.368
1	8.68806	8.79218
1	9.15804	6.73175
1	8.96502	10.8168
1	10.584	9.61832
1	8.45052	8.92374
1	10.3678	10.0026
1	11.3355	8.23074
1	7.923	13.8124
1	10.5226	5.13192
1	11.5021	7.17919
1	10.9466	13.075
1	8.61106	11.8888
1	10.8997	10.8095
1	9.2002	9.1496
1	9.08939	8.36524
1	8.23789	12.2109
1	13.7936	10.0255
1	12.3615	11.3517

Cluster #	x-coordinates	y-coordinates
1	8.19995	10.5163
1	7.69117	11.2006
1	9.75371	11.0339
1	10.5206	7.58053
1	13.6141	10.8036
1	10.88	7.46389
1	8.6799	10.5088
1	7.14465	8.20637
1	13.9754	9.22325
1	13.0996	12.4677
1	13.2212	13.404
1	11.0622	8.78948
1	10.5646	10.3501
1	12.3924	8.55951
1	10.1297	10.2904
1	6.60784	7.08943
1	14.9841	7.66974
1	11.9849	11.3735
1	9.39196	9.12451
1	8.96013	11.8974
1	7.58327	9.72671
1	13.1884	10.3447
1	8.12456	10.4952
1	7.81294	11.3589
1	8.50072	10.7605
1	9.83197	10.9652
1	9.18977	9.49708
1	7.28909	11.5578
1	11.4352	9.27058
1	10.2713	12.6155
1	8.27077	9.12773
1	9.80628	10.9402
1	13.074	9.75425
1	11.6244	10.2268
1	10.8233	11.1769
1	9.53954	8.28286
1	8.89304	9.05684
1	8.21062	9.71568
1	7.75693	9.77286
1	10.9447	11.3285
1	10.3366	9.37285
1	7.38557	10.2635
1	5.99344	11.6282
1	10.3016	10.798
1	10.9883	8.91777

Cluster #	x-coordinates	y-coordinates
1	8.26144	9.37154
1	8.22509	13.6647
1	10.8377	11.6359
1	12.5449	8.66001
1	11.1864	10.5664
1	10.4516	10.3456
1	9.24176	9.97952
1	14.6176	8.82867
1	14.3034	13.3094
1	11.5333	10.1894
1	8.99051	10.8047
1	8.30565	10.0129
1	9.65672	4.48101
1	11.419	10.5332
1	8.89982	6.93163
1	8.05603	11.582
1	9.27771	10.8513
1	13.0889	13.5084
1	10.1137	9.33204
1	8.9874	10.3377
1	5.86696	3.72908
1	11.8231	9.96606
1	10.2509	11.9315
1	6.61921	7.22275
1	9.00662	9.37667
1	11.2048	9.34803
1	10.4439	11.2053
1	6.49574	10.8589
1	11.5033	7.7722
1	11.4136	11.2649
1	11.0854	7.51229
1	12.3264	10.7177
1	10.2974	11.6089
1	6.09451	7.22484
1	7.07543	11.0504
1	8.57765	11.4106
1	10.113	9.01184
1	8.44645	8.80073
1	7.29708	9.42485
1	13.0852	10.3451
1	10.1784	9.76368
1	8.98864	11.5016
1	9.98314	9.26775
1	12.1025	10.0122
1	10.8941	7.72206

Cluster #	x-coordinates	y-coordinates
1	8.09425	9.32517
1	12.5617	7.55383
1	11.8284	12.6769
1	11.9246	11.1072
1	11.4699	13.522
1	8.55783	12.9013
1	12.8298	7.9465
1	9.2984	8.10084
1	11.5606	12.0201
1	11.2796	11.2787
1	9.26861	9.94405
1	10.9214	12.1895
1	12.2121	13.7067
1	11.7335	11.7817
1	10.7228	10.1396
1	11.1728	9.21252
1	10.0655	10.3168
1	12.2733	13.4333
1	11.7034	9.7175
1	12.4301	9.29364
1	12.5375	9.62404
1	10.5535	10.8001
1	9.15439	13.2817
1	7.87427	12.2706
1	10.2936	15.7329
1	14.6806	6.23815
1	8.95995	10.3893
1	12.1811	8.92067
1	10.9608	6.29033
1	4.85143	12.3817
1	12.1244	6.02473
1	8.96757	6.91482
1	11.7386	8.45394
1	10.0257	9.23013
1	8.59417	10.4402
1	5.83841	8.04765
1	9.70338	9.5049
1	6.69742	8.05213
1	11.484	7.7704
1	9.72184	10.7282
1	7.99975	9.84839
1	9.05103	12.2949
1	12.1552	11.3228
1	10.6778	12.2226
1	11.5537	11.6105

Cluster #	x-coordinates	y-coordinates
1	9.72175	13.9799
1	9.06488	10.2915
1	10.1271	10.5359
1	10.0916	10.6914
1	4.9469	10.2548
1	12.485	12.5136
1	8.78335	5.92341
1	11.5852	10.3059
1	11.3542	9.79086
1	9.62026	10.2457
1	8.90339	10.9767
1	5.82896	10.4505
1	11.4205	12.7653
1	7.33706	10.291
1	10.8081	10.3648
1	10.9049	11.5844
1	11.1781	7.60735
1	8.22144	10.5966
1	10.4992	8.21706
1	9.40329	4.6562
1	9.04497	8.49666
1	8.76057	11.2287
1	10.3005	8.83803
1	10.4239	11.6873
1	9.7779	11.4288
1	11.4119	13.3982
1	10.1564	11.5774
1	8.02947	9.46227
1	11.8939	10.7132
1	7.92772	9.10664
1	15.0658	9.48576
1	12.2098	10.5809
1	6.43288	8.18468
1	12.2904	11.4869
1	9.51209	10.4246
1	11.0667	13.7852
1	10.0659	3.6566
1	7.55539	9.34089
1	8.02627	10.3199
1	11.2312	10.5011
1	8.86529	6.25793
1	10.7617	8.81528
1	8.92686	10.2521
1	9.4241	9.82858
1	11.1382	10.5309

Cluster #	x-coordinates	y-coordinates
1	9.30184	9.58822
1	11.6862	7.09235
1	10.5885	12.5343
1	11.1284	13.8004
1	8.91829	10.9595
1	7.20989	6.82066
1	10.4652	7.65442
1	11.4801	12.2281
1	8.97343	9.94565
1	10.5845	9.4713
1	11.3118	13.126
1	13.5629	11.1205
1	12.4572	9.36804
1	10.0915	8.84189
1	10.4289	10.3071
1	10.8063	9.26778
1	6.53268	9.98746
1	9.5066	9.01718
1	8.58066	11.1847
1	9.60144	10.1158
1	12.6916	13.2338
1	10.0809	13.2749
1	5.65623	9.81618
1	12.2767	10.0468
1	10.2858	10.1394
1	9.83867	6.55852
1	9.03894	11.6848
1	8.53992	8.02881
1	9.50943	12.8488
1	12.7279	7.85816
1	10.38	10.482
1	11.7461	9.76796
1	10.7009	9.59991
1	8.83462	9.87968
1	10.6559	8.43993
1	9.43013	10.7689
1	7.96656	6.48615
1	7.15654	10.3203
1	10.8821	10.2653
1	10.2837	13.1129
1	11.9498	7.98478
1	9.21953	10.9258
1	10.832	9.74374
1	13.6719	10.121
1	10.2191	9.8628

Cluster #	x-coordinates	y-coordinates
1	10.3521	9.62285
1	11.8547	9.79455
1	11.2065	9.55941
1	7.04108	10.9299
1	8.3232	7.47834
1	8.58332	10.5916
1	12.3597	9.332
1	11.4088	10.9362
1	9.98518	7.88393
1	7.78461	9.68036
1	10.5488	11.0076
1	10.0503	7.82913
1	12.0031	10.3038
1	12.1214	8.79344
1	9.38037	10.2616
1	8.33598	9.15018
1	8.38598	10.6432
1	9.85346	7.39228
1	11.7006	12.1184
1	8.42385	11.5394
1	8.26369	6.16326
1	9.45567	8.40583
1	8.68462	10.6628
1	12.8761	10.0897
1	9.94184	10.2855
1	13.0969	10.3149
1	8.67146	10.117
1	7.88473	13.8841
1	8.52663	10.1995
1	11.3268	10.8231
1	6.9829	11.3902
1	10.2351	13.5102
1	9.07929	12.3265
1	10.1965	12.1256
1	8.36285	10.131
1	10.6471	9.16923
1	9.22564	8.75443
1	9.41353	11.0297
1	8.45745	8.80728
1	14.6545	10.5321
1	10.4237	9.28147
1	12.1179	7.56837
1	10.0092	7.45614
1	11.1888	11.2968
1	11.5369	9.01416

Cluster #	x-coordinates	y-coordinates
1	12.0782	8.25199
1	11.0409	7.45656
1	8.77283	8.92015
1	9.96012	8.80275
1	12.435	13.014
1	10.8183	6.67993
1	10.6925	7.85647
1	10.8108	10.3533
1	12.987	13.2321
1	9.69215	10.1052
1	9.07185	9.89733
1	5.21251	9.75238
1	11.1382	12.3187
1	11.5011	8.90703
1	13.3721	8.9819
1	10.87	6.59046
1	12.111	10.6642
1	10.29	10.5419
1	7.74604	9.24998
1	10.2652	8.02362
1	11.3945	10.1244
1	11.0715	9.53501
1	9.71278	8.88074
1	5.67517	13.1317
1	10.1521	14.0943
1	8.09135	9.97794
1	8.63612	11.3978
1	8.94673	8.27578
1	11.611	11.2006
1	9.79342	7.3699
1	13.1324	13.0537
1	8.34878	9.37051
1	6.70832	10.5941
1	10.372	11.0969
1	10.0823	8.65497
1	7.84324	11.9941
1	9.80992	8.44993
1	11.4325	7.80047
1	14.0067	9.88184
1	7.42375	11.8388
1	8.29323	6.8388
1	7.10049	9.64062
1	12.4038	12.7862
1	10.6644	8.78223
1	15.4086	7.91216

Cluster #	x-coordinates	y-coordinates
1	5.79341	10.1976
1	7.94788	9.48537
1	8.41636	8.09692
1	10.5092	11.2089
1	10.8303	10.7209
1	13.7557	11.7952
1	12.2342	6.97067
1	5.16328	10.1571
1	10.6665	12.4289
1	11.899	11.327
1	13.8477	6.00502
1	10.0501	9.52532
1	9.46262	9.71814
1	7.48793	11.9092
1	11.0889	10.6071
1	8.61304	10.0545
1	11.6927	11.1904
1	8.24176	8.30187
1	9.63006	8.30831
1	9.39677	9.64433
1	8.05077	9.91366
1	8.20956	14.2716
1	8.66306	10.1032
1	11.1352	12.924
1	8.65615	6.82804
1	13.0822	9.68518
1	11.1388	9.06657
1	12.9709	3.84507
1	11.3677	7.82899
1	7.09297	8.73742
1	8.38542	10.4146
1	8.0612	10.2499
1	7.69899	11.9964
1	12	7.15398
1	14.0366	11.6712
1	10.6944	9.6483
1	10.5207	9.50521
1	8.29434	9.87807
1	11.4631	9.14777
1	10.4924	12.7432
1	10.2469	9.61103
1	10.678	8.33112
1	8.97563	8.20904
1	8.54909	10.6722
1	10.4253	9.64531

Cluster #	x-coordinates	y-coordinates
1	11.1416	9.21496
1	11.6957	13.0638
1	10.5206	10.2748
1	8.57042	8.92525
1	9.89724	11.7303
1	10.7374	7.88287
1	14.5009	7.56714
1	14.1285	11.6302
1	7.40752	9.43451
1	14.0919	11.9924
1	7.32999	8.80763
1	7.92855	11.4773
1	9.73682	10.9469
1	11.2653	10.1761
1	9.40501	10.6738
1	9.44774	13.233
1	10.1072	8.62375
1	8.1291	13.7398
1	12.4774	11.507
1	12.9423	10.2582
1	8.58606	9.81835
1	4.99953	10.5729
1	10.3235	11.4762
1	9.05633	9.10885
1	10.2094	10.9522
1	10.1164	11.8667
1	11.6025	6.55482
1	11.3555	9.19486
1	11.7569	6.61341
1	10.7672	12.5808
1	14.4244	8.79388
1	8.60233	11.4726
1	8.38937	12.5967
1	10.3913	13.1963
1	11.2612	10.133
1	7.70357	9.40515
1	10.8941	8.31102
1	11.5573	9.06004
1	10.8918	14.0648
1	10.9971	7.20352
1	11.2632	9.10212
1	9.37601	3.3263
1	5.87086	11.306
1	10.0199	10.6677
1	10.7069	9.35036

Cluster #	x-coordinates	y-coordinates
1	8.42352	5.92902
1	7.7596	12.3898
1	10.4808	11.1436
1	12.5363	9.70335
1	9.89809	9.60453
1	9.16612	8.0322
1	7.28647	12.0466
1	9.74954	10.5595
1	9.52525	8.9526
1	7.95169	9.46083
1	8.54937	10.2467
1	9.07892	8.82387
1	10.324	12.2041
1	9.92273	10.8209
1	11.4912	10.1373
1	9.94708	12.619
1	8.57657	7.85303
1	6.81715	6.5024
1	11.6489	9.99123
1	10.4597	10.8116
1	10.3686	9.26428
1	8.70675	10.1803
1	10.281	7.48928
1	10.0041	6.2279
1	12.9674	11.4241
1	10.8492	7.14905
1	5.10064	8.24691
1	7.66909	8.85326
1	10.7878	7.28104
1	11.9108	11.1086
1	8.7169	11.203
1	10.0273	15.5648
1	6.68034	10.1737
1	10.4772	13.5555
1	10.2173	8.36863
1	10.3539	8.51841
1	11.234	11.0538
1	10.4996	9.7357
1	7.49137	8.39037
1	7.06861	9.76414
1	11.4811	11.4836
1	10.0891	12.4186
1	6.13673	9.02496
1	8.57357	9.90237
1	9.52098	10.7896

Cluster #	x-coordinates	y-coordinates
1	10.961	6.42147
1	8.24382	9.52104
1	9.12493	10.3672
1	8.636	12.5042
1	9.91586	8.14531
1	10.5911	10.3184
1	11.0956	11.6695
1	11.9021	10.812
1	7.88254	7.85284
1	9.55781	11.6599
1	8.07403	10.7072
1	9.52685	13.0765
1	12.5384	11.0591
1	9.59149	9.5399
1	9.79725	7.99536
1	10.7473	13.2989
1	9.44138	7.15187
1	13.2057	7.43902
1	11.1243	12.9955
1	12.097	9.27379
1	13.9547	13.3202
1	9.32771	12.5966
1	8.47363	8.7214
1	7.17759	11.9432
1	12.4502	9.38928
1	9.63029	4.39701
1	7.69631	11.6684
1	14.5651	11.0458
1	9.25412	13.8967
1	11.1228	8.37712
1	10.2154	9.26414
1	11.4604	10.0429
1	7.48528	11.7121
1	12.4918	10.5119
1	9.29306	12.6789
1	7.3116	10.2001
1	11.942	10.1567
1	7.46086	10.398
1	11.9182	8.04355
1	10.5048	9.78804
1	7.82697	10.1391
1	8.87558	10.0827
1	9.4209	9.6969
1	11.743	10.8382
1	10.7736	9.88596

Cluster #	x-coordinates	y-coordinates
1	11.2315	13.8858
1	11.0692	9.7259
1	11.3989	8.80259
1	10.9686	10.9376
1	8.04747	10.5557
1	9.7921	13.4998
1	11.8339	11.7176
1	4.99809	8.87165
1	7.4673	8.89433
1	13.436	12.4011
1	6.82915	10.5166
1	7.02752	9.66797
1	10.2604	9.27056
1	7.29251	10.4801
1	8.45632	8.2765
1	4.62737	11.3645
1	9.07103	10.9008
1	10.6807	9.5079
1	13.0258	11.1581
1	12.7544	11.9661
1	9.63023	12.8539
1	9.74277	6.69905
1	13.2591	8.57197
1	7.96592	12.3038
1	8.78609	9.45465
1	8.86717	9.70401
1	11.6857	10.231
1	12.839	12.6775
1	9.7377	11.0885
1	11.2749	9.01048
1	8.6599	11.048
1	10.8274	10.2355
1	9.46617	12.1699
1	13.965	10.2589
1	10.2093	10.8708
1	9.90293	6.02081
1	10.3877	11.8977
1	13.1763	7.66768
1	6.28016	10.033
1	10.5281	8.15028
1	4.98785	9.01084
1	11.3962	9.17309
1	10.1688	6.78229
1	10.0702	10.8924
1	11.1779	9.99598

Cluster #	x-coordinates	y-coordinates
1	10.0263	8.29082
1	8.73473	9.49804
1	11.2697	10.7515
1	9.38193	8.20601
1	11.6214	6.35056
1	8.99363	8.86355
1	10.0944	13.1662
1	10.2226	12.0386
1	12.3471	11.6172
1	8.66293	12.5684
1	10.3161	8.15256
1	10.1846	8.40759
1	9.45139	8.93852
1	11.6456	10.3936
1	9.88694	11.6002
1	8.93552	11.4514
1	9.6951	9.91341
1	11.6685	8.57359
1	8.00649	12.7901
1	6.63594	12.1043
1	10.1016	11.3048
1	6.9855	13.4959
1	11.5113	11.2034
1	10.3882	12.4993
1	10.7298	9.61872
1	14.1075	10.6475
1	11.7618	16.2687
1	10.5327	10.1429
1	8.75512	13.4043
1	10.7883	10.8205
1	10.4235	12.1473
1	5.65279	8.53653
1	9.52867	11.1041
1	12.6944	5.33468
1	10.5189	8.66986
1	10.7148	10.5051
1	10.4133	8.71781
1	8.97895	8.37105
1	7.50431	9.52179
1	11.294	10.6397
1	12.4938	13.9646
1	6.14092	10.8915
1	8.38062	9.36562
1	11.6827	9.57211
1	8.1076	9.03256

Cluster #	x-coordinates	y-coordinates
1	7.43198	9.69143
1	8.67604	7.12591
1	11.0545	9.42357
1	6.30996	10.9994
1	5.87171	5.23757
1	9.99545	8.39789
1	11.1416	10.3637
1	7.35206	9.87194
1	8.32265	5.94821
1	9.11632	5.70995
1	11.2794	7.06624
1	6.70881	9.87014
1	7.41243	10.1185
1	9.47617	10.8507
1	12.3635	11.3513
1	7.8153	10.1307
1	14.2507	9.09068
1	8.43891	9.90734
1	8.22326	12.0147
1	7.61477	12.7117
1	13.1786	10.6146
1	9.72357	4.15775
1	8.87579	11.7517
1	8.56133	10.8517
1	7.35155	10.1718
1	8.41769	11.5432
1	9.75027	7.91884
1	7.97935	10.7562
1	8.78172	8.9383
1	8.89966	12.3731
1	4.79748	8.80076
1	14.7217	6.84553
1	9.37654	8.96348
1	11.0523	7.5905
1	9.15847	9.9523
1	9.71902	7.15055
1	11.9937	9.36838
1	8.30161	5.36268
1	8.97805	14.8972
1	10.1892	12.7405
1	9.25317	10.6967
1	9.6697	6.76404
1	10.0282	13.1656
1	9.614	11.701
1	12.6923	11.3228

Cluster #	x-coordinates	y-coordinates
1	12.6939	9.80862
1	10.3861	9.93775
1	12.2359	19.101
1	10.988	10.087
1	5.75422	11.2509
1	12.2493	13.7566
1	10.847	9.02605
1	10.6819	13.2635
1	8.73498	10.1842
1	8.44586	9.81048
1	6.81464	10.7879
1	10.708	15.4629
1	7.31169	16.0543
1	12.3828	11.227
1	9.14216	7.76569
1	10.8018	8.96137
1	12.7452	7.72199
1	11.4552	10.2868
1	10.6025	10.0635
1	11.9244	8.93128
1	8.61645	9.1797
1	6.71431	13.9056
1	8.83993	9.60691
1	7.17559	9.70206
1	11.3469	8.59737
1	10.1402	11.4194
1	11.7447	17.0384
1	5.63899	11.3076
1	10.4955	8.7421
1	10.7231	10.0424
1	8.3234	9.72751
1	8.78314	7.83971
1	5.97789	7.76312
1	4.69003	8.67515
1	9.56033	7.7664
1	10.1418	13.5157
1	12.602	9.76487
1	10.5091	8.33631
1	7.01947	11.2306
1	12.1517	10.8001
1	8.78126	11.9908
1	10.9131	8.83279
1	8.19489	10.6539
1	9.35846	11.123
1	13.1782	9.91973

Cluster #	x-coordinates	y-coordinates
1	10.0844	9.0755
1	11.7775	11.0523
1	12.1478	9.44351
1	8.86596	10.2127
1	5.76536	8.1999
1	11.1851	9.86406
1	7.82146	7.84346
1	6.67554	8.45827
1	7.12663	10.1942
1	8.18845	8.58133
2	8.41998	-1.82541
2	13.1384	-4.86785
2	11.0831	0.0151826
2	13.2276	4.55368
2	9.87016	0.669372
2	9.81586	1.92494
2	7.91675	-0.893096
2	6.29752	-1.88485
2	10.8293	-0.279995
2	10.4901	-0.0496448
2	6.44276	-4.4685
2	8.26741	-3.91719
2	13.7036	1.72321
2	11.5403	-1.51702
2	8.93345	-0.419497
2	6.72924	0.846747
2	10.47	-0.103674
2	9.38407	2.54254
2	9.77357	2.46956
2	12.0241	-1.57486
2	8.61522	2.16746
2	11.0617	-1.89033
2	8.87016	-2.52064
2	7.73234	0.0201097
2	15.4899	2.06303
2	14.0673	-2.83901
2	6.71599	1.7574
2	6.48909	0.230445
2	6.97797	-4.00173
2	8.82668	-2.13668
2	11.9628	0.464803
2	11.0599	0.257883
2	8.14536	-0.414412
2	9.56754	-0.615621
2	8.14205	-1.79358

Cluster #	x-coordinates	y-coordinates
2	13.279	-2.07361
2	10.494	1.84671
2	12.0687	-0.860238
2	7.66924	0.724457
2	7.90006	-1.83699
2	8.88374	0.804698
2	12.6357	-3.61391
2	9.43489	0.199266
2	11.9982	3.05217
2	12.8465	-1.16848
2	8.58099	0.762063
2	11.4408	-1.53226
2	11.66	1.58875
2	11.3547	0.191069
2	10.3793	-1.76293
2	13.217	0.759293
2	11.1378	-4.13905
2	6.09252	-4.00188
2	7.47246	1.10789
2	12.2729	0.61778
2	10.7726	3.42341
2	9.94763	-2.52238
2	14.6143	-0.645713
2	8.64147	1.88124
2	12.4286	-2.55316
2	12.9702	-0.29069
2	9.93146	1.33234
2	11.7264	-2.32503
2	12.6945	2.66497
2	10.9086	1.64279
2	11.0265	1.69435
2	9.58326	-0.516775
2	8.93725	0.544152
2	11.0697	-3.4646
2	10.0002	-1.27936
2	12.0853	-0.986415
2	7.74141	0.486176
2	8.48404	-0.470388
2	8.61203	1.89278
2	12.3186	3.5256
2	11.9408	2.67473
2	8.63223	-0.262502
3	-0.823207	6.21884
3	0.770079	10.7011
3	-0.366687	8.30968

Cluster #	x-coordinates	y-coordinates
3	-1.6768	9.87872
3	-1.5988	9.59061
3	2.87947	12.2587
3	-1.00054	11.037
3	0.452546	8.79797
3	1.75848	8.44889
3	3.97723	12.8546
3	-1.11711	8.54479
3	0.458293	9.43719
3	-1.781	8.82055
3	3.64348	9.42872
3	-0.577629	7.73154
3	3.62559	10.2998
3	2.45118	11.0656
3	1.23068	11.5273
3	2.92727	7.57949
3	-0.154011	10.8565
3	-2.15371	7.72086
3	1.68162	12.8621
3	-4.73877	11.2232
3	4.3517	10.9182
3	-4.4254	8.92338
3	-1.81507	10.2944
3	1.07894	9.15565
3	0.112692	9.11664
3	0.256398	10.5572
3	-1.52009	10.0325
3	-1.96108	9.0107
3	4.62896	7.49023
3	2.58344	12.1998
3	0.120588	9.17856
3	-0.73567	11.1666
3	-0.667698	9.48769
3	-0.139785	8.70573
3	2.04754	9.17896
3	-3.21331	10.0096
3	-3.87779	11.5858
3	2.64911	10.9942
3	1.53348	8.88979
3	3.36791	11.7213
3	-0.560543	9.74794
3	3.7601	9.41015
3	-0.666302	13.1042
3	1.50519	13.5286
3	1.98847	11.4342

Cluster #	x-coordinates	y-coordinates
3	-2.43746	13.6042
3	2.85614	7.45292
3	2.89704	12.055
3	2.75298	12.5308
3	-0.831475	10.1185
3	0.233346	10.9697
3	-0.207825	9.95284
3	0.589381	12.4699
3	-0.194747	9.77858
3	1.09749	10.5995
3	-1.10404	10.526
3	-0.00409853	10.2686
3	-1.57901	11.5201
3	-2.14809	7.26784
3	-0.349655	10.3457
3	-0.178729	9.8941
3	-2.97557	10.3165
3	-2.39714	7.66772
3	1.09521	11.377
3	-2.26924	10.4074
3	-1.22008	7.05122
3	2.0379	8.26964
3	-3.20238	9.43048
3	-0.00710754	12.3837
3	-3.9954	12.2144
3	1.57562	12.9156
3	-1.62371	7.82655
3	-0.0719283	11.6163
3	-2.47918	10.8772
4	-3.62923	-3.09808
4	2.009	-1.87308
4	-0.925613	1.97743
4	0.885448	1.54807
4	-1.48709	1.0756
4	-1.60664	-4.26231
4	-1.06959	1.47002
4	5.02174	-0.295102
4	-5.32088	-0.186381
4	4.99777	-1.35283
4	-0.342985	0.255095
4	1.2257	3.2006
4	-1.73855	-2.08237
4	-0.654133	4.6342
4	-1.63698	0.0343314
4	3.67187	0.419183

Cluster #	x-coordinates	y-coordinates
4	0.300867	2.24501
4	-2.04103	3.14598
4	-2.60218	-0.842164
4	-2.79024	1.07798
4	-0.436555	-0.0776824
4	0.124781	-0.286745
4	2.16834	1.56495
4	-2.66181	1.28653
4	-0.893574	1.91948
4	-0.524675	-2.38481
4	-1.37721	-0.444991
4	-2.43264	-1.1677
4	1.01737	-0.624078
4	0.484473	0.491103
4	-0.61291	1.46619
4	-2.03765	0.651713
4	4.1855	-1.73116
4	2.56479	1.19192
4	-3.05858	-0.555413
4	0.0448892	-2.19431
4	-4.5079	-0.535321
4	-2.96154	0.0059469
4	0.452152	-0.766279
4	-2.06501	1.11046
4	2.87682	-2.05469
4	0.583081	2.77243
4	-0.222002	-3.35365
4	-0.0956423	0.796767
4	1.00487	-0.363257
4	-0.0921501	-1.78807
4	-3.06096	-0.537697
4	-1.73969	-1.92975
4	1.21781	2.1954
4	-0.379072	0.976532
4	0.100096	-4.29977
4	-1.33817	-6.71431
4	-0.6899	-2.1218
4	-0.186766	0.120095
4	-0.839851	-2.0968
4	-4.04476	-2.28471
4	-1.75774	1.39218
4	2.82066	0.946066
4	-1.41052	0.372142
4	2.07725	0.116542
4	2.64708	1.43672

Cluster #	x-coordinates	y-coordinates
4	-3.11942	-4.66994
4	-3.40476	-3.47829
4	0.41902	0.680316
4	2.31121	-1.20395
4	-1.17464	1.39487
4	1.16186	-1.914
4	1.57283	-1.4431
4	3.86712	-2.27142
4	0.443945	-1.16414
4	2.11239	-1.44947
4	0.114929	-5.03818
4	1.79477	-0.0947745
4	0.00876879	-0.305248
4	1.97011	0.119301
4	-1.13789	-3.62316
4	1.59897	-0.911978

Appendix J

Spiral
Number of dimensions: 2
Number of clusters: 2
Number of points: 1000

Cluster #	x-coordinates	y-coordinates
1	1.5	0
1	1.50973	0.0284611
1	1.51892	0.0572891
1	1.52755	0.0864734
1	1.53562	0.116003
1	1.54312	0.145868
1	1.55003	0.176056
1	1.55635	0.206556
1	1.56207	0.237356
1	1.56717	0.268445
1	1.57166	0.29981
1	1.57552	0.331439
1	1.57873	0.363319
1	1.58131	0.395437
1	1.58323	0.42778
1	1.58448	0.460335
1	1.58508	0.493089
1	1.58499	0.526028
1	1.58422	0.559137
1	1.58277	0.592403
1	1.58062	0.625812
1	1.57777	0.659349
1	1.57421	0.692999
1	1.56995	0.726748
1	1.56497	0.760581
1	1.55926	0.794483
1	1.55283	0.828439
1	1.54567	0.862433
1	1.53778	0.896449
1	1.52916	0.930473
1	1.51979	0.964488
1	1.50968	0.998479
1	1.49883	1.03243
1	1.48723	1.06632
1	1.47488	1.10015
1	1.46179	1.13388
1	1.44794	1.16751
1	1.43334	1.20101
1	1.41799	1.23438

Cluster #	x-coordinates	y-coordinates
1	1.40189	1.2676
1	1.38504	1.30064
1	1.36744	1.3335
1	1.34909	1.36615
1	1.32999	1.39858
1	1.31015	1.43078
1	1.28956	1.46272
1	1.26823	1.49439
1	1.24616	1.52577
1	1.22336	1.55685
1	1.19983	1.58761
1	1.17557	1.61803
1	1.15059	1.6481
1	1.12489	1.6778
1	1.09848	1.70712
1	1.07136	1.73603
1	1.04353	1.76452
1	1.01502	1.79258
1	0.985813	1.82018
1	0.955926	1.84732
1	0.925365	1.87398
1	0.894136	1.90014
1	0.86225	1.92578
1	0.829712	1.95089
1	0.796533	1.97546
1	0.762721	1.99946
1	0.728287	2.02289
1	0.693238	2.04573
1	0.657587	2.06797
1	0.621342	2.08958
1	0.584515	2.11055
1	0.547118	2.13088
1	0.509161	2.15055
1	0.470656	2.16954
1	0.431615	2.18783
1	0.392052	2.20542
1	0.351978	2.2223
1	0.311406	2.23844
1	0.270351	2.25384
1	0.228825	2.26849
1	0.186843	2.28237
1	0.144418	2.29546
1	0.101566	2.30777
1	0.0583018	2.31927
1	0.0146397	2.32995

Cluster #	x-coordinates	y-coordinates
1	-0.0294045	2.33982
1	-0.0738153	2.34884
1	-0.118577	2.35702
1	-0.163672	2.36434
1	-0.209086	2.3708
1	-0.254801	2.37638
1	-0.3008	2.38108
1	-0.347066	2.38488
1	-0.393582	2.38778
1	-0.44033	2.38977
1	-0.487292	2.39085
1	-0.534451	2.391
1	-0.581788	2.39021
1	-0.629284	2.38849
1	-0.676921	2.38583
1	-0.72468	2.38221
1	-0.772542	2.37764
1	-0.820489	2.37211
1	-0.8685	2.36561
1	-0.916557	2.35814
1	-0.964639	2.3497
1	-1.01273	2.34027
1	-1.0608	2.32987
1	-1.10884	2.31848
1	-1.15683	2.30611
1	-1.20474	2.29275
1	-1.25256	2.2784
1	-1.30026	2.26305
1	-1.34783	2.24672
1	-1.39524	2.22939
1	-1.44248	2.21107
1	-1.48952	2.19176
1	-1.53634	2.17146
1	-1.58293	2.15017
1	-1.62925	2.12789
1	-1.6753	2.10463
1	-1.72104	2.08039
1	-1.76647	2.05516
1	-1.81155	2.02896
1	-1.85627	2.00179
1	-1.90061	1.97365
1	-1.94454	1.94454
1	-1.98805	1.91448
1	-2.03112	1.88347
1	-2.07372	1.85151

Cluster #	x-coordinates	y-coordinates
1	-2.11583	1.81862
1	-2.15744	1.78479
1	-2.19852	1.75003
1	-2.23905	1.71436
1	-2.27902	1.67778
1	-2.3184	1.64031
1	-2.35718	1.60194
1	-2.39533	1.56269
1	-2.43284	1.52257
1	-2.46968	1.48159
1	-2.50583	1.43975
1	-2.54129	1.39709
1	-2.57602	1.35359
1	-2.61002	1.30928
1	-2.64325	1.26417
1	-2.67571	1.21826
1	-2.70738	1.17159
1	-2.73823	1.12415
1	-2.76825	1.07596
1	-2.79743	1.02704
1	-2.82574	0.977395
1	-2.85317	0.927051
1	-2.8797	0.876019
1	-2.90532	0.824315
1	-2.93001	0.771955
1	-2.95376	0.718957
1	-2.97655	0.665337
1	-2.99836	0.611113
1	-3.01918	0.556302
1	-3.03899	0.500922
1	-3.05779	0.444993
1	-3.07556	0.388533
1	-3.09228	0.331561
1	-3.10794	0.274096
1	-3.12253	0.216158
1	-3.13603	0.157767
1	-3.14845	0.0989439
1	-3.15975	0.0397087
1	-3.16994	-0.0199176
1	-3.179	-0.0799137
1	-3.18692	-0.140258
1	-3.19369	-0.20093
1	-3.1993	-0.261906
1	-3.20374	-0.323165
1	-3.20701	-0.384684

Cluster #	x-coordinates	y-coordinates
1	-3.2091	-0.446441
1	-3.20999	-0.508412
1	-3.20968	-0.570575
1	-3.20817	-0.632907
1	-3.20544	-0.695383
1	-3.20149	-0.757981
1	-3.19632	-0.820677
1	-3.18993	-0.883446
1	-3.18229	-0.946264
1	-3.17342	-1.00911
1	-3.16331	-1.07195
1	-3.15195	-1.13477
1	-3.13935	-1.19754
1	-3.12549	-1.26024
1	-3.11038	-1.32284
1	-3.09402	-1.38532
1	-3.07641	-1.44765
1	-3.05755	-1.50981
1	-3.03743	-1.57176
1	-3.01605	-1.6335
1	-2.99343	-1.69498
1	-2.96956	-1.75619
1	-2.94444	-1.8171
1	-2.91808	-1.87769
1	-2.89047	-1.93793
1	-2.86163	-1.99779
1	-2.83156	-2.05725
1	-2.80026	-2.11628
1	-2.76774	-2.17487
1	-2.734	-2.23297
1	-2.69905	-2.29058
1	-2.66289	-2.34766
1	-2.62555	-2.40418
1	-2.58701	-2.46014
1	-2.5473	-2.51549
1	-2.50641	-2.57021
1	-2.46437	-2.62429
1	-2.42118	-2.67769
1	-2.37684	-2.73039
1	-2.33138	-2.78237
1	-2.2848	-2.8336
1	-2.23711	-2.88407
1	-2.18833	-2.93374
1	-2.13847	-2.98259
1	-2.08755	-3.0306

Cluster #	x-coordinates	y-coordinates
1	-2.03557	-3.07775
1	-1.98256	-3.12401
1	-1.92852	-3.16937
1	-1.87348	-3.2138
1	-1.81744	-3.25727
1	-1.76043	-3.29977
1	-1.70246	-3.34127
1	-1.64356	-3.38176
1	-1.58372	-3.42122
1	-1.52299	-3.45961
1	-1.46136	-3.49693
1	-1.39887	-3.53315
1	-1.33554	-3.56826
1	-1.27137	-3.60222
1	-1.2064	-3.63504
1	-1.14064	-3.66668
1	-1.07412	-3.69713
1	-1.00685	-3.72637
1	-0.938859	-3.75439
1	-0.870171	-3.78116
1	-0.800806	-3.80668
1	-0.730787	-3.83092
1	-0.660139	-3.85387
1	-0.588884	-3.87551
1	-0.517048	-3.89584
1	-0.444654	-3.91483
1	-0.371728	-3.93247
1	-0.298294	-3.94875
1	-0.224379	-3.96365
1	-0.150007	-3.97717
1	-0.0752053	-3.98929
1	1.70E-14	-4
1	0.0755822	-4.00929
1	0.151515	-4.01714
1	0.22777	-4.02356
1	0.30432	-4.02852
1	0.381139	-4.03203
1	0.458197	-4.03406
1	0.535467	-4.03462
1	0.61292	-4.0337
1	0.690529	-4.03129
1	0.768263	-4.02738
1	0.846095	-4.02197
1	0.923996	-4.01505
1	1.00193	-4.00662

Cluster #	x-coordinates	y-coordinates
1	1.07988	-3.99668
1	1.15781	-3.98522
1	1.23569	-3.97224
1	1.31349	-3.95773
1	1.39119	-3.9417
1	1.46874	-3.92414
1	1.54612	-3.90506
1	1.62331	-3.88445
1	1.70027	-3.86232
1	1.77696	-3.83866
1	1.85337	-3.81348
1	1.92946	-3.78678
1	2.0052	-3.75856
1	2.08056	-3.72883
1	2.15551	-3.69759
1	2.23002	-3.66485
1	2.30406	-3.63061
1	2.37759	-3.59488
1	2.4506	-3.55766
1	2.52305	-3.51897
1	2.59491	-3.4788
1	2.66615	-3.43717
1	2.73673	-3.3941
1	2.80664	-3.34957
1	2.87585	-3.30362
1	2.94431	-3.25625
1	3.01201	-3.20746
1	3.07891	-3.15728
1	3.14499	-3.10571
1	3.21021	-3.05277
1	3.27456	-2.99848
1	3.33799	-2.94284
1	3.40049	-2.88587
1	3.46203	-2.82759
1	3.52257	-2.76801
1	3.5821	-2.70715
1	3.64058	-2.64503
1	3.69798	-2.58167
1	3.75429	-2.51708
1	3.80948	-2.45128
1	3.86351	-2.38429
1	3.91638	-2.31614
1	3.96804	-2.24684
1	4.01848	-2.17641
1	4.06767	-2.10488

Cluster #	x-coordinates	y-coordinates
1	4.11558	-2.03226
1	4.1622	-1.95858
1	4.20751	-1.88387
1	4.25147	-1.80815
1	4.29407	-1.73143
1	4.33529	-1.65375
1	4.3751	-1.57513
1	4.41348	-1.4956
1	4.45041	-1.41517
1	4.48588	-1.33389
1	4.51986	-1.25177
1	4.55234	-1.16884
1	4.58329	-1.08513
1	4.61271	-1.00067
1	4.64056	-0.915489
1	4.66683	-0.829609
1	4.69152	-0.743064
1	4.7146	-0.655882
1	4.73605	-0.568093
1	4.75587	-0.479729
1	4.77403	-0.390819
1	4.79053	-0.301394
1	4.80535	-0.211487
1	4.81848	-0.121127
1	4.8299	-0.0303476
1	4.83962	0.0608196
1	4.84761	0.152342
1	4.85386	0.244187
1	4.85837	0.336322
1	4.86113	0.428714
1	4.86213	0.521329
1	4.86136	0.614133
1	4.85882	0.707093
1	4.8545	0.800175
1	4.84838	0.893344
1	4.84048	0.986567
1	4.83079	1.07981
1	4.81929	1.17304
1	4.806	1.26621
1	4.7909	1.3593
1	4.77399	1.45227
1	4.75528	1.54508
1	4.73477	1.63771
1	4.71244	1.73011
1	4.68832	1.82225

Cluster #	x-coordinates	y-coordinates
1	4.66239	1.91409
1	4.63466	2.0056
1	4.60514	2.09674
1	4.57382	2.18748
1	4.54071	2.27779
1	4.50583	2.36762
1	4.46916	2.45694
1	4.43073	2.54573
1	4.39054	2.63393
1	4.34859	2.72152
1	4.30489	2.80847
1	4.25946	2.89473
1	4.21231	2.98028
1	4.16344	3.06507
1	4.11287	3.14908
1	4.06061	3.23227
1	4.00667	3.3146
1	3.95107	3.39605
1	3.89381	3.47658
1	3.83493	3.55616
1	3.77442	3.63474
1	3.71231	3.71231
1	3.64862	3.78883
1	3.58335	3.86426
1	3.51654	3.93857
1	3.4482	4.01173
1	3.37835	4.08372
1	3.307	4.1545
1	3.23419	4.22403
1	3.15993	4.29229
1	3.08424	4.35925
1	3.00715	4.42488
1	2.92867	4.48915
1	2.84885	4.55203
1	2.76768	4.61349
1	2.68522	4.67351
1	2.60147	4.73206
1	2.51647	4.7891
1	2.43024	4.84462
1	2.34281	4.89859
1	2.2542	4.95098
1	2.16446	5.00176
1	2.07359	5.05092
1	1.98165	5.09843
1	1.88864	5.14426

Cluster #	x-coordinates	y-coordinates
1	1.79462	5.1884
1	1.69959	5.23081
1	1.60361	5.27148
1	1.50669	5.31039
1	1.40888	5.34752
1	1.3102	5.38284
1	1.2107	5.41634
1	1.11039	5.44799
1	1.00932	5.47779
1	0.907515	5.50571
1	0.80502	5.53173
1	0.701866	5.55584
1	0.598089	5.57803
1	0.493724	5.59827
1	0.388808	5.61656
1	0.283378	5.63288
1	0.177471	5.64721
1	0.0711238	5.65955
1	-0.0356254	5.66989
1	-0.142739	5.67821
1	-0.250179	5.6845
1	-0.357906	5.68875
1	-0.465882	5.69096
1	-0.574069	5.69112
1	-0.682427	5.68922
1	-0.790916	5.68525
1	-0.899498	5.67921
1	-1.00813	5.67109
1	-1.11678	5.66089
1	-1.2254	5.64861
1	-1.33395	5.63424
1	-1.4424	5.61778
1	-1.5507	5.59923
1	-1.65881	5.5786
1	-1.7667	5.55587
1	-1.87431	5.53105
1	-1.98162	5.50415
1	-2.08857	5.47517
1	-2.19514	5.4441
1	-2.30128	5.41096
1	-2.40694	5.37575
1	-2.5121	5.33848
1	-2.6167	5.29915
1	-2.72071	5.25777
1	-2.82409	5.21435

Cluster #	x-coordinates	y-coordinates
1	-2.9268	5.16889
1	-3.0288	5.12142
1	-3.13004	5.07193
1	-3.23049	5.02044
1	-3.33012	4.96696
1	-3.42887	4.91151
1	-3.52671	4.8541
1	-3.62361	4.79474
1	-3.71951	4.73346
1	-3.8144	4.67025
1	-3.90822	4.60515
1	-4.00094	4.53817
1	-4.09252	4.46933
1	-4.18292	4.39864
1	-4.27211	4.32614
1	-4.36005	4.25183
1	-4.44671	4.17574
1	-4.53204	4.09789
1	-4.61602	4.0183
1	-4.6986	3.93701
1	-4.77976	3.85402
1	-4.85945	3.76938
1	-4.93765	3.68309
1	-5.01432	3.5952
1	-5.08943	3.50572
1	-5.16295	3.41469
1	-5.23483	3.32213
1	-5.30506	3.22807
1	-5.3736	3.13254
1	-5.44042	3.03557
1	-5.5055	2.93719
1	-5.56879	2.83744
1	-5.63028	2.73634
1	-5.68993	2.63394
1	-5.74771	2.53025
1	-5.80361	2.42532
1	-5.85759	2.31918
1	-5.90963	2.21187
1	-5.9597	2.10342
1	-6.00778	1.99386
1	-6.05384	1.88324
1	-6.09787	1.77159
1	-6.13983	1.65895
1	-6.17971	1.54536
1	-6.21748	1.43085

Cluster #	x-coordinates	y-coordinates
1	-6.25313	1.31546
1	-6.28664	1.19924
1	-6.31798	1.08222
1	-6.34714	0.964448
1	-6.37411	0.845959
1	-6.39886	0.726795
1	-6.42137	0.606999
1	-6.44165	0.486611
1	-6.45966	0.365675
1	-6.4754	0.244232
1	-6.48885	0.122326
2	-1.5	1.84E-16
2	-1.50973	-0.0284611
2	-1.51892	-0.0572891
2	-1.52755	-0.0864734
2	-1.53562	-0.116003
2	-1.54312	-0.145868
2	-1.55003	-0.176056
2	-1.55635	-0.206556
2	-1.56207	-0.237356
2	-1.56717	-0.268445
2	-1.57166	-0.29981
2	-1.57552	-0.331439
2	-1.57873	-0.363319
2	-1.58131	-0.395437
2	-1.58323	-0.42778
2	-1.58448	-0.460335
2	-1.58508	-0.493089
2	-1.58499	-0.526028
2	-1.58422	-0.559137
2	-1.58277	-0.592403
2	-1.58062	-0.625812
2	-1.57777	-0.659349
2	-1.57421	-0.692999
2	-1.56995	-0.726748
2	-1.56497	-0.760581
2	-1.55926	-0.794483
2	-1.55283	-0.828439
2	-1.54567	-0.862433
2	-1.53778	-0.896449
2	-1.52916	-0.930473
2	-1.51979	-0.964488
2	-1.50968	-0.998479
2	-1.49883	-1.03243
2	-1.48723	-1.06632

Cluster #	x-coordinates	y-coordinates
2	-1.47488	-1.10015
2	-1.46179	-1.13388
2	-1.44794	-1.16751
2	-1.43334	-1.20101
2	-1.41799	-1.23438
2	-1.40189	-1.2676
2	-1.38504	-1.30064
2	-1.36744	-1.3335
2	-1.34909	-1.36615
2	-1.32999	-1.39858
2	-1.31015	-1.43078
2	-1.28956	-1.46272
2	-1.26823	-1.49439
2	-1.24616	-1.52577
2	-1.22336	-1.55685
2	-1.19983	-1.58761
2	-1.17557	-1.61803
2	-1.15059	-1.6481
2	-1.12489	-1.6778
2	-1.09848	-1.70712
2	-1.07136	-1.73603
2	-1.04353	-1.76452
2	-1.01502	-1.79258
2	-0.985813	-1.82018
2	-0.955926	-1.84732
2	-0.925365	-1.87398
2	-0.894136	-1.90014
2	-0.86225	-1.92578
2	-0.829712	-1.95089
2	-0.796533	-1.97546
2	-0.762721	-1.99946
2	-0.728287	-2.02289
2	-0.693238	-2.04573
2	-0.657587	-2.06797
2	-0.621342	-2.08958
2	-0.584515	-2.11055
2	-0.547118	-2.13088
2	-0.509161	-2.15055
2	-0.470656	-2.16954
2	-0.431615	-2.18783
2	-0.392052	-2.20542
2	-0.351978	-2.2223
2	-0.311406	-2.23844
2	-0.270351	-2.25384
2	-0.228825	-2.26849

Cluster #	x-coordinates	y-coordinates
2	-0.186843	-2.28237
2	-0.144418	-2.29546
2	-0.101566	-2.30777
2	-0.0583018	-2.31927
2	-0.0146397	-2.32995
2	0.0294045	-2.33982
2	0.0738153	-2.34884
2	0.118577	-2.35702
2	0.163672	-2.36434
2	0.209086	-2.3708
2	0.254801	-2.37638
2	0.3008	-2.38108
2	0.347066	-2.38488
2	0.393582	-2.38778
2	0.44033	-2.38977
2	0.487292	-2.39085
2	0.534451	-2.391
2	0.581788	-2.39021
2	0.629284	-2.38849
2	0.676921	-2.38583
2	0.72468	-2.38221
2	0.772542	-2.37764
2	0.820489	-2.37211
2	0.8685	-2.36561
2	0.916557	-2.35814
2	0.964639	-2.3497
2	1.01273	-2.34027
2	1.0608	-2.32987
2	1.10884	-2.31848
2	1.15683	-2.30611
2	1.20474	-2.29275
2	1.25256	-2.2784
2	1.30026	-2.26305
2	1.34783	-2.24672
2	1.39524	-2.22939
2	1.44248	-2.21107
2	1.48952	-2.19176
2	1.53634	-2.17146
2	1.58293	-2.15017
2	1.62925	-2.12789
2	1.6753	-2.10463
2	1.72104	-2.08039
2	1.76647	-2.05516
2	1.81155	-2.02896
2	1.85627	-2.00179

Cluster #	x-coordinates	y-coordinates
2	1.90061	-1.97365
2	1.94454	-1.94454
2	1.98805	-1.91448
2	2.03112	-1.88347
2	2.07372	-1.85151
2	2.11583	-1.81862
2	2.15744	-1.78479
2	2.19852	-1.75003
2	2.23905	-1.71436
2	2.27902	-1.67778
2	2.3184	-1.64031
2	2.35718	-1.60194
2	2.39533	-1.56269
2	2.43284	-1.52257
2	2.46968	-1.48159
2	2.50583	-1.43975
2	2.54129	-1.39709
2	2.57602	-1.35359
2	2.61002	-1.30928
2	2.64325	-1.26417
2	2.67571	-1.21826
2	2.70738	-1.17159
2	2.73823	-1.12415
2	2.76825	-1.07596
2	2.79743	-1.02704
2	2.82574	-0.977395
2	2.85317	-0.927051
2	2.8797	-0.876019
2	2.90532	-0.824315
2	2.93001	-0.771955
2	2.95376	-0.718957
2	2.97655	-0.665337
2	2.99836	-0.611113
2	3.01918	-0.556302
2	3.03899	-0.500922
2	3.05779	-0.444993
2	3.07556	-0.388533
2	3.09228	-0.331561
2	3.10794	-0.274096
2	3.12253	-0.216158
2	3.13603	-0.157767
2	3.14845	-0.0989439
2	3.15975	-0.0397087
2	3.16994	0.0199176
2	3.179	0.0799137

Cluster #	x-coordinates	y-coordinates
2	3.18692	0.140258
2	3.19369	0.20093
2	3.1993	0.261906
2	3.20374	0.323165
2	3.20701	0.384684
2	3.2091	0.446441
2	3.20999	0.508412
2	3.20968	0.570575
2	3.20817	0.632907
2	3.20544	0.695383
2	3.20149	0.757981
2	3.19632	0.820677
2	3.18993	0.883446
2	3.18229	0.946264
2	3.17342	1.00911
2	3.16331	1.07195
2	3.15195	1.13477
2	3.13935	1.19754
2	3.12549	1.26024
2	3.11038	1.32284
2	3.09402	1.38532
2	3.07641	1.44765
2	3.05755	1.50981
2	3.03743	1.57176
2	3.01605	1.6335
2	2.99343	1.69498
2	2.96956	1.75619
2	2.94444	1.8171
2	2.91808	1.87769
2	2.89047	1.93793
2	2.86163	1.99779
2	2.83156	2.05725
2	2.80026	2.11628
2	2.76774	2.17487
2	2.734	2.23297
2	2.69905	2.29058
2	2.66289	2.34766
2	2.62555	2.40418
2	2.58701	2.46014
2	2.5473	2.51549
2	2.50641	2.57021
2	2.46437	2.62429
2	2.42118	2.67769
2	2.37684	2.73039
2	2.33138	2.78237

Cluster #	x-coordinates	y-coordinates
2	2.2848	2.8336
2	2.23711	2.88407
2	2.18833	2.93374
2	2.13847	2.98259
2	2.08755	3.0306
2	2.03557	3.07775
2	1.98256	3.12401
2	1.92852	3.16937
2	1.87348	3.2138
2	1.81744	3.25727
2	1.76043	3.29977
2	1.70246	3.34127
2	1.64356	3.38176
2	1.58372	3.42122
2	1.52299	3.45961
2	1.46136	3.49693
2	1.39887	3.53315
2	1.33554	3.56826
2	1.27137	3.60222
2	1.2064	3.63504
2	1.14064	3.66668
2	1.07412	3.69713
2	1.00685	3.72637
2	0.938859	3.75439
2	0.870171	3.78116
2	0.800806	3.80668
2	0.730787	3.83092
2	0.660139	3.85387
2	0.588884	3.87551
2	0.517048	3.89584
2	0.444654	3.91483
2	0.371728	3.93247
2	0.298294	3.94875
2	0.224379	3.96365
2	0.150007	3.97717
2	0.0752053	3.98929
2	2.04E-13	4
2	-0.0755822	4.00929
2	-0.151515	4.01714
2	-0.22777	4.02356
2	-0.30432	4.02852
2	-0.381139	4.03203
2	-0.458197	4.03406
2	-0.535467	4.03462
2	-0.61292	4.0337

Cluster #	x-coordinates	y-coordinates
2	-0.690529	4.03129
2	-0.768263	4.02738
2	-0.846095	4.02197
2	-0.923996	4.01505
2	-1.00193	4.00662
2	-1.07988	3.99668
2	-1.15781	3.98522
2	-1.23569	3.97224
2	-1.31349	3.95773
2	-1.39119	3.9417
2	-1.46874	3.92414
2	-1.54612	3.90506
2	-1.62331	3.88445
2	-1.70027	3.86232
2	-1.77696	3.83866
2	-1.85337	3.81348
2	-1.92946	3.78678
2	-2.0052	3.75856
2	-2.08056	3.72883
2	-2.15551	3.69759
2	-2.23002	3.66485
2	-2.30406	3.63061
2	-2.37759	3.59488
2	-2.4506	3.55766
2	-2.52305	3.51897
2	-2.59491	3.4788
2	-2.66615	3.43717
2	-2.73673	3.3941
2	-2.80664	3.34957
2	-2.87585	3.30362
2	-2.94431	3.25625
2	-3.01201	3.20746
2	-3.07891	3.15728
2	-3.14499	3.10571
2	-3.21021	3.05277
2	-3.27456	2.99848
2	-3.33799	2.94284
2	-3.40049	2.88587
2	-3.46203	2.82759
2	-3.52257	2.76801
2	-3.5821	2.70715
2	-3.64058	2.64503
2	-3.69798	2.58167
2	-3.75429	2.51708
2	-3.80948	2.45128

Cluster #	x-coordinates	y-coordinates
2	-3.86351	2.38429
2	-3.91638	2.31614
2	-3.96804	2.24684
2	-4.01848	2.17641
2	-4.06767	2.10488
2	-4.11558	2.03226
2	-4.1622	1.95858
2	-4.20751	1.88387
2	-4.25147	1.80815
2	-4.29407	1.73143
2	-4.33529	1.65375
2	-4.3751	1.57513
2	-4.41348	1.4956
2	-4.45041	1.41517
2	-4.48588	1.33389
2	-4.51986	1.25177
2	-4.55234	1.16884
2	-4.58329	1.08513
2	-4.61271	1.00067
2	-4.64056	0.915489
2	-4.66683	0.829609
2	-4.69152	0.743064
2	-4.7146	0.655882
2	-4.73605	0.568093
2	-4.75587	0.479729
2	-4.77403	0.390819
2	-4.79053	0.301394
2	-4.80535	0.211487
2	-4.81848	0.121127
2	-4.8299	0.0303476
2	-4.83962	-0.0608196
2	-4.84761	-0.152342
2	-4.85386	-0.244187
2	-4.85837	-0.336322
2	-4.86113	-0.428714
2	-4.86213	-0.521329
2	-4.86136	-0.614133
2	-4.85882	-0.707093
2	-4.8545	-0.800175
2	-4.84838	-0.893344
2	-4.84048	-0.986567
2	-4.83079	-1.07981
2	-4.81929	-1.17304
2	-4.806	-1.26621
2	-4.7909	-1.3593

Cluster #	x-coordinates	y-coordinates
2	-4.77399	-1.45227
2	-4.75528	-1.54508
2	-4.73477	-1.63771
2	-4.71244	-1.73011
2	-4.68832	-1.82225
2	-4.66239	-1.91409
2	-4.63466	-2.0056
2	-4.60514	-2.09674
2	-4.57382	-2.18748
2	-4.54071	-2.27779
2	-4.50583	-2.36762
2	-4.46916	-2.45694
2	-4.43073	-2.54573
2	-4.39054	-2.63393
2	-4.34859	-2.72152
2	-4.30489	-2.80847
2	-4.25946	-2.89473
2	-4.21231	-2.98028
2	-4.16344	-3.06507
2	-4.11287	-3.14908
2	-4.06061	-3.23227
2	-4.00667	-3.3146
2	-3.95107	-3.39605
2	-3.89381	-3.47658
2	-3.83493	-3.55616
2	-3.77442	-3.63474
2	-3.71231	-3.71231
2	-3.64862	-3.78883
2	-3.58335	-3.86426
2	-3.51654	-3.93857
2	-3.4482	-4.01173
2	-3.37835	-4.08372
2	-3.307	-4.1545
2	-3.23419	-4.22403
2	-3.15993	-4.29229
2	-3.08424	-4.35925
2	-3.00715	-4.42488
2	-2.92867	-4.48915
2	-2.84885	-4.55203
2	-2.76768	-4.61349
2	-2.68522	-4.67351
2	-2.60147	-4.73206
2	-2.51647	-4.7891
2	-2.43024	-4.84462
2	-2.34281	-4.89859

Cluster #	x-coordinates	y-coordinates
2	-2.2542	-4.95098
2	-2.16446	-5.00176
2	-2.07359	-5.05092
2	-1.98165	-5.09843
2	-1.88864	-5.14426
2	-1.79462	-5.1884
2	-1.69959	-5.23081
2	-1.60361	-5.27148
2	-1.50669	-5.31039
2	-1.40888	-5.34752
2	-1.3102	-5.38284
2	-1.2107	-5.41634
2	-1.11039	-5.44799
2	-1.00932	-5.47779
2	-0.907515	-5.50571
2	-0.80502	-5.53173
2	-0.701866	-5.55584
2	-0.598089	-5.57803
2	-0.493724	-5.59827
2	-0.388808	-5.61656
2	-0.283378	-5.63288
2	-0.177471	-5.64721
2	-0.0711238	-5.65955
2	0.0356254	-5.66989
2	0.142739	-5.67821
2	0.250179	-5.6845
2	0.357906	-5.68875
2	0.465882	-5.69096
2	0.574069	-5.69112
2	0.682427	-5.68922
2	0.790916	-5.68525
2	0.899498	-5.67921
2	1.00813	-5.67109
2	1.11678	-5.66089
2	1.2254	-5.64861
2	1.33395	-5.63424
2	1.4424	-5.61778
2	1.5507	-5.59923
2	1.65881	-5.5786
2	1.7667	-5.55587
2	1.87431	-5.53105
2	1.98162	-5.50415
2	2.08857	-5.47517
2	2.19514	-5.4441
2	2.30128	-5.41096

Cluster #	x-coordinates	y-coordinates
2	2.40694	-5.37575
2	2.5121	-5.33848
2	2.6167	-5.29915
2	2.72071	-5.25777
2	2.82409	-5.21435
2	2.9268	-5.16889
2	3.0288	-5.12142
2	3.13004	-5.07193
2	3.23049	-5.02044
2	3.33012	-4.96696
2	3.42887	-4.91151
2	3.52671	-4.8541
2	3.62361	-4.79474
2	3.71951	-4.73346
2	3.8144	-4.67025
2	3.90822	-4.60515
2	4.00094	-4.53817
2	4.09252	-4.46933
2	4.18292	-4.39864
2	4.27211	-4.32614
2	4.36005	-4.25183
2	4.44671	-4.17574
2	4.53204	-4.09789
2	4.61602	-4.0183
2	4.6986	-3.93701
2	4.77976	-3.85402
2	4.85945	-3.76938
2	4.93765	-3.68309
2	5.01432	-3.5952
2	5.08943	-3.50572
2	5.16295	-3.41469
2	5.23483	-3.32213
2	5.30506	-3.22807
2	5.3736	-3.13254
2	5.44042	-3.03557
2	5.5055	-2.93719
2	5.56879	-2.83744
2	5.63028	-2.73634
2	5.68993	-2.63394
2	5.74771	-2.53025
2	5.80361	-2.42532
2	5.85759	-2.31918
2	5.90963	-2.21187
2	5.9597	-2.10342
2	6.00778	-1.99386

Cluster #	x-coordinates	y-coordinates
2	6.05384	-1.88324
2	6.09787	-1.77159
2	6.13983	-1.65895
2	6.17971	-1.54536
2	6.21748	-1.43085
2	6.25313	-1.31546
2	6.28664	-1.19924
2	6.31798	-1.08222
2	6.34714	-0.964448
2	6.37411	-0.845959
2	6.39886	-0.726795
2	6.42137	-0.606999
2	6.44165	-0.486611
2	6.45966	-0.365675
2	6.4754	-0.244232
2	6.48885	-0.122326

Appendix K

Square1
Number of dimensions: 2
Number of clusters: 4
Number of points: 1000

Cluster #	x-coordinates	y-coordinates
1	10.9185	4.88961
1	12.4454	9.74354
1	11.4869	8.48109
1	11.3589	10.262
1	9.15449	10.7772
1	13.8943	10.2023
1	10.3089	7.8211
1	10.0735	8.47487
1	10.5972	11.5332
1	9.58001	7.18364
1	9.28254	10.6751
1	6.5212	7.42879
1	12.7217	10.5749
1	12.4021	10.4588
1	9.99241	9.66111
1	11.121	8.4455
1	11.2099	11.4847
1	12.7127	11.624
1	6.53131	9.24825
1	8.11771	9.09367
1	13.286	8.09741
1	10.2191	10.1657
1	10.8266	8.60227
1	11.5165	9.85531
1	9.69942	9.56236
1	8.16903	10.7732
1	10.179	9.25451
1	9.09495	6.61754
1	4.18748	6.78161
1	12.4226	12.2299
1	13.7967	13.5089
1	10.5839	10.253
1	8.45717	10.6407
1	9.86273	11.203
1	8.78558	6.36908
1	11.3199	8.28916
1	9.22719	10.2892
1	12.4162	13.0268
1	10.402	8.85605

Cluster #	x-coordinates	y-coordinates
1	9.7351	14.328
1	10.1145	11.9807
1	12.4463	9.06225
1	8.3765	9.48224
1	6.94754	9.49249
1	10.8378	10.2192
1	9.19329	11.7556
1	8.70646	7.89747
1	10.6373	12.5112
1	9.89848	11.646
1	11.4446	8.1667
1	12.4901	7.3439
1	9.5061	10.7594
1	9.1052	10.5169
1	14.1729	7.14161
1	11.8023	9.61588
1	8.56484	10.9257
1	12.3523	6.37696
1	7.9028	9.08173
1	7.19313	11.8559
1	16.7028	11.3589
1	11.1384	7.22742
1	7.89456	10.8693
1	10.7093	9.50479
1	12.0348	5.98741
1	8.92957	8.90973
1	12.7134	12.8592
1	11.3752	11.1388
1	8.46402	13.7425
1	6.83641	8.54696
1	9.46343	9.97334
1	12.633	9.72559
1	10.6906	10.3299
1	8.07509	9.7056
1	10.0057	10.9791
1	10.9241	9.30696
1	8.25023	8.56504
1	12.1739	11.0418
1	8.7906	8.13353
1	11.1907	10.9506
1	12.1365	10.5123
1	10.4229	9.21595
1	7.53417	7.18743
1	7.2151	11.9754
1	8.39553	7.9911

Cluster #	x-coordinates	y-coordinates
1	10.3944	8.02614
1	9.03014	8.58279
1	13.3067	9.965
1	10.5762	9.00645
1	10.3504	13.0567
1	9.85428	11.0818
1	8.57604	8.5872
1	9.99609	10.9213
1	8.90139	12.921
1	11.7455	7.49012
1	11.5386	10.2637
1	11.2769	8.38929
1	10.2943	10.2706
1	13.4382	8.77768
1	8.74918	9.67784
1	10.3784	8.58741
1	6.65601	10.9838
1	11.4607	8.42992
1	9.37885	7.61578
1	12.0032	9.98512
1	11.5282	11.4595
1	8.0266	12.2569
1	9.01588	8.04251
1	8.86841	8.75967
1	5.1908	10.7109
1	11.0584	11.393
1	10.1835	7.61699
1	9.90201	10.7604
1	11.853	11.3909
1	8.67662	10.8684
1	10.2428	10.6068
1	11.0021	8.40108
1	11.235	9.89733
1	9.336	13.571
1	11.7275	8.1497
1	8.36691	7.31628
1	10.5679	7.91751
1	9.26132	8.9756
1	11.4246	8.60898
1	11.0692	10.5986
1	9.75679	7.9155
1	6.09359	11.5285
1	10.937	10.334
1	6.82371	7.21379
1	10.5044	11.3444

Cluster #	x-coordinates	y-coordinates
1	5.37289	11.2321
1	9.16776	10.4607
1	10.8287	11.3208
1	8.16338	9.29921
1	10.2184	8.30646
1	10.2308	10.8904
1	9.54046	14.0261
1	8.68443	9.61347
1	11.0875	11.8076
1	12.4552	12.4311
1	9.91185	8.35943
1	15.1861	9.35048
1	8.60526	9.04205
1	10.2579	8.97063
1	10.2763	11.6383
1	8.52845	10.8047
1	10.0433	8.90566
1	11.1187	7.14314
1	10.9113	11.6067
1	14.0012	7.21181
1	13.2952	11.5183
1	8.02287	10.7111
1	8.41754	9.31711
1	12.2152	9.79102
1	5.40527	13.0609
1	10.2841	10.4977
1	7.41771	8.3087
1	7.48926	12.6038
1	6.254	8.27339
1	5.53483	10.4752
1	5.88257	6.80295
1	10.342	12.3517
1	10.7234	6.68882
1	7.59847	8.19143
1	6.68833	9.05672
1	10.1462	10.3754
1	9.47389	7.44517
1	11.6497	12.4336
1	10.1907	9.02386
1	8.37762	9.8629
1	7.08602	8.06749
1	8.998	11.0552
1	11.1943	10.3176
1	9.01295	8.65259
1	9.13298	12.172

Cluster #	x-coordinates	y-coordinates
1	8.67291	12.6823
1	11.3722	9.33004
1	8.1578	7.14484
1	13.673	10.2271
1	6.46028	8.17373
1	8.92991	13.3195
1	8.8441	11.1652
1	7.99194	12.4253
1	9.43326	11.2891
1	9.57835	13.299
1	9.26627	9.12364
1	10.3556	11.7023
1	7.79118	11.9497
1	9.78484	10.7511
1	8.61664	8.78066
1	7.12256	8.51976
1	13.191	11.3626
1	7.52309	11.0431
1	9.90783	10.5621
1	11.2209	11.7377
1	10.4045	10.0534
1	8.35083	10.7356
1	11.8189	8.3248
1	9.65417	10.316
1	6.36656	12.0772
1	12.2057	8.49489
1	13.3787	12.5199
1	9.83828	9.4311
1	10.5118	10.148
1	9.89127	11.0474
1	9.0202	9.20737
1	10.3215	12.1823
1	10.1894	9.61348
1	9.022	8.09583
1	13.2511	8.7982
1	8.18689	10.6024
1	13.0244	10.3577
1	11.1224	8.13541
1	11.92	10.9393
1	7.96652	10.873
1	14.0068	11.5287
1	9.98768	13.438
1	7.85474	10.8648
1	10.7001	10.8782
1	10.0383	7.71143

Cluster #	x-coordinates	y-coordinates
1	11.2328	9.619
1	10.9805	8.98496
1	11.8132	8.14983
1	10.7504	11.2916
1	11.4023	9.83219
1	10.3094	10.6567
1	8.79311	9.71932
1	7.64558	9.75415
1	10.4872	11.9422
1	8.29856	14.7114
1	8.75375	7.58698
1	11.357	9.48912
1	10.6761	9.71997
1	9.81114	8.11375
1	14.4588	9.68787
1	6.0314	9.3301
1	6.91596	6.8957
1	11.3476	5.85833
1	9.61397	7.93544
1	9.14186	12.8408
1	6.81022	8.22929
1	8.08123	9.74728
1	8.31361	12.2509
1	8.63342	7.4519
1	9.06051	9.73406
1	9.51886	9.15221
1	7.52136	11.0996
1	10.0545	9.99982
1	9.56766	11.6414
1	9.22846	7.67221
1	6.60602	10.4298
2	12.371	2.96273
2	11.7958	-2.34756
2	11.9746	-5.31279
2	11.0853	1.93015
2	10.7776	-1.31126
2	11.3691	-0.644471
2	8.52678	0.373238
2	13.7181	1.15121
2	10.5381	3.03549
2	4.78822	0.942808
2	9.41647	1.45347
2	13.5366	0.507458
2	8.85808	1.48022
2	12.1654	1.16551

Cluster #	x-coordinates	y-coordinates
2	7.37836	2.62061
2	11.8862	1.40461
2	10.9516	0.498884
2	8.93189	1.64696
2	8.49196	2.21685
2	9.78405	2.45445
2	9.48588	-0.431642
2	8.85899	0.920064
2	12.9534	3.56249
2	10.1578	2.59515
2	9.78185	0.390867
2	8.95658	3.24879
2	9.31281	-3.32291
2	10.2129	-0.395405
2	9.90687	4.59105
2	10.8204	0.532447
2	12.5477	-0.372323
2	12.2403	5.78334
2	9.49063	-0.237021
2	6.16308	0.742657
2	6.76808	0.471571
2	10.1108	-1.53863
2	9.24716	-0.652237
2	8.76129	-0.792335
2	7.92727	1.18063
2	7.75802	0.511433
2	5.77874	1.14418
2	10.8841	-1.09589
2	13.5731	1.4455
2	11.3429	1.42026
2	12.0738	-0.469501
2	9.90524	0.730892
2	7.55212	-3.1374
2	9.99856	-1.03417
2	7.11444	3.03787
2	12.5967	0.586176
2	11.6312	-0.966577
2	8.36029	-0.537471
2	8.24777	0.320897
2	14.1375	1.51693
2	11.7898	-0.376756
2	10.7052	0.764287
2	8.70778	0.541311
2	13.2404	-0.0800998
2	11.0294	-0.760503

Cluster #	x-coordinates	y-coordinates
2	10.9064	2.95206
2	12.5757	-0.240023
2	12.6854	-2.69425
2	14.5983	-0.283076
2	7.91285	-1.68329
2	10.4494	0.796312
2	12.2508	-2.00946
2	7.77488	1.3142
2	9.06975	-0.919119
2	7.05533	2.10359
2	6.1787	0.707323
2	10.5739	-0.766941
2	13.2178	-0.63944
2	10.7489	-0.507383
2	6.91794	1.98479
2	10.5502	0.134734
2	10.0029	0.595259
2	8.67716	1.69273
2	8.43697	-0.511461
2	11.7998	0.587468
2	12.4222	-0.297002
2	9.65957	2.73434
2	12.8636	-2.42426
2	12.0435	2.34016
2	11.8589	2.14587
2	11.1812	-3.4744
2	10.1304	2.83904
2	8.04948	0.288088
2	11.0909	0.547005
2	11.0714	-0.0311196
2	7.08568	0.426729
2	8.8258	1.74172
2	10.4076	2.11596
2	10.1079	-0.913192
2	8.91176	1.90963
2	10.5085	-1.80505
2	12.3322	2.58652
2	8.65378	-1.00673
2	11.5978	1.3469
2	7.56663	2.90909
2	8.77181	-2.26187
2	7.38416	1.60863
2	7.56918	-0.345081
2	5.92695	2.86353
2	8.45666	2.03882

Cluster #	x-coordinates	y-coordinates
2	11.3333	-0.812984
2	10.9286	-0.361394
2	8.23711	-1.01724
2	9.17354	-1.39034
2	10.6565	-3.98565
2	9.09873	1.85072
2	11.9607	-0.398544
2	9.7646	-1.3707
2	13.0265	-1.36261
2	11.7155	-0.599175
2	11.0173	1.24624
2	8.74608	2.31239
2	6.30942	0.389838
2	10.2939	-0.11942
2	15.9212	-2.03146
2	8.13929	2.76128
2	11.2779	-0.0839633
2	6.84372	0.977867
2	8.92066	-0.534774
2	8.36797	-1.45361
2	9.04556	-1.23981
2	11.5376	1.17522
2	8.06876	-2.18949
2	10.6905	5.38786
2	9.92873	-1.72175
2	9.91902	1.39307
2	10.3919	1.18253
2	10.1683	-0.306947
2	10.2402	-2.13139
2	9.7396	2.45212
2	7.96767	0.499569
2	8.94364	-1.75797
2	10.9281	0.0367325
2	9.66533	0.302059
2	5.8071	-0.259447
2	12.4322	0.613576
2	8.68954	-0.84996
2	13.2126	0.0787014
2	9.93866	4.37041
2	5.7992	4.34671
2	9.6144	3.14346
2	10.995	-0.325482
2	11.2118	-4.15801
2	13.9113	-3.74649
2	9.39227	-2.49996

Cluster #	x-coordinates	y-coordinates
2	12.7561	2.17177
2	12.3668	1.62641
2	5.79292	-2.37974
2	8.82837	4.25295
2	13.7533	1.55443
2	9.15851	1.40395
2	6.35618	-1.23335
2	9.19889	-2.63115
2	6.81934	0.0890129
2	8.51946	-1.00281
2	11.1576	0.516517
2	9.079	1.07582
2	9.96503	2.59539
2	13.1738	2.08507
2	10.4733	-2.32462
2	9.12768	1.34031
2	9.62287	-0.151438
2	10.8324	-0.00620798
2	8.64032	-0.842306
2	10.4526	3.81555
2	5.98352	-0.929479
2	7.69741	0.420563
2	7.32635	4.68164
2	9.87256	-1.99093
2	10.0954	-2.51352
2	13.7498	-3.10865
2	6.71656	0.67455
2	14.2027	-2.33185
2	7.86611	-2.46522
2	5.85567	0.804827
2	14.6136	-1.96284
2	9.02734	0.760524
2	10.7874	3.24377
2	9.88269	-0.587611
2	8.10605	0.996171
2	12.6332	-1.12926
2	6.08146	0.995985
2	5.80939	0.567642
2	10.2077	-3.53096
2	12.8018	-1.09919
2	9.09731	1.2086
2	5.77656	-0.965842
2	10.607	-2.53292
2	11.3875	0.861257
2	12.9219	-0.877962

Cluster #	x-coordinates	y-coordinates
2	9.39697	-0.0301992
2	9.88439	-2.31018
2	9.19941	1.82527
2	7.26177	-2.33739
2	10.5754	-1.49528
2	9.73292	0.851338
2	8.27577	-1.68224
2	7.86655	2.16924
2	11.2704	1.25582
2	9.38803	0.357686
2	12.1408	-0.78988
2	10.599	-0.449738
2	7.13488	-3.94985
2	10.9019	-2.62262
2	10.8926	-1.00075
2	10.2539	3.2753
2	10.072	2.09315
2	6.1381	-3.93668
2	9.19718	0.2157
2	9.82559	1.71255
2	8.47976	-2.07989
2	12.5267	1.79982
2	11.8944	0.560019
2	10.614	-4.43962
2	12.2633	0.582751
2	8.03444	-0.226748
2	15.2042	-0.48522
2	13.4551	-0.718799
2	7.0912	-0.62014
2	11.1606	-2.12069
2	6.3125	3.07961
2	7.82762	4.06849
2	10.6624	-4.34198
2	15.081	0.135357
2	6.52392	-3.20587
2	6.67838	0.0168515
2	11.8538	-1.01302
2	10.103	-0.766667
2	11.6739	0.150377
2	8.61486	1.94783
2	9.65825	-3.99091
2	9.21178	-3.89232
2	9.68887	3.25673
2	8.17207	0.934164
2	9.53159	-2.48335

Cluster #	x-coordinates	y-coordinates
2	12.8144	0.802428
2	10.1157	-2.26044
2	6.88615	3.26866
2	7.7017	0.402345
2	11.06	-1.10844
2	9.42306	3.4284
2	3.76444	2.87711
2	11.8669	-0.383389
2	9.21763	-2.9053
2	10.3333	-0.510629
2	6.12132	-0.0812959
3	0.306727	11.3151
3	5.17095	11.8551
3	1.99612	10.1973
3	1.07188	12.9116
3	-1.66212	10.6478
3	0.801202	10.97
3	-0.885348	10.9521
3	1.92524	7.1356
3	-5.4558	10.9298
3	2.80243	6.52139
3	1.76759	10.3342
3	1.71136	13.1241
3	-1.94153	10.3676
3	2.10267	10.1316
3	1.02853	8.14145
3	3.0764	10.1784
3	-2.62075	9.62061
3	-0.63794	10.0672
3	2.42675	11.4808
3	4.43748	9.64634
3	0.482585	8.84522
3	1.22922	9.17825
3	1.35585	7.61936
3	1.87579	5.6514
3	-0.971684	8.63478
3	1.60785	8.23435
3	3.37413	7.67697
3	1.29928	9.74252
3	2.94512	7.10243
3	-0.978036	9.55965
3	-1.59675	9.3973
3	-0.645095	5.69896
3	-1.71631	10.741
3	-0.796782	8.74937

Cluster #	x-coordinates	y-coordinates
3	-0.715332	9.17189
3	-1.13665	8.55101
3	1.66351	10.8277
3	-2.4871	10.8462
3	0.246919	8.00032
3	-0.541447	11.2129
3	1.78654	12.9312
3	-0.477897	10.9808
3	-3.36777	9.2118
3	2.90405	9.06242
3	-1.60348	10.628
3	3.12756	13.2453
3	1.45507	9.76387
3	2.2268	10.3585
3	2.34761	10.5459
3	-0.830344	9.48598
3	1.32246	10.3718
3	-0.167482	8.64784
3	-1.62346	9.99168
3	0.786088	8.09545
3	-1.71132	10.7998
3	-3.22486	12.0369
3	0.865686	8.15951
3	-0.202484	8.13487
3	1.25544	10.671
3	-1.17296	9.71721
3	0.103919	8.89752
3	3.05779	10.7905
3	-0.348379	8.5548
3	-0.0596768	7.62843
3	-2.14692	11.8564
3	-0.751996	11.5251
3	1.35246	11.5091
3	4.39452	8.90047
3	3.62686	12.8166
3	-0.412103	7.90307
3	-1.98189	12.2674
3	-2.30689	8.83601
3	-4.93527	8.05734
3	2.17365	10.0752
3	2.16128	13.5563
3	-0.949961	12.1505
3	-4.73646	9.35719
3	-0.961917	5.90238
3	0.544097	8.02255

Cluster #	x-coordinates	y-coordinates
3	2.39786	9.78454
3	1.05236	9.77875
3	-0.753827	7.10096
3	0.980346	9.69457
3	0.742897	9.05421
3	0.176966	8.64468
3	-1.28322	11.8564
3	1.06578	7.02501
3	-2.61743	9.17887
3	-1.70915	8.95119
3	1.1219	11.2286
3	-1.34688	9.9333
3	0.115591	11.4972
3	3.16782	11.6729
3	0.976487	9.82648
3	2.48833	11.8771
3	-0.945834	11.0138
3	-1.27483	10.0699
3	-0.167336	10.0242
3	3.21458	12.2709
3	1.05938	10.7663
3	-2.10977	10.8653
3	-0.42784	9.97796
3	-2.79222	11.1383
3	-0.281742	11.1186
3	2.95228	10.8289
3	-0.926795	10.2335
3	1.43314	9.75471
3	0.689115	12.7764
3	0.118	9.1039
3	0.447505	8.90247
3	2.8698	8.06393
3	-1.5733	8.07759
3	-2.90204	12.8213
3	2.44129	10.9938
3	0.0641878	5.79399
3	-1.69653	14.2
3	-0.955837	13.4598
3	-1.08431	10.9864
3	-0.117569	7.94299
3	-4.06032	9.0222
3	1.81456	13.6427
3	-0.707646	8.5557
3	-0.683905	9.65549
3	1.89757	8.44515

Cluster #	x-coordinates	y-coordinates
3	1.67624	11.177
3	-0.126571	8.5235
3	-0.0915738	7.84639
3	-1.91857	9.07972
3	2.7052	14.6477
3	-1.157	9.83488
3	-1.66828	9.49956
3	3.06442	9.86266
3	1.69668	6.34806
3	0.67713	9.50936
3	0.930836	12.1133
3	-2.0641	11.2468
3	-0.511481	11.0022
3	2.77044	10.8145
3	2.66418	10.533
3	-2.4552	12.8073
3	-1.27476	9.77471
3	1.60139	9.11191
3	-0.0343294	12.1436
3	-0.30706	7.01766
3	-1.75158	11.1528
3	0.684455	10.4285
3	0.252376	11.2236
3	-1.51744	11.9894
3	-2.9473	9.80743
3	-1.90381	8.88197
3	2.1115	10.5354
3	-2.63491	8.92244
3	-2.51969	11.9881
3	0.968859	9.87595
3	-0.138175	7.91574
3	2.18976	12.5286
3	-2.04975	9.01558
3	-1.24025	11.4334
3	0.920658	7.79629
3	-0.589995	10.2247
3	2.02086	7.9558
3	1.41767	11.2921
3	0.556434	8.95105
3	-1.12352	12.6208
3	1.7047	9.94791
3	-0.963134	8.5293
3	-1.76121	11.1334
3	-0.190441	10.3843
3	-1.33417	12.2909

Cluster #	x-coordinates	y-coordinates
3	4.04703	9.66569
3	0.291194	7.60402
3	1.17534	6.86204
3	1.74836	9.95608
3	5.52147	12.5317
3	-2.18494	11.0113
3	0.473845	10.0556
3	1.34216	7.28412
3	-3.98026	8.08608
3	-0.531769	6.35919
3	1.64738	11.9501
3	-0.891816	9.16533
3	1.68609	7.13071
3	0.929372	10.3065
3	0.0161873	11.4468
3	0.489532	9.46746
3	2.04614	11.6187
3	-4.96524	12.5142
3	1.02982	13.1379
3	1.76779	8.00182
3	-1.57595	10.6107
3	3.9813	9.72535
3	0.129431	10.0423
3	2.5541	13.3272
3	-2.44088	11.3093
3	2.92646	13.6779
3	1.84335	7.38666
3	-0.151517	11.3069
3	1.66291	8.18913
3	-2.34819	10.1107
3	1.10125	9.98357
3	-2.43963	7.43744
3	-5.0028	9.90037
3	0.697787	9.63589
3	2.36387	10.8444
3	-0.856124	12.6846
3	-1.04237	9.16465
3	2.95895	9.99118
3	0.173674	12.8203
3	1.42369	6.35965
3	1.56401	7.07156
3	-1.32272	8.97541
3	-0.880111	8.88475
3	0.946746	11.9691
3	-4.53819	9.21133

Cluster #	x-coordinates	y-coordinates
3	-4.79167	7.96302
3	0.721291	13.4869
3	-0.204916	11.0078
3	-2.24044	11.4657
3	-0.164427	11.8221
3	1.30293	7.96546
3	3.52054	10.6916
3	-2.86996	11.1593
3	3.60818	14.0723
3	0.590331	4.37577
3	0.562473	10.2794
3	2.92563	11.1238
3	-0.800308	8.14481
3	1.77339	11.8911
3	-1.11164	12.0262
3	1.07244	10.1226
3	1.01521	8.88586
3	1.08527	10.2897
3	-3.00708	10.653
3	-0.84521	11.4484
3	1.51073	12.1335
3	-2.66857	12.7899
3	2.67731	8.9268
3	1.05393	6.00194
3	-3.947	8.00537
3	-0.594244	12.4896
3	-0.297333	9.34092
3	-3.27633	10.9531
3	4.38608	10.9294
3	0.055897	9.56236
3	3.16388	10.0926
3	-0.396562	9.02468
3	3.43302	10.6813
3	2.31897	9.74396
3	1.28836	9.32739
3	-0.94443	12.3045
4	-2.24791	-3.91167
4	0.974215	-0.91293
4	1.38634	-2.05746
4	1.74525	-0.901507
4	-0.677591	-1.18781
4	-0.585782	-1.13773
4	-0.0564082	-1.93547
4	-1.45873	-0.98357
4	1.35238	2.91938

Cluster #	x-coordinates	y-coordinates
4	-3.3444	2.50484
4	0.713283	-0.768338
4	2.46635	3.75131
4	-0.776254	1.29345
4	0.387304	-0.00258549
4	2.3451	-2.90507
4	-0.797118	-2.04371
4	0.29915	-0.293339
4	-1.83045	1.48903
4	2.06028	-0.521355
4	-1.29541	-2.68804
4	-2.35397	-2.24686
4	-3.75267	0.084538
4	1.85227	1.55483
4	0.523157	-2.11477
4	-0.117425	-1.28357
4	3.85955	0.389026
4	-0.90518	0.397938
4	0.398389	-0.357314
4	-1.03842	-2.58305
4	0.0382496	1.20784
4	0.375385	-3.48195
4	-0.335926	-3.53812
4	-2.22178	0.531497
4	0.446128	-1.6669
4	-1.06972	0.711426
4	2.38231	1.2592
4	1.42659	0.623314
4	-2.411	-0.844719
4	1.22061	-0.463149
4	-0.633067	-3.2038
4	2.20366	-3.28039
4	-1.63044	-5.48374
4	-1.51903	2.79274
4	3.48752	-0.9434
4	-2.39604	-3.0276
4	0.985972	0.940161
4	1.41234	0.747235
4	-4.02492	-2.4934
4	-0.663163	1.87645
4	1.82018	-0.593457
4	-1.48968	-2.41517
4	0.423447	-2.54266
4	0.763979	-3.42971
4	2.70825	-0.17633

Cluster #	x-coordinates	y-coordinates
4	3.05168	3.53295
4	3.91511	1.83205
4	-0.678648	-1.44582
4	1.37317	-1.12811
4	-2.40325	3.84042
4	-1.07086	3.09118
4	-0.980645	-2.43516
4	0.181473	-2.23421
4	2.17171	1.96665
4	1.11414	-2.20965
4	-0.287849	-3.16407
4	2.13601	-1.36323
4	0.111725	-3.89225
4	2.72948	2.17736
4	3.68804	1.07546
4	-1.53632	4.89733
4	-0.058343	-2.08991
4	-1.45706	0.160673
4	-1.50621	-2.99308
4	-0.0425853	-1.87135
4	-1.8614	3.56708
4	2.63574	2.82448
4	-3.2627	-3.31979
4	0.615186	0.799779
4	1.51926	0.881006
4	-1.9011	0.66383
4	-2.14836	-1.27959
4	1.72788	2.00609
4	-3.23901	-0.412135
4	1.98257	-1.25456
4	-1.42379	-3.75283
4	-3.181	1.07131
4	-0.445233	-1.01515
4	2.26371	3.58627
4	-1.12621	1.42625
4	-1.33355	-0.631223
4	-3.98539	1.68956
4	1.33856	-2.17618
4	-0.862987	-0.891758
4	-0.560333	0.858254
4	0.567737	-1.98233
4	-1.00678	-1.45358
4	1.91166	3.75135
4	-0.590191	-1.79326
4	-1.11873	-3.34846

Cluster #	x-coordinates	y-coordinates
4	0.76448	-2.91048
4	0.757549	-1.282
4	-3.67275	-2.07471
4	1.09397	-0.551325
4	0.294615	0.230682
4	0.0137885	0.163522
4	-4.87163	1.7738
4	-1.09958	1.74366
4	-0.561848	1.55678
4	0.627504	0.256616
4	1.30804	-0.254863
4	-0.412448	-1.70482
4	0.421025	-1.23371
4	0.0986743	2.80905
4	1.79883	-0.297205
4	0.584487	-4.505
4	-2.73879	0.210771
4	-1.65645	-1.56188
4	-1.18258	-0.181346
4	-0.278845	3.66315
4	0.423797	3.77624
4	-1.78278	-1.57167
4	3.06757	-0.0300872
4	1.70931	-0.974022
4	-1.39449	-2.33311
4	2.53725	-1.55115
4	-0.533777	0.430775
4	1.0652	-0.138254
4	2.67074	0.240761
4	0.422706	-0.963662
4	2.77334	2.22719
4	-1.14679	-0.697444
4	0.151017	1.4409
4	0.965632	2.9415
4	0.211303	-2.66225
4	2.64226	0.528999
4	1.69253	-3.20841
4	1.98478	-1.98478
4	-1.23021	2.52925
4	-1.86775	2.45794
4	3.34069	-2.60261
4	-3.52188	1.05784
4	2.08441	1.41718
4	1.0609	2.88025
4	0.044982	1.47624

Cluster #	x-coordinates	y-coordinates
4	-1.60911	4.39831
4	1.05895	2.20231
4	0.284952	-0.570021
4	2.44855	-3.14846
4	4.33944	-3.86361
4	-1.94168	-0.957187
4	-2.82574	-2.3749
4	2.17931	0.713023
4	0.757968	-0.58687
4	2.72925	-1.10126
4	2.4235	2.4953
4	-3.66318	3.46469
4	0.0982961	-0.389228
4	0.70711	1.79833
4	0.980895	-0.187023
4	-2.26215	-0.153859
4	-3.67191	0.489812
4	1.95767	-1.69749
4	-4.54372	1.41967
4	0.668548	-1.09363
4	-0.629239	4.57638
4	0.305172	-2.05491
4	-1.91964	4.15421
4	0.580467	1.60392
4	-2.45232	-0.516202
4	-0.3681	-1.60406
4	-0.611015	-3.38687
4	-2.97029	-1.12713
4	1.80111	-2.64849
4	-0.508383	-0.411492
4	2.22844	-0.557015
4	-0.0530928	3.36721
4	0.540118	1.46692
4	-5.10117	0.537864
4	2.31196	-2.35928
4	3.60324	-2.25943
4	1.29352	-1.69458
4	0.0977617	-0.00222146
4	-1.35361	-0.794198
4	3.29796	0.354752
4	-1.16356	-0.870329
4	-0.896847	2.50616
4	2.70363	1.31153
4	0.498508	-0.660572
4	-3.35247	0.736836

Cluster #	x-coordinates	y-coordinates
4	-2.68822	-1.35455
4	0.331542	0.0528963
4	1.19688	-1.56676
4	-2.2899	0.839717
4	0.541564	-0.136578
4	-1.79442	-0.515427
4	2.22788	-1.5433
4	0.921941	1.457
4	0.344276	-1.6337
4	1.14147	-2.01319
4	1.62292	2.12185
4	2.45822	1.33695
4	1.16651	-0.845853
4	0.904897	4.59742
4	-1.78885	-0.760699
4	-0.926901	2.51597
4	-0.266385	1.1934
4	1.6145	0.0142848
4	0.143813	-0.828815
4	3.0096	1.44264
4	1.47313	-1.41122
4	0.0697859	-1.0951
4	1.96057	-0.592983
4	3.43157	-0.687957
4	0.274399	-3.78118
4	0.435796	1.7885
4	-0.142913	1.9601
4	-2.30269	0.835235
4	-0.674174	-2.05278
4	1.64786	-4.94918
4	-1.68108	5.96364
4	-1.80611	-1.09375
4	2.90639	2.41441
4	-1.8718	0.879153
4	-0.247329	-0.458584
4	-1.3829	-1.38166
4	-1.97672	-1.69516
4	1.58543	0.66701
4	-1.01164	2.67381
4	-3.73654	3.21831
4	-0.69902	1.59945
4	1.86134	0.271685
4	-1.16785	2.65515
4	-1.91594	-0.659318
4	-0.609889	0.546299

Cluster #	x-coordinates	y-coordinates
4	-1.91219	0.560012
4	0.948187	0.406594
4	1.69217	3.6969
4	0.315268	2.3404
4	-2.58585	1.70093
4	0.624812	-0.706615
4	2.26779	0.246726
4	-2.27027	0.306941
4	2.25385	-0.994743
4	1.99764	-4.81487
4	1.37416	0.540669
4	-1.83333	-0.179394
4	0.149926	0.937712
4	-1.68494	-0.190611
4	-5.70059	-1.22378
4	1.76609	0.397725

Appendix L

Square4
Number of dimensions: 2
Number of clusters: 4
Number of points: 1000

Cluster #	x-coordinates	y-coordinates
1	5.91815	9.53513
1	0.489376	4.88928
1	4.34518	4.53106
1	7.02122	7.55081
1	7.42314	3.95508
1	6.11805	8.21739
1	11.0948	8.26587
1	6.69226	2.1512
1	4.90355	11.4222
1	7.68905	7.01213
1	4.10514	8.93874
1	6.11192	7.71412
1	11.6073	6.48841
1	9.64674	9.09447
1	10.1599	8.72831
1	8.90331	4.85156
1	5.47752	7.31016
1	6.90628	5.67657
1	9.75616	9.00189
1	7.63738	6.43339
1	8.42163	2.68793
1	5.36993	8.13048
1	6.60763	5.32432
1	7.04769	6.66858
1	8.56007	6.39436
1	5.47449	4.94477
1	11.8497	3.4253
1	7.50045	9.1533
1	5.72176	9.63274
1	4.90371	4.33537
1	3.62644	11.0065
1	8.27319	9.53408
1	7.02108	9.68021
1	6.39233	5.23874
1	5.36436	10.9828
1	5.83456	6.41525
1	11.1479	4.91258
1	8.853	8.41605
1	8.66149	4.47548

Cluster #	x-coordinates	y-coordinates
1	8.27385	8.3008
1	6.56856	2.13795
1	9.77993	6.90034
1	4.96913	2.70368
1	8.35928	7.22438
1	6.99146	5.70283
1	6.84831	3.19991
1	7.60372	7.79523
1	8.51754	7.21937
1	5.16194	7.68667
1	8.10396	6.17443
1	8.60915	6.04262
1	9.70981	6.7085
1	3.95594	7.17825
1	9.46291	6.37873
1	7.37492	4.85683
1	8.19263	4.32245
1	6.63306	7.2561
1	7.12003	6.79305
1	5.39196	7.13948
1	5.62329	4.53451
1	5.79916	8.22001
1	6.85258	3.67574
1	2.25863	7.10899
1	11.9434	7.24931
1	3.02526	10.0077
1	5.17088	7.50061
1	7.4635	9.90146
1	5.63447	6.3989
1	5.75099	7.03305
1	5.24696	7.7584
1	4.97803	7.28354
1	5.41997	5.14809
1	6.09495	8.07844
1	5.35601	5.73416
1	7.70847	3.84957
1	6.13056	6.14775
1	4.02715	3.71693
1	7.98289	5.21377
1	5.65542	7.70285
1	8.68665	4.71472
1	4.9471	6.44515
1	9.47412	5.16487
1	7.45509	7.31084
1	6.91576	6.40742

Cluster #	x-coordinates	y-coordinates
1	6.20429	8.01444
1	7.70346	9.23762
1	8.44555	8.3788
1	3.69763	9.8168
1	7.85905	7.10389
1	9.86451	9.75759
1	9.39492	6.1466
1	3.8347	7.4773
1	8.53268	8.39579
1	6.31542	5.70253
1	6.85142	7.22193
1	6.18295	9.30312
1	7.84875	9.67916
1	6.09259	6.41772
1	5.80604	6.50106
1	9.63884	5.08005
1	7.05501	5.2253
1	8.56786	3.69784
1	6.83518	7.91392
1	6.11619	6.20132
1	4.51346	6.11083
1	7.62809	6.86708
1	6.36456	7.15668
1	6.27335	5.05917
1	5.04303	4.07525
1	8.5817	9.70808
1	6.08985	5.83584
1	5.97027	11.6203
1	7.12101	5.97064
1	5.67389	5.36975
1	6.13762	9.27273
1	11.7664	9.27186
1	5.77969	5.91358
1	6.20956	5.85219
1	6.06619	0.715959
1	8.38364	7.31002
1	5.19956	6.09097
1	6.82061	4.61704
1	5.1193	5.24373
1	7.24192	10.2008
1	5.63752	8.11768
1	6.57138	7.14479
1	6.22796	2.62779
1	5.79117	6.7827
1	8.7464	4.80618

Cluster #	x-coordinates	y-coordinates
1	4.4391	7.34459
1	7.71867	8.31104
1	9.41703	3.6764
1	9.38559	7.00976
1	6.70403	5.03889
1	7.77578	8.53613
1	7.84402	6.43441
1	4.41805	8.54089
1	6.16119	6.4582
1	8.40656	6.96582
1	6.02993	8.35238
1	6.43413	6.10602
1	9.48707	8.15835
1	8.11097	4.98782
1	6.74188	5.01177
1	6.52849	7.25595
1	6.648	6.07675
1	6.78541	8.40514
1	9.0121	8.52481
1	7.12516	5.56722
1	6.72628	9.36193
1	10.0291	9.09945
1	8.86359	6.95054
1	6.83835	5.88627
1	6.75587	7.06655
1	6.72892	6.13325
1	8.35552	6.33055
1	8.17556	7.69387
1	7.70491	6.79191
1	5.46582	6.9935
1	10.5578	3.35863
1	4.03373	6.15298
1	9.4866	1.8382
1	6.54625	6.6823
1	6.51781	8.19866
1	8.4737	6.08128
1	6.24349	6.39937
1	10.6148	5.15726
1	8.73645	6.33897
1	10.4371	4.64695
1	11.5642	7.57843
1	3.49915	6.08262
1	6.7712	6.94547
1	6.71917	10.7924
1	7.8903	5.42369

Cluster #	x-coordinates	y-coordinates
1	7.90887	2.67056
1	5.46587	7.71441
1	6.02141	6.65003
1	6.46888	7.75092
1	7.55745	7.58577
1	5.72819	6.86348
1	8.36035	5.55127
1	7.93974	6.86319
1	8.11979	7.87302
1	6.50601	9.77427
1	8.3746	5.53857
1	7.34969	5.50141
1	7.76241	4.5377
1	6.81285	5.51707
1	7.67744	4.77271
1	5.34327	4.43118
1	7.32359	5.32498
1	8.76091	6.12847
1	7.58038	7.39399
1	8.20552	5.72699
1	7.03922	6.35025
1	7.36367	4.93242
1	13.1638	8.31465
1	4.11872	5.43478
1	8.59026	4.81498
1	6.83303	5.29138
1	7.30836	3.01256
1	7.31221	5.59247
1	5.12751	6.06003
1	8.6081	8.92087
1	7.88095	9.12046
1	4.17522	7.64828
1	4.05497	10.3973
1	3.57328	10.196
1	6.9138	3.23703
1	7.65441	7.34124
1	5.67597	8.32939
1	8.11006	7.97858
1	7.2582	9.4857
1	6.94838	3.27585
1	5.59715	4.68085
1	11.1023	5.724
1	7.61597	7.91994
1	7.52017	7.73981
1	8.96876	7.12366

Cluster #	x-coordinates	y-coordinates
1	11.7195	5.0571
1	6.37479	9.58285
1	6.7038	7.62314
1	10.494	5.80632
1	10.8091	5.40665
1	7.62207	7.91971
1	9.47258	10.1978
1	7.34029	5.11724
1	11.965	9.75066
1	4.21487	7.139
1	9.42067	6.99888
1	5.86942	7.90168
1	5.40625	6.83956
1	8.96298	6.64422
1	5.34552	5.61067
1	11.2724	6.52255
1	6.62062	5.4822
1	5.34616	8.99125
1	5.06614	4.73311
1	3.3571	8.79784
1	7.51735	7.02767
1	8.30513	8.74209
1	5.96913	8.07742
1	7.60244	2.22207
1	5.50693	5.77242
1	5.306	7.18888
1	4.15781	7.70042
1	11.3887	8.39585
1	3.58083	7.82524
1	10.3266	7.74475
1	7.27032	4.73111
2	4.39353	-1.20024
2	9.63449	0.571803
2	6.57799	0.220211
2	7.68135	0.473663
2	7.34333	-1.78692
2	7.87917	2.92078
2	5.3193	1.71161
2	8.7986	-0.0679336
2	5.33917	0.136586
2	9.2994	-2.27812
2	5.90088	1.76798
2	6.88099	0.287336
2	5.02071	-2.24806
2	9.47942	-3.178

Cluster #	x-coordinates	y-coordinates
2	4.95665	-2.34003
2	7.56013	-0.416724
2	5.75601	-0.794382
2	5.6571	2.27568
2	7.43269	0.194291
2	5.75846	1.46377
2	6.32657	1.76115
2	10.0461	0.580973
2	5.83838	1.27006
2	4.16201	4.80894
2	8.68687	-0.330219
2	9.78365	-0.146093
2	5.67677	-1.17071
2	8.21321	3.01128
2	4.21576	-1.15005
2	7.07013	1.15632
2	11.3741	0.150317
2	8.58087	-0.219946
2	4.13532	-1.31634
2	9.35418	-1.38723
2	8.29897	-0.656538
2	11.6513	1.88826
2	7.34101	1.16143
2	7.21993	-2.13515
2	8.0105	-3.52719
2	7.77472	2.78558
2	8.33508	0.114915
2	10.2135	0.419203
2	2.72227	-1.3987
2	8.06563	3.16289
2	8.41436	-1.74157
2	5.95045	-1.56304
2	8.41792	-3.26488
2	3.22233	-0.376157
2	6.32017	1.99473
2	10.1391	0.266007
2	6.62292	0.360554
2	10.1297	0.889454
2	4.93789	-1.15538
2	6.14324	-1.27705
2	8.04374	-1.8231
2	7.3992	1.27164
2	5.18983	0.591048
2	8.24588	-0.532007
2	7.16941	2.09375

Cluster #	x-coordinates	y-coordinates
2	7.38357	0.393407
2	9.25474	-2.69018
2	6.33365	-0.308201
2	5.45638	-1.68978
2	9.66566	-1.13097
2	7.13403	1.59558
2	5.0407	-0.0368184
2	7.23112	0.731135
2	6.88594	1.82921
2	6.97679	-3.66308
2	5.36817	1.16798
2	9.51729	0.968589
2	9.18629	0.479526
2	5.98315	-1.59762
2	3.34659	-1.38173
2	5.28307	-1.71295
2	5.63059	1.781
2	6.21861	0.888512
2	9.28644	4.04908
2	6.42839	-0.339265
2	7.91991	0.0261846
2	4.09524	1.91762
2	7.39462	-0.0411347
2	10.1267	0.355148
2	7.89019	-0.116967
2	8.45649	-0.741293
2	7.06005	1.09534
2	8.65723	-3.36526
2	5.9723	0.95563
2	11.4178	-0.091203
2	9.00999	-2.51729
2	7.17554	1.03686
2	6.50023	0.679527
2	8.12497	-1.74248
2	9.51149	-2.21816
2	5.12	-1.13448
2	10.047	2.09004
2	4.50425	-2.16844
2	7.33947	2.68792
2	3.56332	0.407467
2	5.30632	-2.6556
2	9.23509	-0.0668713
2	10.4665	-3.58891
2	5.30747	-2.2761
2	7.08574	-1.06796

Cluster #	x-coordinates	y-coordinates
2	9.18992	-0.696005
2	8.34313	2.08274
2	6.11268	-0.173273
2	9.58059	-1.63033
2	6.20937	0.114912
2	8.42098	-2.13471
2	4.9611	2.39867
2	8.61714	-3.59155
2	6.24679	-0.985826
2	4.64476	1.60056
2	9.44487	-0.07991
2	6.40292	2.15901
2	5.31391	-0.486409
2	6.80083	1.18197
2	7.30442	-4.66904
2	4.58814	-0.90003
2	3.69415	0.234064
2	6.87242	-4.3834
2	6.41894	-1.00996
2	7.92697	2.48792
2	8.38156	-1.68902
2	6.13797	1.00355
2	7.38177	-4.04461
2	5.94103	-1.25021
2	11.1488	1.53209
2	3.18575	-3.47591
2	5.61585	-2.86976
2	6.55687	0.517216
2	3.45877	0.58545
2	7.92077	2.96505
2	9.51534	-2.78924
2	9.30452	-1.74927
2	4.19858	0.949606
2	7.1605	1.93322
2	5.1074	-1.47641
2	5.01784	0.713566
2	4.86723	2.95246
2	8.24271	-0.951154
2	12.6031	-3.50025
2	10.3869	1.04581
2	5.14687	0.298387
2	6.92161	-0.583213
2	7.77022	-1.14033
2	6.27187	-1.13779
2	2.91189	1.26993

Cluster #	x-coordinates	y-coordinates
2	9.56173	1.4558
2	8.12808	-0.520544
2	7.20129	1.07724
2	7.25377	0.831763
2	9.7008	0.208776
2	8.78311	-1.65011
2	7.39212	2.27475
2	7.61346	1.70984
2	10.5126	0.857671
2	8.46972	1.95639
2	5.35489	0.369185
2	1.56414	-3.7009
2	5.78809	1.164
2	9.59484	1.3054
2	6.78471	-1.6115
2	5.27879	0.889668
2	7.30589	-3.06157
2	5.60757	-1.7211
2	8.54689	-2.64531
2	6.28346	1.25499
2	7.51046	1.07213
2	9.51663	-0.0751523
2	6.51851	-1.58686
2	4.19716	-0.0675098
2	9.26378	0.497081
2	9.59384	0.801701
2	4.92324	-1.24049
2	5.82741	-2.15284
2	7.89295	1.30853
2	6.43123	-2.54891
2	7.37916	-3.67488
2	7.6036	-1.24694
2	7.93307	-2.57298
2	6.63375	-1.26459
2	6.68744	1.87543
2	3.94396	0.0203852
2	6.49656	1.51506
2	7.0147	2.10468
2	7.65224	4.08246
2	6.54798	0.306118
2	2.95723	-0.147521
2	9.7284	-0.605429
2	5.76064	0.537446
2	6.94939	-0.81542
2	4.4333	-0.489986

Cluster #	x-coordinates	y-coordinates
2	9.25354	1.23558
2	8.64278	-3.4589
2	6.64917	0.437016
2	7.36209	-2.27483
2	8.33604	-0.220394
2	6.07158	-3.43565
2	7.76208	-2.04028
2	2.63098	1.22741
2	4.01364	-1.94146
2	6.28804	0.983855
2	4.94641	-0.0144272
2	10.4815	-0.939402
2	6.42766	-0.130748
2	4.03496	-3.86797
2	5.32655	-1.51437
2	8.90958	2.16322
2	5.59108	-2.43159
2	8.5912	-1.32814
2	5.80834	-0.262306
2	4.79392	-3.62293
2	4.59938	0.00143903
2	5.13082	0.997248
2	8.34293	0.815243
2	7.09347	0.437471
2	8.38519	3.35734
2	6.20443	-2.54501
2	10.2898	-0.0801789
2	5.75237	3.69017
2	6.21655	1.37331
2	5.4007	-2.50903
2	6.87219	1.77399
2	7.84271	-1.53826
2	7.40732	1.17211
2	5.05058	2.66791
2	5.57618	-1.30347
2	6.23712	1.09409
2	5.90048	0.772503
2	7.63646	1.80943
2	13.9179	-0.197189
2	5.2043	1.04551
2	8.52545	0.254028
2	6.77234	1.80348
2	9.13389	2.02881
2	6.29586	0.735134
2	10.5732	2.50704

Cluster #	x-coordinates	y-coordinates
2	9.9023	1.61045
2	7.8121	-1.31278
2	5.58587	0.274094
2	6.31306	0.323601
2	6.01491	-3.69019
2	9.24115	1.2531
2	8.21484	1.66115
2	7.06882	-0.179804
2	10.8343	-1.32571
2	5.65204	-2.63818
2	2.73562	-3.12102
3	-0.88463	7.41924
3	-1.44173	7.85145
3	2.69977	9.43083
3	-3.83258	9.85435
3	-2.57683	5.04408
3	-2.72109	7.75786
3	1.95623	8.05569
3	-0.855727	3.50002
3	0.199639	8.99733
3	-1.23062	8.91109
3	1.67575	8.27525
3	-2.54947	6.76859
3	-2.05736	8.43954
3	0.673589	6.96298
3	-0.122439	7.67245
3	4.17262	6.01813
3	-0.404633	7.22314
3	-5.25711	7.62481
3	-1.90689	9.88826
3	-1.257	4.75048
3	-2.33276	7.9402
3	1.4941	4.30877
3	0.903441	7.62717
3	1.67957	6.51633
3	0.632445	7.92749
3	-1.68885	5.04659
3	2.22352	6.88267
3	2.45968	6.30073
3	0.958437	1.6875
3	-1.78233	11.8439
3	1.98979	8.95386
3	-0.117685	6.99229
3	0.355566	5.8611
3	2.19982	5.97496

Cluster #	x-coordinates	y-coordinates
3	2.04789	9.66463
3	-1.42221	7.64904
3	1.02988	8.01377
3	-2.30951	6.22235
3	-0.610932	7.48765
3	-2.91502	4.09239
3	2.74999	8.1155
3	0.22299	8.25748
3	-0.255861	4.65353
3	-2.31106	6.67945
3	-1.10657	8.59571
3	-0.423471	4.57648
3	-2.47327	7.16338
3	4.11464	10.2
3	-1.32286	5.72995
3	-2.25064	3.56973
3	0.495123	7.38313
3	-1.04101	9.25265
3	2.45224	6.55959
3	-2.94018	10.2952
3	0.544079	9.69193
3	1.17271	4.97031
3	1.81454	5.54422
3	1.53407	9.85016
3	3.88827	6.01216
3	-1.89175	2.92473
3	2.15882	6.48874
3	-2.62674	8.42262
3	-0.620996	5.08342
3	-3.92603	5.12895
3	0.639454	8.07908
3	-0.539377	6.05422
3	4.2133	4.95412
3	1.78439	7.25014
3	0.474377	6.62908
3	2.35938	4.66535
3	-1.76006	8.86782
3	0.308986	6.79992
3	3.02362	7.27824
3	1.16744	6.27194
3	1.4242	5.42305
3	-2.76744	6.57456
3	0.84374	11.2652
3	-0.940029	9.86396
3	-1.13307	8.35321

Cluster #	x-coordinates	y-coordinates
3	-2.04025	5.53027
3	0.271364	6.11419
3	0.566152	5.28231
3	-0.866938	10.1166
3	-1.34809	7.38279
3	4.12207	6.71616
3	0.688471	9.9701
3	3.87007	8.78409
3	1.5836	5.67737
3	-0.0661454	6.68891
3	1.17755	6.55333
3	1.15269	7.60508
3	3.24	3.46418
3	1.20693	10.9757
3	-0.132663	8.82602
3	-3.35259	4.25403
3	-2.96999	6.59295
3	-1.59157	5.81041
3	-4.02301	5.84235
3	-3.44458	6.23632
3	-0.363455	7.95563
3	0.741928	10.669
3	3.22018	7.46089
3	-1.57566	6.92159
3	0.650953	8.20188
3	-1.37641	7.13552
3	-2.39163	8.5452
3	0.854476	5.58869
3	0.212461	7.33354
3	-2.55859	9.47776
3	3.01094	6.84282
3	-2.34477	8.45843
3	-4.03579	8.15881
3	0.883366	5.81069
3	0.934537	7.33256
3	-0.0794829	9.07753
3	-1.19417	9.33467
3	-3.81485	3.5995
3	-1.03597	3.3391
3	0.276408	9.45537
3	4.83214	8.1435
3	2.02712	7.782
3	-2.7361	7.57567
3	-2.37728	10.7413
3	2.00737	6.47943

Cluster #	x-coordinates	y-coordinates
3	0.0752467	6.0071
3	-1.65686	6.15563
3	4.0427	4.93978
3	0.0293176	10.2881
3	-4.04077	5.48437
3	0.242432	7.61628
3	1.39261	7.83824
3	-1.70549	4.57676
3	-1.1928	6.62438
3	-0.142983	7.80966
3	-0.97931	6.26869
3	-1.3092	7.49385
3	-2.68575	9.81978
3	-0.175218	5.97072
3	-0.218836	8.36867
3	-1.3336	5.83158
3	0.336907	9.58648
3	-2.92943	5.17273
3	2.69172	6.63146
3	1.9186	7.86737
3	-4.36132	7.16643
3	0.0403268	6.96458
3	-0.835189	8.83983
3	-0.318523	7.00269
3	-3.90803	6.81176
3	2.67043	5.17373
3	2.00662	8.0256
3	3.01708	5.92799
3	0.187485	8.39675
3	1.63449	5.8768
3	-0.313301	3.7811
3	2.15827	10.8566
3	-0.62625	6.41988
3	-0.979033	10.2468
3	-0.612606	9.14472
3	-2.13983	9.40597
3	-0.736603	8.20436
3	-2.93442	4.81147
3	0.63369	7.90195
3	-1.39827	8.93039
3	-1.13712	9.31812
3	-2.10686	4.8361
3	-2.39899	8.67149
3	0.618436	8.38539
3	-0.977231	7.99181

Cluster #	x-coordinates	y-coordinates
3	0.235328	8.52853
3	-6.19536	7.4358
3	-1.32778	6.95868
3	-0.615913	5.75322
3	-0.27714	10.8755
3	-0.608864	8.32102
3	-0.25923	6.65996
3	1.63142	7.60007
3	-2.7705	5.79515
3	-1.03958	9.94941
3	-2.65868	5.74112
3	2.66476	9.57749
3	-0.909772	2.87881
3	1.87737	2.74917
3	2.1972	5.40144
3	-0.199304	4.2785
3	-0.98993	3.52789
3	-2.82236	5.46249
3	-2.62299	7.09663
3	-0.310849	10.193
3	-2.51044	5.23618
3	1.49266	9.86883
3	-3.12353	7.73842
3	-0.0771915	6.63637
3	-3.25052	7.88543
3	0.179169	4.26765
3	0.390146	9.02596
3	4.246	6.58088
3	0.834383	9.68958
3	1.41056	6.48653
3	-1.13746	8.27542
3	1.81217	7.22559
3	-0.350204	4.98629
3	-1.84307	3.93739
3	2.84054	3.85295
3	2.35738	3.67572
3	1.85786	5.42312
3	3.93114	6.43097
3	-0.722016	4.94521
3	-1.59771	9.18507
3	-5.61828	6.28412
3	1.87305	7.71558
3	-0.556128	6.90314
3	1.77137	8.15951
3	-0.253143	6.21054

Cluster #	x-coordinates	y-coordinates
3	-0.832279	9.01545
3	-0.829817	6.58792
3	1.24375	10.3218
3	0.646943	5.96354
3	0.440253	7.39638
3	-0.531082	5.36602
3	0.648161	7.228
3	0.532477	8.02471
3	-1.58148	8.51454
3	-1.67687	9.00849
3	-1.84853	7.9405
3	-0.206525	5.30411
3	-1.10879	10.2438
3	-1.44129	6.99166
3	-1.18059	10.2918
3	6.6164	6.76898
3	-1.28755	8.50175
3	3.76921	1.98802
3	-0.126651	7.10155
3	0.389841	4.71839
3	2.14776	6.19742
3	-1.23698	7.90469
3	0.86892	8.57322
3	-0.712217	10.2091
3	-2.47763	5.703
3	2.29638	7.07126
3	-6.72058	6.21722
3	-3.49537	7.4904
3	-1.45136	7.41974
3	-2.14076	11.3098
3	0.318871	6.38503
3	-0.283853	5.57159
3	-2.10516	10.0043
3	0.212308	5.83265
3	-1.96914	4.37215
3	0.0760469	6.92766
4	-1.75104	0.455123
4	3.57628	-1.7249
4	0.627729	-2.08367
4	-0.475252	3.09706
4	1.9137	2.85007
4	-1.05133	1.0378
4	1.0814	-1.76578
4	-0.998765	0.206281
4	1.70214	-2.12256

Cluster #	x-coordinates	y-coordinates
4	0.292305	0.75739
4	-1.66685	1.52251
4	0.934952	1.23891
4	0.124275	0.912049
4	-1.9344	-0.655515
4	0.605343	0.266514
4	-0.986763	-0.923201
4	1.58748	1.82007
4	-4.14561	-2.89872
4	-1.85472	-1.96885
4	-0.220597	2.05051
4	5.10567	-2.03435
4	2.72387	2.64722
4	-0.597341	-0.69472
4	-1.44779	-1.3173
4	-0.335976	-1.22219
4	-1.11141	-0.597659
4	-0.179199	2.74925
4	0.673791	3.81766
4	1.99528	-0.0109918
4	-1.72652	-3.40779
4	-0.375094	0.367475
4	-2.39787	1.56678
4	-2.2294	-0.723344
4	-0.107272	-2.6389
4	-3.02951	0.216485
4	0.32665	-1.15885
4	0.182556	-1.35709
4	1.38232	1.42557
4	-1.64274	0.777005
4	1.6378	0.62668
4	1.12292	2.00346
4	-0.909836	-1.87807
4	1.09013	-0.428428
4	-2.2321	0.207745
4	-2.59773	1.8254
4	-0.366119	0.885201
4	-2.02246	-1.97031
4	-0.00232836	1.56677
4	-2.56691	-2.60793
4	0.910017	1.05143
4	-0.442537	1.13782
4	-0.757977	3.67122
4	1.06225	-1.4107
4	-0.295762	-0.64308

Cluster #	x-coordinates	y-coordinates
4	-0.897845	-5.477
4	-1.52508	4.01341
4	3.19792	2.92941
4	-0.652975	-2.08938
4	0.268023	-1.56893
4	1.30022	-1.47035
4	-4.12676	3.11137
4	-1.84606	-1.52014
4	1.17298	2.41376
4	-7.13692	1.79422
4	-0.0265405	-0.344262
4	-2.47778	-1.94326
4	-0.743324	0.851405
4	-3.16358	2.96239
4	2.94894	0.136855
4	-3.92604	-2.58464
4	-0.755676	1.58076
4	0.153356	0.706809
4	-0.0295902	2.35907
4	-1.12399	0.901225
4	-1.12802	-4.22746
4	-0.498187	-0.105259
4	1.12323	1.27777
4	1.55675	-1.68744
4	0.967236	-2.71144
4	1.46026	-3.005
4	-0.217442	0.565508
4	0.763649	3.78328
4	-1.8655	-1.56275
4	-1.61915	-1.91368
4	0.0499096	2.1724
4	0.338132	-0.267394
4	0.190778	-2.24623
4	-0.369959	-1.42589
4	1.33051	-0.441973
4	0.170559	0.937638
4	3.08525	-1.7951
4	0.94448	1.79365
4	-1.66525	-1.5557
4	-3.30427	-1.84539
4	-1.10588	-1.02464
4	-0.914241	-3.5633
4	0.471111	2.21797
4	5.34985	0.923263
4	-0.43965	-2.05021

Cluster #	x-coordinates	y-coordinates
4	1.02371	-2.07259
4	-0.744132	0.366448
4	1.62478	2.43753
4	-0.226002	0.601471
4	2.47387	-0.436165
4	-2.88536	-0.135811
4	1.30792	4.44434
4	2.97758	-0.549452
4	-1.93147	0.224799
4	1.15278	0.896164
4	0.807567	-0.384002
4	0.0945464	-0.414685
4	-4.4582	0.11135
4	-2.7999	1.44498
4	0.952653	0.969657
4	-0.586934	1.84292
4	0.719338	-1.95423
4	2.82012	-1.3566
4	0.537087	-0.202835
4	0.228019	1.92383
4	1.55313	-0.789077
4	-0.0540795	-1.59414
4	-1.02742	-2.86798
4	-0.375841	2.26702
4	-1.94914	-1.67185
4	-1.60511	-1.20026
4	-0.103857	0.46644
4	-2.86026	-3.3588
4	-0.0638231	-1.23298
4	-0.187031	0.28762
4	3.01343	2.03812
4	-5.15975	-1.07693
4	1.42717	-0.0989295
4	-0.130943	2.50349
4	-0.699615	-3.44139
4	-1.40478	-1.7485
4	3.3929	-1.94705
4	-1.28473	0.86107
4	-2.77373	-1.24569
4	-2.21899	-0.165564
4	-1.10743	-3.54796
4	0.756668	1.17721
4	1.05318	-1.93246
4	1.26879	0.462181
4	-0.323631	1.79838

Cluster #	x-coordinates	y-coordinates
4	1.06724	-2.50506
4	-2.59282	-4.4324
4	1.65249	-2.76449
4	-2.61456	1.42007
4	-1.08727	-0.189084
4	-1.18175	1.60867
4	-1.9461	1.5341
4	-3.28812	2.0767
4	-1.42549	0.884786
4	1.30977	-0.857004
4	-0.676866	-0.764651
4	-1.4494	-1.25813
4	1.39657	0.638231
4	4.58918	-0.302894
4	-3.20005	1.25796
4	-3.33891	-3.83216
4	1.53153	-2.07093
4	0.0539145	1.53998
4	0.744541	-0.332976
4	2.84442	-1.13667
4	1.03143	0.118409
4	-1.72116	-3.03467
4	1.6533	1.27729
4	1.06903	-2.60267
4	0.0971143	1.88012
4	-2.53185	-1.71276
4	-1.69404	-4.07265
4	4.32204	0.461679
4	1.27552	-0.446429
4	2.48253	1.2159
4	-2.43106	-0.392404
4	-2.32953	-2.50177
4	2.52745	-2.20178
4	-2.31023	-1.10795
4	-2.37391	-3.73919
4	-3.20067	1.8459
4	2.2016	-0.10471
4	0.0685817	1.02864
4	-0.870086	-0.291898
4	-1.45371	0.141891
4	-0.0877024	-2.48967
4	0.606611	-0.081751
4	0.171555	-4.61999
4	2.70636	2.12816
4	1.22302	-0.505777

Cluster #	x-coordinates	y-coordinates
4	2.71848	-0.201261
4	0.106061	-1.69553
4	-3.18841	0.637818
4	0.548571	0.843226
4	2.24406	-0.277501
4	1.08862	-1.58337
4	-2.64447	2.68305
4	-0.655907	1.78148
4	2.42658	3.47957
4	2.93732	3.2831
4	-2.25055	1.83578
4	1.68417	1.70844
4	-1.32971	2.02516
4	-0.185534	-1.89569
4	1.84533	1.69829
4	-3.45328	-0.767753
4	3.99306	2.84988
4	3.3882	0.311992
4	-0.125603	-2.6124
4	-1.26544	0.241407
4	2.85579	1.75834
4	-0.505328	0.22071
4	0.575483	-0.00863481
4	1.60888	1.74365
4	2.02842	-1.69187
4	-1.40362	-0.525708
4	-0.447888	2.62316
4	-5.90833	1.46047
4	3.72563	0.0657731
4	0.500163	3.90239
4	0.624924	1.68849
4	0.233193	2.73283
4	0.337826	-0.650743
4	2.36223	-1.98953
4	-1.91809	2.7655
4	0.933046	-2.64742
4	-1.38959	-1.59676
4	-2.04759	-0.573477
4	-3.81247	-1.88292
4	-1.06134	-2.398
4	1.88131	1.1363
4	1.15563	-0.480651
4	1.17409	6.20152
4	2.41377	-0.0791764
4	-1.25479	0.48621

Cluster #	x-coordinates	y-coordinates
4	1.36629	2.31368
4	0.110982	0.991162
4	-1.22486	1.49366
4	0.861805	-2.13737
4	0.273987	-0.219811
4	-0.443873	-2.35277
4	-0.217212	-0.434731
4	-0.387709	-1.47303
4	2.84242	1.86779
4	0.117769	-1.7089
4	-2.79382	-0.0443493
4	-3.22921	-0.936391
4	1.47975	-1.5631
4	2.66172	-1.57756
4	-4.24082	-0.982036
4	2.56557	-0.0840354

Appendix M

Twenty
Number of dimensions: 2
Number of clusters: 20
Number of points: 1000

Cluster #	x-coordinates	y-coordinates
1	0.0673908	0.648651
1	0.184058	0.285643
1	0.216653	-0.626459
1	-0.230196	-0.277555
1	-0.343994	0.952639
1	0.516222	0.527655
1	-0.340626	0.372044
1	-0.891206	-0.0338618
1	0.0231218	0.0798055
1	-0.0480045	-0.112795
1	-0.549665	-0.185131
1	0.610166	0.0548585
1	0.240708	-0.00879581
1	-0.0952792	0.612275
1	0.629652	-0.400976
1	0.535674	0.531926
1	-0.0295303	-0.307669
1	-0.725821	0.361157
1	-0.284648	0.409727
1	0.357847	-0.115821
1	0.153626	-0.909621
1	0.286176	0.13731
1	-0.897728	0.152465
1	-0.895161	0.137934
1	-0.591967	0.218019
1	-0.445756	0.398878
1	0.242248	0.337062
1	-0.420387	-0.315739
1	0.0443506	-0.628764
1	-0.124006	0.499045
1	0.112187	-0.358485
1	-0.0236469	0.134742
1	0.174311	0.170205
1	0.025102	-0.162716
1	-0.293906	-0.0332021
1	-0.50098	0.0855036
1	-0.0341769	0.374567
1	-0.812607	-0.124809
1	-0.39211	-0.06304

Cluster #	x-coordinates	y-coordinates
1	-0.247678	-0.550145
1	1.06066	0.37034
1	0.241095	0.354813
1	0.675214	0.165773
1	0.346748	0.425497
1	-1.03046	0.814915
1	0.607113	-0.270217
1	-0.671951	0.418234
1	0.371304	-0.360211
1	-0.866275	0.336349
1	-0.0924798	0.385889
2	5.23032	0.0733765
2	3.98709	0.290578
2	4.23755	-0.279124
2	4.05608	0.612748
2	5.35788	0.27984
2	4.08401	-0.435649
2	3.79528	0.139738
2	2.89531	0.617328
2	4.35429	0.316387
2	4.17686	0.124034
2	4.38816	0.140594
2	4.70558	-0.328459
2	4.51607	-0.0782005
2	4.59905	0.343144
2	3.251	0.159402
2	3.01802	-0.353557
2	3.92826	-0.211247
2	3.80549	0.0196756
2	4.08564	0.586435
2	3.71035	-0.00805321
2	4.77432	0.16693
2	4.38432	0.169625
2	4.87446	0.0507428
2	4.67376	-0.144914
2	3.59141	0.892578
2	3.82578	0.932617
2	4.1505	0.301503
2	4.43412	-0.186553
2	3.79532	-0.112176
2	3.23263	0.321166
2	3.97525	0.472507
2	3.98389	0.659611
2	3.49448	-0.434678
2	4.52451	-0.180956

Cluster #	x-coordinates	y-coordinates
2	4.3327	-0.505153
2	5.07539	0.0267725
2	4.90042	-0.605792
2	3.11646	0.152985
2	4.96567	-0.112223
2	4.56492	0.837235
2	3.33208	0.459567
2	3.75286	-0.0387904
2	3.8966	-0.109682
2	4.2599	0.23564
2	4.3199	-0.466008
2	3.39795	-0.275024
2	3.15548	0.122624
2	3.78584	0.0197974
2	3.99271	-0.601369
2	4.73071	-0.191239
3	8.30909	-1.09333
3	8.15717	-0.244955
3	7.6083	0.161101
3	8.68201	0.698076
3	8.41225	-0.358268
3	8.12161	0.16769
3	8.35901	0.13728
3	8.06171	0.514286
3	8.65864	0.702299
3	7.57486	-0.00914143
3	7.6876	-0.32635
3	8.0751	0.0687498
3	8.29138	0.168367
3	7.59447	-0.614139
3	7.81159	0.514963
3	7.63687	-0.808251
3	7.53363	-1.06035
3	7.48937	-0.152447
3	8.07675	0.0673557
3	8.04362	-0.53697
3	7.95937	1.02369
3	7.76303	-0.111388
3	6.73637	0.0110952
3	7.31677	0.557347
3	7.29637	-0.344011
3	7.41118	0.175356
3	8.04672	0.656547
3	7.58396	1.37038
3	7.24168	-0.135876

Cluster #	x-coordinates	y-coordinates
3	8.08668	0.180556
3	8.36457	0.099364
3	7.66844	-0.424357
3	8.02776	-0.767283
3	7.24715	0.851412
3	8.35838	0.432283
3	7.75519	0.153029
3	8.099	-0.0839765
3	8.73561	0.629472
3	8.42773	-1.69667
3	7.45885	0.292715
3	7.56676	0.897958
3	8.62853	-0.119195
3	8.20596	0.777928
3	7.63379	-0.338959
3	7.17197	0.451926
3	7.8471	0.346593
3	8.87306	0.867481
3	8.84844	-0.231711
3	8.63226	-0.834833
3	9.05391	0.561159
4	11.1975	-0.164718
4	12.4641	0.928536
4	12.863	-0.145341
4	11.3365	0.432666
4	11.5552	0.397367
4	11.8646	0.415389
4	12.5514	-0.524287
4	11.5798	-0.409786
4	12.0817	-0.546197
4	12.3245	-0.173053
4	12.1314	-0.165384
4	12.2732	0.112706
4	11.3454	-0.396408
4	11.7244	-0.642267
4	11.7995	0.318161
4	12.3045	0.0922153
4	11.6	0.35805
4	12.2717	-0.203684
4	11.9235	0.861123
4	11.9339	-0.0940799
4	12.2208	0.415675
4	11.6661	0.626839
4	11.7969	0.341755
4	11.2261	-0.434285

Cluster #	x-coordinates	y-coordinates
4	11.4189	-0.146451
4	11.4234	0.243083
4	12.1577	0.685386
4	12.9794	0.356174
4	11.5031	-0.589598
4	12.1274	1.30056
4	11.9922	-0.170522
4	12.5542	0.0656623
4	11.9605	-0.170221
4	12.4313	0.360493
4	12.1001	-0.726223
4	12.2734	-0.729638
4	11.9084	-0.286947
4	12.1675	-0.161831
4	11.3516	-0.455417
4	12.1394	-0.257454
4	11.6486	0.596941
4	11.8131	0.0721978
4	12.4061	-0.185393
4	12.4003	-0.620343
4	12.211	0.267456
4	11.9656	0.788727
4	12.0013	0.472102
4	12.3559	0.450007
4	12.3334	0.0824647
4	12.6421	0.644992
5	15.481	-0.0082689
5	15.6691	0.23389
5	16.352	0.474408
5	15.1474	0.0220489
5	16.4678	-1.18061
5	16.0799	1.02031
5	16.7582	-0.447348
5	16.4659	-0.0926586
5	16.7062	-0.359968
5	16.5817	-0.2366
5	16.523	-0.193144
5	15.6235	-0.66605
5	15.5422	-0.34599
5	15.9675	-1.23148
5	15.9527	-0.913096
5	16.2111	0.114058
5	16.7252	0.5625
5	15.9257	0.516454
5	15.7259	0.225129

Cluster #	x-coordinates	y-coordinates
5	16.4893	-0.459633
5	16.5956	-0.439762
5	16.524	0.768607
5	15.0611	0.428078
5	16.0519	-0.0635405
5	15.7468	1.38617
5	15.6896	-0.0903988
5	16.8765	0.550375
5	16.7192	-0.387069
5	15.6919	0.599317
5	16.3653	0.792675
5	16.8072	0.392959
5	16.1332	0.477955
5	16.1053	0.485305
5	16.5409	0.142072
5	15.7261	-0.381532
5	15.6043	-0.45437
5	16.6924	0.0682712
5	15.3472	0.27921
5	15.9568	0.506007
5	14.6238	-0.0709561
5	15.3243	-0.181102
5	15.6126	-0.81875
5	16.0977	-0.569975
5	16.671	-0.0552296
5	16.4629	-0.274468
5	15.591	0.342416
5	16.2158	-0.149446
5	15.2817	-0.880813
5	15.6068	0.283972
5	15.8052	-0.360174
6	0.282894	3.91705
6	-0.41441	3.78084
6	-0.213483	3.63291
6	-0.222538	3.64856
6	-0.36068	3.55096
6	-1.19683	4.59413
6	-0.300099	4.06088
6	-0.152305	3.44536
6	0.669164	3.8584
6	-0.357115	3.83705
6	-0.163598	4.65006
6	-0.409927	3.32486
6	0.286284	4.2984
6	-0.263569	4.25857

Cluster #	x-coordinates	y-coordinates
6	0.0946303	4.04858
6	0.323287	4.45822
6	0.622007	4.47662
6	-0.217128	4.32798
6	0.243647	4.53147
6	-0.56877	3.40727
6	0.577585	4.21663
6	-0.248929	5.27933
6	-0.990793	3.8323
6	-0.589283	3.81054
6	0.724117	4.20198
6	-0.554375	4.09256
6	-0.525596	4.04451
6	-0.121267	3.08932
6	0.214802	4.802
6	0.0593401	3.75914
6	0.300533	3.87956
6	0.153858	3.17574
6	0.252946	3.61422
6	0.0460974	4.23559
6	0.151107	3.84503
6	-0.690217	4.21754
6	-0.515965	3.74699
6	-0.109494	3.6508
6	0.718708	3.85472
6	0.601063	4.32685
6	0.194383	4.77808
6	0.603961	3.25839
6	-0.610043	3.70249
6	0.43602	3.82369
6	0.444846	4.01725
6	0.844407	4.34777
6	0.307099	3.70642
6	-0.329707	3.78596
6	-0.00161739	3.88852
6	-0.28156	4.75253
7	5.12099	4.00031
7	4.09926	4.76627
7	4.79617	4.43093
7	3.28071	4.40062
7	3.62578	4.4691
7	4.71485	3.69164
7	4.28083	4.29032
7	3.8492	4.56995
7	3.53145	4.00683

Cluster #	x-coordinates	y-coordinates
7	4.28603	4.46554
7	4.13996	3.9174
7	4.42046	4.2479
7	3.30614	3.84257
7	3.92437	3.12806
7	3.90017	3.98961
7	4.16854	3.86099
7	3.56403	4.62612
7	3.41247	3.07497
7	4.13162	4.46009
7	4.07877	3.94274
7	3.51849	4.04574
7	3.89145	4.17386
7	4.65176	3.47697
7	3.04358	3.08821
7	3.8724	3.88686
7	4.0685	3.72569
7	4.16617	3.07653
7	4.39104	3.53819
7	3.91191	4.67912
7	5.42823	3.6881
7	3.78436	4.04065
7	4.12998	4.04376
7	4.14224	4.09543
7	4.01635	4.03918
7	4.28739	4.29558
7	4.18501	3.92994
7	3.55602	4.05028
7	4.12481	3.10731
7	4.03737	4.73642
7	3.59103	3.84966
7	3.94111	3.64217
7	4.38946	4.20032
7	3.04202	3.64214
7	3.08171	3.76405
7	4.39057	3.57686
7	4.55546	3.51822
7	3.832	3.85653
7	3.79331	3.11332
7	3.86225	4.17737
7	4.0684	3.98073
8	7.75143	4.61778
8	8.22714	4.22969
8	7.84117	4.3709
8	7.47462	4.24503

Cluster #	x-coordinates	y-coordinates
8	8.1275	3.99632
8	8.1942	3.58048
8	8.67604	4.28992
8	7.57851	3.75935
8	7.58709	4.14592
8	8.1836	4.04037
8	7.91594	3.81041
8	7.27513	3.84654
8	7.74604	3.97933
8	7.79332	4.70674
8	8.16827	2.87172
8	7.46523	3.96342
8	7.75331	3.38503
8	7.55699	3.70213
8	8.1122	3.51454
8	8.15801	3.49785
8	8.03032	3.80335
8	8.57628	4.39155
8	8.58271	4.49227
8	7.3175	4.48151
8	8.1247	4.56108
8	7.95457	3.4092
8	7.89296	3.25791
8	8.32101	2.82374
8	8.24601	3.81752
8	7.81918	4.45099
8	8.60989	3.61028
8	8.07377	4.46998
8	8.14262	4.33649
8	7.75666	3.81313
8	8.03759	3.64959
8	8.51334	2.74173
8	8.33296	4.62972
8	8.06287	3.06728
8	6.69428	4.05078
8	8.77707	4.47377
8	7.61708	4.27038
8	7.82067	3.43311
8	7.91851	4.07968
8	8.38736	3.20104
8	8.14075	2.9079
8	7.79916	3.66591
8	7.74245	4.05201
8	7.72474	3.81593
8	7.90007	3.37467

Cluster #	x-coordinates	y-coordinates
8	7.89377	4.27223
9	12.4125	4.43672
9	11.6493	3.64278
9	10.7694	3.71925
9	11.8119	3.84882
9	12.38	4.0457
9	10.9896	3.91538
9	12.4815	4.08317
9	11.7358	3.0295
9	12.4167	3.36762
9	11.031	3.90484
9	11.6985	4.03793
9	12.0748	3.60231
9	11.3596	3.47605
9	10.8776	4.01251
9	11.542	3.60522
9	12.1067	3.92075
9	11.1656	3.22323
9	12.1949	4.88616
9	12.9641	3.45367
9	12.2669	4.03248
9	12.0941	4.04107
9	11.4279	3.5431
9	12.1765	4.09161
9	12.7585	5.1521
9	11.8391	4.93748
9	12.5375	3.5063
9	11.4529	4.47693
9	11.9743	4.85003
9	12.2291	4.66045
9	11.8464	3.56291
9	12.1824	2.46943
9	12.4651	4.12443
9	11.7544	3.92842
9	12.5703	4.38635
9	10.972	3.4972
9	12.3385	4.626
9	11.2561	4.23074
9	11.4579	4.05632
9	12.2547	4.21181
9	11.1748	4.15105
9	12.4465	4.59165
9	12.6514	4.56934
9	12.8069	3.72996
9	12.4315	4.31298

Cluster #	x-coordinates	y-coordinates
9	12.4504	4.61528
9	12.4951	4.21744
9	11.23	3.07835
9	11.5277	4.80729
9	11.674	4.136
9	12.1898	4.13033
10	15.6164	3.62238
10	15.7808	4.22362
10	16.3857	3.33265
10	16.3511	4.5521
10	16.5444	3.84708
10	17.1476	4.70602
10	15.3444	4.61923
10	15.0533	4.44829
10	15.5285	3.80653
10	16.8356	4.43457
10	15.705	3.76347
10	16.5164	3.74189
10	15.5242	4.27841
10	16.5008	3.84486
10	16.1526	3.76838
10	15.8781	4.0774
10	17.0915	4.50208
10	16.7783	3.32227
10	16.6931	3.93892
10	15.4856	3.63898
10	16.4754	3.79331
10	16.8882	4.45603
10	16.548	3.69698
10	15.5729	4.05186
10	16.7725	3.21395
10	16.3039	4.27339
10	15.4969	3.2925
10	16.7983	3.52937
10	15.5137	3.73933
10	15.5062	3.65765
10	15.9966	4.09464
10	15.8612	3.92463
10	15.698	3.36379
10	16.0353	4.08678
10	16.4796	3.68646
10	16.7391	3.62718
10	15.0939	4.22233
10	16.3753	3.60646
10	15.7244	5.25474

Cluster #	x-coordinates	y-coordinates
10	16.0173	4.72567
10	16.4282	3.36528
10	15.806	4.02601
10	16.9523	4.07561
10	16.6283	4.5147
10	16.0069	4.02876
10	16.0536	3.4768
10	15.8639	4.01898
10	16.3885	3.56908
10	15.9764	3.87393
10	16.4705	4.02086
11	0.960097	7.73212
11	0.268969	7.63238
11	0.167427	7.87609
11	-0.208084	8.45318
11	0.315124	8.85375
11	0.0298709	7.39016
11	-0.193779	7.11617
11	0.797659	7.52694
11	-0.905043	8.22467
11	-0.589853	8.37255
11	-0.219029	8.4334
11	0.0648199	8.52684
11	-0.407643	7.76886
11	0.500173	6.40104
11	0.665297	8.4837
11	0.298141	8.58317
11	-0.770565	7.96648
11	-0.0400082	7.86671
11	0.385382	8.65497
11	-0.347176	8.46211
11	0.19741	8.20269
11	0.603425	7.99696
11	0.822034	8.08116
11	-1.12992	7.71134
11	0.0567572	8.30997
11	0.377609	8.12173
11	0.0164275	8.22395
11	-0.167131	8.22701
11	-0.56829	7.96865
11	-0.283002	8.23038
11	0.00782418	7.80963
11	0.157824	7.63202
11	0.25873	7.51951
11	0.353849	6.59608

Cluster #	x-coordinates	y-coordinates
11	-0.31066	9.09027
11	-0.309796	8.73867
11	-0.375442	8.00483
11	-0.203053	8.59388
11	0.589527	7.24537
11	0.380273	8.00471
11	-0.101529	7.64235
11	0.225858	7.01106
11	1.30326	8.27878
11	0.311248	7.37571
11	-0.0806733	6.91285
11	0.313292	7.9038
11	0.374351	8.23684
11	0.211268	7.29448
11	0.767362	7.80568
11	-0.320876	7.21244
12	4.68024	8.57821
12	3.96996	8.36285
12	4.15641	7.68476
12	3.78038	7.5274
12	4.83574	8.14793
12	3.86085	7.86433
12	4.4791	8.32008
12	4.31831	8.30433
12	3.66603	7.10271
12	3.73112	7.31916
12	3.17828	7.69679
12	3.89709	9.03181
12	3.23441	9.32306
12	4.45769	8.5976
12	4.1642	7.4149
12	4.83152	7.09416
12	4.0012	7.5383
12	3.05473	8.16368
12	3.29617	8.59483
12	3.19333	7.33856
12	3.37514	7.12922
12	3.8009	8.70887
12	3.21752	7.7531
12	4.53309	8.09744
12	3.83364	7.69513
12	3.787	8.33383
12	4.90664	7.83815
12	4.43573	8.06951
12	3.35295	8.41184

Cluster #	x-coordinates	y-coordinates
12	4.31239	8.15452
12	4.73138	7.79582
12	5.06055	8.35499
12	2.78393	7.49387
12	4.13324	7.90567
12	4.38604	7.77832
12	4.13399	8.63185
12	3.54178	8.14829
12	4.51838	8.22596
12	3.78994	7.66744
12	4.82928	7.8602
12	3.75902	8.74235
12	4.31011	8.94762
12	3.75507	8.56572
12	4.1939	7.6935
12	4.22376	8.50417
12	4.38278	7.09826
12	3.91087	7.82775
12	3.58564	7.63392
12	3.14092	8.28153
12	3.85931	8.63808
13	7.92143	7.50217
13	8.04183	7.67034
13	7.7551	8.32385
13	8.04784	8.94517
13	8.13209	8.88923
13	8.91866	8.56629
13	9.19659	7.12771
13	8.45603	8.28192
13	8.95202	7.95442
13	7.76694	8.04536
13	7.66799	7.90887
13	7.85931	7.83325
13	7.83502	8.29738
13	8.74866	7.68854
13	8.02684	7.68714
13	7.6023	8.07776
13	8.03847	7.99219
13	7.32241	8.10494
13	7.89047	7.86904
13	8.3893	7.40224
13	8.59369	9.12513
13	8.80877	8.14616
13	7.11003	7.19754
13	8.11603	7.96069

Cluster #	x-coordinates	y-coordinates
13	8.07732	7.46686
13	7.89244	7.38287
13	7.99066	8.30746
13	8.22959	8.35572
13	8.70381	8.44453
13	6.76642	7.35729
13	7.83067	8.23185
13	7.34521	8.97171
13	7.85309	7.93091
13	7.88151	8.15409
13	8.29412	7.06578
13	7.93634	7.5888
13	7.80825	8.37044
13	7.63512	8.36943
13	8.68297	8.07262
13	8.2376	7.39796
13	8.29697	7.51081
13	7.92087	7.97239
13	8.33227	8.76401
13	7.92374	8.88555
13	8.41151	7.87882
13	7.56211	8.09314
13	8.55807	8.14289
13	7.53021	8.40386
13	8.79938	8.35518
13	7.53421	7.37478
14	12.0913	8.5472
14	11.9962	8.64115
14	11.5469	8.51513
14	11.9789	8.43984
14	12.1412	8.20644
14	12.6601	7.5557
14	13.0644	7.70892
14	11.7011	8.49028
14	12.5135	7.57758
14	11.9616	8.12205
14	12.8653	8.20649
14	12.3768	7.88295
14	11.4666	7.18164
14	11.6957	8.23743
14	12.1107	7.93288
14	11.9642	7.85492
14	12.0685	6.71084
14	12.2522	8.80025
14	10.9836	7.33868

Cluster #	x-coordinates	y-coordinates
14	12.2939	8.86685
14	12.464	8.0346
14	10.7939	7.99247
14	12.0956	7.80047
14	11.7806	7.7047
14	12.5594	8.49704
14	11.7612	8.00734
14	12.2158	8.15015
14	12.222	7.62585
14	12.0026	7.64398
14	12.1633	8.48642
14	11.7978	8.17095
14	11.0861	8.32811
14	11.1796	7.32614
14	12.106	7.86439
14	11.7222	8.66192
14	12.7728	7.28232
14	11.343	7.68985
14	11.7779	8.81476
14	11.4938	7.25105
14	11.8353	8.05638
14	12.1857	7.75175
14	11.3713	7.4544
14	11.3924	7.31864
14	12.5373	7.62522
14	11.6739	7.98703
14	11.5675	7.96418
14	12.1804	8.22725
14	12.4105	8.52416
14	12.2021	7.57355
14	12.9334	8.28669
15	15.6067	7.42603
15	15.4521	7.65593
15	15.9871	7.71588
15	15.9522	7.47597
15	15.8043	7.01559
15	15.565	7.58807
15	16.9317	7.46299
15	16.4349	8.26702
15	16.9631	7.67646
15	15.9684	8.42781
15	16.1381	7.80399
15	16.224	8.96472
15	17.0446	8.02146
15	15.9193	7.886

Cluster #	x-coordinates	y-coordinates
15	15.4992	8.29895
15	16.3626	7.49244
15	15.5585	7.69286
15	16.6166	8.68676
15	15.8831	7.72595
15	16.0834	8.26312
15	16.0097	7.70442
15	15.9371	7.75194
15	16.1358	8.21526
15	16.3515	6.67749
15	16.5317	8.14592
15	16.3966	8.42154
15	15.77	7.9248
15	16.8513	8.32948
15	17.5652	7.68939
15	16.1936	8.37678
15	15.9618	8.0286
15	16.103	8.14577
15	16.8444	8.08015
15	15.8665	7.7121
15	15.9911	7.93134
15	14.9977	8.88423
15	14.7585	7.8228
15	15.1706	7.65506
15	16.649	7.3848
15	17.1845	7.90201
15	16.035	8.26769
15	15.1473	8.20698
15	16.7763	7.97767
15	15.436	7.8137
15	16.4963	7.79129
15	16.0836	7.84868
15	16.7272	9.24403
15	15.7219	7.99965
15	16.1768	8.58904
15	16.0652	7.55158
16	-0.895134	11.9365
16	-0.010683	11.3672
16	-0.777263	11.4042
16	0.42972	11.9252
16	0.299282	12.3782
16	0.0152985	11.4955
16	-0.392803	11.9747
16	-0.324311	12.3251
16	-0.571363	11.9163

Cluster #	x-coordinates	y-coordinates
16	0.675694	12.3052
16	0.438772	11.5392
16	0.286779	11.936
16	-0.567493	11.7442
16	-0.369303	12.4217
16	0.0931385	11.8368
16	0.361797	12.1267
16	0.898352	11.827
16	-0.488508	12.6069
16	-0.792566	11.7095
16	0.958065	12.4165
16	-0.0116939	12.9041
16	0.979147	12.4358
16	-0.608957	11.291
16	0.449273	11.8459
16	0.533961	11.6164
16	-0.389316	11.0781
16	0.062604	11.902
16	1.08294	11.9168
16	0.387406	11.9141
16	-0.439769	12.6449
16	0.0813357	11.2381
16	0.389551	11.7064
16	0.584351	12.1126
16	0.16898	12.786
16	-0.00103628	11.4132
16	-0.416814	11.4972
16	-0.0151941	12.133
16	0.0582517	11.7473
16	-0.166667	11.3549
16	-0.509524	11.7654
16	-0.48356	12.2409
16	-0.406631	12.6576
16	0.201283	11.8355
16	-0.19275	11.2001
16	0.155949	12.1936
16	0.121677	12.3693
16	-0.664493	12.3762
16	-0.00782211	11.7126
16	0.684893	12.04
16	-0.0741672	12.863
17	4.01243	11.9407
17	4.77506	11.8705
17	4.43272	11.6807
17	4.15874	13.0479

Cluster #	x-coordinates	y-coordinates
17	3.73354	12.513
17	3.4866	12.3983
17	3.50606	12.8169
17	3.62673	11.9501
17	3.90154	11.2866
17	4.12457	11.6808
17	4.19562	11.7985
17	3.68511	11.857
17	4.26974	12.2497
17	3.68762	12.4879
17	4.27699	11.8117
17	3.80837	12.1566
17	4.19353	12.3567
17	3.19957	12.6575
17	4.02617	12.0253
17	2.99954	12.0392
17	4.37059	11.9651
17	3.73941	11.0846
17	3.70757	11.9529
17	3.90727	12.7242
17	4.03791	12.1498
17	3.94755	12.5919
17	3.1792	11.2432
17	4.25806	11.6001
17	3.92544	12.5774
17	4.04524	12.0403
17	4.13304	12.3553
17	3.76669	12.3731
17	3.66683	12.8513
17	4.23463	12.155
17	4.62016	12.1053
17	4.60687	12.1912
17	4.4025	11.7338
17	4.48491	11.977
17	3.61421	11.658
17	4.07732	12.0365
17	4.20694	12.7509
17	4.38396	12.3777
17	3.65763	12.0879
17	3.38039	12.1605
17	3.38809	11.6185
17	4.26128	11.3694
17	3.9215	11.882
17	4.04585	11.7825
17	3.49288	12.4623

Cluster #	x-coordinates	y-coordinates
17	4.3107	11.9288
18	7.43843	12.1812
18	7.51689	11.4414
18	8.3159	11.8275
18	7.64893	10.5016
18	7.54702	11.8348
18	7.57649	12.8092
18	7.45405	11.9973
18	7.90009	12.5679
18	7.74833	11.543
18	8.33514	12.7039
18	7.76897	12.0263
18	8.12061	12.5927
18	7.12735	11.9193
18	8.45655	11.889
18	8.30073	11.718
18	8.16082	11.6652
18	7.8184	12.5965
18	7.80666	10.8182
18	7.51579	13.5369
18	8.53757	11.311
18	8.67581	11.7085
18	7.80391	11.3441
18	8.59202	12.3203
18	6.82211	12.5992
18	8.17012	11.778
18	7.99554	12.7754
18	7.68904	11.8984
18	8.66656	12.7464
18	7.87001	12.2422
18	7.96458	12.5465
18	7.15197	11.6687
18	8.35926	11.8226
18	7.98205	11.6608
18	7.59235	11.4781
18	7.77592	12.5508
18	8.56814	12.382
18	7.8595	12.5855
18	7.28441	11.3268
18	8.50099	11.3462
18	8.49807	12.1443
18	7.63616	11.6006
18	7.84591	11.8382
18	8.43725	11.7566
18	8.1206	11.932

Cluster #	x-coordinates	y-coordinates
18	8.23799	12.0281
18	7.25023	12.3464
18	7.50216	12.337
18	8.45813	12.559
18	8.45142	10.9584
18	7.56261	11.7353
19	12.4601	12.3125
19	11.9114	11.894
19	11.5523	11.8456
19	11.5406	11.3597
19	12.487	12.2594
19	12.6436	11.7906
19	12.1418	11.7946
19	11.9115	12.5573
19	11.0342	12.7842
19	11.4311	11.9743
19	12.4075	12.0682
19	13.0317	11.8352
19	12.128	12.7074
19	11.782	12.278
19	12.1513	11.7874
19	12.4304	11.2998
19	11.9598	12.0295
19	11.5518	11.3765
19	12.9343	12.2611
19	12.0457	12.1194
19	12.1131	12.261
19	11.9418	10.7807
19	11.7642	11.8149
19	12.0735	12.273
19	11.9685	12.6215
19	12.5697	11.3998
19	12.3929	12.2206
19	11.8751	11.9774
19	11.8029	12.4356
19	11.4502	12.6382
19	12.2321	11.3786
19	12.52	11.213
19	12.4148	12.5081
19	11.6116	12.5874
19	11.352	12.7665
19	11.4806	12.0839
19	12.2257	11.3333
19	12.3641	12.6633
19	11.3619	12.5001

Cluster #	x-coordinates	y-coordinates
19	11.844	11.5821
19	11.7392	11.627
19	11.9169	12.4779
19	12.2605	11.1183
19	12.022	11.3724
19	11.9197	11.6289
19	12.2381	12.618
19	11.3699	12.125
19	12.5291	11.877
19	11.1549	11.7365
19	12.7088	12.2883
20	17.319	12.1197
20	16.0085	11.2975
20	16.3937	12.1507
20	15.5	12.3055
20	16.3374	11.3238
20	16.7728	11.8459
20	15.9321	10.8666
20	15.8277	12.1153
20	15.9089	12.4748
20	15.5781	12.7923
20	16.5172	12.2267
20	16.0484	12.1516
20	15.4764	11.3945
20	16.2064	11.7892
20	15.2823	12.3237
20	16.068	12.0182
20	16.3836	12.0875
20	16.2067	12.2755
20	16.6593	12.3142
20	16.5074	12.0386
20	15.4706	12.0209
20	16.1432	11.3607
20	16.6127	12.6656
20	16.5309	13.1719
20	16.5535	11.4719
20	16.0259	12.7326
20	16.2549	12.1135
20	16.0301	12.1509
20	16.2002	12.551
20	15.7403	12.4722
20	15.9022	11.9524
20	16.062	12.3973
20	16.224	12.3431
20	16.2654	11.5798

Cluster #	x-coordinates	y-coordinates
20	16.855	12.5128
20	15.7019	12.0843
20	15.4499	11.585
20	15.1437	12.9077
20	16.4504	12.1194
20	16.4674	11.9249
20	16.3184	11.2705
20	15.8259	11.8885
20	16.1919	11.8333
20	16.2748	12.3323
20	15.4829	12.6298
20	16.0013	12.4242
20	16.3041	12.9095
20	16.2549	11.0824
20	16.2623	12.6437
20	16.0895	11.901

Appendix N

Fourty
Number of dimensions: 2
Number of clusters: 40
Number of points: 1000

Cluster #	x-coordinates	y-coordinates
1	-0.796472	-0.538789
1	1.07415	0.283016
1	-0.0882453	-0.0154108
1	0.238279	-0.0570198
1	0.708839	0.165585
1	0.163807	-0.0402066
1	-0.200248	0.692106
1	0.0641332	-0.47997
1	-0.388032	-0.415745
1	-0.751184	0.672181
1	-0.607715	-0.814858
1	0.26011	0.335198
1	-0.546444	0.251202
1	0.101309	0.424941
1	0.436923	0.784839
1	-0.0189449	0.603242
1	-0.476297	0.00590647
1	0.199583	-0.177538
1	-0.981166	0.472785
1	0.129555	0.156186
1	0.824547	-0.69325
1	0.223874	-0.0875816
1	-0.601323	-0.569979
1	0.762652	0.387553
1	0.120625	0.537594
2	3.01053	-0.212716
2	3.79178	-0.0714351
2	3.97677	0.0425788
2	4.21977	-0.737742
2	4.7352	-0.275757
2	4.29667	-0.0791719
2	3.9759	-0.0661088
2	4.04327	-0.0410543
2	4.01564	0.0331227
2	4.27207	0.113586
2	4.6347	-0.377884
2	3.72788	-0.705706
2	4.55564	-0.0716247
2	3.92393	-1.42917

Cluster #	x-coordinates	y-coordinates
2	3.11443	-0.239851
2	4.30702	-0.107685
2	4.29674	0.60503
2	4.03765	0.00249412
2	5.15323	0.276954
2	4.29675	0.152049
2	4.00786	-0.0671036
2	3.69175	-0.550353
2	2.87988	0.363631
2	3.74257	1.18664
2	3.97348	0.506361
3	8.32419	-1.17856
3	7.73708	1.44513
3	7.1999	0.28729
3	8.07462	0.337588
3	7.54879	-0.310762
3	7.56662	-0.704622
3	9.24661	-0.121555
3	8.81637	0.276002
3	8.64203	0.415519
3	7.44744	-0.46857
3	7.41791	0.548326
3	8.29063	-0.846479
3	7.9532	-0.955921
3	7.6995	0.31141
3	7.61241	0.37876
3	7.76966	-0.232545
3	8.11709	0.103819
3	7.90047	-0.517087
3	7.65481	-0.248699
3	8.65947	-0.370796
3	7.81227	0.269439
3	8.38809	-0.17127
3	7.56891	0.538494
3	7.61015	0.278909
3	7.84017	-0.790742
4	12.2809	0.303709
4	11.1523	-0.336136
4	11.3569	-0.497622
4	12.4477	-0.291661
4	11.7262	0.552916
4	12.3093	-0.279156
4	11.9166	0.447148
4	12	0.00645392
4	13.0704	0.591449

Cluster #	x-coordinates	y-coordinates
4	11.6717	0.569751
4	11.4784	0.698509
4	12.8676	-0.979057
4	12.503	-0.280088
4	11.4247	0.0910561
4	13.0199	-0.0895692
4	12.9604	0.265662
4	12.1187	-0.110861
4	13.0311	-0.226739
4	11.2842	0.562014
4	12.2879	0.234708
4	11.9574	-0.36161
4	12.3445	-0.725916
4	12.8942	0.145759
4	11.6446	-0.416159
4	11.6631	-1.54415
5	15.6993	-0.416461
5	15.9364	0.338083
5	16.6751	-0.141313
5	16.273	0.777651
5	15.7235	0.742506
5	15.8778	-0.186271
5	15.5987	-0.302537
5	16.8896	1.03886
5	15.4213	-0.609232
5	16.9586	0.130016
5	15.7548	0.202269
5	16.5594	0.52837
5	16.5707	-0.31159
5	14.5954	-0.512358
5	15.8228	0.130091
5	16.5049	-0.0937206
5	16.0924	-0.392365
5	15.6908	0.233581
5	16.0681	0.133851
5	16.525	0.902382
5	15.965	0.91102
5	16.6208	0.790518
5	16.271	-0.479934
5	15.7912	-0.0525799
5	15.7649	0.199848
6	-0.400469	4.53691
6	-0.0968634	4.50867
6	-0.187944	4.02405
6	-0.873714	3.76999

Cluster #	x-coordinates	y-coordinates
6	0.935654	5.37757
6	-0.346292	3.73288
6	1.11593	4.0069
6	0.728485	4.66706
6	0.175503	5.11378
6	0.552438	3.62921
6	-0.0639243	4.12319
6	0.00233343	4.02054
6	0.487888	3.76658
6	0.237496	3.4453
6	-0.586712	4.41836
6	0.25498	4.32225
6	0.753669	3.8778
6	-0.255619	3.6784
6	-0.0664025	3.56703
6	0.50721	4.28617
6	0.175297	3.96575
6	1.10055	3.66583
6	0.69815	4.33183
6	0.103895	3.73596
6	0.882744	4.82995
7	3.60791	4.35873
7	4.02089	3.89618
7	4.4513	4.28694
7	4.19773	4.09148
7	4.10998	3.95141
7	4.18485	3.69915
7	4.75913	4.60196
7	3.92758	4.06112
7	3.82976	4.37559
7	3.62118	3.56982
7	3.01888	3.89984
7	3.74945	4.49419
7	3.41476	4.53944
7	4.00047	4.8749
7	4.48065	4.44873
7	3.93146	3.67659
7	3.75353	3.79958
7	4.16299	4.31182
7	3.99231	3.93775
7	4.15217	3.4812
7	4.3181	3.75948
7	4.67728	4.65658
7	3.79562	3.55473
7	3.88985	3.79529

Cluster #	x-coordinates	y-coordinates
7	3.93726	4.27393
8	8.29873	3.38475
8	8.22472	3.16729
8	7.72151	3.84654
8	7.35678	4.08594
8	8.80231	4.07732
8	7.95642	4.54662
8	7.41659	3.64959
8	7.71529	3.93861
8	7.79562	3.76861
8	7.56522	3.882
8	7.69313	3.5759
8	6.66802	4.34386
8	7.99377	4.19416
8	8.219	4.25412
8	7.0989	4.72362
8	8.50083	4.93503
8	9.34245	4.51824
8	7.88111	3.89439
8	7.86579	3.83493
8	8.60149	3.59284
8	7.99102	3.7765
8	7.87104	4.22742
8	8.2503	4.39172
8	7.02483	4.00498
8	8.34851	4.62209
9	12.0397	3.56849
9	12.5822	4.03329
9	12.5281	3.82999
9	12.5537	4.04238
9	12.4262	4.38597
9	12.579	3.99377
9	11.9759	4.12265
9	13.2895	4.50097
9	12.3915	4.28201
9	11.9145	3.38511
9	12.3275	4.50976
9	12.2082	4.31848
9	11.9116	3.76931
9	12.2697	4.04646
9	12.6677	4.44352
9	12.112	3.17371
9	12.3845	3.37243
9	11.8085	4.68242
9	11.9154	3.66935

Cluster #	x-coordinates	y-coordinates
9	12.6266	4.77965
9	11.7132	4.10217
9	12.3003	4.86216
9	11.6807	4.44927
9	11.2805	4.15022
9	11.4623	3.63941
10	15.8416	3.52499
10	15.5225	2.84921
10	16.7106	4.67125
10	15.8422	3.16156
10	15.5107	3.45824
10	16.0057	4.56791
10	16.594	4.48895
10	16.3173	4.23208
10	16.1673	3.45855
10	16.3178	3.36127
10	17.0975	3.20025
10	15.9159	3.75032
10	15.334	3.40451
10	15.7469	3.87649
10	17.1669	3.95842
10	16.311	3.27976
10	15.8008	4.54787
10	16.1231	3.68588
10	16.3409	4.08619
10	16.5888	4.41906
10	15.6103	4.48753
10	16.6368	3.85915
10	16.3442	3.76203
10	15.3046	4.00494
10	15.9868	4.47535
11	-0.238043	7.77529
11	-0.156919	7.91536
11	-0.477023	8.4939
11	1.05136	8.1613
11	-0.0806346	8.01404
11	0.437855	8.90328
11	0.854143	7.79479
11	0.0205852	7.81385
11	0.0573788	7.36663
11	0.0146803	8.31008
11	-0.000501475	8.15822
11	0.377673	7.26594
11	0.0652555	7.36144
11	-0.725486	8.16953

Cluster #	x-coordinates	y-coordinates
11	-0.561303	6.94982
11	-0.262935	7.89618
11	-0.126494	7.98927
11	0.122046	7.31109
11	-0.233286	8.31813
11	-0.0951861	8.38905
11	-0.0459457	7.75927
11	0.291698	7.98718
11	0.789141	8.18664
11	0.195394	7.81626
11	0.183484	7.96095
12	3.82378	8.19359
12	2.64181	8.22515
12	3.68237	7.66394
12	3.94599	8.80704
12	4.21587	8.51938
12	4.4694	8.2143
12	4.64999	8.54899
12	4.25338	8.30637
12	3.63877	7.91247
12	3.08302	7.77863
12	3.5907	7.34658
12	4.9149	7.91834
12	3.49211	7.41565
12	3.965	8.9205
12	4.20597	7.05887
12	4.09382	7.81821
12	4.58079	8.10268
12	4.34856	7.87344
12	3.80219	7.44517
12	4.39093	7.50505
12	3.28161	7.42356
12	4.95557	7.18295
12	4.7	8.31714
12	3.65486	8.4101
12	3.69912	8.51398
13	7.55276	8.55086
13	7.78987	6.79719
13	8.55566	8.11632
13	8.02022	8.3045
13	7.54463	8.69394
13	8.36397	7.81641
13	7.72049	8.72943
13	8.10773	7.82423
13	8.14186	8.57309

Cluster #	x-coordinates	y-coordinates
13	8.64493	7.5114
13	8.93473	7.40131
13	7.83365	7.17706
13	9.01344	8.44753
13	7.1984	7.65443
13	8.57559	8.26139
13	8.10794	7.92101
13	6.87298	7.54991
13	7.95203	7.61078
13	7.59422	8.04617
13	7.70636	8.95698
13	7.96514	7.94858
13	7.8508	7.94113
13	7.02758	8.31633
13	8.59756	8.34825
13	7.61358	8.11069
14	11.8626	7.91218
14	11.4723	8.44743
14	12.2005	8.2181
14	11.6484	7.13005
14	12.0303	9.45924
14	12.2685	7.4675
14	11.3471	7.75341
14	11.6244	7.4704
14	12.2907	8.84796
14	11.8822	8.21831
14	12.1229	7.6639
14	12.4616	7.547
14	12.625	7.61878
14	12.0227	8.45424
14	11.4506	8.85839
14	11.4776	8.94921
14	12.3313	7.87894
14	11.8247	8.12964
14	12.2947	8.23479
14	12.3245	7.58659
14	11.4545	9.28397
14	11.7065	8.00276
14	11.5713	8.60388
14	13.0731	7.54767
14	11.6684	8.06806
15	15.2812	8.45972
15	15.8221	7.89977
15	16.395	7.51146
15	16.2387	7.98565

Cluster #	x-coordinates	y-coordinates
15	15.4244	7.3249
15	15.5004	8.01048
15	16.1344	7.58169
15	15.52	8.88884
15	17.1112	8.9034
15	14.8586	8.37262
15	15.9951	7.53108
15	16.2455	8.18902
15	15.8333	7.76508
15	15.4944	7.61279
15	16.6565	8.38178
15	15.494	8.08684
15	14.5039	8.28948
15	16.9246	8.62038
15	16.2885	7.38404
15	16.104	7.82267
15	16.1326	7.69581
15	15.8592	8.30326
15	15.3785	7.97671
15	15.6521	7.99903
15	15.6945	8.73113
16	0.909529	13.4163
16	0.651567	11.5584
16	0.359847	11.9278
16	0.223655	11.7149
16	0.407562	11.7086
16	0.0578853	12.3926
16	-0.656921	12.4511
16	-0.35719	12.6263
16	0.140048	11.842
16	-0.5763	11.0048
16	0.268712	11.109
16	0.141295	11.3359
16	0.530255	11.7258
16	-0.271167	12.9722
16	1.56196	11.7119
16	0.122903	11.5137
16	0.498132	11.2111
16	-0.417756	11.825
16	0.193604	11.5293
16	-0.645529	12.3297
16	0.148739	12.2177
16	-0.849397	12.5498
16	1.25394	12.0534
16	0.543383	11.7283

Cluster #	x-coordinates	y-coordinates
16	0.457112	11.0648
17	3.24327	11.9437
17	3.59604	11.8752
17	3.62178	12.4652
17	4.0727	12.1658
17	3.88346	11.6055
17	4.33174	11.9764
17	3.62406	11.3182
17	4.17453	12.8809
17	4.219	12.534
17	4.57884	12.6508
17	3.77729	11.6719
17	5.15255	12.5581
17	3.52745	12.1592
17	3.86597	12.1409
17	3.81223	12.6096
17	4.02111	11.7681
17	4.45574	11.5244
17	3.86023	12.7595
17	4.62775	11.3413
17	3.911	11.3282
17	4.24901	12.0052
17	3.97988	11.2634
17	4.31996	11.9319
17	3.4511	12.1822
17	3.83904	12.3352
18	7.7797	11.2457
18	8.11967	12.1642
18	8.1139	11.6601
18	8.55865	11.8363
18	8.83061	12.5126
18	8.27982	11.7085
18	8.25199	11.8686
18	8.67451	11.3617
18	7.95686	13.5027
18	7.88611	12.9548
18	7.63327	11.5858
18	8.50375	11.3619
18	6.89892	11.13
18	7.42687	12.6222
18	8.69137	12.1683
18	7.97624	12.0905
18	8.32536	11.9486
18	7.79148	12.0795
18	8.03285	11.8389

Cluster #	x-coordinates	y-coordinates
18	7.81757	12.2054
18	6.74727	12.1451
18	8.46834	11.8646
18	8.27303	12.2523
18	8.94691	11.0981
18	8.31322	10.8229
19	11.8199	12.0577
19	12.7762	11.2258
19	11.6837	11.9465
19	12.6047	12.3528
19	11.5454	11.8215
19	12.47	12.0257
19	11.5768	12.5944
19	11.786	12.0878
19	12.2591	11.8527
19	11.0918	11.328
19	12.5757	12.1613
19	11.9175	11.0947
19	13.4602	12.1927
19	12.2272	11.4026
19	11.9333	11.9166
19	11.8061	11.1773
19	12.1671	11.6176
19	11.8557	12.3263
19	11.7439	12.0495
19	12.322	12.5175
19	11.4164	11.9411
19	12.141	11.4017
19	12.6801	11.8326
19	11.6357	11.8024
19	12.4142	12.689
20	15.128	11.7025
20	15.6766	11.893
20	15.9124	10.8533
20	16.0716	11.8832
20	16.0578	11.9422
20	16.1882	12.6373
20	16.2995	11.8284
20	16.0641	11.9675
20	16.0467	11.2401
20	15.3984	11.9482
20	16.4227	11.3749
20	16.1368	11.8614
20	15.8749	12.3062
20	15.4924	11.76

Cluster #	x-coordinates	y-coordinates
20	16.0283	12.0546
20	16.9585	12.58
20	15.0059	11.7376
20	16.2393	12.1735
20	16.3895	12.0291
20	16.0321	11.8056
20	15.5401	12.5557
20	15.9278	11.8326
20	15.9384	12.5611
20	15.5917	10.3039
20	15.5422	11.2224
21	0.0846935	16.1015
21	-0.0629106	14.9544
21	-0.534322	15.5722
21	-0.728986	15.4731
21	-0.870909	17.1145
21	0.0762788	15.5467
21	-0.222623	16.5637
21	-0.643904	16.6066
21	0.2528	16.4949
21	-0.296353	16.4561
21	0.135365	15.3969
21	0.390075	15.6173
21	-1.64307	16.7766
21	-0.0989464	16.0314
21	0.391972	15.5105
21	0.509406	16.6987
21	0.254012	15.7446
21	-0.500488	16.1022
21	-0.082121	16.0113
21	0.138895	16.5932
21	0.550171	15.8634
21	-1.02153	16.0635
21	0.306447	15.8089
21	0.370815	16.3322
21	0.32258	16.7707
22	3.61691	16.5064
22	3.94788	16.389
22	4.30386	16.92
22	4.24113	15.8733
22	5.11659	16.4741
22	4.63458	15.7902
22	3.94288	16.2742
22	4.21438	15.7854
22	3.58873	16.7653

Cluster #	x-coordinates	y-coordinates
22	3.90376	15.8847
22	5.03058	16.1309
22	3.79911	16.2024
22	4.17266	15.9019
22	4.44495	15.5082
22	3.76016	16.1307
22	4.27535	16.5386
22	3.49508	16.978
22	4.51192	16.1662
22	3.82034	15.7578
22	2.83278	16.0693
22	4.94706	15.2291
22	3.9746	15.4755
22	3.70027	16.5092
22	4.10334	16.2068
22	4.09859	15.6414
23	7.78753	15.7056
23	8.2181	15.8382
23	8.90288	16.9498
23	8.90292	16.3868
23	7.8362	15.561
23	7.31105	15.9613
23	8.15394	15.7817
23	8.18841	16.4766
23	8.42664	16.0937
23	7.73658	15.5944
23	8.93033	16.1828
23	8.36286	16.0578
23	8.62132	15.2249
23	7.75967	15.6315
23	8.93108	15.7171
23	7.66594	15.986
23	8.14733	15.3543
23	7.45126	16.1818
23	7.40407	16.4984
23	6.94429	16.0044
23	8.69791	16.1819
23	9.04102	16.5319
23	8.42015	16.3044
23	8.07374	16.4045
23	7.09762	15.8584
24	12.6891	16.427
24	11.0678	15.7628
24	12.2164	16.2068
24	12.0736	14.9181

Cluster #	x-coordinates	y-coordinates
24	11.7823	14.8911
24	11.7734	15.3871
24	12.1817	16.0019
24	12.1294	15.5623
24	11.0143	15.8563
24	12.4011	15.191
24	11.8097	15.9897
24	11.9433	17.297
24	12.5458	15.3446
24	12.1071	16.4812
24	11.3569	15.707
24	12.541	16.0168
24	12.5925	16.318
24	12.3012	15.5115
24	13.0071	16.7002
24	12.5459	15.688
24	11.8782	15.6954
24	11.9914	16.0748
24	11.9824	16.0934
24	11.9298	16.6214
24	11.4466	15.9549
25	15.7503	16.4381
25	16.3814	15.6721
25	17.0863	16.0025
25	15.8719	16.3205
25	15.9765	16.0626
25	15.8133	15.363
25	16.9764	16.6488
25	15.3837	16.2685
25	16.2352	16.1467
25	16.3933	15.7222
25	15.7801	16.4342
25	15.5581	16.3972
25	16.7404	16.621
25	16.1438	15.235
25	15.5668	16.3649
25	16.6804	15.6467
25	15.9326	16.1907
25	15.8264	16.4081
25	16.7502	16.5089
25	15.1435	16.723
25	16.0655	15.9349
25	16.6574	16.4854
25	15.551	16.2384
25	15.2866	16.2392

Cluster #	x-coordinates	y-coordinates
25	15.6152	16.9634
26	-0.194097	19.9816
26	0.742912	20.3
26	0.165944	19.9247
26	0.153585	20.4008
26	-0.558453	20.1525
26	-1.56186	19.7831
26	-0.137329	19.4478
26	-0.0252384	19.6199
26	0.301935	19.7208
26	-0.158652	20.5331
26	-0.327152	19.7398
26	0.326257	20.5679
26	0.560011	20.2291
26	-0.526231	19.3205
26	-0.0302972	19.5361
26	-0.584648	20.6194
26	0.21816	20.3753
26	-0.590461	20.0437
26	0.273079	20.4909
26	0.220026	19.8279
26	-0.41815	19.9536
26	0.029305	20.9649
26	-0.431118	20.5875
26	0.663752	18.383
26	1.25507	19.8302
27	3.55144	19.871
27	4.25725	20.1995
27	3.84064	20.0481
27	3.61563	19.6629
27	4.25054	20.5199
27	3.85858	20.1851
27	3.61588	19.7607
27	3.83433	20.5021
27	3.51751	19.7316
27	4.37949	20.1679
27	3.3942	20.0592
27	3.69171	19.7894
27	3.22681	20.067
27	3.82075	19.801
27	4.37699	20.2317
27	4.12257	19.9564
27	3.94093	21.016
27	4.30282	20.3047
27	3.16692	19.7924

Cluster #	x-coordinates	y-coordinates
27	3.51879	20.0978
27	4.22761	19.7197
27	3.12748	20.5049
27	3.81915	19.859
27	5.29412	20.6452
27	4.37891	20.393
28	8.47544	20.5488
28	8.13628	20.7827
28	8.21228	20.8708
28	8.22139	19.8725
28	8.23102	19.6088
28	8.87588	19.6437
28	7.48087	20.6465
28	7.68051	19.6292
28	8.15751	20.2495
28	7.62699	19.8774
28	8.45332	19.8099
28	7.38328	20.8016
28	8.33209	20.7549
28	8.03975	20.4176
28	8.03034	19.8372
28	8.22414	19.3517
28	8.24068	19.406
28	7.79372	19.5139
28	7.28961	19.4687
28	8.32418	20.6098
28	8.39521	19.5715
28	8.1165	19.8459
28	8.89935	20.1772
28	7.90208	20.2122
28	8.16203	19.8488
29	12.147	20.6538
29	12.7924	19.7027
29	11.9746	20.0466
29	11.7001	20.7858
29	11.0486	20.0886
29	12.1394	19.3246
29	11.9173	19.7686
29	10.914	20.2679
29	10.8239	19.9315
29	11.7145	20.3659
29	11.5396	19.364
29	12.3207	20.3679
29	12.2053	20.1533
29	12.5592	20.9693

Cluster #	x-coordinates	y-coordinates
29	10.6808	20.9767
29	11.3505	19.8166
29	11.4872	19.9818
29	11.9327	19.9081
29	12.3916	19.3864
29	12.2001	20.001
29	12.4364	19.5443
29	11.8617	20.9345
29	11.4188	19.9597
29	10.9562	20.5286
29	10.8198	20.7104
30	16.0972	19.1318
30	16.4587	19.9914
30	15.6857	20.2862
30	16.387	20.2749
30	15.9266	19.0424
30	16.1083	19.8842
30	15.6227	20.6566
30	16.8035	19.7613
30	16.2368	20.1893
30	16.0796	19.8109
30	15.957	19.905
30	15.7336	18.8686
30	15.3313	19.3653
30	16.4556	20.0383
30	15.8315	20.6735
30	16.3912	20.1831
30	15.1352	20.1974
30	15.6522	20.3308
30	15.7193	20.4953
30	16.4407	19.3174
30	16.0567	19.3669
30	15.3213	21.0848
30	16.1046	19.4916
30	16.0715	20.4651
30	15.3915	19.7442
31	0.632645	24.6059
31	0.987001	24.6592
31	-0.548389	23.8945
31	1.1931	23.1801
31	-0.118482	23.614
31	0.391596	24.3307
31	-0.395312	23.3707
31	-1.00256	23.7477
31	-0.674337	23.7711

Cluster #	x-coordinates	y-coordinates
31	-0.0507031	24.7796
31	-0.822762	23.3377
31	0.655339	24.0782
31	-0.224241	23.8877
31	0.638164	23.5072
31	0.343328	24.4249
31	0.143778	25.0502
31	0.187998	24.5791
31	-0.321969	23.2713
31	0.439318	24.7903
31	-0.413292	24.0148
31	-0.826991	23.7531
31	0.317507	23.6407
31	0.170693	24.5217
31	-0.219188	24.2209
31	-0.0351043	24.5611
32	4.56953	23.842
32	2.4542	24.6702
32	4.41148	23.9149
32	3.94636	23.6151
32	3.89253	23.1334
32	4.98217	24.0509
32	3.89703	23.3395
32	2.76909	23.7198
32	3.84132	24.242
32	4.15609	23.9824
32	3.87784	24.0803
32	4.3405	24.3581
32	3.31848	24.1868
32	4.29557	24.3421
32	4.10709	23.9015
32	3.31774	23.8857
32	4.2792	24.1847
32	4.18055	23.4539
32	4.15495	23.7341
32	3.81058	24.0681
32	4.72811	24.8196
32	4.53113	24.5886
32	4.44041	23.0642
32	3.31421	24.7078
32	3.89635	23.9944
33	8.19764	24.5927
33	8.15413	23.9694
33	9.11676	24.6299
33	7.7256	23.8196

Cluster #	x-coordinates	y-coordinates
33	8.29475	23.2826
33	7.56859	23.592
33	8.25972	23.9265
33	8.18616	23.4621
33	7.71636	23.7073
33	7.94222	23.778
33	7.80867	23.8749
33	8.03619	24.1546
33	8.61701	24.3289
33	7.86023	23.9833
33	7.81557	24.0081
33	7.76777	23.8834
33	7.97503	24.8707
33	7.49282	23.9347
33	8.201	24.2291
33	7.10059	23.5752
33	7.65168	23.4849
33	8.07216	23.6492
33	7.73572	24.6697
33	8.1743	24.7952
33	7.30211	24.5373
34	12.5739	24.5352
34	11.5972	24.0579
34	12.1961	24.7839
34	12.3023	23.703
34	11.5504	23.3796
34	11.7932	23.9614
34	11.3786	23.7851
34	11.9515	22.8846
34	11.2132	24.039
34	12.4769	23.9134
34	11.367	23.2548
34	11.2968	23.7458
34	12.0166	23.7949
34	11.4797	24.0284
34	11.5733	22.9588
34	12.2114	23.7877
34	11.7819	23.9277
34	12.8633	23.7395
34	12.1521	24.0401
34	12.6834	24.1532
34	11.7757	24.37
34	13.1988	23.5766
34	11.2344	24.2361
34	12.1776	24.9023

Cluster #	x-coordinates	y-coordinates
34	12.379	23.7894
35	16.1481	23.6404
35	16.3475	24.0362
35	15.2496	23.7193
35	15.4955	24.1802
35	16.4945	24.323
35	16.0112	24.2164
35	16.4327	24.4311
35	16.2011	23.6161
35	16.2118	24.2003
35	15.7124	24.1927
35	14.6601	24.0565
35	16.1088	23.7876
35	16.0867	23.4621
35	15.0628	23.3625
35	16.0806	24.1304
35	14.8851	24.1961
35	15.532	23.1019
35	16.2474	23.7968
35	16.3857	23.6005
35	14.9145	23.9723
35	16.3526	24.6161
35	16.3442	23.7276
35	15.7363	25.237
35	15.8688	24.5774
35	15.5192	23.7585
36	-0.348015	28.3976
36	0.230346	28.2647
36	0.51041	27.621
36	-0.352239	27.9669
36	0.825421	28.0482
36	-0.244419	28.2842
36	-0.392117	27.6426
36	0.325533	28.0905
36	0.260497	28.6312
36	-1.37343	28.4405
36	0.285071	27.9223
36	1.10118	26.9938
36	1.03217	28.1225
36	-0.199316	28.7185
36	-0.503333	27.3265
36	0.682658	27.6353
36	0.899104	28.1693
36	-1.20372	27.8749
36	0.147599	28.1602

Cluster #	x-coordinates	y-coordinates
36	-0.189986	28.0279
36	-0.118285	27.6294
36	-0.0201428	27.4499
36	-0.234716	27.7466
36	-0.127178	27.8006
36	-0.957088	27.5627
37	3.85155	27.4995
37	3.81859	28.1015
37	3.64062	29.0399
37	2.88057	28.8279
37	3.9127	28.1471
37	4.99278	27.4635
37	4.37683	28.3377
37	3.40596	27.7312
37	3.30507	28.4145
37	3.68553	27.4866
37	3.95025	28.3694
37	3.14292	28.346
37	3.8983	28.3304
37	4.26256	28.3765
37	3.95048	28.6234
37	4.4557	27.7404
37	3.48363	27.9027
37	4.18162	28.4086
37	3.81135	29.0128
37	3.94778	27.8359
37	4.05286	28.0764
37	3.42484	28.4452
37	4.66501	27.6999
37	4.59853	28.7397
37	4.42849	28.5301
38	7.81977	27.7879
38	8.0194	28.4166
38	8.08782	27.9418
38	8.5999	28.1806
38	8.20462	28.1052
38	8.70695	27.5476
38	8.39698	28.569
38	8.17137	28.6996
38	7.57162	29.0003
38	7.33094	28.3871
38	7.8229	28.8402
38	8.38685	28.0031
38	7.71822	27.9888
38	7.55257	27.588

Cluster #	x-coordinates	y-coordinates
38	7.56087	28.9163
38	7.43572	28.3359
38	7.93693	26.9331
38	8.2493	27.9509
38	8.83001	28.4076
38	8.22416	27.3397
38	8.49665	28.1426
38	8.34326	27.3794
38	8.85307	27.9851
38	8.26093	28.0645
38	9.03589	27.9854
39	11.6988	28.8858
39	11.508	27.7086
39	10.767	28.1624
39	11.9671	28.2031
39	12.9797	28.0338
39	11.3463	28.82
39	11.9785	27.529
39	12.8503	27.5156
39	12.5433	27.6647
39	12.5806	27.6187
39	11.3901	27.9296
39	11.8066	28.5997
39	11.4991	27.0477
39	11.6133	28.2131
39	11.421	28.2042
39	12.837	28.2114
39	11.1536	27.7965
39	12.3721	28.3067
39	12.341	27.7555
39	12.3929	27.8647
39	12.2111	27.8103
39	11.3878	27.401
39	12.1495	28.4337
39	12.525	28.3996
39	11.6448	29.151
40	16.5083	27.7588
40	15.7535	28.6723
40	15.9682	28.2131
40	16.5293	27.1849
40	16.3317	27.9065
40	15.7716	26.9676
40	15.8171	28.1413
40	16.5308	27.6448
40	16.6987	27.2523

Cluster #	x-coordinates	y-coordinates
40	15.4627	28.022
40	15.3211	28.6208
40	15.802	28.6367
40	15.6995	28.3541
40	15.7387	27.3023
40	16.2085	27.1268
40	15.5551	28.9068
40	16.7572	28.4343
40	16.385	28.5382
40	17.2927	28.2474
40	16.1385	28.1618
40	16.3141	27.9282
40	15.894	27.0093
40	15.8949	29.0202
40	16.3056	27.7856
40	15.853	28.3272

Appendix O

Iris
Number of dimensions: 4
Number of clusters: 3
Number of points: 150

Cluster	Sepal length	Sepal width	Petal length	Petal width
Iris setosa	5.1	3.5	1.4	0.2
Iris setosa	4.9	3	1.4	0.2
Iris setosa	4.7	3.2	1.3	0.2
Iris setosa	4.6	3.1	1.5	0.2
Iris setosa	5	3.6	1.4	0.2
Iris setosa	5.4	3.9	1.7	0.4
Iris setosa	4.6	3.4	1.4	0.3
Iris setosa	5	3.4	1.5	0.2
Iris setosa	4.4	2.9	1.4	0.2
Iris setosa	4.9	3.1	1.5	0.1
Iris setosa	5.4	3.7	1.5	0.2
Iris setosa	4.8	3.4	1.6	0.2
Iris setosa	4.8	3	1.4	0.1
Iris setosa	4.3	3	1.1	0.1
Iris setosa	5.8	4	1.2	0.2
Iris setosa	5.7	4.4	1.5	0.4
Iris setosa	5.4	3.9	1.3	0.4
Iris setosa	5.1	3.5	1.4	0.3
Iris setosa	5.7	3.8	1.7	0.3
Iris setosa	5.1	3.8	1.5	0.3
Iris setosa	5.4	3.4	1.7	0.2
Iris setosa	5.1	3.7	1.5	0.4
Iris setosa	4.6	3.6	1	0.2
Iris setosa	5.1	3.3	1.7	0.5
Iris setosa	4.8	3.4	1.9	0.2
Iris setosa	5	3	1.6	0.2
Iris setosa	5	3.4	1.6	0.4
Iris setosa	5.2	3.5	1.5	0.2
Iris setosa	5.2	3.4	1.4	0.2
Iris setosa	4.7	3.2	1.6	0.2
Iris setosa	4.8	3.1	1.6	0.2
Iris setosa	5.4	3.4	1.5	0.4
Iris setosa	5.2	4.1	1.5	0.1
Iris setosa	5.5	4.2	1.4	0.2
Iris setosa	4.9	3.1	1.5	0.1
Iris setosa	5	3.2	1.2	0.2
Iris setosa	5.5	3.5	1.3	0.2

Cluster	Sepal length	Sepal width	Petal length	Petal width
Iris setosa	4.9	3.1	1.5	0.1
Iris setosa	4.4	3	1.3	0.2
Iris setosa	5.1	3.4	1.5	0.2
Iris setosa	5	3.5	1.3	0.3
Iris setosa	4.5	2.3	1.3	0.3
Iris setosa	4.4	3.2	1.3	0.2
Iris setosa	5	3.5	1.6	0.6
Iris setosa	5.1	3.8	1.9	0.4
Iris setosa	4.8	3	1.4	0.3
Iris setosa	5.1	3.8	1.6	0.2
Iris setosa	4.6	3.2	1.4	0.2
Iris setosa	5.3	3.7	1.5	0.2
Iris setosa	5	3.3	1.4	0.2
Iris versicolor	7	3.2	4.7	1.4
Iris versicolor	6.4	3.2	4.5	1.5
Iris versicolor	6.9	3.1	4.9	1.5
Iris versicolor	5.5	2.3	4	1.3
Iris versicolor	6.5	2.8	4.6	1.5
Iris versicolor	5.7	2.8	4.5	1.3
Iris versicolor	6.3	3.3	4.7	1.6
Iris versicolor	4.9	2.4	3.3	1
Iris versicolor	6.6	2.9	4.6	1.3
Iris versicolor	5.2	2.7	3.9	1.4
Iris versicolor	5	2	3.5	1
Iris versicolor	5.9	3	4.2	1.5
Iris versicolor	6	2.2	4	1
Iris versicolor	6.1	2.9	4.7	1.4
Iris versicolor	5.6	2.9	3.6	1.3
Iris versicolor	6.7	3.1	4.4	1.4
Iris versicolor	5.6	3	4.5	1.5
Iris versicolor	5.8	2.7	4.1	1
Iris versicolor	6.2	2.2	4.5	1.5
Iris versicolor	5.6	2.5	3.9	1.1
Iris versicolor	5.9	3.2	4.8	1.8
Iris versicolor	6.1	2.8	4	1.3
Iris versicolor	6.3	2.5	4.9	1.5
Iris versicolor	6.1	2.8	4.7	1.2
Iris versicolor	6.4	2.9	4.3	1.3
Iris versicolor	6.6	3	4.4	1.4
Iris versicolor	6.8	2.8	4.8	1.4
Iris versicolor	6.7	3	5	1.7
Iris versicolor	6	2.9	4.5	1.5
Iris versicolor	5.7	2.6	3.5	1

Cluster	Sepal length	Sepal width	Petal length	Petal width
Iris versicolor	5.5	2.4	3.8	1.1
Iris versicolor	5.5	2.4	3.7	1
Iris versicolor	5.8	2.7	3.9	1.2
Iris versicolor	6	2.7	5.1	1.6
Iris versicolor	5.4	3	4.5	1.5
Iris versicolor	6	3.4	4.5	1.6
Iris versicolor	6.7	3.1	4.7	1.5
Iris versicolor	6.3	2.3	4.4	1.3
Iris versicolor	5.6	3	4.1	1.3
Iris versicolor	5.5	2.5	4	1.3
Iris versicolor	5.5	2.6	4.4	1.2
Iris versicolor	6.1	3	4.6	1.4
Iris versicolor	5.8	2.6	4	1.2
Iris versicolor	5	2.3	3.3	1
Iris versicolor	5.6	2.7	4.2	1.3
Iris versicolor	5.7	3	4.2	1.2
Iris versicolor	5.7	2.9	4.2	1.3
Iris versicolor	6.2	2.9	4.3	1.3
Iris versicolor	5.1	2.5	3	1.1
Iris versicolor	5.7	2.8	4.1	1.3
Iris virginica	6.3	3.3	6	2.5
Iris virginica	5.8	2.7	5.1	1.9
Iris virginica	7.1	3	5.9	2.1
Iris virginica	6.3	2.9	5.6	1.8
Iris virginica	6.5	3	5.8	2.2
Iris virginica	7.6	3	6.6	2.1
Iris virginica	4.9	2.5	4.5	1.7
Iris virginica	7.3	2.9	6.3	1.8
Iris virginica	6.7	2.5	5.8	1.8
Iris virginica	7.2	3.6	6.1	2.5
Iris virginica	6.5	3.2	5.1	2
Iris virginica	6.4	2.7	5.3	1.9
Iris virginica	6.8	3	5.5	2.1
Iris virginica	5.7	2.5	5	2
Iris virginica	5.8	2.8	5.1	2.4
Iris virginica	6.4	3.2	5.3	2.3
Iris virginica	6.5	3	5.5	1.8
Iris virginica	7.7	3.8	6.7	2.2
Iris virginica	7.7	2.6	6.9	2.3
Iris virginica	6	2.2	5	1.5
Iris virginica	6.9	3.2	5.7	2.3
Iris virginica	5.6	2.8	4.9	2
Iris virginica	7.7	2.8	6.7	2

Cluster	Sepal length	Sepal width	Petal length	Petal width
Iris virginica	6.3	2.7	4.9	1.8
Iris virginica	6.7	3.3	5.7	2.1
Iris virginica	7.2	3.2	6	1.8
Iris virginica	6.2	2.8	4.8	1.8
Iris virginica	6.1	3	4.9	1.8
Iris virginica	6.4	2.8	5.6	2.1
Iris virginica	7.2	3	5.8	1.6
Iris virginica	7.4	2.8	6.1	1.9
Iris virginica	7.9	3.8	6.4	2
Iris virginica	6.4	2.8	5.6	2.2
Iris virginica	6.3	2.8	5.1	1.5
Iris virginica	6.1	2.6	5.6	1.4
Iris virginica	7.7	3	6.1	2.3
Iris virginica	6.3	3.4	5.6	2.4
Iris virginica	6.4	3.1	5.5	1.8
Iris virginica	6	3	4.8	1.8
Iris virginica	6.9	3.1	5.4	2.1
Iris virginica	6.7	3.1	5.6	2.4
Iris virginica	6.9	3.1	5.1	2.3
Iris virginica	5.8	2.7	5.1	1.9
Iris virginica	6.8	3.2	5.9	2.3
Iris virginica	6.7	3.3	5.7	2.5
Iris virginica	6.7	3	5.2	2.3
Iris virginica	6.3	2.5	5	1.9
Iris virginica	6.5	3	5.2	2
Iris virginica	6.2	3.4	5.4	2.3
Iris virginica	5.9	3	5.1	1.8

Appendix P

Breast Cancer Wisconsin
Number of dimensions: 9
Number of clusters: 2
Number of points: 683
Tumor is benign (1) os malignant (2)

Cluster	ClumpThickness	CellSize	CellShape	MarginalAdhesion	EpithelialSize	BareNuclei	BlandChromatin	NormalNucleoli	Mitoses
1	5	1	1	1	2	1	3	1	1
1	5	4	4	5	7	10	3	2	1
1	3	1	1	1	2	2	3	1	1
1	6	8	8	1	3	4	3	7	1
1	4	1	1	3	2	1	3	1	1
2	8	10	10	8	7	10	9	7	1
1	1	1	1	1	2	10	3	1	1
1	2	1	2	1	2	1	3	1	1
1	2	1	1	1	2	1	1	1	5
1	4	2	1	1	2	1	2	1	1
1	1	1	1	1	1	1	3	1	1
1	2	1	1	1	2	1	2	1	1
2	5	3	3	3	2	3	4	4	1
2	8	7	5	10	7	9	5	5	4
2	7	4	6	4	6	1	4	3	1
1	4	1	1	1	2	1	2	1	1
1	4	1	1	1	2	1	3	1	1
2	10	7	7	6	4	10	4	1	2
1	6	1	1	1	2	1	3	1	1
2	7	3	2	10	5	10	5	4	4
2	10	5	5	3	6	7	7	10	1
1	3	1	1	1	2	1	2	1	1
1	1	1	1	1	2	1	3	1	1
2	5	2	3	4	2	7	3	6	1
1	3	2	1	1	1	1	2	1	1

Cluster	ClumpThickness	CellSize	CellShape	MarginalAdhesion	EpithelialSize	BareNuclei	BlandChromatin	NormalNucleoli	Mitoses
1	5	1	1	1	2	1	2	1	1
1	2	1	1	1	2	1	2	1	1
1	1	1	3	1	2	1	1	1	1
1	3	1	1	1	1	1	2	1	1
1	2	1	1	1	2	1	3	1	1
2	10	7	7	3	8	5	7	4	3
1	2	1	1	2	2	1	3	1	1
1	3	1	2	1	2	1	2	1	1
2	10	10	10	8	6	1	8	9	1
1	6	2	1	1	1	1	7	1	1
2	5	4	4	9	2	10	5	6	1
2	2	5	3	3	6	7	7	5	1
2	10	4	3	1	3	3	6	5	2
2	6	10	10	2	8	10	7	3	3
2	10	10	10	4	8	1	8	10	1
1	1	1	1	1	2	1	2	1	2
2	3	7	7	4	4	9	4	8	1
1	1	1	1	1	2	1	2	1	1
1	4	1	1	3	2	1	3	1	1
2	7	8	7	2	4	8	3	8	2
2	9	5	8	1	2	3	2	1	5
2	5	3	3	4	2	4	3	4	1
2	10	3	6	2	3	5	4	10	2
2	5	5	5	8	10	8	7	3	7
2	10	5	5	6	8	8	7	1	1
2	10	6	6	3	4	5	3	6	1
2	8	10	10	1	3	6	3	9	1
2	8	2	4	1	5	1	5	4	4
2	5	2	3	1	6	10	5	1	1
2	9	5	5	2	2	2	5	1	1
2	5	3	5	5	3	3	4	10	1
2	9	10	10	1	10	8	3	3	1

Cluster	ClumpThickness	CellSize	CellShape	MarginalAdhesion	EpithelialSize	BareNuclei	BlandChromatin	NormalNucleoli	Mitoses
2	6	3	4	1	5	2	3	9	1
1	1	1	1	1	2	1	2	1	1
2	10	4	2	1	3	2	4	3	10
1	4	1	1	1	2	1	3	1	1
2	5	3	4	1	8	10	4	9	1
2	8	3	8	3	4	9	8	9	8
1	1	1	1	1	2	1	3	2	1
1	5	1	3	1	2	1	2	1	1
2	6	10	2	8	10	2	7	8	10
1	1	3	3	2	2	1	7	2	1
2	10	6	4	1	3	4	3	2	3
1	1	1	2	1	2	2	4	2	1
1	1	1	4	1	2	1	2	1	1
1	5	3	1	2	2	1	2	1	1
1	3	1	1	1	2	3	3	1	1
1	2	1	1	1	3	1	2	1	1
1	2	2	2	1	1	1	7	1	1
1	5	2	1	1	2	1	3	1	1
1	3	1	1	1	2	2	7	1	1
2	3	5	7	8	8	9	7	10	7
2	5	10	6	1	10	4	4	10	10
2	3	3	6	4	5	8	4	4	1
2	3	6	6	6	5	10	6	8	3
1	4	1	1	1	2	1	3	1	1
1	2	1	1	2	3	1	2	1	1
1	1	1	1	1	2	1	3	1	1
1	3	1	1	2	2	1	1	1	1
1	4	1	1	1	2	1	3	1	1
1	1	1	1	1	2	1	2	1	1
1	2	1	1	1	2	1	3	1	1
1	1	1	1	1	2	1	3	1	1
1	2	1	1	2	2	1	1	1	1

Cluster	ClumpThickness	CellSize	CellShape	MarginalAdhesion	EpithelialSize	BareNuclei	BlandChromatin	NormalNucleoli	Mitoses
1	5	1	1	1	2	1	3	1	1
2	9	6	9	2	10	6	2	9	10
2	7	5	6	10	5	10	7	9	4
2	10	3	5	1	10	5	3	10	2
2	2	3	4	4	2	5	2	5	1
1	4	1	2	1	2	1	3	1	1
2	8	2	3	1	6	3	7	1	1
2	10	10	10	8	2	10	4	1	1
2	1	6	8	10	8	10	5	7	1
1	1	1	1	1	2	1	2	3	1
2	6	5	4	4	3	9	7	8	3
1	1	3	1	2	2	2	5	3	2
2	8	6	4	3	5	9	3	1	1
2	10	3	3	10	2	10	7	3	3
2	10	10	10	3	10	8	8	1	1
1	3	3	2	1	2	3	3	1	1
1	1	1	1	1	2	5	1	1	1
1	8	3	3	1	2	2	3	2	1
2	4	5	5	10	4	10	7	5	8
1	1	1	1	1	4	3	1	1	1
1	3	2	1	1	2	2	3	1	1
1	1	1	2	2	2	1	3	1	1
1	4	2	1	1	2	2	3	1	1
2	10	10	10	2	10	10	5	3	3
2	5	4	6	7	9	7	8	10	1
1	1	1	1	1	2	1	2	1	1
2	7	5	3	7	4	10	7	5	5
1	3	1	1	1	2	1	3	1	1
2	8	3	5	4	5	10	1	6	2
1	1	1	1	1	10	1	1	1	1
1	5	1	3	1	2	1	2	1	1
1	2	1	1	1	2	1	3	1	1

Cluster	ClumpThickness	CellSize	CellShape	MarginalAdhesion	EpithelialSize	BareNuclei	BlandChromatin	NormalNucleoli	Mitoses
2	5	10	8	10	8	10	3	6	3
1	3	1	1	1	2	1	2	2	1
1	3	1	1	1	3	1	2	1	1
1	5	1	1	1	2	2	3	3	1
1	3	1	1	1	2	1	1	1	1
1	4	1	2	1	2	1	2	1	1
1	3	1	1	1	2	1	1	1	1
1	2	1	1	1	2	1	1	1	1
2	9	5	5	4	4	5	4	3	3
1	2	1	1	1	2	1	2	1	1
2	3	4	5	2	6	8	4	1	1
1	1	1	1	1	3	2	2	1	1
2	8	8	7	4	10	10	7	8	7
1	1	1	1	1	1	1	3	1	1
2	7	2	4	1	6	10	5	4	3
2	10	10	8	6	4	5	8	10	1
1	4	1	1	1	2	3	1	1	1
1	1	1	1	1	2	1	1	1	1
2	5	5	5	6	3	10	3	1	1
1	1	2	2	1	2	1	2	1	1
1	2	1	1	1	2	1	3	1	1
2	9	9	10	3	6	10	7	10	6
2	10	7	7	4	5	10	5	7	2
1	4	1	1	1	2	1	3	2	1
1	3	1	1	1	2	1	3	1	1
1	1	1	1	2	1	3	1	1	7
1	4	1	1	1	2	2	3	2	1
2	5	6	7	8	8	10	3	10	3
1	3	1	1	1	2	1	3	1	1
1	1	1	1	2	1	1	1	1	1
1	3	1	1	1	2	1	1	1	1
1	1	1	1	1	2	1	3	1	1

Cluster	ClumpThickness	CellSize	CellShape	MarginalAdhesion	EpithelialSize	BareNuclei	BlandChromatin	NormalNucleoli	Mitoses
1	1	1	1	1	2	1	2	1	1
2	6	10	10	10	8	10	10	10	7
2	8	6	5	4	3	10	6	1	1
2	5	8	7	7	10	10	5	7	1
1	2	1	1	1	2	1	3	1	1
2	5	10	10	3	8	1	5	10	3
1	4	1	1	1	2	1	3	1	1
2	5	3	3	3	6	10	3	1	1
1	1	1	1	1	1	1	3	1	1
1	1	1	1	1	2	1	1	1	1
1	6	1	1	1	2	1	3	1	1
2	5	8	8	8	5	10	7	8	1
2	1	5	8	6	5	8	7	10	1
2	10	5	6	10	6	10	7	7	10
2	5	8	4	10	5	8	9	10	1
1	1	2	3	1	2	1	3	1	1
2	10	10	10	8	6	8	7	10	1
2	7	5	10	10	10	10	4	10	3
1	5	1	1	1	2	1	2	1	1
1	1	1	1	1	2	1	3	1	1
1	3	1	1	1	2	1	3	1	1
1	4	1	1	1	2	1	3	1	1
1	5	1	1	4	2	1	3	1	1
1	3	1	1	1	2	1	2	1	1
2	9	7	7	5	5	10	7	8	3
2	10	8	8	4	10	10	8	1	1
1	1	1	1	1	2	1	3	1	1
1	5	1	1	1	2	1	3	1	1
1	1	1	1	1	2	1	3	1	1
2	5	10	10	9	6	10	7	10	5
2	10	10	9	3	7	5	3	5	1
1	1	1	1	1	1	1	3	1	1

Cluster	ClumpThickness	CellSize	CellShape	MarginalAdhesion	EpithelialSize	BareNuclei	BlandChromatin	NormalNucleoli	Mitoses
1	5	1	1	1	1	1	3	1	1
2	8	10	10	10	5	10	8	10	6
2	8	10	8	8	4	8	7	7	1
1	1	1	1	1	2	1	3	1	1
2	10	10	10	10	7	10	7	10	4
2	10	10	10	10	3	10	10	6	1
2	8	7	8	7	5	5	5	10	2
1	1	1	1	1	2	1	2	1	1
1	1	1	1	1	2	1	3	1	1
2	6	10	7	7	6	4	8	10	2
1	6	1	3	1	2	1	3	1	1
1	1	1	1	2	2	1	3	1	1
2	10	6	4	3	10	10	9	10	1
2	4	1	1	3	1	5	2	1	1
2	7	5	6	3	3	8	7	4	1
2	10	5	5	6	3	10	7	9	2
1	1	1	1	1	2	1	2	1	1
2	10	5	7	4	4	10	8	9	1
2	8	9	9	5	3	5	7	7	1
1	1	1	1	1	1	1	3	1	1
2	7	4	7	4	3	7	7	6	1
2	6	8	7	5	6	8	8	9	2
1	8	4	6	3	3	1	4	3	1
2	10	4	5	5	5	10	4	1	1
1	3	3	2	1	3	1	3	6	1
2	10	8	8	2	8	10	4	8	10
2	9	8	8	5	6	2	4	10	4
2	8	10	10	8	6	9	3	10	10
2	10	4	3	2	3	10	5	3	2
1	5	1	3	3	2	2	2	3	1
1	3	1	1	3	1	1	3	1	1
1	2	1	1	1	2	1	3	1	1

Cluster	ClumpThickness	CellSize	CellShape	MarginalAdhesion	EpithelialSize	BareNuclei	BlandChromatin	NormalNucleoli	Mitoses
1	1	1	1	1	2	5	5	1	1
1	1	1	1	1	2	1	3	1	1
1	5	1	1	2	2	2	3	1	1
2	8	10	10	8	5	10	7	8	1
2	8	4	4	1	2	9	3	3	1
1	4	1	1	1	2	1	3	6	1
1	1	2	2	1	2	1	1	1	1
2	10	4	4	10	2	10	5	3	3
1	6	3	3	5	3	10	3	5	3
2	6	10	10	2	8	10	7	3	3
2	5	6	6	2	4	10	3	6	1
1	3	1	1	1	2	1	1	1	1
1	3	1	1	1	2	1	3	1	1
1	5	7	7	1	5	8	3	4	1
2	10	5	8	10	3	10	5	1	3
2	5	10	10	6	10	10	10	6	5
2	10	4	4	10	6	10	5	5	1
2	7	9	4	10	10	3	5	3	3
1	5	1	4	1	2	1	3	2	1
2	10	10	6	3	3	10	4	3	2
2	3	3	5	2	3	10	7	1	1
2	10	8	8	2	3	4	8	7	8
1	1	1	1	1	2	1	3	1	1
2	8	4	7	1	3	10	3	9	2
2	3	3	5	2	3	10	7	1	1
2	7	2	4	1	3	4	3	3	1
1	3	1	1	1	2	1	3	2	1
1	3	1	1	1	2	1	2	1	1
1	1	1	1	1	2	1	2	1	1
2	10	5	7	3	3	7	3	3	8
1	3	1	1	1	2	1	3	1	1
1	2	1	1	2	2	1	3	1	1

Cluster	ClumpThickness	CellSize	CellShape	MarginalAdhesion	EpithelialSize	BareNuclei	BlandChromatin	NormalNucleoli	Mitoses
2	1	4	3	10	4	10	5	6	1
2	10	4	6	1	2	10	5	3	1
2	7	4	5	10	2	10	3	8	2
2	8	10	10	10	8	10	10	7	3
2	10	10	10	10	10	10	4	10	10
1	3	1	1	1	3	1	2	1	1
2	6	1	3	1	4	5	5	10	1
2	5	6	6	8	6	10	4	10	4
1	1	1	1	1	2	1	1	1	1
1	1	1	1	1	2	1	3	1	1
2	10	4	4	6	2	10	2	3	1
2	5	5	7	8	6	10	7	4	1
1	5	3	4	3	4	5	4	7	1
1	8	2	1	1	5	1	1	1	1
2	9	1	2	6	4	10	7	7	2
1	1	1	1	1	2	1	3	1	1
1	1	1	1	1	2	1	3	1	1
2	8	3	4	9	3	10	3	3	1
1	1	1	1	1	2	1	3	1	1
1	1	1	1	1	2	1	3	1	1
2	7	8	7	6	4	3	8	8	4
1	2	1	1	1	3	1	2	1	1
1	1	1	1	1	2	1	1	1	1
2	8	6	4	10	10	1	3	5	1
1	1	1	1	1	2	1	1	1	1
1	1	1	1	1	1	1	2	1	1
2	5	5	5	2	5	10	4	3	1
2	6	8	7	8	6	8	8	9	1
1	1	1	1	1	5	1	3	1	1
1	4	4	4	4	6	5	7	3	1
2	5	4	6	10	2	10	4	1	1
1	1	1	1	1	2	1	3	1	1

Cluster	ClumpThickness	CellSize	CellShape	MarginalAdhesion	EpithelialSize	BareNuclei	BlandChromatin	NormalNucleoli	Mitoses
1	3	2	2	1	2	1	2	3	1
2	10	1	1	1	2	10	5	4	1
1	1	1	1	1	2	1	2	1	1
2	8	10	3	2	6	4	3	10	1
2	10	4	7	2	2	8	6	1	1
1	5	1	1	1	2	1	3	1	2
1	5	2	2	2	2	1	2	2	1
2	5	4	6	6	4	10	4	3	1
2	8	6	7	3	3	10	3	4	2
1	1	1	1	1	2	1	1	1	1
2	6	5	5	8	4	10	3	4	1
1	1	1	1	1	2	1	3	1	1
2	10	3	3	1	2	10	7	6	1
1	1	1	1	1	2	1	3	1	1
1	1	1	1	1	2	1	1	1	1
2	7	6	4	8	10	10	9	5	3
1	1	1	1	1	2	1	1	1	1
1	5	2	2	2	3	1	1	3	1
1	1	1	1	1	1	1	1	3	1
2	3	4	4	10	5	1	3	3	1
2	4	2	3	5	3	8	7	6	1
1	5	1	1	3	2	1	1	1	1
2	2	7	10	10	7	10	4	9	4
1	1	1	1	1	2	1	2	1	1
2	5	3	3	1	3	3	3	3	3
2	8	10	10	7	10	10	7	3	8
2	8	10	5	3	8	4	4	10	3
2	6	10	10	10	10	10	8	10	10
2	3	10	3	10	6	10	5	1	4
1	3	2	2	1	4	3	2	1	1
1	4	4	4	2	2	3	2	1	1
1	2	1	1	1	2	1	3	1	1

Cluster	ClumpThickness	CellSize	CellShape	MarginalAdhesion	EpithelialSize	BareNuclei	BlandChromatin	NormalNucleoli	Mitoses
1	2	1	1	1	2	1	2	1	1
2	6	10	10	10	8	10	7	10	7
2	5	8	8	10	5	10	8	10	3
1	1	1	3	1	2	1	1	1	1
1	1	1	3	1	1	1	2	1	1
1	4	3	2	1	3	1	2	1	1
1	1	1	3	1	2	1	1	1	1
1	4	1	2	1	2	1	2	1	1
1	5	1	1	2	2	1	2	1	1
1	3	1	2	1	2	1	2	1	1
1	1	1	1	1	2	1	1	1	1
1	1	1	1	1	2	1	2	1	1
1	1	1	1	1	1	1	2	1	1
1	3	1	1	4	3	1	2	2	1
1	5	3	4	1	4	1	3	1	1
1	1	1	1	1	2	1	1	1	1
2	10	6	3	6	4	10	7	8	4
1	3	2	2	2	2	1	3	2	1
1	2	1	1	1	2	1	1	1	1
1	2	1	1	1	2	1	1	1	1
1	3	3	2	2	3	1	1	2	3
2	7	6	6	3	2	10	7	1	1
1	5	3	3	2	3	1	3	1	1
1	2	1	1	1	2	1	2	2	1
1	5	1	1	1	3	2	2	2	1
1	1	1	1	2	2	1	2	1	1
2	10	8	7	4	3	10	7	9	1
1	3	1	1	1	2	1	2	1	1
1	1	2	3	1	2	1	2	1	1
1	3	1	1	1	2	1	2	1	1
1	3	1	1	1	2	1	3	1	1
1	4	1	1	1	2	1	1	1	1

Cluster	ClumpThickness	CellSize	CellShape	MarginalAdhesion	EpithelialSize	BareNuclei	BlandChromatin	NormalNucleoli	Mitoses
1	3	2	1	1	2	1	2	2	1
1	1	2	3	1	2	1	1	1	1
2	3	10	8	7	6	9	9	3	8
1	3	1	1	1	2	1	1	1	1
1	5	3	3	1	2	1	2	1	1
1	3	1	1	1	2	4	1	1	1
1	1	2	1	3	2	1	1	2	1
1	4	2	2	1	2	1	2	1	1
1	1	1	1	1	2	1	2	1	1
1	2	3	2	2	2	2	3	1	1
1	3	1	2	1	2	1	2	1	1
1	1	1	1	1	2	1	2	1	1
2	10	10	10	6	8	4	8	5	1
1	5	1	2	1	2	1	3	1	1
2	8	5	6	2	3	10	6	6	1
1	3	3	2	6	3	3	3	5	1
2	8	7	8	5	10	10	7	2	1
1	1	1	1	1	2	1	2	1	1
1	5	2	2	2	2	2	3	2	2
1	3	2	2	3	2	3	3	1	1
2	10	10	10	7	10	10	8	2	1
1	4	3	3	1	2	1	3	3	1
1	5	1	3	1	2	1	2	1	1
1	3	1	1	1	2	1	1	1	1
2	9	10	10	10	10	10	10	10	1
1	5	3	6	1	2	1	1	1	1
2	8	7	8	2	4	2	5	10	1
1	1	1	1	1	2	1	2	1	1
1	2	1	1	1	2	1	2	1	1
1	1	3	1	1	2	1	2	2	1
1	5	1	1	3	4	1	3	2	1
1	5	1	1	1	2	1	2	2	1

Cluster	ClumpThickness	CellSize	CellShape	MarginalAdhesion	EpithelialSize	BareNuclei	BlandChromatin	NormalNucleoli	Mitoses
1	3	2	2	3	2	1	1	1	1
1	6	9	7	5	5	8	4	2	1
2	10	8	10	1	3	10	5	1	1
2	10	10	10	1	6	1	2	8	1
1	4	1	1	1	2	1	1	1	1
1	5	1	1	1	2	1	1	1	1
2	10	4	3	10	4	10	10	1	1
1	5	2	2	4	2	4	1	1	1
1	1	1	1	3	2	3	1	1	1
1	1	1	1	1	2	2	1	1	1
1	5	1	1	6	3	1	2	1	1
1	2	1	1	1	2	1	1	1	1
1	1	1	1	1	2	1	1	1	1
1	5	1	1	1	2	1	1	1	1
1	1	1	1	1	1	1	1	1	1
2	5	7	9	8	6	10	8	10	1
1	4	1	1	3	1	1	2	1	1
1	5	1	1	1	2	1	1	1	1
1	3	1	1	3	2	1	1	1	1
2	4	5	5	8	6	10	10	7	1
1	2	3	1	1	3	1	1	1	1
2	10	2	2	1	2	6	1	1	2
2	10	6	5	8	5	10	8	6	1
2	8	8	9	6	6	3	10	10	1
1	5	1	2	1	2	1	1	1	1
1	5	1	1	3	2	1	1	1	1
1	3	1	1	1	2	5	1	1	1
1	6	1	1	3	2	1	1	1	1
1	4	1	1	1	2	1	1	2	1
2	10	9	8	7	6	4	7	10	3
2	10	6	6	2	4	10	9	7	1
2	6	6	6	5	4	10	7	6	2

Cluster	ClumpThickness	CellSize	CellShape	MarginalAdhesion	EpithelialSize	BareNuclei	BlandChromatin	NormalNucleoli	Mitoses
1	4	1	1	1	2	1	1	1	1
1	1	1	2	1	2	1	2	1	1
1	3	1	1	1	1	1	2	1	1
1	6	1	1	3	2	1	1	1	1
1	6	1	1	1	1	1	1	1	1
1	4	1	1	1	2	1	1	1	1
1	3	1	1	1	2	1	1	1	1
1	4	1	2	1	2	1	1	1	1
1	4	1	1	1	2	1	1	1	1
1	5	2	1	1	2	1	1	1	1
2	4	8	7	10	4	10	7	5	1
1	5	1	1	1	1	1	1	1	1
1	5	3	2	4	2	1	1	1	1
2	9	10	10	10	10	5	10	10	10
2	8	7	8	5	5	10	9	10	1
1	5	1	2	1	2	1	1	1	1
1	1	1	1	3	1	3	1	1	1
1	3	1	1	1	1	1	2	1	1
2	10	10	10	10	6	10	8	1	5
2	3	6	4	10	3	3	3	4	1
2	6	3	2	1	3	4	4	1	1
2	5	8	9	4	3	10	7	1	1
1	4	1	1	1	1	1	2	1	1
2	5	10	10	10	6	10	6	5	2
1	5	1	2	10	4	5	2	1	1
1	3	1	1	1	1	1	2	1	1
1	1	1	1	1	1	1	1	1	1
1	4	2	1	1	2	1	1	1	1
1	4	1	1	1	2	1	2	1	1
1	6	1	1	1	2	1	3	1	1
1	4	1	1	1	2	1	2	1	1
1	4	1	1	1	2	1	3	1	1

Cluster	ClumpThickness	CellSize	CellShape	MarginalAdhesion	EpithelialSize	BareNuclei	BlandChromatin	NormalNucleoli	Mitoses
1	1	1	1	1	2	1	1	1	1
1	3	3	1	1	2	1	1	1	1
2	8	10	10	10	7	5	4	8	7
1	1	1	1	1	2	4	1	1	1
1	5	1	1	1	2	1	1	1	1
1	2	1	1	1	2	1	1	1	1
1	1	1	1	1	2	1	1	1	1
1	5	1	1	1	2	1	2	1	1
1	5	1	1	1	2	1	1	1	1
1	3	1	1	1	1	1	2	1	1
2	6	6	7	10	3	10	8	10	2
2	4	10	4	7	3	10	9	10	1
1	1	1	1	1	1	1	1	1	1
1	1	1	1	1	1	1	2	1	1
1	3	1	2	2	2	1	1	1	1
2	4	7	8	3	4	10	9	1	1
1	1	1	1	1	3	1	1	1	1
1	4	1	1	1	3	1	1	1	1
2	7	5	6	10	4	10	5	3	1
1	3	1	1	1	2	1	2	1	1
1	3	1	1	2	2	1	1	1	1
1	4	1	1	1	2	1	1	1	1
1	4	1	1	1	2	1	3	1	1
1	6	1	3	2	2	1	1	1	1
1	4	1	1	1	1	1	2	1	1
2	7	4	4	3	4	10	6	9	1
1	4	2	2	1	2	1	2	1	1
1	1	1	1	1	1	1	3	1	1
1	3	1	1	1	2	1	2	1	1
1	2	1	1	1	2	1	2	1	1
1	1	1	3	2	2	1	3	1	1
1	5	1	1	1	2	1	3	1	1

Cluster	ClumpThickness	CellSize	CellShape	MarginalAdhesion	EpithelialSize	BareNuclei	BlandChromatin	NormalNucleoli	Mitoses
1	5	1	2	1	2	1	3	1	1
1	4	1	1	1	2	1	2	1	1
1	6	1	1	1	2	1	2	1	1
1	5	1	1	1	2	2	2	1	1
1	3	1	1	1	2	1	1	1	1
1	5	3	1	1	2	1	1	1	1
1	4	1	1	1	2	1	2	1	1
1	2	1	3	2	2	1	2	1	1
1	5	1	1	1	2	1	2	1	1
2	6	10	10	10	4	10	7	10	1
1	2	1	1	1	1	1	1	1	1
1	3	1	1	1	1	1	1	1	1
2	7	8	3	7	4	5	7	8	2
1	3	1	1	1	2	1	2	1	1
1	1	1	1	1	2	1	3	1	1
1	3	2	2	2	2	1	4	2	1
1	4	4	2	1	2	5	2	1	2
1	3	1	1	1	2	1	1	1	1
1	5	2	2	2	1	1	2	1	1
1	5	1	1	3	2	1	1	1	1
1	2	1	1	1	2	1	2	1	1
1	5	1	1	1	2	1	2	1	1
1	5	1	1	1	2	1	3	1	1
1	5	1	1	1	2	1	3	1	1
1	1	1	1	1	2	1	3	1	1
1	3	1	1	1	2	1	2	1	1
1	4	1	1	1	2	1	3	2	1
2	5	7	10	10	5	10	10	10	1
1	3	1	2	1	2	1	3	1	1
1	4	1	1	1	2	3	2	1	1
2	8	4	4	1	6	10	2	5	2
2	8	10	4	4	8	10	8	2	1

Cluster	ClumpThickness	CellSize	CellShape	MarginalAdhesion	EpithelialSize	BareNuclei	BlandChromatin	NormalNucleoli	Mitoses
1	3	1	1	1	2	1	2	1	1
1	1	1	1	1	2	1	2	1	1
2	10	9	7	3	4	2	7	7	1
1	5	1	2	1	2	1	3	1	1
1	5	1	1	1	2	1	2	1	1
1	1	1	1	1	2	1	2	1	1
1	1	1	1	1	2	1	3	1	1
2	5	7	10	6	5	10	7	5	1
2	6	10	5	5	4	10	6	10	1
1	3	1	1	1	2	1	1	1	1
1	5	1	1	6	3	1	1	1	1
1	1	1	1	1	2	1	1	1	1
2	8	10	10	10	6	10	10	10	1
1	5	1	1	1	2	1	2	2	1
2	9	8	8	9	6	3	4	1	1
1	5	1	1	1	2	1	1	1	1
2	4	10	8	5	4	1	10	1	1
2	2	5	7	6	4	10	7	6	1
2	10	3	4	5	3	10	4	1	1
1	5	1	2	1	2	1	1	1	1
2	4	8	6	3	4	10	7	1	1
1	5	1	1	1	2	1	2	1	1
1	4	1	2	1	2	1	2	1	1
1	5	1	3	1	2	1	3	1	1
1	3	1	1	1	2	1	2	1	1
1	5	2	4	1	1	1	1	1	1
1	3	1	1	1	2	1	2	1	1
1	1	1	1	1	1	1	2	1	1
1	4	1	1	1	2	1	2	1	1
2	5	4	6	8	4	1	8	10	1
2	5	3	2	8	5	10	8	1	2
2	10	5	10	3	5	8	7	8	3

Cluster	ClumpThickness	CellSize	CellShape	MarginalAdhesion	EpithelialSize	BareNuclei	BlandChromatin	NormalNucleoli	Mitoses
1	4	1	1	2	2	1	1	1	1
2	5	10	10	10	10	10	10	1	1
1	5	1	1	1	2	1	1	1	1
2	5	10	10	10	5	2	8	5	1
2	8	10	10	10	6	10	10	10	10
1	2	3	1	1	2	1	2	1	1
1	2	1	1	1	1	1	2	1	1
1	4	1	3	1	2	1	2	1	1
1	3	1	1	1	2	1	2	1	1
1	4	1	1	1	2	1	2	1	1
1	5	1	1	1	2	1	2	1	1
1	3	1	1	1	2	1	2	1	1
1	6	3	3	3	3	2	6	1	1
1	7	1	2	3	2	1	2	1	1
1	1	1	1	1	2	1	1	1	1
1	5	1	1	2	1	1	2	1	1
1	3	1	3	1	3	4	1	1	1
2	4	6	6	5	7	6	7	7	3
1	2	1	1	1	2	5	1	1	1
1	2	1	1	1	2	1	1	1	1
1	4	1	1	1	2	1	1	1	1
1	1	1	1	1	2	1	1	1	1
2	8	7	4	4	5	3	5	10	1
1	3	1	1	1	2	1	1	1	1
2	10	10	7	8	7	1	10	10	3
1	4	2	4	3	2	2	2	1	1
1	4	1	1	1	2	1	1	1	1
1	5	1	1	3	2	1	1	1	1
1	4	1	1	3	2	1	1	1	1
1	3	1	1	1	2	1	2	1	1
1	3	1	1	1	2	1	2	1	1
1	2	1	1	1	2	1	1	1	1

Cluster	ClumpThickness	CellSize	CellShape	MarginalAdhesion	EpithelialSize	BareNuclei	BlandChromatin	NormalNucleoli	Mitoses
1	3	1	1	1	2	1	2	1	1
1	1	1	1	3	2	1	1	1	1
2	5	10	10	10	10	2	10	10	10
1	3	1	1	1	2	1	2	1	1
1	1	2	1	3	2	1	2	1	1
1	5	1	1	1	2	1	2	2	1
1	4	1	1	1	2	1	2	1	1
1	3	1	1	1	2	1	3	1	1
1	5	1	1	1	2	1	2	1	1
1	5	4	5	1	8	1	3	6	1
2	7	8	8	7	3	10	7	2	3
1	1	1	1	1	2	1	1	1	1
1	1	1	1	1	2	1	2	1	1
1	4	1	1	1	2	1	3	1	1
1	1	1	3	1	2	1	2	1	1
1	1	1	3	1	2	1	2	1	1
1	3	1	1	3	2	1	2	1	1
1	1	1	1	1	2	1	1	1	1
1	5	2	2	2	2	1	1	1	2
1	3	1	1	1	2	1	3	1	1
2	5	7	4	1	6	1	7	10	3
2	5	10	10	8	5	5	7	10	1
2	3	10	7	8	5	8	7	4	1
1	3	2	1	2	2	1	3	1	1
1	5	3	2	1	3	1	1	1	1
1	1	1	1	1	2	1	2	1	1
1	4	1	4	1	2	1	1	1	1
1	1	1	2	1	2	1	2	1	1
1	5	1	1	1	2	1	1	1	1
1	1	1	1	1	2	1	1	1	1
1	2	1	1	1	2	1	1	1	1
2	10	10	10	10	5	10	10	10	7

Cluster	ClumpThickness	CellSize	CellShape	MarginalAdhesion	EpithelialSize	BareNuclei	BlandChromatin	NormalNucleoli	Mitoses
1	5	1	1	1	2	1	3	2	1
1	1	1	1	1	2	1	1	1	1
1	1	1	1	1	2	1	1	1	1
1	1	1	1	1	2	1	1	1	1
1	1	1	1	1	2	1	1	1	1
1	3	1	1	1	2	1	2	3	1
1	4	1	1	1	2	1	1	1	1
1	1	1	1	1	2	1	1	1	8
2	5	10	10	5	4	5	4	4	1
1	3	1	1	1	2	1	1	1	1
1	3	1	1	1	2	1	2	1	2
1	2	1	1	1	2	1	1	1	1
2	5	10	10	3	7	3	8	10	2
2	4	8	6	4	3	4	10	6	1
2	4	8	8	5	4	5	10	4	1
1	1	1	1	1	2	3	3	1	1
1	2	1	1	1	2	1	2	1	1
2	5	6	5	6	10	1	3	1	1
1	1	1	1	1	2	2	2	1	1
2	9	4	5	10	6	10	4	8	1
1	4	1	1	2	2	1	2	1	1
2	10	10	10	10	10	1	8	8	8
2	7	3	4	4	3	3	3	2	7
2	5	3	5	1	8	10	5	3	1
1	4	1	1	1	2	1	2	1	1
1	1	1	1	1	2	5	1	1	1
1	3	1	1	3	8	1	5	8	1
2	10	8	10	10	6	1	3	1	10
2	8	7	6	4	4	10	5	1	1
1	2	1	1	1	1	1	3	1	1
1	8	4	4	5	4	7	7	8	2
1	1	1	1	1	2	1	1	1	1

Cluster	ClumpThickness	CellSize	CellShape	MarginalAdhesion	EpithelialSize	BareNuclei	BlandChromatin	NormalNucleoli	Mitoses
1	1	1	1	1	1	1	3	1	1
2	10	10	10	3	10	10	9	10	1
2	9	10	10	1	10	8	3	3	1
1	3	1	1	1	2	1	2	1	1
2	8	8	9	4	5	10	7	8	1
1	5	1	1	1	2	1	3	1	1
1	1	1	1	1	2	1	3	1	1
2	8	4	10	5	4	4	7	10	1
2	10	10	10	7	9	10	7	10	10
2	10	8	4	4	4	10	3	10	4
1	3	1	1	1	2	5	5	1	1
2	7	6	3	2	5	10	7	4	6
1	3	1	1	1	2	1	3	1	1
2	10	4	6	4	5	10	7	1	1
1	1	1	1	1	1	1	2	1	1
2	8	5	5	5	2	10	4	3	1
1	2	1	1	1	2	1	1	1	1
1	2	1	1	1	2	1	3	1	1
1	3	4	5	3	7	3	4	6	1
1	4	1	1	1	3	1	2	2	1
2	10	3	5	4	3	7	3	5	3
1	1	1	1	1	1	1	1	1	1
1	1	1	1	1	2	1	2	1	1
1	2	3	1	1	5	1	1	1	1
1	4	1	3	3	2	1	1	1	1
1	5	1	3	1	2	1	1	1	1
1	4	1	1	1	2	1	1	1	1
1	5	1	1	1	2	1	1	1	1
1	1	1	1	1	2	1	1	1	1
1	4	1	1	1	2	1	2	1	1
1	4	1	1	2	2	1	2	1	1
2	10	4	5	4	3	5	7	3	1

Cluster	ClumpThickness	CellSize	CellShape	MarginalAdhesion	EpithelialSize	BareNuclei	BlandChromatin	NormalNucleoli	Mitoses
1	4	3	1	1	2	1	4	8	1
2	10	10	8	10	6	5	10	3	1
2	7	6	10	5	3	10	9	10	2
1	1	1	1	1	2	1	2	1	1
1	5	1	2	1	2	1	2	1	1
1	1	1	1	1	2	1	1	1	1
2	10	4	3	10	3	10	7	1	2
1	6	2	3	1	2	1	1	1	1
1	5	1	1	1	2	1	2	1	1
1	3	1	4	1	2	1	1	1	1
1	1	1	1	1	2	1	1	1	1
1	1	2	2	1	2	1	1	1	1
1	3	1	1	2	3	4	1	1	1
1	3	1	1	1	2	1	2	1	1
1	2	1	1	1	2	1	3	1	1
2	5	10	10	10	4	10	5	6	3
1	1	1	1	3	2	1	1	1	1
1	3	1	1	1	3	2	1	1	1

Appendix Q

Newthyroid

Number of dimensions: 5

Number of clusters: 3

Number of points: 215

Patient is normal (1) or suffers from hyperthyroidism (2) or hypothyroidism (3)

Cluster	T3 resin	Thyroxin	Triiodothyronine	Thyroidstimulating	TSH_value
1	107	10.1	2.2	0.9	2.7
1	113	9.9	3.1	2	5.9
1	127	12.9	2.4	1.4	0.6
1	109	5.3	1.6	1.4	1.5
1	105	7.3	1.5	1.5	-0.1
1	105	6.1	2.1	1.4	7
1	110	10.4	1.6	1.6	2.7
1	114	9.9	2.4	1.5	5.7
1	106	9.4	2.2	1.5	0
1	107	13	1.1	0.9	3.1
1	106	4.2	1.2	1.6	1.4
1	110	11.3	2.3	0.9	3.3
1	116	9.2	2.7	1	4.2
1	112	8.1	1.9	3.7	2
1	122	9.7	1.6	0.9	2.2
1	109	8.4	2.1	1.1	3.6
1	111	8.4	1.5	0.8	1.2
1	114	6.7	1.5	1	3.5
1	119	10.6	2.1	1.3	1.1
1	115	7.1	1.3	1.3	2
1	101	7.8	1.2	1	1.7
1	103	10.1	1.3	0.7	0.1
1	109	10.4	1.9	0.4	-0.1
1	102	7.6	1.8	2	2.5
1	121	10.1	1.7	1.3	0.1
1	100	6.1	2.4	1.8	3.8
1	106	9.6	2.4	1	1.3
1	116	10.1	2.2	1.6	0.8
1	105	11.1	2	1	1
1	110	10.4	1.8	1	2.3
1	120	8.4	1.1	1.4	1.4
1	116	11.1	2	1.2	2.3
1	110	7.8	1.9	2.1	6.4
1	90	8.1	1.6	1.4	1.1
1	117	12.2	1.9	1.2	3.9
1	117	11	1.4	1.5	2.1
1	113	9	2	1.8	1.6
1	106	9.4	1.5	0.8	0.5
1	130	9.5	1.7	0.4	3.2

Cluster	T3 resin	Thyroxin	Triiodothyronine	Thyroidstimulating	TSH_value
1	100	10.5	2.4	0.9	1.9
1	121	10.1	2.4	0.8	3
1	110	9.2	1.6	1.5	0.3
1	129	11.9	2.7	1.2	3.5
1	121	13.5	1.5	1.6	0.5
1	123	8.1	2.3	1	5.1
1	107	8.4	1.8	1.5	0.8
1	109	10	1.3	1.8	4.3
1	120	6.8	1.9	1.3	1.9
1	100	9.5	2.5	1.3	-0.2
1	118	8.1	1.9	1.5	13.7
1	100	11.3	2.5	0.7	-0.3
1	103	12.2	1.2	1.3	2.7
1	115	8.1	1.7	0.6	2.2
1	119	8	2	0.6	3.2
1	106	9.4	1.7	0.9	3.1
1	114	10.9	2.1	0.3	1.4
1	93	8.9	1.5	0.8	2.7
1	120	10.4	2.1	1.1	1.8
1	106	11.3	1.8	0.9	1
1	110	8.7	1.9	1.6	4.4
1	103	8.1	1.4	0.5	3.8
1	101	7.1	2.2	0.8	2.2
1	115	10.4	1.8	1.6	2
1	116	10	1.7	1.5	4.3
1	117	9.2	1.9	1.5	6.8
1	106	6.7	1.5	1.2	3.9
1	118	10.5	2.1	0.7	3.5
1	97	7.8	1.3	1.2	0.9
1	113	11.1	1.7	0.8	2.3
1	104	6.3	2	1.2	4
1	96	9.4	1.5	1	3.1
1	120	12.4	2.4	0.8	1.9
1	133	9.7	2.9	0.8	1.9
1	126	9.4	2.3	1	4
1	113	8.5	1.8	0.8	0.5
1	109	9.7	1.4	1.1	2.1
1	119	12.9	1.5	1.3	3.6
1	101	7.1	1.6	1.5	1.6
1	108	10.4	2.1	1.3	2.4
1	117	6.7	2.2	1.8	6.7
1	115	15.3	2.3	2	2
1	91	8	1.7	2.1	4.6
1	103	8.5	1.8	1.9	1.1
1	98	9.1	1.4	1.9	-0.3

Cluster	T3 resin	Thyroxin	Triiodothyronine	Thyroidstimulating	TSH_value
1	111	7.8	2	1.8	4.1
1	107	13	1.5	2.8	1.7
1	119	11.4	2.3	2.2	1.6
1	122	11.8	2.7	1.7	2.3
1	105	8.1	2	1.9	-0.5
1	109	7.6	1.3	2.2	1.9
1	105	9.5	1.8	1.6	3.6
1	112	5.9	1.7	2	1.3
1	112	9.5	2	1.2	0.7
1	98	8.6	1.6	1.6	6
1	109	12.4	2.3	1.7	0.8
1	114	9.1	2.6	1.5	1.5
1	114	11.1	2.4	2	-0.3
1	110	8.4	1.4	1	1.9
1	120	7.1	1.2	1.5	4.3
1	108	10.9	1.2	1.9	1
1	108	8.7	1.2	2.2	2.5
1	116	11.9	1.8	1.9	1.5
1	113	11.5	1.5	1.9	2.9
1	105	7	1.5	2.7	4.3
1	114	8.4	1.6	1.6	-0.2
1	114	8.1	1.6	1.6	0.5
1	105	11.1	1.1	0.8	1.2
1	107	13.8	1.5	1	1.9
1	116	11.5	1.8	1.4	5.4
1	102	9.5	1.4	1.1	1.6
1	116	16.1	0.9	1.3	1.5
1	118	10.6	1.8	1.4	3
1	109	8.9	1.7	1	0.9
1	110	7	1	1.6	4.3
1	104	9.6	1.1	1.3	0.8
1	105	8.7	1.5	1.1	1.5
1	102	8.5	1.2	1.3	1.4
1	112	6.8	1.7	1.4	3.3
1	111	8.5	1.6	1.1	3.9
1	111	8.5	1.6	1.2	7.7
1	103	7.3	1	0.7	0.5
1	98	10.4	1.6	2.3	-0.7
1	117	7.8	2	1	3.9
1	111	9.1	1.7	1.2	4.1
1	101	6.3	1.5	0.9	2.9
1	106	8.9	0.7	1	2.3
1	102	8.4	1.5	0.8	2.4
1	115	10.6	0.8	2.1	4.6
1	130	10	1.6	0.9	4.6

Cluster	T3 resin	Thyroxin	Triiodothyronine	Thyroidstimulating	TSH_value
1	101	6.7	1.3	1	5.7
1	110	6.3	1	0.8	1
1	103	9.5	2.9	1.4	-0.1
1	113	7.8	2	1.1	3
1	112	10.6	1.6	0.9	-0.1
1	118	6.5	1.2	1.2	1.7
1	109	9.2	1.8	1.1	4.4
1	116	7.8	1.4	1.1	3.7
1	127	7.7	1.8	1.9	6.4
1	108	6.5	1	0.9	1.5
1	108	7.1	1.3	1.6	2.2
1	105	5.7	1	0.9	0.9
1	98	5.7	0.4	1.3	2.8
1	112	6.5	1.2	1.2	2
1	118	12.2	1.5	1	2.3
1	94	7.5	1.2	1.3	4.4
1	126	10.4	1.7	1.2	3.5
1	114	7.5	1.1	1.6	4.4
1	111	11.9	2.3	0.9	3.8
1	104	6.1	1.8	0.5	0.8
1	102	6.6	1.2	1.4	1.3
2	139	16.4	3.8	1.1	-0.2
2	111	16	2.1	0.9	-0.1
2	113	17.2	1.8	1	0
2	65	25.3	5.8	1.3	0.2
2	88	24.1	5.5	0.8	0.1
2	65	18.2	10	1.3	0.1
2	134	16.4	4.8	0.6	0.1
2	110	20.3	3.7	0.6	0.2
2	67	23.3	7.4	1.8	-0.6
2	95	11.1	2.7	1.6	-0.3
2	89	14.3	4.1	0.5	0.2
2	89	23.8	5.4	0.5	0.1
2	88	12.9	2.7	0.1	0.2
2	105	17.4	1.6	0.3	0.4
2	89	20.1	7.3	1.1	-0.2
2	99	13	3.6	0.7	-0.1
2	80	23	10	0.9	-0.1
2	89	21.8	7.1	0.7	-0.1
2	99	13	3.1	0.5	-0.1
2	68	14.7	7.8	0.6	-0.2
2	97	14.2	3.6	1.5	0.3
2	84	21.5	2.7	1.1	-0.6
2	84	18.5	4.4	1.1	-0.3
2	98	16.7	4.3	1.7	0.2

Cluster	T3 resin	Thyroxin	Triiodothyronine	Thyroidstimulating	TSH_value
2	94	20.5	1.8	1.4	-0.5
2	99	17.5	1.9	1.4	0.3
2	76	25.3	4.5	1.2	-0.1
2	110	15.2	1.9	0.7	-0.2
2	144	22.3	3.3	1.3	0.6
2	105	12	3.3	1.1	0
2	88	16.5	4.9	0.8	0.1
2	97	15.1	1.8	1.2	-0.2
2	106	13.4	3	1.1	0
2	79	19	5.5	0.9	0.3
2	92	11.1	2	0.7	-0.2
3	125	2.3	0.9	16.5	9.5
3	120	6.8	2.1	10.4	38.6
3	108	3.5	0.6	1.7	1.4
3	120	3	2.5	1.2	4.5
3	119	3.8	1.1	23	5.7
3	141	5.6	1.8	9.2	14.4
3	129	1.5	0.6	12.5	2.9
3	118	3.6	1.5	11.6	48.8
3	120	1.9	0.7	18.5	24
3	119	0.8	0.7	56.4	21.6
3	123	5.6	1.1	13.7	56.3
3	115	6.3	1.2	4.7	14.4
3	126	0.5	0.2	12.2	8.8
3	121	4.7	1.8	11.2	53
3	131	2.7	0.8	9.9	4.7
3	134	2	0.5	12.2	2.2
3	141	2.5	1.3	8.5	7.5
3	113	5.1	0.7	5.8	19.6
3	136	1.4	0.3	32.6	8.4
3	120	3.4	1.8	7.5	21.5
3	125	3.7	1.1	8.5	25.9
3	123	1.9	0.3	22.8	22.2
3	112	2.6	0.7	41	19
3	134	1.9	0.6	18.4	8.2
3	119	5.1	1.1	7	40.8
3	118	6.5	1.3	1.7	11.5
3	139	4.2	0.7	4.3	6.3
3	103	5.1	1.4	1.2	5
3	97	4.7	1.1	2.1	12.6
3	102	5.3	1.4	1.3	6.7

Appendix R

LiverDisorder

Number of dimensions: 6

Number of clusters: 2

Number of points: 345

No suffers from alcoholism (1) or suffers from alcoholism (2)

Cluster	Mcv	Alkphos	Sgpt	Sgot	Gammagt	Drinks
1	85	92	45	27	31	0
2	85	64	59	32	23	0
2	86	54	33	16	54	0
2	91	78	34	24	36	0
2	87	70	12	28	10	0
2	98	55	13	17	17	0
1	88	62	20	17	9	0.5
1	88	67	21	11	11	0.5
1	92	54	22	20	7	0.5
1	90	60	25	19	5	0.5
1	89	52	13	24	15	0.5
1	82	62	17	17	15	0.5
1	90	64	61	32	13	0.5
1	86	77	25	19	18	0.5
1	96	67	29	20	11	0.5
1	91	78	20	31	18	0.5
1	89	67	23	16	10	0.5
1	89	79	17	17	16	0.5
1	91	107	20	20	56	0.5
1	94	116	11	33	11	0.5
1	92	59	35	13	19	0.5
1	93	23	35	20	20	0.5
1	90	60	23	27	5	0.5
1	96	68	18	19	19	0.5
1	84	80	47	33	97	0.5
1	92	70	24	13	26	0.5
1	90	47	28	15	18	0.5
1	88	66	20	21	10	0.5
1	91	102	17	13	19	0.5
1	87	41	31	19	16	0.5
1	86	79	28	16	17	0.5
1	91	57	31	23	42	0.5
1	93	77	32	18	29	0.5
1	88	96	28	21	40	0.5
1	94	65	22	18	11	0.5
2	91	72	155	68	82	0.5
2	85	54	47	33	22	0.5
2	79	39	14	19	9	0.5
2	85	85	25	26	30	0.5

Cluster	Mcv	Alkphos	Sgpt	Sgot	Gammagt	Drinks
2	89	63	24	20	38	0.5
2	84	92	68	37	44	0.5
2	89	68	26	39	42	0.5
2	89	101	18	25	13	0.5
2	86	84	18	14	16	0.5
2	85	65	25	14	18	0.5
2	88	61	19	21	13	0.5
2	92	56	14	16	10	0.5
2	95	50	29	25	50	0.5
2	91	75	24	22	11	0.5
2	83	40	29	25	38	0.5
2	89	74	19	23	16	0.5
2	85	64	24	22	11	0.5
2	92	57	64	36	90	0.5
2	94	48	11	23	43	0.5
2	87	52	21	19	30	0.5
2	85	65	23	29	15	0.5
2	84	82	21	21	19	0.5
2	88	49	20	22	19	0.5
2	96	67	26	26	36	0.5
2	90	63	24	24	24	0.5
2	90	45	33	34	27	0.5
2	90	72	14	15	18	0.5
2	91	55	4	8	13	0.5
2	91	52	15	22	11	0.5
1	87	71	32	19	27	1
1	89	77	26	20	19	1
2	89	67	5	17	14	1
2	85	51	26	24	23	1
2	103	75	19	30	13	1
2	90	63	16	21	14	1
1	90	63	29	23	57	2
1	90	67	35	19	35	2
1	87	66	27	22	9	2
1	90	73	34	21	22	2
1	86	54	20	21	16	2
1	90	80	19	14	42	2
2	87	90	43	28	156	2
2	96	72	28	19	30	2
2	91	55	9	25	16	2
2	95	78	27	25	30	2
2	92	101	34	30	64	2
2	89	51	41	22	48	2
2	91	99	42	33	16	2
2	94	58	21	18	26	2

Cluster	Mcv	Alkphos	Sgpt	Sgot	Gammagt	Drinks
2	92	60	30	27	297	2
2	94	58	21	18	26	2
2	88	47	33	26	29	2
2	92	65	17	25	9	2
1	92	79	22	20	11	3
1	84	83	20	25	7	3
1	88	68	27	21	26	3
1	86	48	20	20	6	3
1	99	69	45	32	30	3
1	88	66	23	12	15	3
1	89	62	42	30	20	3
1	90	51	23	17	27	3
2	81	61	32	37	53	3
2	89	89	23	18	104	3
2	89	65	26	18	36	3
2	92	75	26	26	24	3
2	85	59	25	20	25	3
2	92	61	18	13	81	3
1	89	63	22	27	10	4
1	90	84	18	23	13	4
1	88	95	25	19	14	4
1	89	35	27	29	17	4
1	91	80	37	23	27	4
1	91	109	33	15	18	4
1	91	65	17	5	7	4
2	88	107	29	20	50	4
2	87	76	22	55	9	4
2	87	86	28	23	21	4
2	87	42	26	23	17	4
2	88	80	24	25	17	4
2	90	96	34	49	169	4
2	86	67	11	15	8	4
2	92	40	19	20	21	4
2	85	60	17	21	14	4
2	89	90	15	17	25	4
2	91	57	15	16	16	4
2	96	55	48	39	42	4
2	79	101	17	27	23	4
2	90	134	14	20	14	4
2	89	76	14	21	24	4
2	88	93	29	27	31	4
2	90	67	10	16	16	4
2	92	73	24	21	48	4
2	91	55	28	28	82	4
2	83	45	19	21	13	4

Cluster	Mcv	Alkphos	Sgpt	Sgot	Gammagt	Drinks
2	90	74	19	14	22	4
1	92	66	21	16	33	5
1	93	63	26	18	18	5
2	86	78	47	39	107	5
2	97	44	113	45	150	5
2	87	59	15	19	12	5
2	86	44	21	11	15	5
2	87	64	16	20	24	5
2	92	57	21	23	22	5
2	90	70	25	23	112	5
2	99	59	17	19	11	5
1	92	80	10	26	20	6
1	95	60	26	22	28	6
1	91	63	25	26	15	6
1	92	62	37	21	36	6
1	95	50	13	14	15	6
1	90	76	37	19	50	6
1	96	70	70	26	36	6
1	95	62	64	42	76	6
1	92	62	20	23	20	6
1	91	63	25	26	15	6
2	82	56	67	38	92	6
2	92	82	27	24	37	6
2	90	63	12	26	21	6
2	88	37	9	15	16	6
2	100	60	29	23	76	6
2	98	43	35	23	69	6
2	91	74	87	50	67	6
2	92	87	57	25	44	6
2	93	99	36	34	48	6
2	90	72	17	19	19	6
2	97	93	21	20	68	6
2	93	50	18	25	17	6
2	90	57	20	26	33	6
2	92	76	31	28	41	6
2	88	55	19	17	14	6
2	89	63	24	29	29	6
1	92	79	70	32	84	7
1	92	93	58	35	120	7
2	93	84	58	47	62	7
1	97	71	29	22	52	8
1	84	99	33	19	26	8
1	96	44	42	23	73	8
1	90	62	22	21	21	8
1	92	94	18	17	6	8

Cluster	Mcv	Alkphos	Sgpt	Sgot	Gammagt	Drinks
1	90	67	77	39	114	8
1	97	71	29	22	52	8
2	91	69	25	25	66	8
2	93	59	17	20	14	8
2	92	95	85	48	200	8
2	90	50	26	22	53	8
2	91	62	59	47	60	8
1	92	93	22	28	123	9
1	92	77	86	41	31	10
2	86	66	22	24	26	10
2	98	57	31	34	73	10
2	95	80	50	64	55	10
2	92	108	53	33	94	12
2	97	92	22	28	49	12
1	93	77	39	37	108	16
1	94	83	81	34	201	20
1	87	75	25	21	14	0
1	88	56	23	18	12	0
2	84	97	41	20	32	0
1	94	91	27	20	15	0.5
1	97	62	17	13	5	0.5
1	92	85	25	20	12	0.5
1	82	48	27	15	12	0.5
1	88	74	31	25	15	0.5
1	95	77	30	14	21	0.5
1	88	94	26	18	8	0.5
1	91	70	19	19	22	0.5
1	83	54	27	15	12	0.5
1	91	105	40	26	56	0.5
1	86	79	37	28	14	0.5
1	91	96	35	22	135	0.5
1	89	82	23	14	35	0.5
1	90	73	24	23	11	0.5
1	90	87	19	25	19	0.5
1	89	82	33	32	18	0.5
1	85	79	17	8	9	0.5
1	85	119	30	26	17	0.5
1	78	69	24	18	31	0.5
1	88	107	34	21	27	0.5
1	89	115	17	27	7	0.5
1	92	67	23	15	12	0.5
1	89	101	27	34	14	0.5
1	91	84	11	12	10	0.5
2	94	101	41	20	53	0.5
2	88	46	29	22	18	0.5

Cluster	Mcv	Alkphos	Sgpt	Sgot	Gammagt	Drinks
2	88	122	35	29	42	0.5
2	84	88	28	25	35	0.5
2	90	79	18	15	24	0.5
2	87	69	22	26	11	0.5
2	65	63	19	20	14	0.5
2	90	64	12	17	14	0.5
2	85	58	18	24	16	0.5
2	88	81	41	27	36	0.5
2	86	78	52	29	62	0.5
2	82	74	38	28	48	0.5
2	86	58	36	27	59	0.5
2	94	56	30	18	27	0.5
2	87	57	30	30	22	0.5
2	98	74	148	75	159	0.5
2	94	75	20	25	38	0.5
2	83	68	17	20	71	0.5
2	93	56	25	21	33	0.5
2	101	65	18	21	22	0.5
2	92	65	25	20	31	0.5
2	92	58	14	16	13	0.5
2	86	58	16	23	23	0.5
2	85	62	15	13	22	0.5
2	86	57	13	20	13	0.5
2	86	54	26	30	13	0.5
1	81	41	33	27	34	1
1	91	67	32	26	13	1
1	91	80	21	19	14	1
1	92	60	23	15	19	1
1	91	60	32	14	8	1
1	93	65	28	22	10	1
2	90	63	45	24	85	1
2	87	92	21	22	37	1
2	83	78	31	19	115	1
2	95	62	24	23	14	1
2	93	59	41	30	48	1
1	84	82	43	32	38	2
1	87	71	33	20	22	2
1	86	44	24	15	18	2
1	86	66	28	24	21	2
1	88	58	31	17	17	2
1	90	61	28	29	31	2
1	88	69	70	24	64	2
1	93	87	18	17	26	2
1	98	58	33	21	28	2
2	91	44	18	18	23	2

Cluster	Mcv	Alkphos	Sgpt	Sgot	Gammagt	Drinks
2	87	75	37	19	70	2
2	94	91	30	26	25	2
2	88	85	14	15	10	2
2	89	109	26	25	27	2
2	87	59	37	27	34	2
2	93	58	20	23	18	2
2	88	57	9	15	16	2
1	94	65	38	27	17	3
1	91	71	12	22	11	3
1	90	55	20	20	16	3
2	91	64	21	17	26	3
2	88	47	35	26	33	3
2	82	72	31	20	84	3
2	85	58	83	49	51	3
1	91	54	25	22	35	4
2	98	50	27	25	53	4
2	86	62	29	21	26	4
2	89	48	32	22	14	4
2	82	68	20	22	9	4
2	83	70	17	19	23	4
2	96	70	21	26	21	4
2	94	117	77	56	52	4
2	93	45	11	14	21	4
2	93	49	27	21	29	4
2	84	73	46	32	39	4
2	91	63	17	17	46	4
2	90	57	31	18	37	4
2	87	45	19	13	16	4
2	91	68	14	20	19	4
2	86	55	29	35	108	4
2	91	86	52	47	52	4
2	88	46	15	33	55	4
2	85	52	22	23	34	4
2	89	72	33	27	55	4
2	95	59	23	18	19	4
2	94	43	154	82	121	4
2	96	56	38	26	23	5
2	90	52	10	17	12	5
2	94	45	20	16	12	5
2	99	42	14	21	49	5
2	93	102	47	23	37	5
2	94	71	25	26	31	5
2	92	73	33	34	115	5
1	87	54	41	29	23	6
1	92	67	15	14	14	6

Cluster	Mcv	Alkphos	Sgpt	Sgot	Gammagt	Drinks
1	98	101	31	26	32	6
1	92	53	51	33	92	6
1	97	94	43	43	82	6
1	93	43	11	16	54	6
1	93	68	24	18	19	6
1	95	36	38	19	15	6
1	99	86	58	42	203	6
1	98	66	103	57	114	6
1	92	80	10	26	20	6
2	96	74	27	25	43	6
2	95	93	21	27	47	6
2	86	109	16	22	28	6
2	91	46	30	24	39	7
2	102	82	34	78	203	7
2	85	50	12	18	14	7
1	91	57	33	23	12	8
1	91	52	76	32	24	8
1	93	70	46	30	33	8
1	87	55	36	19	25	8
1	98	123	28	24	31	8
2	82	55	18	23	44	8
2	95	73	20	25	225	8
2	97	80	17	20	53	8
2	100	83	25	24	28	8
2	88	91	56	35	126	9
1	91	138	45	21	48	10
1	92	41	37	22	37	10
2	86	123	20	25	23	10
2	91	93	35	34	37	10
2	87	87	15	23	11	10
2	87	56	52	43	55	10
1	99	75	26	24	41	12
2	96	69	53	43	203	12
1	98	77	55	35	89	15
1	91	68	27	26	14	16
1	98	99	57	45	65	20

Appendix S

Glass
Number of dimensions: 9
Number of clusters: 6
Number of points: 214
1 building windows float
2 building windows non float
3 vehicle windows float
4 containers
5 tableware
6 headlamps

Cluster	RI	Na	Mg	Al	Si	K	Ca	Ba	Fe
1	1.52101	13.64	4.49	1.1	71.78	0.06	8.75	0	0
1	1.51761	13.89	3.6	1.36	72.73	0.48	7.83	0	0
1	1.51618	13.53	3.55	1.54	72.99	0.39	7.78	0	0
1	1.51766	13.21	3.69	1.29	72.61	0.57	8.22	0	0
1	1.51742	13.27	3.62	1.24	73.08	0.55	8.07	0	0
1	1.51596	12.79	3.61	1.62	72.97	0.64	8.07	0	0.26
1	1.51743	13.3	3.6	1.14	73.09	0.58	8.17	0	0
1	1.51756	13.15	3.61	1.05	73.24	0.57	8.24	0	0
1	1.51918	14.04	3.58	1.37	72.08	0.56	8.3	0	0
1	1.51755	13	3.6	1.36	72.99	0.57	8.4	0	0.11
1	1.51571	12.72	3.46	1.56	73.2	0.67	8.09	0	0.24
1	1.51763	12.8	3.66	1.27	73.01	0.6	8.56	0	0
1	1.51589	12.88	3.43	1.4	73.28	0.69	8.05	0	0.24
1	1.51748	12.86	3.56	1.27	73.21	0.54	8.38	0	0.17
1	1.51763	12.61	3.59	1.31	73.29	0.58	8.5	0	0
1	1.51761	12.81	3.54	1.23	73.24	0.58	8.39	0	0
1	1.51784	12.68	3.67	1.16	73.11	0.61	8.7	0	0
1	1.52196	14.36	3.85	0.89	71.36	0.15	9.15	0	0
1	1.51911	13.9	3.73	1.18	72.12	0.06	8.89	0	0
1	1.51735	13.02	3.54	1.69	72.73	0.54	8.44	0	0.07
1	1.5175	12.82	3.55	1.49	72.75	0.54	8.52	0	0.19
1	1.51966	14.77	3.75	0.29	72.02	0.03	9	0	0
1	1.51736	12.78	3.62	1.29	72.79	0.59	8.7	0	0
1	1.51751	12.81	3.57	1.35	73.02	0.62	8.59	0	0
1	1.5172	13.38	3.5	1.15	72.85	0.5	8.43	0	0
1	1.51764	12.98	3.54	1.21	73	0.65	8.53	0	0
1	1.51793	13.21	3.48	1.41	72.64	0.59	8.43	0	0
1	1.51721	12.87	3.48	1.33	73.04	0.56	8.43	0	0
1	1.51768	12.56	3.52	1.43	73.15	0.57	8.54	0	0
1	1.51784	13.08	3.49	1.28	72.86	0.6	8.49	0	0
1	1.51768	12.65	3.56	1.3	73.08	0.61	8.69	0	0.14
1	1.51747	12.84	3.5	1.14	73.27	0.56	8.55	0	0
1	1.51775	12.85	3.48	1.23	72.97	0.61	8.56	0.09	0.22
1	1.51753	12.57	3.47	1.38	73.39	0.6	8.55	0	0.06
1	1.51783	12.69	3.54	1.34	72.95	0.57	8.75	0	0
1	1.51567	13.29	3.45	1.21	72.74	0.56	8.57	0	0
1	1.51909	13.89	3.53	1.32	71.81	0.51	8.78	0.11	0

Cluster	RI	Na	Mg	Al	Si	K	Ca	Ba	Fe
1	1.51797	12.74	3.48	1.35	72.96	0.64	8.68	0	0
1	1.52213	14.21	3.82	0.47	71.77	0.11	9.57	0	0
1	1.52213	14.21	3.82	0.47	71.77	0.11	9.57	0	0
1	1.51793	12.79	3.5	1.12	73.03	0.64	8.77	0	0
1	1.51755	12.71	3.42	1.2	73.2	0.59	8.64	0	0
1	1.51779	13.21	3.39	1.33	72.76	0.59	8.59	0	0
1	1.5221	13.73	3.84	0.72	71.76	0.17	9.74	0	0
1	1.51786	12.73	3.43	1.19	72.95	0.62	8.76	0	0.3
1	1.519	13.49	3.48	1.35	71.95	0.55	9	0	0
1	1.51869	13.19	3.37	1.18	72.72	0.57	8.83	0	0.16
1	1.52667	13.99	3.7	0.71	71.57	0.02	9.82	0	0.1
1	1.52223	13.21	3.77	0.79	71.99	0.13	10.02	0	0
1	1.51898	13.58	3.35	1.23	72.08	0.59	8.91	0	0
1	1.5232	13.72	3.72	0.51	71.75	0.09	10.06	0	0.16
1	1.51926	13.2	3.33	1.28	72.36	0.6	9.14	0	0.11
1	1.51808	13.43	2.87	1.19	72.84	0.55	9.03	0	0
1	1.51837	13.14	2.84	1.28	72.85	0.55	9.07	0	0
1	1.51778	13.21	2.81	1.29	72.98	0.51	9.02	0	0.09
1	1.51769	12.45	2.71	1.29	73.7	0.56	9.06	0	0.24
1	1.51215	12.99	3.47	1.12	72.98	0.62	8.35	0	0.31
1	1.51824	12.87	3.48	1.29	72.95	0.6	8.43	0	0
1	1.51754	13.48	3.74	1.17	72.99	0.59	8.03	0	0
1	1.51754	13.39	3.66	1.19	72.79	0.57	8.27	0	0.11
1	1.51905	13.6	3.62	1.11	72.64	0.14	8.76	0	0
1	1.51977	13.81	3.58	1.32	71.72	0.12	8.67	0.69	0
1	1.52172	13.51	3.86	0.88	71.79	0.23	9.54	0	0.11
1	1.52227	14.17	3.81	0.78	71.35	0	9.69	0	0
1	1.52172	13.48	3.74	0.9	72.01	0.18	9.61	0	0.07
1	1.52099	13.69	3.59	1.12	71.96	0.09	9.4	0	0
1	1.52152	13.05	3.65	0.87	72.22	0.19	9.85	0	0.17
1	1.52152	13.05	3.65	0.87	72.32	0.19	9.85	0	0.17
1	1.52152	13.12	3.58	0.9	72.2	0.23	9.82	0	0.16
1	1.523	13.31	3.58	0.82	71.99	0.12	10.17	0	0.03
2	1.51574	14.86	3.67	1.74	71.87	0.16	7.36	0	0.12
2	1.51848	13.64	3.87	1.27	71.96	0.54	8.32	0	0.32
2	1.51593	13.09	3.59	1.52	73.1	0.67	7.83	0	0
2	1.51631	13.34	3.57	1.57	72.87	0.61	7.89	0	0
2	1.51596	13.02	3.56	1.54	73.11	0.72	7.9	0	0
2	1.5159	13.02	3.58	1.51	73.12	0.69	7.96	0	0
2	1.51645	13.44	3.61	1.54	72.39	0.66	8.03	0	0
2	1.51627	13	3.58	1.54	72.83	0.61	8.04	0	0
2	1.51613	13.92	3.52	1.25	72.88	0.37	7.94	0	0.14
2	1.5159	12.82	3.52	1.9	72.86	0.69	7.97	0	0
2	1.51592	12.86	3.52	2.12	72.66	0.69	7.97	0	0
2	1.51593	13.25	3.45	1.43	73.17	0.61	7.86	0	0
2	1.51646	13.41	3.55	1.25	72.81	0.68	8.1	0	0
2	1.51594	13.09	3.52	1.55	72.87	0.68	8.05	0	0.09
2	1.51409	14.25	3.09	2.08	72.28	1.1	7.08	0	0
2	1.51625	13.36	3.58	1.49	72.72	0.45	8.21	0	0

Cluster	RI	Na	Mg	Al	Si	K	Ca	Ba	Fe
2	1.51569	13.24	3.49	1.47	73.25	0.38	8.03	0	0
2	1.51645	13.4	3.49	1.52	72.65	0.67	8.08	0	0.1
2	1.51618	13.01	3.5	1.48	72.89	0.6	8.12	0	0
2	1.5164	12.55	3.48	1.87	73.23	0.63	8.08	0	0.09
2	1.51841	12.93	3.74	1.11	72.28	0.64	8.96	0	0.22
2	1.51605	12.9	3.44	1.45	73.06	0.44	8.27	0	0
2	1.51588	13.12	3.41	1.58	73.26	0.07	8.39	0	0.19
2	1.5159	13.24	3.34	1.47	73.1	0.39	8.22	0	0
2	1.51629	12.71	3.33	1.49	73.28	0.67	8.24	0	0
2	1.5186	13.36	3.43	1.43	72.26	0.51	8.6	0	0
2	1.51841	13.02	3.62	1.06	72.34	0.64	9.13	0	0.15
2	1.51743	12.2	3.25	1.16	73.55	0.62	8.9	0	0.24
2	1.51689	12.67	2.88	1.71	73.21	0.73	8.54	0	0
2	1.51811	12.96	2.96	1.43	72.92	0.6	8.79	0.14	0
2	1.51655	12.75	2.85	1.44	73.27	0.57	8.79	0.11	0.22
2	1.5173	12.35	2.72	1.63	72.87	0.7	9.23	0	0
2	1.5182	12.62	2.76	0.83	73.81	0.35	9.42	0	0.2
2	1.52725	13.8	3.15	0.66	70.57	0.08	11.64	0	0
2	1.5241	13.83	2.9	1.17	71.15	0.08	10.79	0	0
2	1.52475	11.45	0	1.88	72.19	0.81	13.24	0	0.34
2	1.53125	10.73	0	2.1	69.81	0.58	13.3	3.15	0.28
2	1.53393	12.3	0	1	70.16	0.12	16.19	0	0.24
2	1.52222	14.43	0	1	72.67	0.1	11.52	0	0.08
2	1.51818	13.72	0	0.56	74.45	0	10.99	0	0
2	1.52664	11.23	0	0.77	73.21	0	14.68	0	0
2	1.52739	11.02	0	0.75	73.08	0	14.96	0	0
2	1.52777	12.64	0	0.67	72.02	0.06	14.4	0	0
2	1.51892	13.46	3.83	1.26	72.55	0.57	8.21	0	0.14
2	1.51847	13.1	3.97	1.19	72.44	0.6	8.43	0	0
2	1.51846	13.41	3.89	1.33	72.38	0.51	8.28	0	0
2	1.51829	13.24	3.9	1.41	72.33	0.55	8.31	0	0.1
2	1.51708	13.72	3.68	1.81	72.06	0.64	7.88	0	0
2	1.51673	13.3	3.64	1.53	72.53	0.65	8.03	0	0.29
2	1.51652	13.56	3.57	1.47	72.45	0.64	7.96	0	0
2	1.51844	13.25	3.76	1.32	72.4	0.58	8.42	0	0
2	1.51663	12.93	3.54	1.62	72.96	0.64	8.03	0	0.21
2	1.51687	13.23	3.54	1.48	72.84	0.56	8.1	0	0
2	1.51707	13.48	3.48	1.71	72.52	0.62	7.99	0	0
2	1.52177	13.2	3.68	1.15	72.75	0.54	8.52	0	0
2	1.51872	12.93	3.66	1.56	72.51	0.58	8.55	0	0.12
2	1.51667	12.94	3.61	1.26	72.75	0.56	8.6	0	0
2	1.52081	13.78	2.28	1.43	71.99	0.49	9.85	0	0.17
2	1.52068	13.55	2.09	1.67	72.18	0.53	9.57	0.27	0.17
2	1.5202	13.98	1.35	1.63	71.76	0.39	10.56	0	0.18
2	1.52177	13.75	1.01	1.36	72.19	0.33	11.14	0	0
2	1.52614	13.7	0	1.36	71.24	0.19	13.44	0	0.1
2	1.51813	13.43	3.98	1.18	72.49	0.58	8.15	0	0
2	1.518	13.71	3.93	1.54	71.81	0.54	8.21	0	0.15
2	1.51811	13.33	3.85	1.25	72.78	0.52	8.12	0	0

Cluster	RI	Na	Mg	Al	Si	K	Ca	Ba	Fe
2	1.51789	13.19	3.9	1.3	72.33	0.55	8.44	0	0.28
2	1.51806	13	3.8	1.08	73.07	0.56	8.38	0	0.12
2	1.51711	12.89	3.62	1.57	72.96	0.61	8.11	0	0
2	1.51674	12.79	3.52	1.54	73.36	0.66	7.9	0	0
2	1.51674	12.87	3.56	1.64	73.14	0.65	7.99	0	0
2	1.5169	13.33	3.54	1.61	72.54	0.68	8.11	0	0
2	1.51851	13.2	3.63	1.07	72.83	0.57	8.41	0.09	0.17
2	1.51662	12.85	3.51	1.44	73.01	0.68	8.23	0.06	0.25
2	1.51709	13	3.47	1.79	72.72	0.66	8.18	0	0
2	1.5166	12.99	3.18	1.23	72.97	0.58	8.81	0	0.24
2	1.51839	12.85	3.67	1.24	72.57	0.62	8.68	0	0.35
3	1.51769	13.65	3.66	1.11	72.77	0.11	8.6	0	0
3	1.5161	13.33	3.53	1.34	72.67	0.56	8.33	0	0
3	1.5167	13.24	3.57	1.38	72.7	0.56	8.44	0	0.1
3	1.51643	12.16	3.52	1.35	72.89	0.57	8.53	0	0
3	1.51665	13.14	3.45	1.76	72.48	0.6	8.38	0	0.17
3	1.52127	14.32	3.9	0.83	71.5	0	9.49	0	0
3	1.51779	13.64	3.65	0.65	73	0.06	8.93	0	0
3	1.5161	13.42	3.4	1.22	72.69	0.59	8.32	0	0
3	1.51694	12.86	3.58	1.31	72.61	0.61	8.79	0	0
3	1.51646	13.04	3.4	1.26	73.01	0.52	8.58	0	0
3	1.51655	13.41	3.39	1.28	72.64	0.52	8.65	0	0
3	1.52121	14.03	3.76	0.58	71.79	0.11	9.65	0	0
3	1.51776	13.53	3.41	1.52	72.04	0.58	8.79	0	0
3	1.51796	13.5	3.36	1.63	71.94	0.57	8.81	0	0.09
3	1.51832	13.33	3.34	1.54	72.14	0.56	8.99	0	0
3	1.51934	13.64	3.54	0.75	72.65	0.16	8.89	0.15	0.24
3	1.52211	14.19	3.78	0.91	71.36	0.23	9.14	0	0.37
4	1.51514	14.01	2.68	3.5	69.89	1.68	5.87	2.2	0
4	1.51915	12.73	1.85	1.86	72.69	0.6	10.09	0	0
4	1.52171	11.56	1.88	1.56	72.86	0.47	11.41	0	0
4	1.52151	11.03	1.71	1.56	73.44	0.58	11.62	0	0
4	1.51969	12.64	0	1.65	73.75	0.38	11.53	0	0
4	1.51666	12.86	0	1.83	73.88	0.97	10.17	0	0
4	1.51994	13.27	0	1.76	73.03	0.47	11.32	0	0
4	1.52369	13.44	0	1.58	72.22	0.32	12.24	0	0
4	1.51316	13.02	0	3.04	70.48	6.21	6.96	0	0
4	1.51321	13	0	3.02	70.7	6.21	6.93	0	0
4	1.52043	13.38	0	1.4	72.25	0.33	12.5	0	0
4	1.52058	12.85	1.61	2.17	72.18	0.76	9.7	0.24	0.51
4	1.52119	12.97	0.33	1.51	73.39	0.13	11.27	0	0.28
5	1.51905	14	2.39	1.56	72.37	0	9.57	0	0
5	1.51937	13.79	2.41	1.19	72.76	0	9.77	0	0
5	1.51829	14.46	2.24	1.62	72.38	0	9.26	0	0
5	1.51852	14.09	2.19	1.66	72.67	0	9.32	0	0
5	1.51299	14.4	1.74	1.54	74.55	0	7.59	0	0
5	1.51888	14.99	0.78	1.74	72.5	0	9.95	0	0
5	1.51916	14.15	0	2.09	72.74	0	10.88	0	0
5	1.51969	14.56	0	0.56	73.48	0	11.22	0	0

Cluster	RI	Na	Mg	Al	Si	K	Ca	Ba	Fe
5	1.51115	17.38	0	0.34	75.41	0	6.65	0	0
6	1.51131	13.69	3.2	1.81	72.81	1.76	5.43	1.19	0
6	1.51838	14.32	3.26	2.22	71.25	1.46	5.79	1.63	0
6	1.52315	13.44	3.34	1.23	72.38	0.6	8.83	0	0
6	1.52247	14.86	2.2	2.06	70.26	0.76	9.76	0	0
6	1.52365	15.79	1.83	1.31	70.43	0.31	8.61	1.68	0
6	1.51613	13.88	1.78	1.79	73.1	0	8.67	0.76	0
6	1.51602	14.85	0	2.38	73.28	0	8.76	0.64	0.09
6	1.51623	14.2	0	2.79	73.46	0.04	9.04	0.4	0.09
6	1.51719	14.75	0	2	73.02	0	8.53	1.59	0.08
6	1.51683	14.56	0	1.98	73.29	0	8.52	1.57	0.07
6	1.51545	14.14	0	2.68	73.39	0.08	9.07	0.61	0.05
6	1.51556	13.87	0	2.54	73.23	0.14	9.41	0.81	0.01
6	1.51727	14.7	0	2.34	73.28	0	8.95	0.66	0
6	1.51531	14.38	0	2.66	73.1	0.04	9.08	0.64	0
6	1.51609	15.01	0	2.51	73.05	0.05	8.83	0.53	0
6	1.51508	15.15	0	2.25	73.5	0	8.34	0.63	0
6	1.51653	11.95	0	1.19	75.18	2.7	8.93	0	0
6	1.51514	14.85	0	2.42	73.72	0	8.39	0.56	0
6	1.51658	14.8	0	1.99	73.11	0	8.28	1.71	0
6	1.51617	14.95	0	2.27	73.3	0	8.71	0.67	0
6	1.51732	14.95	0	1.8	72.99	0	8.61	1.55	0
6	1.51645	14.94	0	1.87	73.11	0	8.67	1.38	0
6	1.51831	14.39	0	1.82	72.86	1.41	6.47	2.88	0
6	1.5164	14.37	0	2.74	72.85	0	9.45	0.54	0
6	1.51623	14.14	0	2.88	72.61	0.08	9.18	1.06	0
6	1.51685	14.92	0	1.99	73.06	0	8.4	1.59	0
6	1.52065	14.36	0	2.02	73.42	0	8.44	1.64	0
6	1.51651	14.38	0	1.94	73.61	0	8.48	1.57	0
6	1.51711	14.23	0	2.08	73.36	0	8.62	1.67	0
